# Identification of immunization-related new prognostic biomarkers for papillary renal cell carcinoma by integrated bioinformatics analysis

**DOI:** 10.1186/s12920-021-01092-w

**Published:** 2021-10-07

**Authors:** Ping Wu, Tingting Xiang, Jing Wang, Run Lv, Shaoxin Ma, Limei Yuan, Guangzhen Wu, Xiangyu Che

**Affiliations:** 1 Department of Anesthesiology, The First Affiliated Hospital of Dalian Medical University, Dalian, 116000 China; 2 Department of Rehabilitation, Liguang Rehabilitation Hospital of Dalian Development Zone, Dalian, 116600 China; 3 Department of Neurobiology, Harbin Medical University, Harbin, 150086 China; 4 Department of Anesthesiology, Dalian Medical University, Dalian, 116044 China; 5 Department of Urology, The First Affiliated Hospital of Dalian Medical University, Dalian, 116000 China

**Keywords:** Carcinoma, Renal cell, Computational biology, Prolyl hydroxylases, Biological markers, Immunotherapy

## Abstract

**Background:**

Despite papillary renal cell carcinoma (pRCC) being the second most common type of kidney cancer, the underlying molecular mechanism remains unclear. Targeted therapies in the past have not been successful because of the lack of a clear understanding of the molecular mechanism. Hence, exploring the underlying mechanisms and seeking novel biomarkers for pursuing a precise prognostic biomarker and appropriate therapies are critical.

**Material and methods:**

In our research, the differentially expressed genes (DEGs) were screened from the TCGA and GEO databases, and a total of 149 upregulated and 285 downregulated genes were sorted. This was followed by construction of functional enrichment and protein–protein interaction (PPI) network, and then the top 15 DEGs were selected for further analysis. The *P4HB* gene was chosen as our target gene by repetitively validating multiple datasets, and higher levels of *P4HB* expression predicted lower overall survival (OS) in patients with pRCC.

**Results:**

We found that *P4HB* not only connects with immune cell infiltration and co-expression with *PD-1, PD-L2,* and *CTLA-4*, but also has a strong connection with the newly discovered hot gene, *TOX*.

**Conclusion:**

We speculate that *P4HB* is a novel gene involved in the progression of pRCC through immunomodulation.

**Supplementary Information:**

The online version contains supplementary material available at 10.1186/s12920-021-01092-w.

## Introduction

The incidence of kidney cancer is growing globally, with approximately 338,000 people diagnosed annually, resulting in 143,000 deaths [1]. Renal cancer mainly includes three histological types: clear cell carcinoma (70%), papillary cell carcinoma (10–15%), and chromophobe cell carcinoma (5%) [2]. Papillary renal cell carcinoma (pRCC) is a renal parenchymal malignancy. The histological features include the presence of papillary or tubular capillary structures and are classified as type 1 and type 2 papillary cell carcinoma [3]. It is now accepted that pRCC is indolent and multifocal, while in some cases, it has an aggressive and lethal tumor phenotype. No effective treatment has been found to date. *MET, SETD2, NF2, KDM6A, SMARCB1, FAT1, BAP1, PBRM1, STAG2, NFE2L2*, and *TP53* mutations have been reported in pRCC [4, 5]. However, the number of pRCC cases is limited, and patients with pRCC are often excluded from genetic testing and randomized clinical trials of kidney cancer [6]. Therefore, further study of the molecular spectrum of pRCC may provide a new target for prognosis and further treatment.

With the help of gene sequencing technology, a number of messenger RNAs (mRNAs) have been discovered in cancer. Research has shown that mRNA is closely related to the biological development of cells. For example, it plays a role in cell differentiation, proliferation, apoptosis, invasion, migration, and immune escape [7]. Generally, a change in a gene triggers a series of changes in the expression of its associated genes, which could regulate the biological behavior of the cell in different regulatory ways such as network regulation, which is usually defined as a “cancer panel” [8]. Using bioinformatics analysis methods, genes in the “cancer panel” involving similar biological functions can be analyzed. These genes can also affect biological behavior through the same or through different signaling pathways [9]. Identification of the association between these genes and their interaction networks allows us to demonstrate better the mechanism by which the target genes in the present study cause the development and progression of pRCC.

Immunotherapy is a hot topic in cancer treatment currently. Moreover, programmed death-1 (PD-1)/programmed death-ligand 1 (PD-L1) inhibitors, is becoming a favorable novel treatment for pRCC. PD-1/PD-L1 blockade showed modest anti-tumor activity in pRCC with a response rate of approximately 30%, which is much higher than that in other clear cell types [10]. However, feasible immune biomarkers for predicting the prognosis of patients with pRCC and possible new immunotherapeutic targets for pRCC treatment are few. Therefore, it is imperative to find a robust immune signature of pRCC that can serve as a predictor of survival in patients with pRCC from a tumor immunology perspective. Thus, this is a new target for pRCC immunotherapy [11, 12].

In our study, we first gathered data from different cancers through the GEO and TCGA databases, and then integrated the data using bioinformatics methods. Through pathway analysis, protein–protein interaction (PPI) analysis, co-expression analysis, and other bioinformatics analysis methods, we identified the genes influencing the progress of pRCC, further screened the Hub genes, and performed differential verification and survival analysis using various databases. We found that the gene *P4HB* is significantly upregulated in pRCC and is closely related to survival. Finally, we analyzed the potential pathways of *P4HB* and related "gene panel" and conducted immunological correlation analysis and co-expression analysis. It was found that P4HB is associated with multiple immune cell infiltration and is co-expressed with important markers of immunological checkpoints, such as PD1, PDL2, CTLA-4, and TOX. Thus, P4HB is likely to play a pivotal role in the development of pRCC, serving as an oncogene through immunomodulation.

## Materials and methods

### Microarray data

Gene Expression Omnibus (GEO; www.ncbi.nlm.nih.gov/geo/) is a publicly available genomic database with high-throughput gene expression and microarray data [13]. The two datasets (GSE11151 and GSE15641) were selected from the GEO dataset for further analysis based on the Affymetrix Human Genome (GPL570) platform and the Affymetrix Human Genome (GPL96) platform, respectively. The GSE11151 dataset included 19 pRCC samples and 5 normal samples, while the GSE15641 dataset contained 11 pRCC samples and 23 normal samples. GSE11151 and GSE15641 were then combined with The Cancer Genome Atlas (TCGA) dataset to analyze overlapping DEGs, and logFC > 1 and *p* < 0.05 were set as the cut-off criteria [14–16].

### Gene ontology and KEGG pathway analysis

GO analysis was performed to analyze the potential functions of DEGs using the Metascape and Webgestalt website (http://metascape.org/gp/index.html#/main/step/) [17, 18]. DAVID is an online bioinformatics database for DEG functional analysis and KEGG pathway enrichment (DAVID, http://david.ncifcrf.gov/) [19]. The DAVID online tool visualized using R language and CluGO-plugin using Cytoscape software (http://www.cytoscape.org/) [20, 21] were combined to analyze the KEGG pathways. Statistical significance was set at *p* < 0.05.

### PPI network analysis and hub genes screening

The String online tool was used to study the interaction network among various DEGs (http://string-db.org/cgi/) [22]. First, the DEGs were entered into the database, and the confidence score was set to ≥ 0.7, the unlinked DEGs were deleted, and the remaining DEG protein interaction data and images were obtained. The data obtained from the string website were substituted into the Cytoscape software, and hub genes were obtained through the cytohubba plugin. The top 15 genes were obtained using MCC algorithms. GO analysis was performed using webgestalt and DAVID website, and then visualized using the R language. The CluGO plugin in Cytoscape software was used to analyze the potential pathways of Hub genes (http://www.cytoscape.org/).

### DEGs in pRCC

The TCGA database was used to screen for DEGs between pRCC and the normal kidney tissue through the GEPIA website, and logFC > 1 and *p*  < 0.05 were set as the cut-off criteria.

### Survival outcome of the hub genes in pRCC

The correlation between hub gene expression and survival in pRCC was analyzed using the GEPIA online tool (http://gepia.Cancer-pku.cn/) [15]. The cut-off criterion was set to 50%. The hazard ratio (HR) of 95% confidence interval and log-rank P-value were automatically calculated by the website and were displayed directly on the web page. Statistical significance was set at *p* < 0.05. The TIMER website (http://cistrome.shinyapps.io/timer/) [23] was used to identify the differential expression of P4HB in various tumors.

### Immunohistochemistry (IHC)

Samples of renal cell carcinoma and adjacent tissues of the First Affiliated Hospital of Dalian Medical University were selected for immunohistochemical analysis. All the relevant patients provided informed consent. The study was approved by the Ethics Committee of the First Affiliated Hospital of Dalian Medical University. Paraffin pathological sections were first incubated for 2 h and then subjected to antigen retrieval. Pathological sections were stained with rabbit anti-human P4HB antibody (1:100, ABclonal, A0692, China) at 4 °C and then stained with horseradish peroxidase (HRP)-conjugated secondary antibody for 1 h. Immunohistochemistry was performed using a DAB substrate kit. Hematoxylin staining and sealing were performed followed by image capture. The analysis was performed using Image Pro Plus software, semi-quantitative analysis was performed using the IOD/Area method, and statistical analyses were performed using the GraphPad Prism software (version 8.0). Data was expressed as mean ± SD and statistical significance was set at *p* < 0.05.

### TIMER

Since TIMER is a portal for systemic analysis of tumor infiltration (http://cistrome.shinyapps.io/timer/) [23], we used the TIMER website to analyze the relationship between P4HB and immune cell infiltration. Additionally, we performed immunological co-expression analysis to observe the connection between P4HB and PD-1, PD-L1, PD-L2, CTLA-4, and TOX.

### R language analysis of hub genes and P4HB

The RNA-seq transcriptome data of the pRCC cohort were downloaded from the TCGA (https://cancergenome.nih.gov/) data portal. The Limma package and pheatmap package were used to analyze the expression of hub genes and *P4HB* in 271 tumor patients and 32 normal renal tissues. The survival package was used for the survival analysis of the P4HB.

## Results

### Identification of DEGs in pRCC

**Fig. 1:**
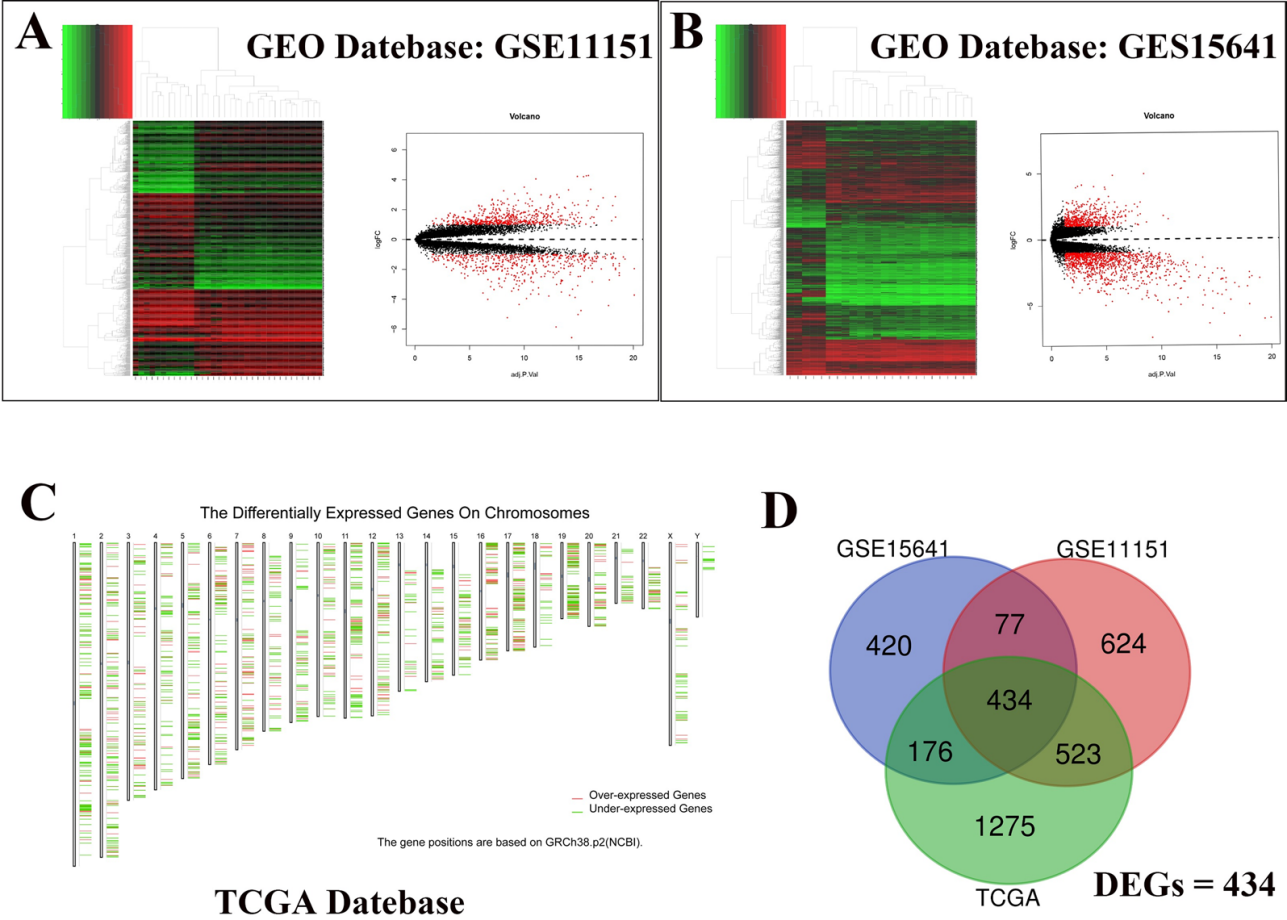
Identification of overlapping DEGs in the GEO database and the TCGA database. **a** Heatmap and volcano plots of DEGs in the GEO database using the R language (GES11151). **b** Heatmap and volcano plots of DEGs in the GEO database using the R language (GES15641). **c** Screening for differential genes in the TCGA database via the GEPIA website. **d** Venn plots of DEGs across the GEO database and the TCGA database

R studio was used to investigate the DEGs by mining the (GEO GSE11151 [16] and GSE15641 [14]) database (https://www.ncbi.nlm.nih.gov/geo/) in pRCC. The DEGs in the two data were analyzed and heat map and volcano map analysis was performed (Fig. 1a, b). The DEGs of pRCC in the TCGA database was carried out using the GEPIA website [15] (Fig. 1c). The data were filtered using the criteria logFC > 1, *P* < 0.05. The overlapping DEGs identified among the three datasets comprised 149 upregulated and 285 downregulated genes, which were presented using Venn analysis and are presented in tables (Fig. 1d, Table 1, Additional file 1: Tables S1–S3).

**Table 1:** A total of 434 DEGs were identified from TCGA and GEO datasets, including 149 upregulated and 285 downregulated genes in pRCC compared with normal tissues

DEGs	Gene name
Upregulated genes (n = 149)	*UGCG, TKT, HSPB1, TMSB10, HSPB8, ANXA1, MMP7, MET, ALOX5, RNASE6, GPNMB, ITGA3, FCGR2B, SEL1L3, DDB2, MGAT4B, EVI2A, HS3ST1, C3, FAS, TNFSF10, CRYAB, BCHE, C3AR1, PPAP2C, ELF3, HLA-J, LY86, PIGR, EZR, KDELC1, LPXN, SNX10, CXCL8, PERP, AHR, LACTB2, IFI27, TMEM140, CCDC109B, TNFAIP6, CYP3A5, SOX9, SNAP25, CTSS, PLS1, ABCC3, KRT8, CASP1, CORO1C, CAPG, ENO2, OLR1, SEZ6L2, NEFL, LIPA, GBP2, IL18, GALNT12, CDH6, ITGB8, ANG, ITGB2, PRUNE2, RCN1, PSMB8, APOL1, QPCT, SCEL, TES, PLOD3, TIMP1, HAVCR1, RAB31, THEMIS2, ALDOA, TNFRSF12A, C17orf62, FUT8, S100A11, TNFSF13, MVP, RGS1, CD14, ANXA2, CD37, TYROBP, CCL18, SERPINE2, PNMA2, GPR183, PYGL, ALOX5AP, TREM2, CLDN3, IGFBP6, LGALS3, WIPI1, CLU, ARL6IP5, CORO1A, EVI2B, EMP3, TPK1, TNFAIP8, NLGN1, HLA-G, ISG20, CXCL1, ARPC1B, HRH1, PLA2G7, KRT18, C1QB, RHBDF2, BAZ1A, TFPI, VCAN, GLRB, AKR1C1, IL32, NIT2, HLA-B, RNASET2, LAPTM5, CXCL6, SLC34A2, FDXR, PLA2G16, CLDN1, CAV2, IGSF6, P4HB, LCN2, FZD1, C1QA, GPX1, AHNAK2, B4GALT5, ANXA4, TFPI2, APBB1IP, PSMB10, BHLHE41, CNTN6, APOC1, TMEM176B, PSMB9, CTSC*
Downregulated genes (n = 285)	*PTGER3, ERBB4, RALYL, XPNPEP2, SPINK1, SLC4A1, CUBN, GSTA1, MPPED2, KLK6, HMGCS2, ITIH5, HOXD11, SH3BP4, HRG, MMRN2, CALB1, NR2F1, HSD11B2, TUBAL3, TIMP3, CNN1, GPC5, TEK, PLN, A1CF, DACH1, GHR, TMEM30B, BHMT, ANGPTL3, TFCP2L1, PAK6, SLC16A5, ALDH4A1, TSPAN8, MEIS2, C8orf4, SLC22A6, BDKRB2, IDH2, EFHD1, RAP1GAP, CRHBP, ASS1, AQP2, DAO, TBX2, TSPAN7, PTPRB, FGF1, APLNR, TFAP2B, ITGA8, CEL, NPHS1, COLEC11, KHK, ESRRG, PBLD, GRHL2, STON1, NDNF, EGFL7, SLC34A1, STAP1, ACSF2, MT1G, ASAP2, WT1, SLC22A8, IL13RA2, AOC3, GJA4, GC, ALDH6A1, STC1, SEMA3G, TCF21, MEIS1, EGF, ANK2, THY1, HSPA2, TYRP1, MAN1C1, TMPRSS4, PHGDH, MYH11, ITPR1, PCK1, SLC26A4, ADH1C, DPYS, IGFBP2, NPY1R, PAPPA2, TIE1, CLDN10, AGTR1, SCNN1B, PODXL, RAMP3, CLIC5, AFM, ACPP, GIMAP6, MME, RHCG, CYP4A11, GRB14, MT1X, ARAP3, PLS3, C7, MST1L, PDK4, CLCNKB, SLC7A8, PDE1A, IGFBP5, TMPRSS2, CTGF, RERGL, ANGPT1, PTGER4, EMX1, EFEMP1, ATP6V0A4, ASPN, LHX1, CDH3, CD34, L1CAM, NR3C2, TAGLN, EMCN, BLNK, MGAM, CHGB, KDR, PACRG, HPD, SYNDIG1, GRAMD1B, PDE2A, NRN1, SPAG5, GATA3, PLCG2, KCNJ1, NFASC, SLC12A3, CLDN5, PTGDS, TCEAL2, SLC5A12, TLN2, ACADSB, NTRK2, MFGE8, CA4, EPN3, FABP1, PLAT, CYP4F2, S1PR1, HOXD10, IL1R2, SH3BP5, NELL1, AQP3, FGF9, PLCL1, NPHS2, CALML3, SLC12A1, SLC7A9, AZGP1, SLC43A1, CXCL12, DDN, APOD, LDB2, EHD3, PVALB, FOXI1, TBX3, SPARCL1, RASL11B, FAM184A, PRSS23, NTF3, SORD, FBP1, PDZD2, SPTBN2, LHFP, SUSD4, DDC, GSTM3, HBB, KNG1, CASR, TFAP2A, SRPX, COX7A1, MECOM, CDKN1C, GAS1, PEG3, PCDH9, FECH, PLG, SOCS2, ARG2, EIF1AY, HAO2, RNF186, MT1F, ERVMER34-1, PPP1R16B, DCXR, TGFBR3, UMOD, ADH1B, LPL, PPP2R2B, DIO1, ELF5, OLFML3, SIM2, CSRP2, RHOBTB3, HRC, GPC3, FCN3, PDGFRA, FRZB, CLDN8, FAM107A, NES, FXYD1, SLC5A2, REN, G6PC, ADH6, MT1H, GPRC5B, RAMP2, KIAA1462, TGFB1I1, KDM5D, ALB, CEACAM1, ALDOB, DCN, PLVAP, EPAS1, CKMT2, SLC13A3, CPN2, AGMAT, ASB9, PTH1R, UGT8, SNAI2, CYFIP2, GAD1, KLHL3, ATP6V1B1, DUSP9, SERPINA5, TM4SF5, SFRP1, OLFML1, CDH5, SELENBP1, PIPOX, PRODH2, FOLR3, PARM1, FBLN5, EFS, WLS*

### GO and KEGG pathway analysis

**Fig. 2:**
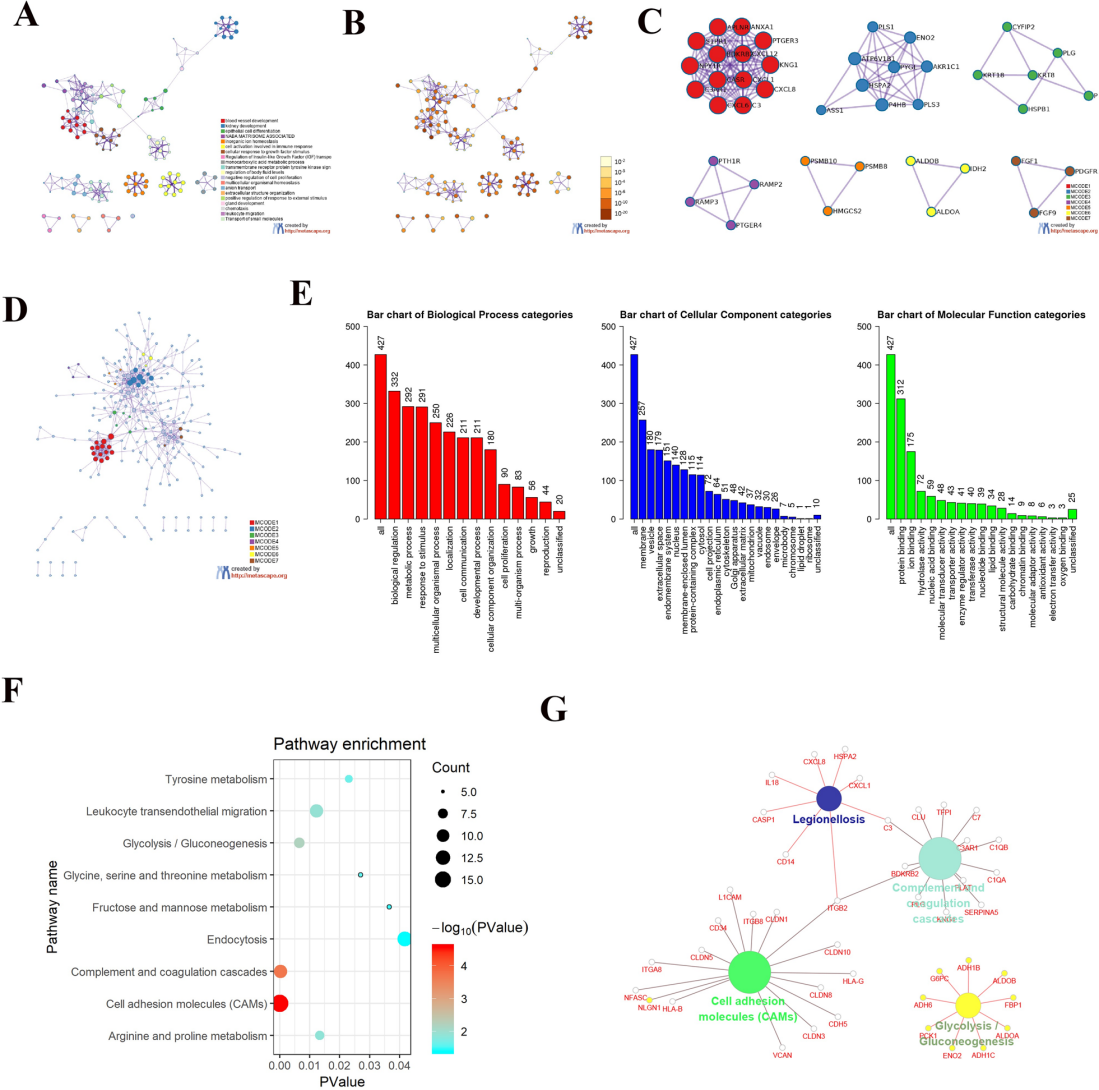
GO and KEGG analysis of DEGs. **a**, **b** GO analysis of DEGs using the metascape online website. **c**, **d** PPI analysis of DEGs using the metascape online website. **e** GO (BP. MF.CC) pathway analysis of DEGs using Webgestalt online website. **f** KEGG pathway analysis via DAVID online tool and bubble chart display via R language. **g** KEGG pathway analysis of DEGs via clugo plugin in Cytoscape software

To further study the feature of the DEGs, we performed GO analysis on DEGs through the metascape (http://metascape.org/gp/index.html#/main/step1) [18] and webgestalt (http://www.webgestalt.org/) [17] online tools (Fig. 2a–d). We found that the DEGs were primarily enriched in cellular components of the membrane, vesicle, extracellular space, endomembrane system, and nucleus. Regarding the biological process (BP), the DEGs were enriched in biological regulation, metabolic processes, responses to stimuli, multicellular organismal processes, and localization. The changes in molecular function (MF) were remarkably strengthened in protein binding, ion binding, hydrolase activity, nucleic acid binding, and molecular transducer activity (Fig. 2e). We further analyzed the DEG-enriched KEGG pathway using the DAVID online tool (DAVID, https://david.ncifcrf.gov/) [19] and visualized it using the R language. The KEGG pathway was analyzed using the CLUGO plugin in Cytoscape software (http://www.cytoscape.org/) [20, 21], and the two methods were integrated. We identified that DEGs were mainly associated with three pathways: cell adhesion molecules (CAMs), glycolysis, and complement and coagulation cascades (Fig. 2f, g).

### PPI, screening of hub genes, and hub genes related pathway analysis

**Fig. 3:**
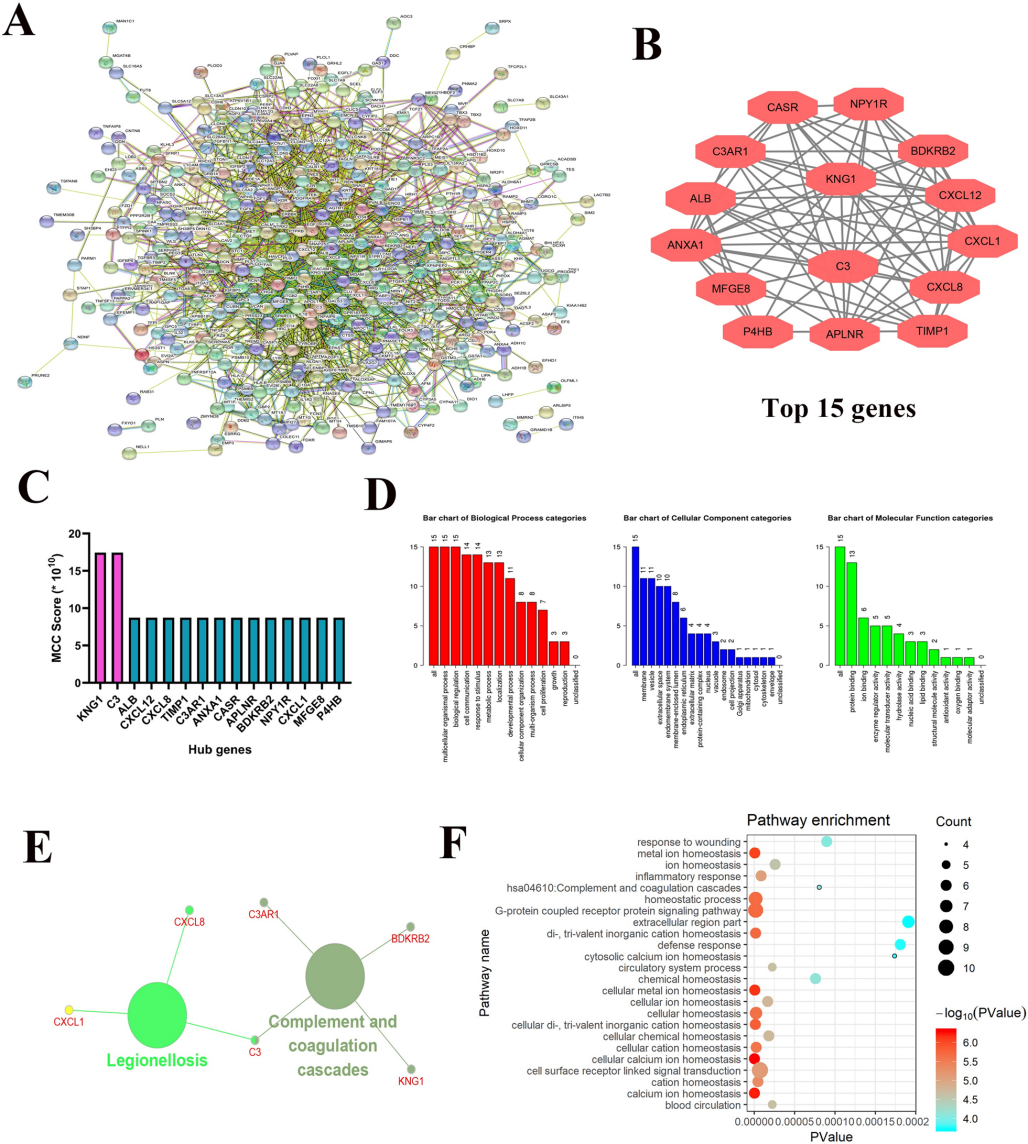
PPI analysis of DEGs, screening of Hub gene, and pathway analysis of Hub gene. **a** PPI analysis of DEGs through the STRING website tool. **b**, **c** Screening the top 15 Hub genes by MCC operation through the cytohubba plugin in Cytoscape software. **d** GO (BP. MF.CC) analysis of hub genes using the webgestalt online website. **e** KEGG pathway analysis of DEGs via clugo plugin in Cytoscape software. **f** GO pathway analysis via DAVID online tool and bubble chart display via R language

To better understand the relationship between DEGs, we used the String online tool (https://string-db.org/cgi/) [24] to investigate the connection between various DEGs(Fig. 3a). Then the top 15 genes: *KNG1, C3, ALB, CXCL12, CXCL8, TIMP1, C3AR1, ANXA1, CASR, APLNR, BDKRB2, NPY1R, CXCL1, MFGE8*, and *P4HB* were confirmed as potential hub genes according to the MCC method generated by cytohubba plugin in Cytoscape software (Fig. 3b, c) [25]. We then applied GO analysis through webgestalt and DAVID websites and visualized the data using the R language. Through integration analysis of the results obtained from the two websites, the Hub gene was found to be principally enriched in the GO pathways, such as metal ion homeostasis, homeostatic process, cellular homeostasis, cellular ion homeostasis, and extracellular region (Fig. 3d). On analyzing the KEGG pathway, we discovered that Hub genes were substantial in the complement and coagulation cascades, and legionellosis pathways (Fig. 3e, f).

### Differential expression analysis of Hub genes in pRCC and normal kidney tissues

**Fig. 4:**
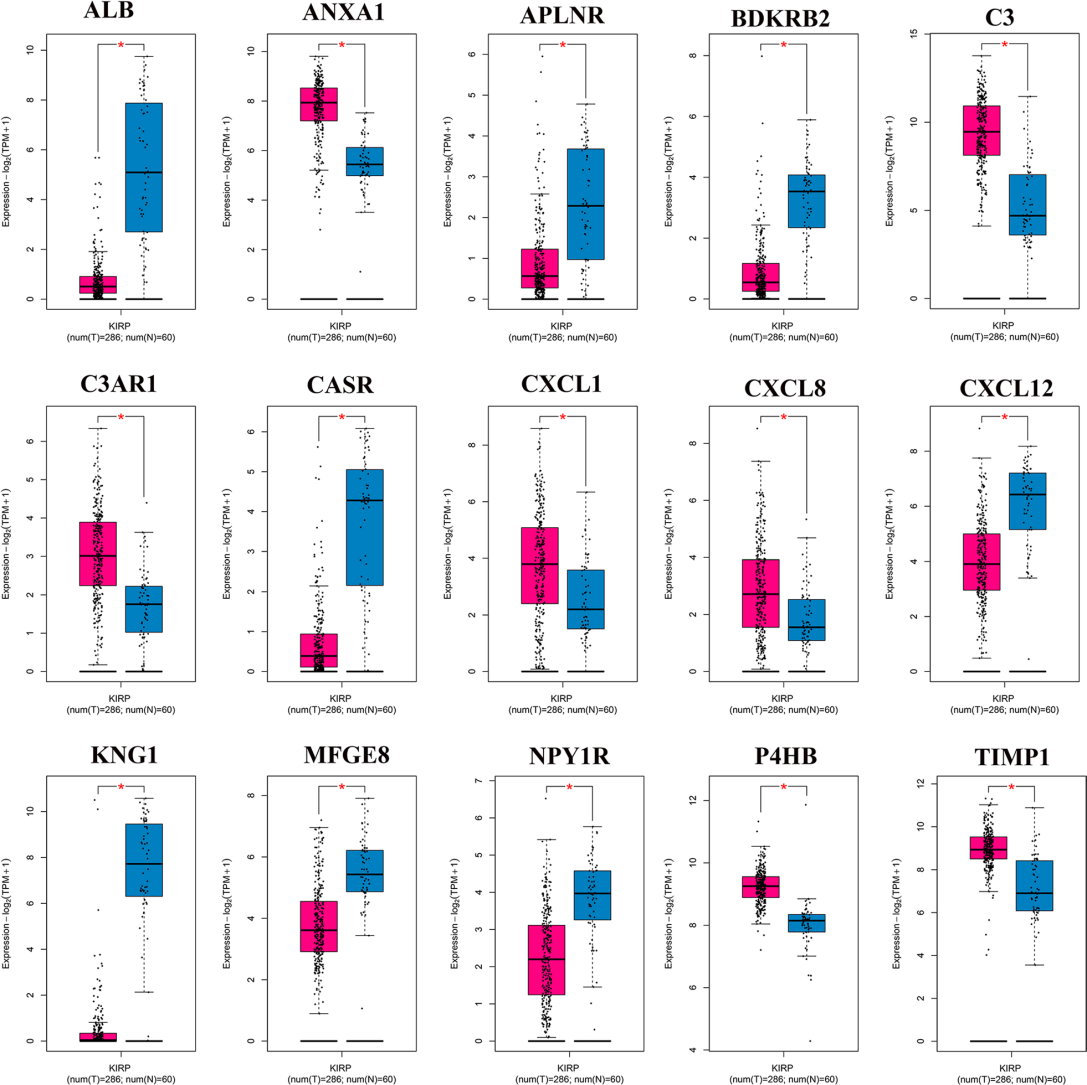
The difference in mRNA expression of Hub gene in PRCC and normal kidney tissues by TCGA database (GEPIA online tool)

**Fig. 5:**
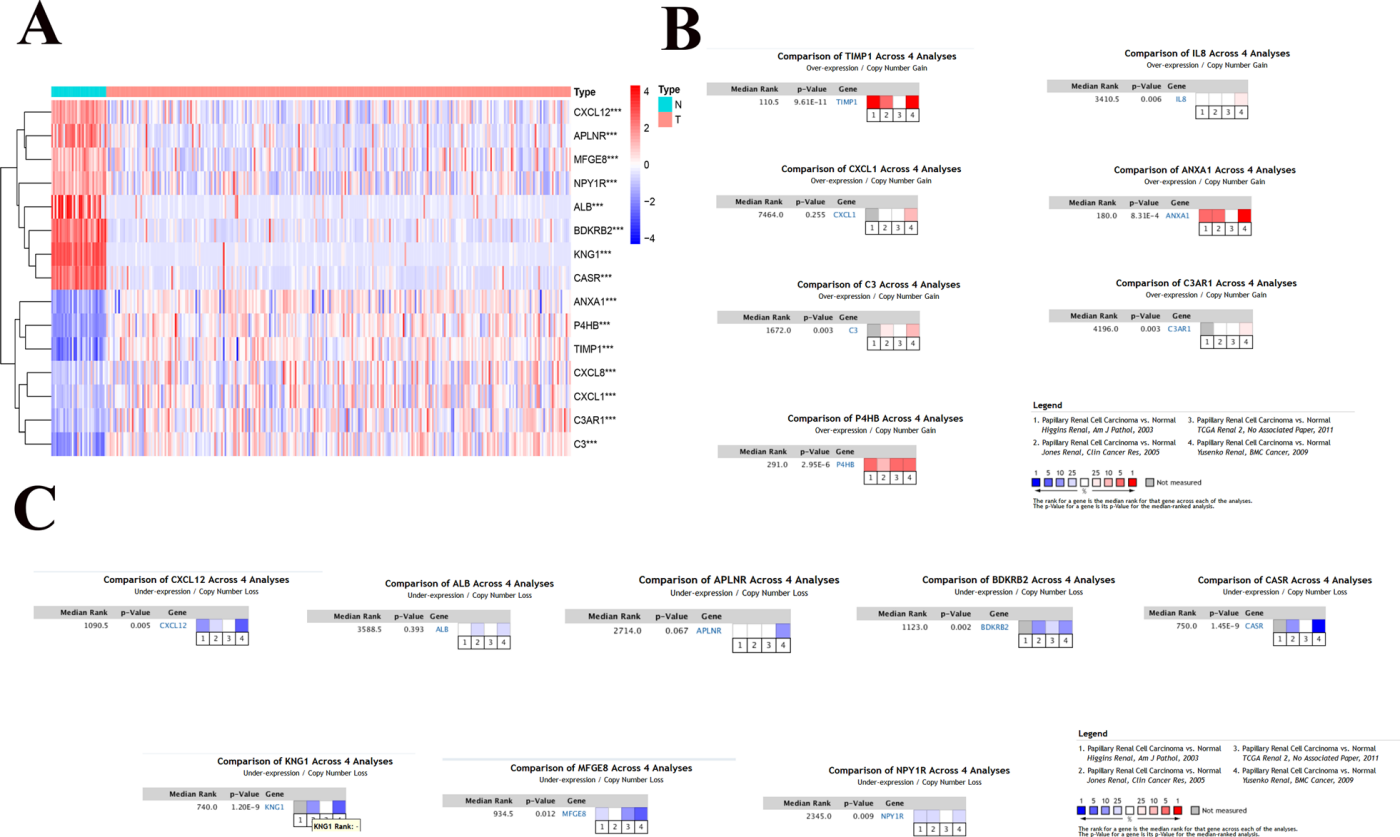
Survival analysis of Hub genes in PRCC. **a** The differential expression of the hub genes between pRCC and normal kidney tissue. **b**, **c** Meta-analysis of four different databases in the TCGA database and revalidation of seven upregulated genes and eight downregulated genes

The differential expression of hub genes between pRCC and normal kidney tissue was verified using the TCGA database. We analyzed each single hub gene using the GEPIA website and concluded that *C3, CXCL8, TIMP1, C3AR1, ANXA1, CXCL1*, and *P4HB* were significantly upregulated in pRCC, compared with normal renal tissues, while *CXCL12, ALB, APLNR, BDKRB2, CASR, KNG1, MFGE8*, and *NPY1R* were significantly downregulated with statistically significant differences (Fig. 4). This is because the data in GEPIA are automatically generated by the website. To further confirm the credibility of the above analysis results, we included papillary RCC samples from the TGCA database for re-analysis, including 32 normal kidney tissue samples and 271 tumor samples. We downloaded the latest data from TCGA GDC data portal, excluded the samples of non-papillary RCC, and screened the samples of papillary RCC according to a paper published article in Cell Report, which can be seen in the supplementary material (Additional file 1: Table S4) [26]. By analyzing the new data, we found that the results obtained by the re-analysis were consistent with those obtained in the previous analysis (Fig. 5a). To verify the reliability of our results, we performed a meta-analysis using the ONCOMINE database. We found that the results were consistent with previous reports, but the differences between *CXCL1* and *APLNR* genes were not statistically significant between pRCC and normal kidney tissues (Fig. 5b, c). This inconsistency may be related to the different DEGs between different databases.

### To determine P4HB as the target gene by survival analysis

**Fig. 6:**
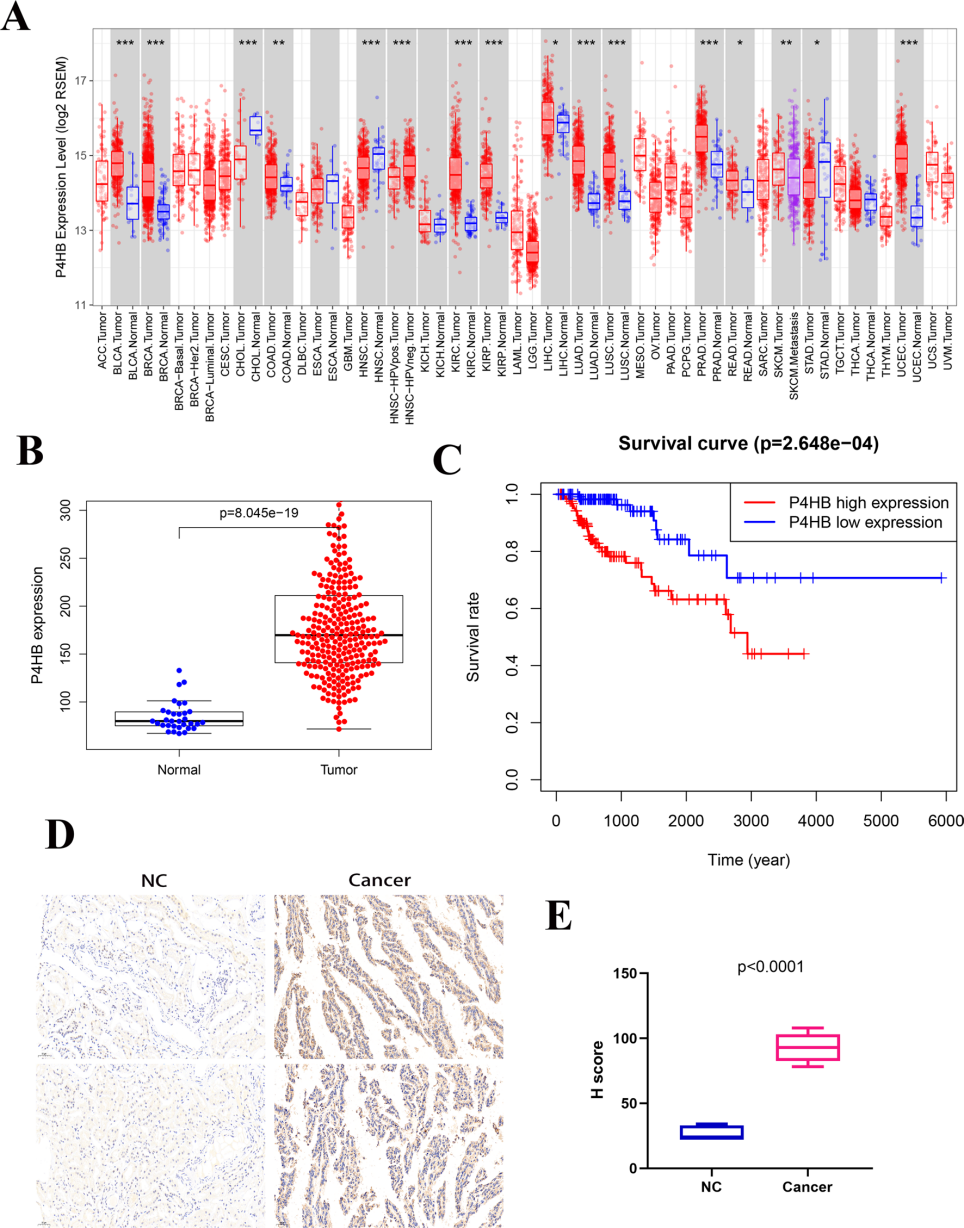
Analysis of P4HB information in PRCC through the TCGA database. **a** Analysis of the differential expression of P4HB between PRCC and normal kidney tissue in multiple tumors via TIMER online tool. **b** Analysis of P4HB expression differences in PRCC and normal kidney tissues. **c** Analysis relationship of different P4HB expressions and overall survival time. **d** Immunohistochemical images of P4HB in kidney cancer and normal tissues. **e** H score was performed to assess protein levels of gene P4HB in five normal tissues and five papillary renal cell carcinoma samples. *P* values < 0.0001 were considered statistically significant. All results are expressed as mean ± standard deviation (SD)

To investigate the link between the Hub gene and pRCC survival, we applied survival analysis to the Hub gene using the GEPIA online tool (http://gepia.cancer-pku.cn/) for differential analysis [15]. We detected that the survival with *P4HB, MFGE8*, and *BDKRB2* had statistically significant features in pRCC, and genes with high expression status indicated low OS (Additional file 2: Figure S1). However, *MFGE8* and *BDKRB2* expression was downregulated and *P4HB* was upregulated in pRCC compared with normal renal tissues. Therefore, we chose *P4H*B as our target gene. To understand whether the *P4HB* gene also acts as an oncogene in other types of tumors, we analyzed the differential expression of P4HB in different tumors and normal healthy tissues through the TIMER website (https://cistrome.shinyapps.io/timer/) [23]. We would like to conclude that *P4HB* is upregulated in a set of tumors, such as BLCA, BRCA, COAD, KIRC, LIHC, LUAD, LUSC, PPAD, READ, and UCEC, but downregulated in CHOL and STAD (Fig. 6a). To further verify the expression of *P4HB* in pRCC and its relationship with prognosis, we downloaded the latest data from the TCGA GDC data portal, excluded the samples of non-papillary RCC, and screened the samples of papillary RCC, and the results showed that the expression of *P4H*B was significantly upregulated in pRCC compared with that in normal kidney tissues (Fig. 6b). Through further survival analysis, we found that the high *P4HB* expression group had a poorer prognosis than the low *P4HB* expression group. These results indicate that P4HB may play a role as an oncogene in pRCC. To further evaluate the expression of P4HB, IHC analysis was performed to assess the protein level of P4HB in five pRCC and normal tissue samples. We found that the expression of the P4HB gene in pRCC was significantly higher than that in normal kidney tissues (Fig. 6d, e).

### P4HB acts as an immune-related gene in pRCC

We identified genes with a PPI network with P4HB to explore the molecular mechanisms of P4HB through the STRING website (Additional file 3: Figure S2A). When scanning these related genes through the KEGG pathway analysis, we identified the P4HB-related genes that were primarily enriched in antigen processing and presentation, protein processing in the endoplasmic reticulum, and thyroid hormone pathway (Additional file 3: Figure S2B). Because immunotherapy has always been a hot topic in kidney cancer research, and P4HB is related to antigen processing and presentation, we concluded that P4HB is likely to promote the development and progression of pRCC through immunomodulation.

Therefore, we performed co-expression analysis of P4HB, antigen processing, and presentation-related genes in the KEGG pathway. We found that *P4HB* has a strong co-expression relationship with the immune-related genes *PDIA3, CALR, CANX*, and *HSP90B1*. (Additional file 3: Figure S2C, S2D, S2E, S2F).

**Fig. 7:**
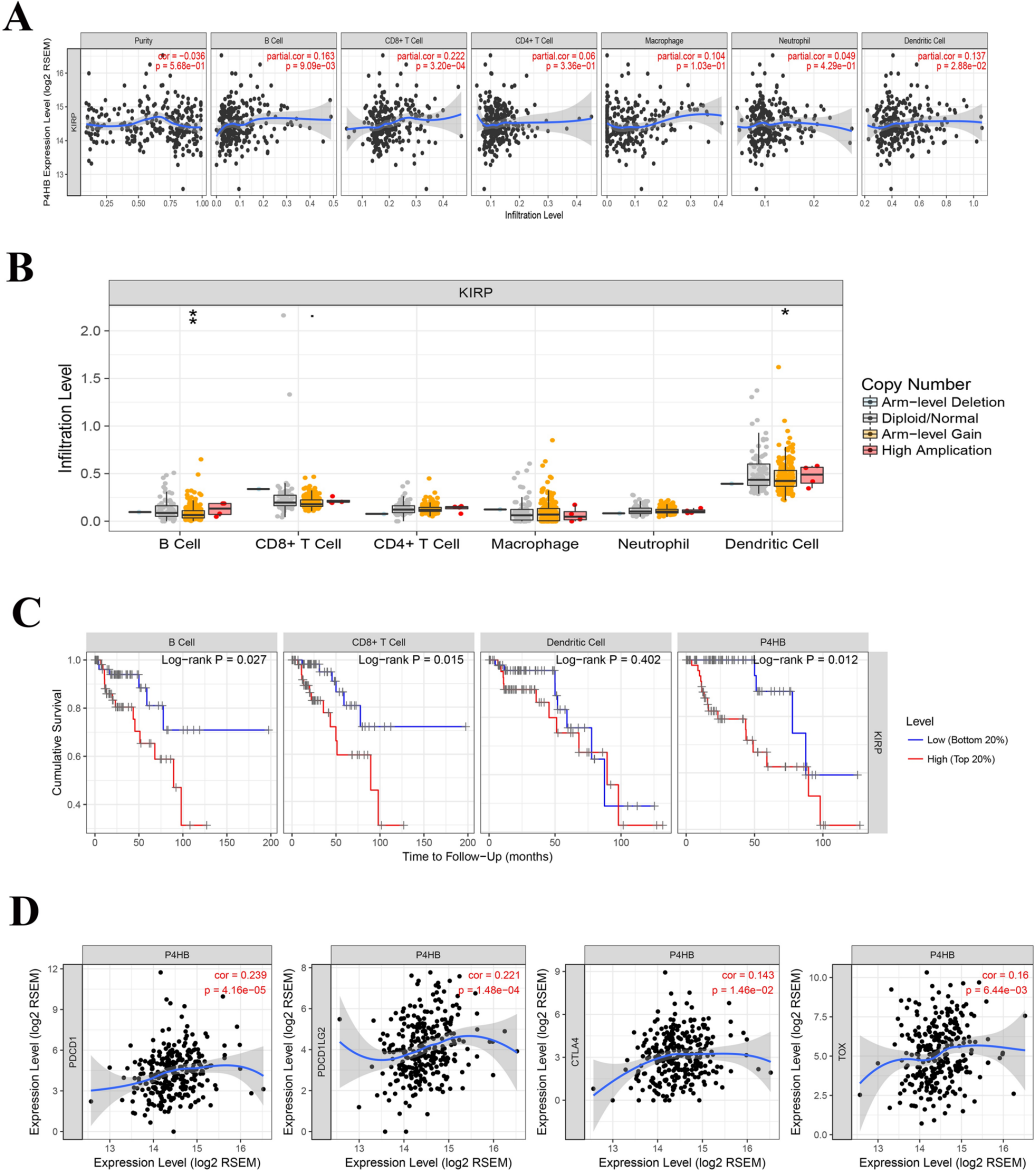
Immunological correlation analysis of P4HB. **a**, **b** P4HB expression and immune cell infiltration analysis. **c** Survival analysis of P4HB and immune cells in PRCC. **d** Co-expression analysis of P4HB and immunological checkpoint related genes

To further prove the validity of the study, we analyzed the relationship between P4HB and immune cell infiltration using the TIMER website. As a result, in pRCC, P4HB is involved in the infiltration of B cells, CD8^+^ cells, and dendritic cells (Fig. 7a, b). Through the survival analysis of immune cells and P4HB, we found that high expression of B cells, CD8^+^ T cells, and P4HB was associated with poor prognosis (Fig. 7c). Considering that in the current scenario immunotherapy is mainly focused on, immunological checkpoint inhibitors, such as PD1, PDL1, and CTAL4, we further analyzed the co-expression relationship between P4HB and immune checkpoint-related genes PD1, PDL1, PDL2, and CTAL4. Surprisingly, P4HB had a significant co-expression relationship with PD1, PDL2, and CTAL4, but no clear relationship with PDL1 (data not shown). *TOX* is a newly discovered gene, and three consecutive articles published in *Nature* refer to the role of the *TOX* gene in tumor immunotherapy [27–29]. Nevertheless, our study found that P4HB and TOX have strong co-expression relationships, which provides a reliable basis for future research (Fig. 7d). However, the above results require further experimental confirmation.

## Discussion

RCC is the most common type of cancer in the kidney, including ccRCC(70%) and nccRCC(30%) [6]. Currently, the most widely used RCC overall survival prediction system in clinical practice is the International Metastatic Renal Cell Database (IMDC) standard [30]. As the most common type of cancer in nccRCC, pRCC accounts for 15% of all kidney cancers [31]. Despite the recent introduction in the clinical practice of several targeted drugs for patients with advanced RCC, the evidence regarding their efficiency in pRCC is less robust. The case of pRCC is limited, and most of the genetic testing and RCTs were excluded or presented as a small subgroup [6]. Moreover, as nccRCC, pRCC is different from ccRCC, so the relevant research results of ccRCC are not applicable to pRCC. Studies have shown that due to the heterogeneity between inter- and intra-tumors, the genomic assessment between ccRCC and different nccRCC subtypes is very different [32]. Therefore, it is essential to explore the underlying molecular mechanisms and identify novel biomarkers for precise treatment.

Fortunately, as bioinformatics technologies develop rapidly, we can better understand the molecular basis of cancer. Identification of the potential genes and their interaction networks leads to the identification of useful prognostic and predictive biomarkers and provide new ideas for the treatment of pRCC. Our research identified 434 DEGs (149 upregulated and 285 downregulated) based on the TCGA and GEO datasets. Some bioinformatics methods were subsequently utilized to identify DEGs, including GO and KEGG pathway analysis, PPI network construction, survival analysis, and co-expression analysis.

Gene enrichment assays are universally performed through GO analysis. In our study, these DEGs were abundant in biological regulation and metabolic processes. Many studies have shown that carcinogenesis may be closely related to metabolism [33, 34]. RCC is considered to be cancer derived from metabolic changes owing to the high frequency of mutations in genes that control aspects of metabolism [35], such as mutations in VHL and MET. TCGA projects reported that pRCC has the highest MET mutation rate among all types of RCC [36]. These findings support our study.

Next, the interrelation analysis of pathways was carried out using KEGG processes in ClUGO. KEGG proved that the pathways associated with the DEGs were significantly correlated with tumor growth and metastasis [37–39]. According to relevant studies, CAMs are essential for transducing intracellular signals responsible for the adhesion, migration, invasion, and progression of tumors [39]. Unlike normal benign differentiated cells, most cancer cells survive aerobic glycolysis to meet energy and membrane structure requirements, termed as the "Warburg effect." Increased glycolysis is a hallmark of malignancy, indicating tumor invasion and poor prognosis [38].

To further systemically analyze the relationship and functions of important DEGs in pRCC, we obtained 15 DEGs with the highest degree. Interestingly, some of these hub genes are immune-related genes that play a vital role in the progression of tumors, including pRCC, such as *C3, NPY1R*, and *BDKRB2.* In the tumor microenvironment, complement activation accelerates tumor growth and promotes tumor metastasis [40]. Activation of C3 is central to the complement pathways, which collectively results in the elimination of the antigen target [41]. A high level of C3 has been observed in many cancer patients` serum [42, 43]. Recent studies have suggested that C3 aids tumor growth through an immunosuppressive mechanism [44, 45]. Pang et al. [46] reported that C3 plays a crucial role in the regulation network of DEGs in pRCC, which is consistent with our results. Since NPY1R is expressed in various types of tumors, the role of NPY1R, as a tumor-facilitated gene, has been postulated [47–49]. Lv et al. [50] reported that NPY1R suppresses human hepatocellular carcinoma (HCC) cell growth by inactivating the mitogen-activated protein kinase signaling pathway. Low expression of NPY1R helps to indicate highly aggressive clinical features and poor prognosis in HCC patients. The growth of solid tumors requires blood supply, meeting metabolic demands, and hematogenous dissemination. Studies have shown that BDKRB2 is one of the downregulated genes involved in angiogenesis in several tumors [51, 52]. These reports are in line with our biosignal analysis results, and further investigation of these genes for clinical research is required.

To better understand the role of P4HB in immunomodulation, we performed a co-expression analysis of P4HB and immunology-related KEGG pathways. It is noteworthy that *P4HB* strongly co-expresses with immune-related genes, *PDIA3, CALR, CANX*, and *HSP90B1*. KEGG results showed that the P4HB related genes were mainly enriched in antigen processing and presentation, and protein processing in the endoplasmic reticulum. Interestingly, these two pathways have intimated connections, as the report showed that the ER stress response plays an important role in anti-tumor immunity [53, 54]. The ER stress response promotes tumor immune evasion by impairing tumor antigen presentation. In addition, the ER stress response enhances anti-tumor immunity by interfering with the processing and presentation of the tumor antigens [55]. According to previous studies, both PDIA3 and CANX reside in the endoplasmic reticulum [56, 57]. It is reported that PDIA3 is involved in multiple biological functions, including antigen processing and presentation [58]. The high expression status of PDIA3 accounts for the poor prognosis of several tumors [59–62]. CALR, an antigen processing and presentation molecule, supports the induction of DC maturation [63, 64]. HSP90B1 is an endoplasmic reticulum stress-related protein. Converging studies have shown that HSP90B1 is highly expressed in some malignancies, including RCC [65–68].

Recent studies have shown that P4HB is upregulated in many cancer cell types, in which high expression is closely associated with advanced tumor stage and poor prognosis [61, 69–71]. Zou [62] concluded that the upregulated expression of P4HB is significantly correlated with poor prognosis of diffuse gliomas. As reported, P4HB may also serve as a promising chemotherapeutic target for ovarian and gastric cancer cells [72, 73]. Targeting P4HB could attenuate temozolomide resistance in malignant glioma by inhibiting ER stress response pathways [74]. The ER stress response pathway plays a dual role in the modulation of tumor immunity [53, 54]. As for kidney cancer, overexpression of P4HB is an unfavorable prognostic factor in patients with clear cell renal cell carcinoma [75]. The development and progression of pRCC is the direction of our further study, irrespective of its connection to ER stress and APC.

To further identify the relationship between P4HB and tumor immunity, we conducted an immunological correlation analysis. We found that P4HB is related to a high infiltration rate in immune cells, including B cells, CD8^+^ T cells, and dendritic cells. It is well documented that immune effector cells such as T and B cells, DCs, MDSCs, and macrophages are important components of the tumor microenvironment. Vincenzo Di Nunno et al. demonstrated that in the overall RCC population, a higher neutrophil-to-lymphocyte ratio resulted in worse overall survival [76]. In addition, high expression of B cells, CD8^+^ T cells, and P4HB is related to poor prognosis in patients with pRCC. A study showed that P4HB could be internalized by T cells, which play a vital role in T cell activation and proliferation, adhesion, and migration [77]. P4HB could cause the proliferation of mutant Ba/F3 murine pre-B cells in a screen of ST2 murine bone marrow stromal cells [78]. Hurst [79] found that, as a new inhibitor, E64FC26 improved viability and limited the unfolded protein response, decreased global P4HB expression in normal healthy T cells, and reshaped T cell metabolism, which helps to enhance anti-tumor immune responses. In addition, P4HB inhibited the endoplasmic reticulum stress response pathways and ER stress had a strong connection with immune responses, which indicated that P4HB expression was significantly related to immune responses. We know that the development of cancer and its response to treatment is influenced by both innate and adaptive immunity, with which immune-related cells play a vital regulatory role in the occurrence and progression of tumors. Hence, we speculated that P4HB interacts with immune cells.

Blockade of immune checkpoints seems to be the most promising approach for activating therapeutic anti-tumor immunity [80]. Our target genes were strongly correlated with PD-1, CTLA-4, and PD-L2. PD-1 and CTLA-4 are critical immune checkpoint receptors, and high expression of PD-1 and CTLA-4 is characterized by poor prognosis in RCC patients [81]. Ciccarese et al. showed that several anti-PD-1/PD-L1-targeted therapies are expected to be effective in RCC patients [82]. It has been reported that PDL-2 has the highest frequency rate among all types of RCC [83], which is consistent with our results. Recent studies have shown that PD-L2 expression in tumor cells is closely related to poor prognosis in esophageal cancer [83, 84]. In addition, other RCC-related immune checkpoints are gradually being discovered, such as vascular endothelial growth factor receptor (VEGFR), mammalian target of rapamycin (mTOR), glucocorticoid-induced TNFR-related protein (GITR), and lymphocyte-activation gene 3 (LAG-3), among others, and related targeted drugs are currently under evaluation [81, 85, 86]. Our results showed that P4HB is significantly upregulated in pRCC and has significant co-expression with these immune checkpoint genes, and overexpression of P4HB is linked to a higher grade and worse outcome in pRCC patients. Consistent with TCGA findings, our IHC result also confirmed that compared to adjacent tissues of pRCC patients, P4HB is significantly upregulated in pRCC tissues, which has not been reported by others. These findings provide new ideas for further studies.

The success of anti-PD-1 and CTLA-4 in cancer immunotherapy has stimulated the search for other cancer therapeutic targets. Exhausted CD8^+^ T (Tex) cells are also a significant checkpoint blockade target for immunotherapies [87]. *TOX* is a newly discovered hot gene. It serves as a primary regulator of Tex cells. TOX plays a significant role in inducing the canonical features of T cell exhaustion and initiating a tex-cell-specific epigenetic program [88]. Fortunately, our key gene was significantly co-expressed with *TOX*. The target gene we dug might be of high value and is probably crucial for determining immunotherapy efficacy. Of course, this study also has limitations. We processed integrated bioinformatics analysis of multiple datasets to see pRCC related hub genes, and mainly focused on how to apply databases. The lack of corresponding clinical experimental verification, which will be improved in future research.

## Conclusion

Thus, we performed integrated bioinformatics analysis of multiple datasets to identify pRCC-related hub genes. Finally, *P4HB* has been identified as a potential immune-related prognostic biomarker. High expression of P4HB was associated with OS and DFS in patients with pRCC. Interestingly, P4HB has a significant co-expression relationship with *PD-1, PD-L2*, and *CTLA-4*. It also has a strong correlation with *TOX*. These findings provide new clues for future studies.

## Supplementary Information

**Additional file 1: Table S1.** DEGs of KIRP in GSE11151 downloaded from the GEO dataset, including 19 pRCC samples and five normal samples. **Table S2.** DEGs of KIRP in GSE15641 downloaded from the GEOdataset, containing 11 pRCC samples and 23 normal samples. **Table S3.** DEGs between pRCC and normal kidney tissue inTCGA database screened through the GEPIA website. **Table S4.** 32normal kidney tissues samples and 271tumor samples downloaded from TCGA GDC data portal.

**Additional file 2: Figure S1. **Survival analysis of Hub genes in PRCC. (A) Analysis of Hub genes' OS and DFS in PRCC through TCGA database through DEPIA online website. (B, C) Analysis of the relevance of Hub genes and OS in PRCC through the DEPIA online website.

**Additional file 3: Figure S2. **Pathway analysis and co-expression analysis of P4HB related genes. (A) Analysis of genes having a PPI with P4HB via STRING website, and visualization of the PPI genes by Cytoscape software. (B) P4HB related genes for KEGG analysis using CLUE GO plugin in Cytoscape. (C–F) Co-expression analysis of genes involved in antigen processing and presentation in the KEGG pathway via cbioportal online tool.

## Data Availability

In the study, different web-based datasets were used for data analysis. The web links to all the original data sources were listed as below: The two datasets (GSE11151 and GSE15641) were picked up from the GEO dataset (http://www.ncbi.nlm.nih.gov/geo/). The RNA-seq transcriptome data of pRCC cohort were obtained from The Cancer Genome Atlas Program (TCGA) (https://cancergenome.nih.gov/) data portal. All data generated from the analysis process of this study are available from the corresponding author on reasonable request.

## Abbreviations

BP: Biological process
ccRCC: Clear cell renal cell carcinoma
CAMs: Cell adhesion molecules
CTLA-4: Cytotoxic T lymphocyte associated antigen-4
DEGs: Differentially expressed genes
HCC: Human hepatocellular carcinoma
GO: Gene ontology
GEO: Gene expression omnibus
KEGG: Kyoto encyclopedia of genes and genomes
MF: Molecular function
mRNAs: Messenger RNAs
PPI: Protein–protein interact
PD-1: Programmed cell death protein 1

## References

[1] Bray F, Ferlay J, Soerjomataram I (2018). Global cancer statistics 2018: GLOBOCAN estimates of incidence and mortality worldwide for 36 cancers in 185 countries. CA Cancer J Clin.

[2] Znaor A, Lortet-Tieulent J, Laversanne M (2015). International variations and trends in renal cell carcinoma incidence and mortality. Eur Urol.

[3] Moch H, Cubilla AL, Humphrey PA (2016). The 2016 WHO classification of tumours of the urinary system and male genital organs-part a: renal, penile, and testicular tumours. Eur Urol.

[4] Luo Q, Cui M, Deng Q (2019). Comprehensive analysis of differentially expressed profiles and reconstruction of a competing endogenous RNA network in papillary renal cell carcinoma. Mol Med Rep.

[5] Durinck S, Stawiski EW, Pavía-Jiménez A (2015). Spectrum of diverse genomic alterations define non-clear cell renal carcinoma subtypes. Nat Genetics.

[6] Courthod G, Tucci M, Di Maio M (2015). Papillary renal cell carcinoma: a review of the current therapeutic landscape. Crit Rev Oncol Hematol.

[7] Goodall GJ, Wickramasinghe VO (2021). RNA in cancer. Nat Rev Cancer.

[8] Laduca H, Stuenkel AJ, Dolinsky JS (2014). Utilization of multigene panels in hereditary cancer predisposition testing: analysis of more than 2000 patients. Genet Med.

[9] Ye M, He Z, Dai W (2018). TOP2AA -derived cancer panel drives cancer progression in papillary renal cell carcinoma. Oncol Lett.

[10] Mckay RR, Bossé D, Xie W (2018). The clinical activity of PD-1/PD-L1 inhibitors in metastatic non-clear cell renal cell carcinoma. Cancer Immunol Res.

[11] Chen DS, Mellman I (2017). Elements of cancer immunity and the cancer-immune set point. Nature.

[12] Wang Z, Song Q, Yang Z (2019). Construction of immune-related risk signature for renal papillary cell carcinoma. Cancer Med.

[13] Edgar R, Domrachev M, Lash AE (2002). Gene Expression Omnibus: NCBI gene expression and hybridization array data repository. Nucleic Acids Res.

[14] Jones J, Otu H, Spentzos D (2005). Gene signatures of progression and metastasis in renal cell cancer. Clin Cancer Res.

[15] Tang Z, Li C, Kang B (2017). GEPIA: a web server for cancer and normal gene expression profiling and interactive analyses. Nucleic Acids Res.

[16] Yusenko MV, Kuiper RP, Boethe T (2009). High-resolution DNA copy number and gene expression analyses distinguish chromophobe renal cell carcinomas and renal oncocytomas. BMC Cancer.

[17] Liao Y, Wang J, Jaehnig EJ (2019). WebGestalt 2019: gene set analysis toolkit with revamped UIs and APIs. Nucleic Acids Res.

[18] Zhou Y, Zhou B, Pache L (2019). Metascape provides a biologist-oriented resource for the analysis of systems-level datasets. Nat Commun.

[19] Da Huang W, Sherman BT, Lempicki RA (2009). Bioinformatics enrichment tools: paths toward the comprehensive functional analysis of large gene lists. Nucleic Acids Res.

[20] Bindea G, Mlecnik B, Hackl H (2009). ClueGO: a cytoscape plug-in to decipher functionally grouped gene ontology and pathway annotation networks. Bioinformatics.

[21] Smoot ME, Ono K, Ruscheinski J (2011). Cytoscape 2.8: new features for data integration and network visualization. Bioinformatics.

[22] Yang H, Wu J, Zhang J (2019). Integrated bioinformatics analysis of key genes involved in progress of colon cancer. Mol Genet Genomic Med.

[23] Li T, Fan J, Wang B (2017). TIMER: a web server for comprehensive analysis of tumor-infiltrating immune cells. Cancer Res.

[24] Szklarczyk D, Gable AL, Lyon D (2019). STRING v11: protein-protein association networks with increased coverage, supporting functional discovery in genome-wide experimental datasets. Nucleic Acids Res.

[25] Chin CH, Chen SH, Wu HH (2014). cytoHubba: identifying hub objects and sub-networks from complex interactome. BMC Syst Biol.

[26] Ricketts CJ, De Cubas AA, Fan H (2018). The cancer genome atlas comprehensive molecular characterization of renal cell carcinoma. Cell Rep.

[27] Scott AC, Dündar F, Zumbo P (2019). TOX is a critical regulator of tumour-specific T cell differentiation. Nature.

[28] Khan O, Giles JR, Mcdonald S (2019). TOX transcriptionally and epigenetically programs CD8(+) T cell exhaustion. Nature.

[29] Alfei F, Kanev K, Hofmann M (2019). TOX reinforces the phenotype and longevity of exhausted T cells in chronic viral infection. Nature.

[30] Massari F, Di Nunno V, Guida A (2021). Addition of primary metastatic site on bone, brain, and liver to IMDC criteria in patients with metastatic renal cell carcinoma: a validation study. Clin Genitourin Cancer.

[31] Akhtar M, Al-Bozom IA, Al Hussain HT (2019). Papillary renal cell carcinoma (PRCC): an update. Adv Anat Pathol.

[32] Massari F, Di Nunno V, Santoni M (2019). Toward a genome-based treatment landscape for renal cell carcinoma. Crit Rev Oncol Hematol.

[33] Robertson-Tessi M, Gillies RJ, Gatenby RA (2015). Impact of metabolic heterogeneity on tumor growth, invasion, and treatment outcomes. Cancer Res.

[34] Yang M, Soga T, Pollard PJ (2013). Oncometabolites: linking altered metabolism with cancer. J Clin Invest.

[35] Linehan WM, Spellman PT, Ricketts CJ (2016). Comprehensive molecular characterization of papillary renal-cell carcinoma. N Engl J Med.

[36] Linehan WM, Ricketts CJ (2019). The cancer genome atlas of renal cell carcinoma: findings and clinical implications. Nat Rev Urol.

[37] Vander Heiden MG, Cantley LC, Thompson CB (2009). Understanding the Warburg effect: the metabolic requirements of cell proliferation. Science.

[38] Ma Q, Xu Y, Liao H (2019). Identification and validation of key genes associated with non-small-cell lung cancer. J Cell Physiol.

[39] Harjunpää H, Llort Asens M, Guenther C (2019). Cell adhesion molecules and their roles and regulation in the immune and tumor microenvironment. Front Immunol.

[40] Afshar-Kharghan V (2017). The role of the complement system in cancer. J Clin Invest.

[41] Janssen BJ, Huizinga EG, Raaijmakers HC (2005). Structures of complement component C3 provide insights into the function and evolution of immunity. Nature.

[42] Yuan K, Ye J, Liu Z (2020). Complement C3 overexpression activates JAK2/STAT3 pathway and correlates with gastric cancer progression. J Exp Clin Cancer Res.

[43] Kim PY, Tan O, Diakiw SM (2014). Identification of plasma complement C3 as a potential biomarker for neuroblastoma using a quantitative proteomic approach. J Proteomics.

[44] Markiewski MM, Deangelis RA, Benencia F (2008). Modulation of the antitumor immune response by complement. Nat Immunol.

[45] Rutkowski MJ, Sughrue ME, Kane AJ (2010). Cancer and the complement cascade. Mol Cancer Res.

[46] Pang JS, Li ZK, Lin P (2019). The underlying molecular mechanism and potential drugs for treatment in papillary renal cell carcinoma: a study based on TCGA and Cmap datasets. Oncol Rep.

[47] Kitlinska J, Abe K, Kuo L (2005). Differential effects of neuropeptide Y on the growth and vascularization of neural crest-derived tumors. Cancer Res.

[48] Körner M, Waser B, Reubi JC (2008). High expression of neuropeptide Y1 receptors in ewing sarcoma tumors. Clin Cancer Res.

[49] Ruscica M, Dozio E, Boghossian S (2006). Activation of the Y1 receptor by neuropeptide Y regulates the growth of prostate cancer cells. Endocrinology.

[50] Lv X, Zhao F, Huo X (2016). Neuropeptide Y1 receptor inhibits cell growth through inactivating mitogen-activated protein kinase signal pathway in human hepatocellular carcinoma. Med Oncol.

[51] Ishihara K, Kamata M, Hayashi I (2002). Roles of bradykinin in vascular permeability and angiogenesis in solid tumor. Int Immunopharmacol.

[52] Wu J, Akaike T, Hayashida K (2002). Identification of bradykinin receptors in clinical cancer specimens and murine tumor tissues. Int J Cancer.

[53] Bettigole SE, Glimcher LH (2015). Endoplasmic reticulum stress in immunity. Annu Rev Immunol.

[54] Todd DJ, Lee AH, Glimcher LH (2008). The endoplasmic reticulum stress response in immunity and autoimmunity. Nat Rev Immunol.

[55] Rausch MP, Sertil AR (2015). A stressful microenvironment: opposing effects of the endoplasmic reticulum stress response in the suppression and enhancement of adaptive tumor immunity. Int Rev Immunol.

[56] Kobayashi M, Nagashio R, Jiang SX (2015). Calnexin is a novel sero-diagnostic marker for lung cancer. Lung Cancer.

[57] Turano C, Gaucci E, Grillo C (2011). ERp57/GRP58: a protein with multiple functions. Cell Mol Biol Lett.

[58] Hettinghouse A, Liu R, Liu CJ (2018). Multifunctional molecule ERp57: From cancer to neurodegenerative diseases. Pharmacol Ther.

[59] Choe MH, Min JW, Jeon HB (2015). ERp57 modulates STAT3 activity in radioresistant laryngeal cancer cells and serves as a prognostic marker for laryngeal cancer. Oncotarget.

[60] Shimoda T, Wada R, Kure S (2019). Expression of protein disulfide isomerase A3 and its clinicopathological association in gastric cancer. Oncol Rep.

[61] Takata H, Kudo M, Yamamoto T (2016). Increased expression of PDIA3 and its association with cancer cell proliferation and poor prognosis in hepatocellular carcinoma. Oncol Lett.

[62] Zou H, Wen C, Peng Z (2018). P4HB and PDIA3 are associated with tumor progression and therapeutic outcome of diffuse gliomas. Oncol Rep.

[63] Liu X, Song N, Liu Y (2015). Efficient induction of anti-tumor immune response in esophageal squamous cell carcinoma via dendritic cells expressing MAGE-A3 and CALR antigens. Cell Immunol.

[64] Siebenkäs C, Chiappinelli KB, Guzzetta AA (2017). Inhibiting DNA methylation activates cancer testis antigens and expression of the antigen processing and presentation machinery in colon and ovarian cancer cells. PLoS ONE.

[65] Langer R, Feith M, Siewert JR (2008). Expression and clinical significance of glucose regulated proteins GRP78 (BiP) and GRP94 (GP96) in human adenocarcinomas of the esophagus. BMC Cancer.

[66] Takahashi H, Wang JP, Zheng HC (2011). Overexpression of GRP78 and GRP94 is involved in colorectal carcinogenesis. Histol Histopathol.

[67] Yang Z, Zhuang L, Szatmary P (2015). Upregulation of heat shock proteins (HSPA12A, HSP90B1, HSPA4, HSPA5 and HSPA6) in tumour tissues is associated with poor outcomes from HBV-related early-stage hepatocellular carcinoma. Int J Med Sci.

[68] Zheng HC, Takahashi H, Li XH (2008). Overexpression of GRP78 and GRP94 are markers for aggressive behavior and poor prognosis in gastric carcinomas. Hum Pathol.

[69] Negroni L, Taouji S, Arma D (2014). Integrative quantitative proteomics unveils proteostasis imbalance in human hepatocellular carcinoma developed on nonfibrotic livers. Mol Cell Proteomics.

[70] Shen H, Huang J, Pei H (2013). Comparative proteomic study for profiling differentially expressed proteins between Chinese left- and right-sided colon cancers. Cancer Sci.

[71] Xia W, Zhuang J, Wang G (2017). P4HB promotes HCC tumorigenesis through downregulation of GRP78 and subsequent upregulation of epithelial-to-mesenchymal transition. Oncotarget.

[72] Zhang J, Wu Y, Lin YH (2018). Prognostic value of hypoxia-inducible factor-1 alpha and prolyl 4-hydroxylase beta polypeptide overexpression in gastric cancer. World J Gastroenterol.

[73] Althurwi SI, Yu JQ, Beale P (2020). Sequenced combinations of cisplatin and selected phytochemicals towards overcoming drug resistance in ovarian tumour models. Int J Mol Sci.

[74] Sun S, Lee D, Ho AS (2013). Inhibition of prolyl 4-hydroxylase, beta polypeptide (P4HB) attenuates temozolomide resistance in malignant glioma via the endoplasmic reticulum stress response (ERSR) pathways. Neuro Oncol.

[75] Zhu Z, He A, Lv T (2019). Overexpression of P4HB is correlated with poor prognosis in human clear cell renal cell carcinoma. Cancer Biomark Sect A Dis Mark.

[76] Nunno VD, Mollica V, Gatto L (2019). Prognostic impact of neutrophil-to-lymphocyte ratio in renal cell carcinoma: a systematic review and meta-analysis. Immunotherapy.

[77] Schaefer K, Webb NE, Pang M (2017). Galectin-9 binds to O-glycans on protein disulfide isomerase. Glycobiology.

[78] Bortnov V, Annis DS, Fogerty FJ (2018). Myeloid-derived growth factor is a resident endoplasmic reticulum protein. J Biol Chem.

[79] Hurst KE, Lawrence KA, Reyes Angeles L (2019). Endoplasmic reticulum protein disulfide isomerase shapes T cell efficacy for adoptive cellular therapy of tumors. Cells.

[80] Shalapour S, Karin M (2015). Immunity, inflammation, and cancer: an eternal fight between good and evil. J Clin Invest.

[81] Mollica V, Di Nunno V, Gatto L (2019). Resistance to systemic agents in renal cell carcinoma predict and overcome genomic strategies adopted by tumor. Cancers (Basel).

[82] Ciccarese C, Di Nunno V, Iacovelli R (2017). Future perspectives for personalized immunotherapy in renal cell carcinoma. Expert Opin Biol Ther.

[83] Shin SJ, Jeon YK, Kim PJ (2016). Clinicopathologic analysis of PD-L1 and PD-L2 expression in renal cell carcinoma: association with oncogenic proteins status. Ann Surg Oncol.

[84] Okadome K, Baba Y, Nomoto D (2020). Prognostic and clinical impact of PD-L2 and PD-L1 expression in a cohort of 437 oesophageal cancers. Br J Cancer.

[85] Mollica V, Di Nunno V, Gatto L (2019). Novel therapeutic approaches and targets currently under evaluation for renal cell carcinoma: waiting for the revolution. Clin Drug Investig.

[86] Santoni M, Massari F, Piva F (2018). Tivozanib for the treatment of renal cell carcinoma. Expert Opin Pharmacother.

[87] Pauken KE, Wherry EJ (2015). Overcoming T cell exhaustion in infection and cancer. Trends Immunol.

[88] Khan O, Giles JR, Mcdonald S (2019). TOX transcriptionally and epigenetically programs CD8 T cell exhaustion. Nature.

