# Supplementary material for: Identification of immunization-related new prognostic biomarkers for papillary renal cell carcinoma by integrated bioinformatics analysis
Source: BMC Med Genomics. 2021 Oct 7;14:241. doi: 10.1186/s12920-021-01092-w (PMC8499437; doi:10.1186/s12920-021-01092-w)
Supplement: Supplementary file 1 — Additional file 1: Table S1. DEGs of KIRP in GSE11151 downloaded from the GEO dataset, including 19 pRCC samples and five normal samples. Table S2. DEGs of KIRP in GSE15641 downloaded from the GEOdataset, containing 11 pRCC samples and 23 normal samples. Table S3. DEGs between pRCC and normal kidney tissue inTCGA database screened through the GEPIA website. Table S4. 32normal kidney tissues samples and 271tumor samples downloaded from TCGA GDC data portal. [file 12920_2021_1092_MOESM1_ESM.pdf]

Table S1. DEGs of KIRP in GSE11151

	logFC	AveExpr	t	P.Value	adj.P.Val	B
SLC16A10	-1.966823842	5.194256704	-26.50395382	1.02E-24	8.30E-21	46.20252588
ATP6V0A4	-3.741519784	6.663030318	-26.29281552	1.33E-24	8.30E-21	45.94584662
GEMIN7	-1.337976358	5.320124123	-24.48357509	1.42E-23	5.89E-20	43.65679327
OR1E1	-1.184363646	4.878363038	-24.01566109	2.69E-23	8.35E-20	43.03726531
GSTA3	-2.260552367	5.644248061	-23.22189614	8.12E-23	1.98E-19	41.95867115
MMRN2	-1.787731376	5.548739272	-23.00373143	1.11E-22	1.98E-19	41.65592852
GRAMD1B	-2.434319467	6.255076682	-22.97530962	1.15E-22	1.98E-19	41.61628372
ERBB4	-1.627787011	5.336660408	-22.90394879	1.28E-22	1.98E-19	41.51653527
RFC2	1.486157236	4.838143969	22.19358592	3.58E-22	4.32E-19	40.50697066
GAD1	-1.304211887	4.486890583	-22.19312099	3.58E-22	4.32E-19	40.50629983
DYSF	-2.038772847	6.517057887	-22.09459391	4.14E-22	4.32E-19	40.36383783
S1PR1	-1.939087235	5.113466596	-22.09024404	4.17E-22	4.32E-19	40.35753437
WNT5A	2.853697075	5.216313981	22.00035401	4.76E-22	4.56E-19	40.22700865
CHAC1	-1.651714839	6.301805112	-21.58327941	8.89E-22	6.94E-19	39.61470883
AQP5	-1.204346291	5.324951058	-21.580077	8.93E-22	6.94E-19	39.6099644
L1CAM	-1.774323678	6.29026725	-21.52514195	9.71E-22	7.10E-19	39.52847364
DKFZP434L187	-1.168430363	4.800864344	-21.43438764	1.11E-21	7.69E-19	39.39341958
C1RL	2.256111246	5.059075271	21.37557978	1.22E-21	7.97E-19	39.30561922
NTF3	-1.304898066	5.297484506	-21.27656755	1.42E-21	8.80E-19	39.15728125
KNTC1	1.229741123	4.283213114	21.1608492	1.69E-21	9.73E-19	38.98309463
PAX7	-1.241725883	5.635129336	-21.14871207	1.72E-21	9.73E-19	38.96477356
DIO3	-1.006278165	5.609699535	-20.99720792	2.17E-21	1.18E-18	38.73524949
HRG	-2.735084835	6.401827534	-20.77812731	3.05E-21	1.58E-18	38.40061385
KCNJ1	-4.437158953	7.82303716	-20.63821649	3.80E-21	1.89E-18	38.18519379
GRK1	-1.044859595	5.274838569	-20.41759677	5.38E-21	2.57E-18	37.84275698
BIK	-1.29218739	5.458218365	-20.16757903	8.01E-21	3.55E-18	37.45056281
HIST1H4E	-1.160597185	5.540559912	-20.05404371	9.60E-21	4.12E-18	37.27099593
AHR	2.766207874	5.782038706	19.79377139	1.46E-20	6.06E-18	36.85583855
LOC145678	-1.186144956	4.455588513	-19.77205124	1.51E-20	6.07E-18	36.82096978
DDN	-1.988762324	6.838998332	-19.53645963	2.23E-20	8.64E-18	36.44052256
OGFRL1	2.221957144	4.942135001	19.09857298	4.60E-20	1.68E-17	35.72233147
PAK6	-1.18170856	4.900870273	-19.02192876	5.23E-20	1.81E-17	35.59511815
HRC	-1.133838472	4.782881258	-18.93126632	6.09E-20	2.05E-17	35.44404938
RHBDF2	1.998698315	5.464447494	18.85592121	6.91E-20	2.26E-17	35.31801568
XPNPEP3	-1.275353202	5.070086157	-18.81043521	7.47E-20	2.29E-17	35.24171338
TEK	-1.293954062	6.634523635	-18.71134089	8.84E-20	2.50E-17	35.074919
AMELY	-1.003165904	4.745707438	-18.70543369	8.93E-20	2.50E-17	35.06495152
ARAP3	-1.490490692	5.930677677	-18.69835367	9.03E-20	2.50E-17	35.05300147
TFAP2A	-1.149496623	4.837806658	-18.48744108	1.30E-19	3.43E-17	34.69517721
ERVMER34-1	-1.477741061	4.724654345	-18.44039551	1.41E-19	3.56E-17	34.61487473
DHX38	1.082679294	6.0812892	18.35873369	1.62E-19	4.02E-17	34.47506002
ZAK	1.46581215	4.791215805	18.30488258	1.78E-19	4.29E-17	34.3825638

CEACAM1	-1.471413038	5.745823806	-18.29941146	1.79E-19	4.29E-17	34.37315319
HMP19	-1.34255067	5.214557285	-18.1540258	2.31E-19	5.41E-17	34.12218328
SPAG5	-2.852463247	5.96725278	-18.09828136	2.54E-19	5.83E-17	34.02549313
CHST2	-1.264248997	4.900382971	-18.08679267	2.60E-19	5.83E-17	34.00553369
SCN4A	-1.242233875	5.291910459	-18.08023724	2.63E-19	5.83E-17	33.99413996
MECOM	-1.607554921	5.105928483	-18.06843452	2.68E-19	5.85E-17	33.97361713
LOC102723620	-1.45656995	6.926760235	-18.04243008	2.81E-19	6.01E-17	33.92835913
PTGER3	-1.955024096	5.105786427	-18.02827848	2.88E-19	6.06E-17	33.9037061
IGSF6	1.50073598	3.836613341	17.99829301	3.03E-19	6.28E-17	33.8514142
RASL11B	-1.761025349	6.741556812	-17.82554239	4.11E-19	8.24E-17	33.54868453
SLC34A2	3.338490363	6.043833796	17.8158809	4.18E-19	8.25E-17	33.53167936
PRKG2	-1.409150379	5.357099383	-17.78196175	4.44E-19	8.63E-17	33.4719157
NAPG	1.404967163	5.072775158	17.76097919	4.61E-19	8.82E-17	33.43489671
DHX57	-1.249716426	5.359804407	-17.59359843	6.21E-19	1.15E-16	33.13824565
PACRG	-1.225156785	4.896537034	-17.59296237	6.22E-19	1.15E-16	33.13711375
FOXI1	-1.925837261	5.375795537	-17.49762772	7.38E-19	1.33E-16	32.96706935
FHL1	4.270143534	7.510807457	17.49570977	7.40E-19	1.33E-16	32.96364031
NBL1	2.571936202	7.525093131	17.44690246	8.08E-19	1.42E-16	32.87627196
MYBL2	-1.008490266	6.547473204	-17.27671787	1.10E-18	1.87E-16	32.5700033
GTF2I	2.273401144	5.056687403	17.259614	1.13E-18	1.91E-16	32.53908208
AHNAK2	4.247491987	5.264905825	17.20435053	1.25E-18	2.08E-16	32.43899743
IRS4	-1.020075085	4.691241259	-17.1754089	1.32E-18	2.16E-16	32.38647491
ZNF200	1.00645948	4.751093311	17.14455058	1.40E-18	2.26E-16	32.33039209
GJA4	-1.380582334	5.73243069	-17.12306759	1.45E-18	2.32E-16	32.29129824
RPL3L	-1.081297889	4.982987718	-17.07694045	1.58E-18	2.46E-16	32.2072189
TFAP2B	-1.801233796	4.537346849	-17.01649972	1.77E-18	2.64E-16	32.09676094
ERMAP	-1.132872224	4.476014082	-16.9608561	1.96E-18	2.86E-16	31.99477973
ANGPT1	-1.951741905	5.752968336	-16.9193628	2.11E-18	3.01E-16	31.91855087
GAGE3	-1.209556894	5.39366016	-16.91516641	2.13E-18	3.01E-16	31.91083284
PLA2G3	-1.507395898	5.43873733	-16.90306678	2.18E-18	3.01E-16	31.88857026
KLK6	-1.896705207	5.529104414	-16.90248329	2.18E-18	3.01E-16	31.88749633
ANXA9	-1.480598435	5.654315372	-16.87857342	2.28E-18	3.11E-16	31.84346331
FAM107A	-2.105526108	6.507611592	-16.85874577	2.37E-18	3.20E-16	31.80690886
LHX1	-1.177484598	4.937288754	-16.80126835	2.63E-18	3.47E-16	31.70074076
PAPPA2	-1.109982619	5.875568644	-16.79712515	2.65E-18	3.47E-16	31.69307611
GPR162	-1.111573299	5.441195237	-16.78344629	2.72E-18	3.51E-16	31.66775996
SPTBN4	-1.034990086	6.520066967	-16.77890207	2.74E-18	3.51E-16	31.65934596
VCAN	4.179901369	6.552342034	16.74219499	2.94E-18	3.72E-16	31.59131047
DNAH6	-1.381150421	5.775712638	-16.63306377	3.60E-18	4.44E-16	31.38830801
PTPRB	-1.323444634	4.86268813	-16.63176359	3.61E-18	4.44E-16	31.38588283
EPN3	-1.269821957	4.381743264	-16.5881846	3.91E-18	4.77E-16	31.30450626
MPPED2	-1.638650378	4.471288424	-16.50400511	4.59E-18	5.53E-16	31.14681572
RALA	1.440600662	4.516178998	16.48308171	4.77E-18	5.70E-16	31.1075182
HEYL	-1.081659855	5.827264155	-16.44425977	5.13E-18	6.08E-16	31.03449588

EGF	-4.768252143	7.387594816	-16.41986737	5.37E-18	6.30E-16	30.98854263
LOC103344931	-1.127172841	4.849227086	-16.41222336	5.45E-18	6.33E-16	30.97413047
GABARAPL3	-1.332625677	5.434626045	-16.36636004	5.95E-18	6.84E-16	30.88754347
MCTP2	1.177739458	5.04118728	16.33887394	6.26E-18	7.08E-16	30.83555649
ARG2	-2.477969713	6.410086218	-16.33306979	6.33E-18	7.09E-16	30.82456945
KCNE2	-1.007526133	6.003839337	-16.2833979	6.96E-18	7.67E-16	30.73041207
GPR32	-1.121384422	4.642264172	-16.28286643	6.97E-18	7.67E-16	30.72940337
NOTCH2	1.075664987	6.710034124	16.20756549	8.04E-18	8.69E-16	30.58621354
HIST1H1T	-1.154673442	5.520336573	-16.19896464	8.17E-18	8.76E-16	30.56982409
CPN2	-1.530721907	5.102211282	-16.11486596	9.60E-18	1.02E-15	30.40919615
RECQL	1.779334146	5.096686924	16.10492772	9.79E-18	1.02E-15	30.39016936
GRAMD4	1.044278116	5.441901135	16.10420362	9.80E-18	1.02E-15	30.38878272
CEL	-2.48794906	5.422263739	-16.04619675	1.10E-17	1.13E-15	30.27753489
KNG1	-5.043056051	7.61745915	-16.0234423	1.14E-17	1.16E-15	30.23380693
SEL1L3	2.308897314	7.224239695	16.02322411	1.15E-17	1.16E-15	30.23338738
FOLR3	-1.419407818	5.513835257	-16.0140326	1.17E-17	1.17E-15	30.21570938
TRIM5	1.45358326	4.488016019	15.97603312	1.25E-17	1.25E-15	30.1425383
ACPP	-3.108101228	5.606624786	-15.96568136	1.28E-17	1.26E-15	30.12258089
KLK11	-1.342546076	5.728549498	-15.94347444	1.34E-17	1.31E-15	30.07973251
TFPI2	4.20361947	5.772861401	15.90460585	1.44E-17	1.40E-15	30.00461987
SMARCE1	1.199628196	7.493060068	15.8078418	1.74E-17	1.67E-15	29.81698484
CABP5	-1.121996032	4.853456538	-15.73465983	2.00E-17	1.91E-15	29.67446743
MDK	1.624204206	5.104285683	15.73132709	2.02E-17	1.91E-15	29.66796455
CLDN8	-3.883622142	6.422369365	-15.64274986	2.40E-17	2.19E-15	29.494729
TES	2.265262563	6.952134876	15.57884246	2.72E-17	2.46E-15	29.36925766
CHST12	1.324769169	5.558471202	15.57238782	2.75E-17	2.46E-15	29.35656242
C3orf36	-1.421835597	5.3787057	-15.55770915	2.83E-17	2.52E-15	29.32767634
TASP1	1.395487997	5.297959899	15.48934748	3.24E-17	2.82E-15	29.19286358
LOC79999	-1.073375745	5.491335458	-15.48617811	3.26E-17	2.82E-15	29.18660203
FIP1L1	1.01232296	3.954587554	15.43679691	3.60E-17	3.08E-15	29.08891193
PPP2R2B	-1.165526465	5.178074642	-15.43438782	3.62E-17	3.08E-15	29.08413978
STAP1	-1.806542307	4.969201523	-15.39675541	3.90E-17	3.30E-15	29.00951785
NXPH4	-1.192315265	5.892360055	-15.34142206	4.35E-17	3.63E-15	28.89953608
EPHA7	2.443143593	4.16842981	15.32809611	4.47E-17	3.70E-15	28.8730027
PDE6A	-1.147271293	5.229685856	-15.31736251	4.56E-17	3.73E-15	28.85161785
SERPINA5	-3.333433865	7.911757569	-15.30423882	4.68E-17	3.78E-15	28.82545521
HOXD10	-1.646425185	4.967020727	-15.27784718	4.94E-17	3.96E-15	28.77278922
APBB1IP	1.675278272	5.042854399	15.26459712	5.07E-17	4.04E-15	28.74632119
LASP1	1.02739331	8.716406815	15.2535191	5.18E-17	4.10E-15	28.72417822
MYO1D	1.780841943	5.43632305	15.21758436	5.57E-17	4.33E-15	28.65226476
UMOD	-6.572116259	10.36722029	-15.19578392	5.82E-17	4.49E-15	28.6085727
TEX15	-1.325542926	4.929795995	-15.16407042	6.20E-17	4.73E-15	28.54492595
TUBB2B	-1.717692843	5.142471773	-15.11560022	6.83E-17	5.15E-15	28.44744967
SEMA3B	-1.224649048	5.002801777	-15.07080235	7.48E-17	5.57E-15	28.35714298

ESRRG	-2.202360291	6.530409954	-15.04149194	7.93E-17	5.84E-15	28.29794456
TUBAL3	-1.798184938	5.093444042	-15.01765802	8.33E-17	6.07E-15	28.24974132
CALU	1.47020566	6.651302623	15.01608498	8.35E-17	6.07E-15	28.24655784
ANK2	-2.36335998	6.031668891	-14.99273861	8.76E-17	6.33E-15	28.19927957
LOC101928104	-1.010927454	4.717337036	-14.95577129	9.44E-17	6.78E-15	28.12430164
LAMC1	1.645816164	7.565643951	14.94483466	9.65E-17	6.89E-15	28.10209238
PIPOX	-3.755719191	7.030430823	-14.93222605	9.90E-17	7.03E-15	28.0764723
GRHL2	-1.611449271	5.580604945	-14.92477319	1.01E-16	7.10E-15	28.06132064
EYA2	-1.147409985	5.166188544	-14.83094267	1.22E-16	8.40E-15	27.87006481
TFCP2L1	-1.311534539	7.403354439	-14.82200504	1.24E-16	8.51E-15	27.85179885
TM4SF5	-1.814108621	5.828937107	-14.80039335	1.29E-16	8.85E-15	27.80759592
CCL18	3.311673835	5.516524376	14.79239499	1.32E-16	8.94E-15	27.79122418
APOL3	-1.178015548	5.889175485	-14.74364813	1.45E-16	9.83E-15	27.69129862
GPRC5B	-1.444248693	6.096184111	-14.7314051	1.49E-16	1.00E-14	27.66616224
PTPRA	1.269277458	5.445056787	14.6784001	1.66E-16	1.11E-14	27.55715313
GNG4	-1.221795442	5.695330191	-14.62986784	1.84E-16	1.21E-14	27.45708025
SPHK2	-1.528709497	5.991938407	-14.57871337	2.04E-16	1.31E-14	27.35132795
BPIFA1	-1.254011219	5.706647084	-14.55392279	2.15E-16	1.37E-14	27.29997718
C5	-1.059647585	4.912247846	-14.46895125	2.56E-16	1.62E-14	27.12346701
DYRK2	1.277118375	5.24659894	14.44879202	2.67E-16	1.68E-14	27.08147621
DERL1	2.077621743	6.055347387	14.44131027	2.72E-16	1.70E-14	27.06588085
RARS	1.00935216	6.698123357	14.41418047	2.87E-16	1.79E-14	27.00927923
APLNR	-1.490632063	6.498462706	-14.4007212	2.95E-16	1.83E-14	26.98116919
MPZL2	2.149366921	4.685665418	14.33429737	3.39E-16	2.08E-14	26.84215286
ASB9	-1.963694962	5.650715092	-14.3103132	3.57E-16	2.16E-14	26.79183905
TMPRSS4	-1.715093671	6.31405282	-14.30040573	3.64E-16	2.20E-14	26.77103688
GATA3	-1.950396651	5.587957144	-14.27432031	3.85E-16	2.31E-14	26.71621545
FAM89B	1.307775233	6.986546966	14.2015799	4.48E-16	2.68E-14	26.56294931
LPPR1	-2.75749226	6.791891874	-14.12245389	5.30E-16	3.14E-14	26.39556721
APLP1	-1.112896044	5.771748964	-14.0978253	5.58E-16	3.29E-14	26.34332703
PYCARD	1.577388154	5.343317556	14.04941465	6.19E-16	3.63E-14	26.24044618
ZHX2	1.323267964	6.467831559	14.0405639	6.30E-16	3.68E-14	26.22160869
PALB2	1.118502167	5.772689242	14.02463646	6.52E-16	3.79E-14	26.18768757
JRKL	1.076716868	5.274545335	14.00935024	6.74E-16	3.89E-14	26.1551055
GRB14	-2.80415319	6.279145084	-14.00601324	6.78E-16	3.90E-14	26.14798933
HMGCS2	-3.535441085	7.150898207	-14.00045781	6.86E-16	3.93E-14	26.13613961
TYRP1	-2.729634258	5.405102238	-13.99134399	7.00E-16	3.99E-14	26.11669237
ACN9	1.232557157	3.992089582	13.97488832	7.25E-16	4.11E-14	26.08155545
LILRB4	1.088945627	3.798527039	13.96632352	7.38E-16	4.16E-14	26.06325553
GSTA1	-2.83976941	6.931493584	-13.96530202	7.40E-16	4.16E-14	26.06107239
PEG3	-1.087454833	4.503973185	-13.94681917	7.70E-16	4.31E-14	26.02155105
KLKB1	-1.905903713	5.79301823	-13.93835212	7.84E-16	4.35E-14	26.00343344
CEMP1	-1.051928823	4.579901045	-13.93781169	7.84E-16	4.35E-14	26.00227677
CASR	-1.629712312	6.210860514	-13.89947076	8.51E-16	4.68E-14	25.92013274

FAM184A	-1.269220619	6.006261221	-13.89780424	8.54E-16	4.68E-14	25.91655854
DUSP9	-2.956569056	6.556782487	-13.88993753	8.69E-16	4.74E-14	25.89968253
BAAT	-1.097024401	5.558858597	-13.87951925	8.89E-16	4.82E-14	25.87732206
PSMB9	1.557821179	6.491082629	13.86431675	9.18E-16	4.96E-14	25.84467149
NUP37	1.3463082	6.167271473	13.84871618	9.49E-16	5.11E-14	25.81113889
TPP2	1.337207622	6.07809718	13.82135244	1.01E-15	5.39E-14	25.75225576
MYD88	1.019236084	7.922504836	13.81119376	1.03E-15	5.44E-14	25.73037413
MAPK12	-1.192532977	5.042427707	-13.77922804	1.10E-15	5.80E-14	25.66144437
ELAVL3	-1.22557434	5.826369658	-13.74573071	1.18E-15	6.19E-14	25.58908777
PDE2A	-1.295291252	6.437240464	-13.73934103	1.20E-15	6.25E-14	25.57527116
ANXA1	2.806065897	8.567927486	13.71928118	1.25E-15	6.49E-14	25.53186496
ENTPD5	-2.165205756	5.908311147	-13.68307676	1.36E-15	6.96E-14	25.45340883
EGLN3	1.9922846	5.24859316	13.65552546	1.44E-15	7.30E-14	25.39360424
POT1	1.495937191	5.853045474	13.64188254	1.48E-15	7.46E-14	25.36395799
POU5F1B	1.179375252	6.813752117	13.58116665	1.69E-15	8.42E-14	25.23176345
HS3ST1	1.813244752	5.225052852	13.58027919	1.69E-15	8.42E-14	25.22982809
ITIH5	-1.48988522	6.041820548	-13.56451177	1.75E-15	8.65E-14	25.19542757
RXRA	-1.034916259	5.942304544	-13.56095477	1.77E-15	8.68E-14	25.18766314
TPK1	2.986336197	6.079589481	13.47001194	2.15E-15	1.03E-13	24.98865367
CKMT2	-1.108959399	5.574765062	-13.45760042	2.21E-15	1.06E-13	24.96141969
ANGPTL3	-2.249890902	4.654118963	-13.45379231	2.23E-15	1.06E-13	24.95306016
RB1	1.080774044	4.816112616	13.44067677	2.30E-15	1.08E-13	24.92425626
GPR116	-3.024589367	7.564376686	-13.43958246	2.30E-15	1.08E-13	24.92185207
CALML3	-1.043062344	5.588139902	-13.40046507	2.51E-15	1.17E-13	24.83582055
SLC12A1	-5.868645286	8.207814347	-13.38730084	2.58E-15	1.20E-13	24.80682832
C17orf62	1.225447607	5.783742338	13.34538038	2.83E-15	1.29E-13	24.71437076
ZNF587B	-1.05940606	6.079818081	-13.31893306	3.00E-15	1.36E-13	24.65593474
OCEL1	-1.017695153	6.802332024	-13.31810069	3.01E-15	1.36E-13	24.65409428
ART4	-1.04357291	4.499331842	-13.261479	3.41E-15	1.54E-13	24.52870732
NELL1	-2.358009294	5.955375752	-13.25530141	3.45E-15	1.55E-13	24.51500455
EGFL7	-1.213407495	6.64488494	-13.23704395	3.59E-15	1.61E-13	24.47448086
KIF5B	1.232795453	6.956470242	13.22150193	3.72E-15	1.66E-13	24.43995348
NCK1	1.244604678	5.721408455	13.18384117	4.04E-15	1.79E-13	24.35617063
AGTR1	-1.777140863	5.230844089	-13.16650285	4.20E-15	1.85E-13	24.3175425
KIR3DL3	-1.064216072	5.662650355	-13.14824761	4.38E-15	1.92E-13	24.27683334
SLC43A1	-1.058435714	5.831195396	-13.12314397	4.63E-15	2.00E-13	24.2207881
HOXD11	-1.016686698	5.427956293	-13.11839447	4.68E-15	2.02E-13	24.21017622
NFKB1	1.074941924	6.989064455	13.10321432	4.84E-15	2.08E-13	24.17624112
MFGE8	-1.337881039	6.387696735	-13.10240905	4.85E-15	2.08E-13	24.17444018
SLC4A1	-2.205376873	7.79493906	-13.0849249	5.04E-15	2.15E-13	24.1353191
MMP11	1.263984679	5.37911157	13.08308456	5.06E-15	2.15E-13	24.13119921
SFRP1	-2.556922801	6.238475875	-13.06479252	5.27E-15	2.23E-13	24.09022778
CTSC	2.331157929	9.395241929	13.04359209	5.53E-15	2.32E-13	24.04269238
SCIN	1.315718718	7.111738608	13.03881408	5.59E-15	2.34E-13	24.03197182

CDH9	-1.050583611	4.532176616	-13.01638769	5.87E-15	2.45E-13	23.98161686
STRA6	-1.376075002	6.584705154	-13.01298258	5.92E-15	2.46E-13	23.97396601
C7orf49	1.738999328	7.371162309	12.9768096	6.42E-15	2.65E-13	23.89260468
IL1R2	-1.749961122	5.24495241	-12.97266292	6.48E-15	2.67E-13	23.88326789
MET	2.445152518	7.021976609	12.94311289	6.92E-15	2.82E-13	23.81667288
SOAT1	1.68319898	4.253627356	12.92294581	7.24E-15	2.94E-13	23.77116383
ADAM10	2.107003641	6.448871849	12.91394271	7.39E-15	2.98E-13	23.75083173
TFPI	1.47512343	5.151300633	12.8276343	8.98E-15	3.58E-13	23.55542549
CCND2	1.106182876	5.937419398	12.81494675	9.24E-15	3.67E-13	23.52662497
PYGL	1.493169095	5.307418887	12.81307374	9.28E-15	3.67E-13	23.52237164
IFI27	2.890099143	8.039621599	12.77689083	1.01E-14	3.94E-13	23.44012256
MANSC1	1.136521115	4.626614774	12.77623348	1.01E-14	3.94E-13	23.43862684
COPG2IT1	-1.161226054	5.113386333	-12.76422098	1.04E-14	4.02E-13	23.41128488
MARCKS	1.561606868	6.635702071	12.76265291	1.04E-14	4.03E-13	23.40771446
SEMA3G	-2.174668711	6.183063936	-12.71955868	1.15E-14	4.39E-13	23.30947511
ERV3-2	2.798854234	5.353069337	12.70716745	1.18E-14	4.48E-13	23.28118604
RPA1	1.165954194	6.718801969	12.68830814	1.23E-14	4.65E-13	23.23809475
TSPAN8	-3.791925225	6.446176564	-12.60182486	1.50E-14	5.59E-13	23.03993853
TBX3	-1.707651277	4.951848355	-12.57283589	1.60E-14	5.93E-13	22.97331366
KANSL1L	2.256330722	5.479866295	12.54948735	1.69E-14	6.23E-13	22.91957767
NFASC	-1.485988872	5.532069111	-12.53531447	1.75E-14	6.40E-13	22.88692676
CA4	-2.137733608	5.821615004	-12.51228902	1.84E-14	6.72E-13	22.83382934
ARL6IP5	2.03202775	8.654225116	12.51160395	1.84E-14	6.72E-13	22.83224856
DOC2A	1.150009913	4.51981558	12.50994226	1.85E-14	6.72E-13	22.82841402
CPOX	1.09366598	4.414648261	12.50934193	1.85E-14	6.72E-13	22.8270286
MAP1LC3B	1.63457439	7.812215517	12.50278369	1.88E-14	6.80E-13	22.81189087
ME3	-1.155263364	6.921187779	-12.49687183	1.91E-14	6.87E-13	22.7982406
ATP2B1	1.277721959	5.017933418	12.49350079	1.92E-14	6.90E-13	22.79045508
TNFAIP8	1.544456046	6.380046302	12.48551671	1.96E-14	7.01E-13	22.77201005
ARPC1B	2.176511523	7.74005726	12.48344208	1.97E-14	7.02E-13	22.76721592
CLDN1	2.03633718	5.072446007	12.48181989	1.97E-14	7.03E-13	22.76346692
CCDC71	-1.006316296	5.997277891	-12.45252472	2.11E-14	7.50E-13	22.69570833
PCDH9	-1.21782767	4.819935293	-12.42011098	2.28E-14	8.01E-13	22.62061394
ABCC2	-1.542408338	5.315036956	-12.41016509	2.33E-14	8.15E-13	22.59754597
FLOT2	1.46755979	6.627553315	12.41011138	2.33E-14	8.15E-13	22.59742136
FOXH1	-1.036280613	5.601017015	-12.40750319	2.34E-14	8.18E-13	22.59137002
ARF6	1.449785606	7.564128066	12.4044977	2.36E-14	8.21E-13	22.58439588
COL9A3	-1.00419221	4.587317766	-12.39036655	2.44E-14	8.45E-13	22.55159007
APOL1	1.263752047	7.042767014	12.38830441	2.45E-14	8.45E-13	22.5468007
ITGB2	1.698396503	6.370223807	12.36624242	2.58E-14	8.82E-13	22.49552851
KRR1	1.015840583	4.828021914	12.34762457	2.69E-14	9.19E-13	22.45221387
SYNGR3	1.20816263	4.6300988	12.33535458	2.77E-14	9.43E-13	22.42364422
SEZ6L2	1.954105196	6.011356488	12.32822074	2.81E-14	9.56E-13	22.40702513
WT1	-1.817286159	5.126167216	-12.31380437	2.91E-14	9.83E-13	22.37342139

ZCCHC10	1.406381733	4.394181444	12.29071125	3.07E-14	1.03E-12	22.31953911
TUSC3	1.682641286	6.578999537	12.28350675	3.12E-14	1.04E-12	22.30271562
SLC1A6	-1.048406215	5.467659487	-12.2699017	3.22E-14	1.07E-12	22.27092848
CDH18	-1.039439773	5.145591292	-12.23881823	3.46E-14	1.15E-12	22.19821832
TSPAN7	-2.29706147	6.635660673	-12.20700572	3.73E-14	1.23E-12	22.1236787
TXLNGY	-1.843749496	4.717634718	-12.19047414	3.88E-14	1.27E-12	22.08489407
ANXA2	1.533886673	10.34771223	12.17493378	4.02E-14	1.31E-12	22.04840394
ATP6V1B1	-2.474032302	5.785588456	-12.15208702	4.24E-14	1.37E-12	21.99470319
PNMA2	1.598384557	5.112039952	12.10690877	4.71E-14	1.51E-12	21.88832131
ATF2	1.025764212	4.778110579	12.09213387	4.88E-14	1.56E-12	21.85347536
PLA2G7	1.496356429	3.884377473	12.07768733	5.05E-14	1.60E-12	21.81937748
IVL	-1.097899445	5.1268758	-12.04906307	5.40E-14	1.69E-12	21.75173911
IGFBP5	-1.753168477	6.731070772	-12.04208725	5.49E-14	1.72E-12	21.73523989
TRRAP	1.314679809	5.652421914	12.0381121	5.54E-14	1.73E-12	21.72583512
MREG	1.708518115	4.828969465	12.02552471	5.71E-14	1.77E-12	21.69604171
ASCC3	1.446494962	5.732915644	12.01959141	5.79E-14	1.78E-12	21.68199117
CSDC2	-1.501623804	6.751619438	-12.00015957	6.06E-14	1.86E-12	21.635944
SYNDIG1	-1.387837667	6.159328898	-11.98635109	6.26E-14	1.92E-12	21.60319357
BBS4	1.200684201	5.190165229	11.95987696	6.66E-14	2.02E-12	21.54033627
RNF219	1.023384429	4.218172905	11.95758772	6.70E-14	2.03E-12	21.53489682
RHBG	-1.335837361	6.422128979	-11.92442555	7.24E-14	2.17E-12	21.45602627
SGK2	-1.104834452	7.735720046	-11.92349309	7.26E-14	2.17E-12	21.4538066
MYOM2	-1.28407392	5.225189924	-11.8971222	7.73E-14	2.30E-12	21.390986
TMSB10	1.746102912	10.65958686	11.84724473	8.70E-14	2.54E-12	21.2719283
OSBPL11	1.083105391	5.678393999	11.84510728	8.74E-14	2.55E-12	21.26681919
RAMP2	-1.318652819	6.095415259	-11.84417847	8.76E-14	2.55E-12	21.26459889
TBX2	-1.232056343	5.682009605	-11.83811997	8.89E-14	2.58E-12	21.25011347
POU5F1P3	1.192022562	5.232817822	11.83521208	8.95E-14	2.59E-12	21.24315929
NT5C	1.088094113	5.895670867	11.8315385	9.03E-14	2.60E-12	21.23437241
LDB2	-2.505419192	6.378587863	-11.80045267	9.72E-14	2.78E-12	21.15994968
CHL1	2.973084633	6.128409741	11.78437896	1.01E-13	2.88E-12	21.12141956
CDCP1	1.78167723	5.111526721	11.7727465	1.04E-13	2.95E-12	21.09351506
GPC4	2.098178274	6.480794749	11.71406621	1.19E-13	3.38E-12	20.95248835
AP5S1	-1.048554525	6.104385674	-11.70418589	1.22E-13	3.44E-12	20.92869989
COG5	1.134999313	5.608830287	11.69408315	1.25E-13	3.50E-12	20.90436307
CLIP1	1.096502667	5.807665104	11.69346195	1.26E-13	3.50E-12	20.90286621
TNKS2	1.010276198	6.258905635	11.69334556	1.26E-13	3.50E-12	20.90258576
SLC28A1	-1.195518275	5.933348204	-11.61885364	1.50E-13	4.11E-12	20.72273163
ITPR1	-1.470756773	5.503157181	-11.61468313	1.52E-13	4.14E-12	20.71264139
SERPINH1	1.088528245	7.419839183	11.61032464	1.53E-13	4.16E-12	20.70209398
SHFM1	1.11851517	7.444827024	11.58658159	1.62E-13	4.37E-12	20.6445939
SCN10A	-1.264889717	6.036028852	-11.58552479	1.63E-13	4.37E-12	20.64203291
ALOX5AP	1.865731292	6.297909404	11.55991497	1.73E-13	4.64E-12	20.57992775
HAO2	-3.669979608	7.861049461	-11.55329525	1.76E-13	4.70E-12	20.56386093

DACH1	-1.308025913	4.282166664	-11.55098566	1.77E-13	4.72E-12	20.55825397
CYP4F3	-3.245294613	6.054123499	-11.53704524	1.83E-13	4.87E-12	20.52439654
CYP2A13	-1.12100355	5.190684589	-11.52931082	1.86E-13	4.95E-12	20.50560102
EFS	-1.007093732	5.32036903	-11.52858092	1.87E-13	4.95E-12	20.50382688
VDR	-1.132276283	5.885454773	-11.49524409	2.02E-13	5.32E-12	20.42272385
EPB41L4B	-1.142346055	5.089840474	-11.48259646	2.09E-13	5.44E-12	20.39191696
FAM208B	1.591486662	5.150220262	11.47063009	2.15E-13	5.57E-12	20.36275057
PDLIM1	1.537759378	8.493126703	11.4530618	2.24E-13	5.78E-12	20.31989698
TNFRSF10C	1.178589469	3.960948871	11.41299248	2.47E-13	6.29E-12	20.22200919
EVI2B	1.682004639	4.61623783	11.40866459	2.50E-13	6.34E-12	20.21142396
DEGS1	1.359130699	6.218808203	11.39652492	2.57E-13	6.52E-12	20.18171972
PDIA4	1.123048396	6.405092086	11.3771578	2.69E-13	6.81E-12	20.13429154
PDZD2	-2.236331132	5.153186795	-11.36896991	2.75E-13	6.90E-12	20.1142257
GALNT12	1.978195465	4.881682179	11.34198525	2.94E-13	7.33E-12	20.04803387
ZBTB18	1.50258268	5.818031291	11.31285397	3.15E-13	7.84E-12	19.97647112
ABCA1	1.107797437	6.017578886	11.30577574	3.21E-13	7.94E-12	19.95906648
GLRB	1.90120875	4.540914395	11.29615324	3.28E-13	8.11E-12	19.93539539
FUT8	1.709955205	6.120975965	11.2953609	3.29E-13	8.11E-12	19.9334457
CELF2	1.241327331	5.254827126	11.29200863	3.32E-13	8.15E-12	19.92519605
DNASE1L3	-1.967692958	5.991307841	-11.28362909	3.39E-13	8.30E-12	19.90456837
LCN2	4.049916972	6.300379252	11.26667784	3.53E-13	8.62E-12	19.86281219
PLVAP	-2.115244892	7.067504262	-11.26363512	3.55E-13	8.67E-12	19.85531311
LPXN	1.282342782	6.848069346	11.26187664	3.57E-13	8.69E-12	19.8509786
PSMB8	1.667738436	6.756663386	11.25862945	3.60E-13	8.74E-12	19.84297352
LPGAT1	1.96812988	5.61392504	11.24956349	3.68E-13	8.92E-12	19.82061657
SNAP25	1.001118134	5.308564184	11.19422564	4.21E-13	1.02E-11	19.68392106
TLR2	1.102047262	5.600162248	11.18442732	4.32E-13	1.03E-11	19.65967592
PPP1R16B	-1.71662881	5.719969018	-11.18274207	4.34E-13	1.03E-11	19.65550465
THEMIS2	1.575846567	5.660762823	11.13354584	4.89E-13	1.15E-11	19.53357374
NOTCH4	-1.024730004	6.069380994	-11.12981144	4.94E-13	1.16E-11	19.52430534
ZNF217	1.865930311	5.577345793	11.11311862	5.15E-13	1.20E-11	19.48285337
SCNN1B	-1.786782343	6.205635571	-11.10642227	5.23E-13	1.21E-11	19.46621467
ATP13A3	1.408812	6.263792356	11.10472993	5.25E-13	1.22E-11	19.46200874
MED17	1.403059414	6.031431021	11.0896849	5.45E-13	1.26E-11	19.42460119
FGD6	1.196793939	4.358687715	11.07445473	5.66E-13	1.30E-11	19.38670334
TDRD7	1.044729449	5.798068117	11.05878369	5.89E-13	1.34E-11	19.34767696
GOLT1B	1.134080866	4.894041395	11.0281087	6.35E-13	1.44E-11	19.27119305
PRODH2	-2.892822133	8.10151684	-11.02393676	6.42E-13	1.45E-11	19.2607814
CEBPA	1.058151009	6.39716465	11.01870894	6.50E-13	1.47E-11	19.24773149
PARN	1.083888145	7.210254631	11.00197598	6.78E-13	1.52E-11	19.205938
LSR	1.148158552	5.973960651	10.98118752	7.14E-13	1.60E-11	19.15396435
PODXL	-3.401721477	8.148501603	-10.97468927	7.25E-13	1.62E-11	19.13770641
TCF21	-2.316449318	5.558532202	-10.97179226	7.30E-13	1.63E-11	19.13045661
MAD1L1	1.599226976	4.604429412	10.96994922	7.34E-13	1.63E-11	19.12584382

MT1G	-2.245773574	8.376584767	-10.96206181	7.48E-13	1.66E-11	19.10609805
HSD17B14	-1.453417413	6.344618242	-10.95228442	7.67E-13	1.70E-11	19.08160954
OR52A1	-1.180211545	4.991309944	-10.94806913	7.75E-13	1.71E-11	19.07104805
GNAI2	1.218496158	7.479335322	10.93284044	8.05E-13	1.77E-11	19.03287296
SLC29A2	-1.212859846	4.733278923	-10.92717218	8.16E-13	1.78E-11	19.01865611
THY1	-2.166303928	7.454885772	-10.91841083	8.34E-13	1.82E-11	18.99667307
TLX1	-1.017687278	4.604483726	-10.91292592	8.46E-13	1.83E-11	18.98290582
CLU	2.549578919	7.849206168	10.91238607	8.47E-13	1.83E-11	18.98155057
B4GALT5	1.596839363	7.872697895	10.9039988	8.65E-13	1.86E-11	18.96049015
ALLC	-1.237847789	5.353844745	-10.90399491	8.65E-13	1.86E-11	18.96048036
ELF5	-1.37977766	5.122792098	-10.90193557	8.69E-13	1.87E-11	18.95530796
BAZ1A	1.166071088	4.75871282	10.90115505	8.71E-13	1.87E-11	18.95334741
LMO3	-1.427774669	6.09337132	-10.89457415	8.85E-13	1.89E-11	18.9368139
PHGDH	-1.518594277	6.315644939	-10.89397907	8.87E-13	1.89E-11	18.93531857
FZD1	2.2469611	6.378638075	10.88795399	9.00E-13	1.92E-11	18.92017605
RCBTB2	1.449457863	7.117048796	10.84810522	9.94E-13	2.08E-11	18.81990669
REEP1	-1.097805603	5.409841204	-10.82229607	1.06E-12	2.21E-11	18.75485369
WLS	-1.49044411	7.227550425	-10.81823261	1.07E-12	2.23E-11	18.74460364
EMCN	-2.82775594	6.889253798	-10.8061865	1.10E-12	2.29E-11	18.71420469
DNAJC12	-2.544603679	6.20445056	-10.80203342	1.12E-12	2.30E-11	18.70371979
SEPT9	1.000173324	6.793090513	10.78367367	1.17E-12	2.38E-11	18.65734157
C7	-3.671644496	8.633773439	-10.76001089	1.24E-12	2.49E-11	18.59750234
GPC5	-2.603555705	5.119338297	-10.75964061	1.24E-12	2.49E-11	18.59656538
ISG20	1.016575181	5.038770806	10.75013834	1.27E-12	2.55E-11	18.5725146
BMP2	1.297451952	5.211184943	10.73150507	1.33E-12	2.65E-11	18.52531845
RPS6KA3	1.329174058	5.221931819	10.72246353	1.36E-12	2.71E-11	18.50240073
SMCP	-1.125531602	6.481865325	-10.71984637	1.37E-12	2.72E-11	18.49576499
FBRS	-1.05100911	6.133977341	-10.70734738	1.42E-12	2.80E-11	18.4640617
CLDN3	2.699338546	6.957119823	10.69351131	1.47E-12	2.89E-11	18.42894308
PMS2P8	1.131659459	5.345293073	10.68022482	1.52E-12	2.97E-11	18.39519576
LRRFIP2	1.074935637	6.326858488	10.66857194	1.56E-12	3.05E-11	18.36557869
TLR3	1.649560786	5.610538647	10.65113169	1.63E-12	3.16E-11	18.3212191
C1orf95	-1.067888857	6.297804419	-10.64001699	1.68E-12	3.24E-11	18.29292784
TMEM43	1.190148106	8.472218312	10.62927614	1.72E-12	3.31E-11	18.26557277
ALOX5	1.073254978	4.994728776	10.62827238	1.73E-12	3.31E-11	18.2630156
NES	-1.711459445	7.625156196	-10.6258605	1.74E-12	3.32E-11	18.25687056
TIMP1	2.212414983	8.795812109	10.62082174	1.76E-12	3.35E-11	18.24403021
ALDH5A1	-1.106554623	5.715951443	-10.57255335	1.99E-12	3.77E-11	18.12085837
HRH1	1.223559358	5.5181296	10.56932701	2.01E-12	3.79E-11	18.11261444
TMEM248	1.041449306	7.650259826	10.56043427	2.05E-12	3.85E-11	18.08988466
TREM2	1.097106082	6.012626171	10.54980137	2.11E-12	3.94E-11	18.06269342
RNASET2	1.897840038	7.886201103	10.54073013	2.16E-12	4.02E-11	18.03948402
HDC	-1.025496778	5.643747706	-10.51976977	2.28E-12	4.22E-11	17.98581412
PLCL1	-2.362434974	5.780831576	-10.51597057	2.30E-12	4.25E-11	17.97607991

VKORC1	1.612352862	7.978435469	10.50605448	2.36E-12	4.35E-11	17.95066426
CRELD1	1.038620389	5.395637511	10.49192387	2.44E-12	4.49E-11	17.91442412
IGFBP6	2.358436622	7.685213198	10.48805158	2.47E-12	4.52E-11	17.90448846
RAPGEF3	-1.020870024	6.584634127	-10.45665839	2.67E-12	4.85E-11	17.82386579
LSM5	1.044179618	5.079555075	10.44519764	2.75E-12	4.98E-11	17.79440047
CAPZA1	1.15923625	8.023106393	10.42648196	2.89E-12	5.14E-11	17.74624573
PDGFRA	-1.169982009	4.977962746	-10.40728202	3.03E-12	5.37E-11	17.69679709
BHLHE41	3.088679855	5.575970269	10.40671231	3.04E-12	5.37E-11	17.69532909
PAK3	-1.002366756	6.147687114	-10.39738111	3.11E-12	5.47E-11	17.67127874
PROZ	-1.374828577	6.537224311	-10.3737438	3.31E-12	5.76E-11	17.6103044
DUSP2	-1.536228602	5.776377935	-10.3529416	3.49E-12	6.05E-11	17.5565826
DIO1	-3.967381129	6.263451749	-10.34695547	3.54E-12	6.13E-11	17.54111285
AKR1C1	1.440811445	7.638124187	10.33578319	3.64E-12	6.29E-11	17.51222803
PPM1D	1.207019013	4.961601904	10.33535097	3.65E-12	6.29E-11	17.51111024
HMGB3	1.239640262	6.387316864	10.30368458	3.96E-12	6.80E-11	17.42914886
RAC1	1.087738242	9.863844823	10.28270979	4.18E-12	7.14E-11	17.37478761
BTBD1	1.267668735	7.447967093	10.27115028	4.30E-12	7.31E-11	17.34480356
QPCT	2.42159549	4.780539283	10.25501291	4.49E-12	7.59E-11	17.30291563
HHLA2	1.818564041	3.871000567	10.24208969	4.64E-12	7.80E-11	17.26934598
MTMR6	1.173719452	5.713017129	10.23402878	4.74E-12	7.95E-11	17.24839564
EXO1	-1.013868713	5.332337625	-10.23231005	4.76E-12	7.95E-11	17.24392753
STON1	-1.853498793	5.742097471	-10.22429786	4.86E-12	8.09E-11	17.22309352
KLHL3	-2.261120038	7.080989265	-10.21399599	4.99E-12	8.28E-11	17.19629326
MTMR2	1.229513737	6.189272708	10.20904806	5.05E-12	8.36E-11	17.18341625
S100A11	1.800909525	8.619864548	10.20817998	5.06E-12	8.37E-11	17.18115674
UBA3	1.175806824	6.294258897	10.19276281	5.27E-12	8.68E-11	17.14101113
TNS1	-1.076977118	7.103920282	-10.18828757	5.33E-12	8.77E-11	17.12935193
PLOD3	1.160552527	7.480038897	10.18152383	5.42E-12	8.88E-11	17.11172559
PYGB	1.535979975	8.005949133	10.18017693	5.44E-12	8.90E-11	17.10821484
TCEAL2	-2.306329251	4.907283569	-10.17662191	5.49E-12	8.97E-11	17.09894739
IL18	1.245547735	3.93544327	10.17073587	5.58E-12	9.09E-11	17.08359963
UPB1	-2.835451514	6.498393619	-10.16391019	5.68E-12	9.24E-11	17.06579604
RHCG	-2.774887826	6.447586068	-10.15669533	5.78E-12	9.40E-11	17.04697071
ACADSB	-1.33679469	4.583125878	-10.15043483	5.88E-12	9.53E-11	17.03062997
NPY1R	-2.734600614	6.393356002	-10.1481214	5.91E-12	9.57E-11	17.0245903
TNFSF13	1.30942583	8.724020135	10.12177796	6.33E-12	1.02E-10	16.95576583
TNFAIP6	3.497747477	5.210003201	10.12152056	6.34E-12	1.02E-10	16.9550929
TMEM87A	1.195813177	7.358799547	10.11976058	6.37E-12	1.02E-10	16.95049149
RNF186	-1.952506706	6.030416519	-10.11933425	6.37E-12	1.02E-10	16.94937679
CHI3L1	-1.367288333	5.638030336	-10.11589215	6.43E-12	1.02E-10	16.94037615
GPR125	1.827703244	6.39402086	10.1035875	6.64E-12	1.05E-10	16.90818836
C11orf95	-1.108564924	6.449797488	-10.09078922	6.86E-12	1.08E-10	16.87468815
SLC5A2	-1.943098841	7.521811381	-10.08886604	6.90E-12	1.08E-10	16.86965224
RGS1	1.774411666	4.831977646	10.05348089	7.56E-12	1.18E-10	16.77690841

PPP1R12A	1.027461082	5.041790695	10.04182493	7.80E-12	1.21E-10	16.74632226
LY86	1.207864928	6.573147163	10.0262414	8.12E-12	1.26E-10	16.70540191
GP2	-1.133823935	5.108490044	-10.00863863	8.50E-12	1.31E-10	16.65914089
EAF2	-2.14441552	5.849142415	-10.00344754	8.62E-12	1.33E-10	16.64549065
PVALB	-2.362977646	6.439178611	-9.978291375	9.20E-12	1.42E-10	16.57929095
INPP5J	-1.131345436	6.571234237	-9.947249015	9.98E-12	1.52E-10	16.49748672
HOXA1	1.172056033	4.59838362	9.947003272	9.99E-12	1.52E-10	16.49683862
CLCNKB	-2.963198057	7.380127905	-9.946063493	1.00E-11	1.53E-10	16.49436007
ELTD1	-1.750256466	5.202724525	-9.931852344	1.04E-11	1.57E-10	16.45686571
MST1L	-1.393861932	4.580536343	-9.922513597	1.07E-11	1.61E-10	16.43221213
NPHS1	-1.490986385	5.287463729	-9.911224276	1.10E-11	1.65E-10	16.40239388
APOC1	1.777282445	7.137381671	9.901852282	1.12E-11	1.68E-10	16.3776271
STX4	1.174993742	6.388688244	9.862054267	1.25E-11	1.85E-10	16.27232686
CLIP2	1.459055702	5.140948384	9.859166909	1.26E-11	1.86E-10	16.2646792
TGFB1I1	-2.189252576	6.226823339	-9.858791817	1.26E-11	1.86E-10	16.26368562
JAK1	1.112695795	8.619268259	9.853748113	1.28E-11	1.88E-10	16.25032364
COLEC11	-1.875745278	6.841684514	-9.840622396	1.32E-11	1.95E-10	16.21553482
TYROBP	1.730344	6.574523107	9.816225305	1.41E-11	2.06E-10	16.15081191
IQCC	-1.046237201	5.356239321	-9.804730102	1.45E-11	2.11E-10	16.12028928
CCDC109B	1.19217015	5.941792352	9.791961784	1.50E-11	2.18E-10	16.0863659
EREG	1.270977298	3.41357025	9.777404545	1.56E-11	2.25E-10	16.04766355
HLA-J	1.689359544	8.392983943	9.754312089	1.66E-11	2.37E-10	15.98621223
UBE2S	1.28467678	5.183504935	9.746491906	1.69E-11	2.42E-10	15.96538611
PIBF1	1.071869424	4.454368803	9.742556461	1.71E-11	2.44E-10	15.9549025
NARF	1.14188216	6.544290963	9.716218344	1.83E-11	2.59E-10	15.88468832
PLCH2	-1.136662482	5.403652406	-9.712923177	1.85E-11	2.61E-10	15.87589741
SLC5A12	-2.775772623	4.814525753	-9.702161324	1.90E-11	2.68E-10	15.84717686
FABP1	-4.366740561	6.333706589	-9.666750947	2.09E-11	2.92E-10	15.75256894
G6PC	-3.126311315	5.094341256	-9.665176006	2.10E-11	2.92E-10	15.74835728
HADH	-1.352925757	7.591546123	-9.657819502	2.14E-11	2.97E-10	15.7286804
DARS	1.183268542	7.103529492	9.636091238	2.27E-11	3.12E-10	15.67052125
PDE1A	-2.551903261	6.087058894	-9.633449559	2.29E-11	3.14E-10	15.66344617
FAS	1.885128335	5.606294452	9.620250189	2.37E-11	3.24E-10	15.62808129
MAPT	-1.011942595	5.61323073	-9.604745531	2.47E-11	3.36E-10	15.58651085
CDH3	-1.233649965	5.691726159	-9.599481537	2.50E-11	3.40E-10	15.57239011
MGAM	-3.365651811	6.175063029	-9.598499393	2.51E-11	3.41E-10	15.5697551
LAMA3	2.593211379	6.260526794	9.581075499	2.63E-11	3.56E-10	15.52298725
LRRC42	1.049820561	4.915334271	9.568088479	2.72E-11	3.68E-10	15.48810276
SDS	1.153065735	5.264006466	9.562966654	2.76E-11	3.72E-10	15.47433897
ALDH18A1	1.302078299	7.161231052	9.553630229	2.83E-11	3.80E-10	15.44924054
OLFML2A	-1.194290467	6.194079534	-9.512903647	3.15E-11	4.20E-10	15.33962555
AGMAT	-2.260609263	6.295842102	-9.508334147	3.19E-11	4.24E-10	15.32731334
PLCG2	-1.621035672	7.33977476	-9.501893646	3.25E-11	4.30E-10	15.30995524
ARL4C	1.656918829	6.17138817	9.49068286	3.35E-11	4.43E-10	15.27972765

BLNK	-1.774591895	6.390572196	-9.486014474	3.39E-11	4.47E-10	15.26713549
GGH	-2.775276793	6.345287236	-9.484469069	3.40E-11	4.49E-10	15.2629664
DYNC2LI1	1.872913272	6.291015643	9.482638137	3.42E-11	4.50E-10	15.25802664
TIE1	-1.730098767	5.108744886	-9.473736286	3.50E-11	4.60E-10	15.23400368
PTPN14	1.061575733	4.430092376	9.464759618	3.59E-11	4.70E-10	15.2097684
MICALL2	1.4301303	5.637075947	9.461786984	3.62E-11	4.73E-10	15.20174055
NMI	1.460364167	6.334181843	9.449874732	3.73E-11	4.86E-10	15.16955901
PXDN	1.400713352	6.678061514	9.43727216	3.86E-11	5.02E-10	15.1354925
PARM1	-1.435189111	6.818342882	-9.416459397	4.08E-11	5.28E-10	15.07918761
XPNPEP2	-3.006171957	6.878210856	-9.415254198	4.10E-11	5.28E-10	15.07592545
HS3ST2	1.236927726	6.037913304	9.413161901	4.12E-11	5.30E-10	15.07026172
PNPLA3	-1.023442653	5.467680357	-9.393808326	4.34E-11	5.56E-10	15.01784576
FGF1	-1.686244788	6.699474449	-9.39295766	4.35E-11	5.56E-10	15.01554076
GTF3C3	1.107553564	5.119606499	9.386016109	4.43E-11	5.65E-10	14.99672814
HLA-G	1.541610154	9.558573923	9.377756087	4.53E-11	5.75E-10	14.97433415
FGF9	-2.467634053	7.168506726	-9.37406207	4.58E-11	5.79E-10	14.96431633
CAPG	2.275454526	7.599957558	9.372922192	4.59E-11	5.81E-10	14.96122474
BCAM	-1.034388679	7.541988902	-9.346581367	4.93E-11	6.20E-10	14.88973597
CYP17A1	-2.562260185	6.688369003	-9.340631182	5.01E-11	6.29E-10	14.87357481
DNAJC1	1.335755502	6.663744094	9.337950713	5.05E-11	6.32E-10	14.86629296
MCM3	1.017436854	7.014432097	9.335028524	5.08E-11	6.37E-10	14.85835339
TMEM158	-1.238564163	4.877072412	-9.324404597	5.23E-11	6.54E-10	14.82947894
OLR1	1.476393443	4.162449193	9.31835671	5.32E-11	6.63E-10	14.81303508
IRF2	1.116363508	6.163741794	9.318356547	5.32E-11	6.63E-10	14.81303463
CRYAB	1.991309242	10.19226798	9.272432432	6.02E-11	7.40E-10	14.68801575
NUS1P3	3.030191836	4.451430721	9.271743477	6.03E-11	7.40E-10	14.68613814
IDH2	-1.624781634	7.615164301	-9.257349613	6.27E-11	7.68E-10	14.64689669
ADAMTS5	-1.567626164	5.565935294	-9.253245326	6.34E-11	7.72E-10	14.63570245
PTGER4	-1.150087757	5.915254856	-9.248080273	6.43E-11	7.83E-10	14.62161194
OLFML1	-1.053882605	4.869774827	-9.244040632	6.50E-11	7.89E-10	14.61058922
TMEM30B	-2.433255087	6.069296571	-9.242430086	6.53E-11	7.92E-10	14.60619404
ANXA4	1.362840892	9.381243662	9.233762054	6.69E-11	8.06E-10	14.58253325
PLAT	-2.714071242	6.880461305	-9.230492402	6.75E-11	8.10E-10	14.5736057
UBE3C	1.077059362	6.869541584	9.224782441	6.85E-11	8.22E-10	14.55801178
RAI2	1.248864884	6.605882649	9.222422152	6.90E-11	8.27E-10	14.5515646
WDR43	1.355505469	5.100528526	9.205917365	7.21E-11	8.60E-10	14.50646147
PBLD	-2.449137768	8.996436488	-9.198053034	7.37E-11	8.75E-10	14.48495809
ACO1	-1.553432294	8.42225505	-9.189997881	7.53E-11	8.93E-10	14.46292474
HSD11B2	-3.284161049	7.475704201	-9.181748525	7.70E-11	9.12E-10	14.44035158
GPR126	1.548753096	6.160240847	9.177705783	7.79E-11	9.21E-10	14.42928602
PKD2	1.466605063	7.708852145	9.175661971	7.83E-11	9.25E-10	14.42369102
BAMBI	1.778488111	7.243645561	9.168870119	7.98E-11	9.42E-10	14.40509428
ITGB8	2.198762295	4.724448783	9.165691464	8.05E-11	9.48E-10	14.3963888
CMAHP	-1.321818232	5.541320325	-9.154015906	8.31E-11	9.76E-10	14.36440148

SATB2	1.645381501	4.993662224	9.152500678	8.34E-11	9.79E-10	14.36024896
NFIB	1.013507324	6.537514781	9.147938315	8.44E-11	9.89E-10	14.34774392
NMD3	1.586775902	5.557119364	9.133650121	8.78E-11	1.02E-09	14.30856404
PTGER2	1.893335377	4.605453788	9.124061689	9.01E-11	1.05E-09	14.28225685
TMEM106B	2.036280308	6.157463359	9.122305571	9.06E-11	1.05E-09	14.27743743
LIPA	1.633520181	8.304349728	9.11844791	9.15E-11	1.06E-09	14.26684923
MAN1C1	-1.719064907	6.825389124	-9.108859189	9.39E-11	1.08E-09	14.24052266
DAO	-1.616107624	6.835070451	-9.096471107	9.72E-11	1.12E-09	14.20649291
TLN2	-1.17930337	7.167597678	-9.091266133	9.86E-11	1.13E-09	14.19218915
RAMP3	-1.767279559	7.057679392	-9.091163409	9.86E-11	1.13E-09	14.19190682
TRIM38	1.139446433	7.269986447	9.071107086	1.04E-10	1.19E-09	14.13675767
CRHBP	-1.965965894	5.812180364	-9.065867887	1.06E-10	1.20E-09	14.12234296
RIMBP2	-1.062404828	5.197820384	-9.057731086	1.08E-10	1.23E-09	14.0999491
ALB	-1.765046184	4.511772002	-9.046677198	1.11E-10	1.26E-09	14.06951347
ABHD11	1.601534019	7.761978469	9.029489688	1.17E-10	1.31E-09	14.02215885
PSMB10	1.358088211	5.900915938	9.025048978	1.18E-10	1.32E-09	14.00991782
AMMECR1	1.140898086	5.705269915	9.024908105	1.18E-10	1.32E-09	14.00952946
EXOC5	1.647476915	4.573011309	9.016279432	1.21E-10	1.35E-09	13.98573683
DSG2	1.479427993	8.296587149	9.012477241	1.22E-10	1.36E-09	13.97524971
ALDOB	-3.472697226	7.099721919	-9.003998478	1.25E-10	1.39E-09	13.95185719
CLIC5	-2.383308849	6.455372386	-8.993780608	1.29E-10	1.42E-09	13.92365449
SLC26A4	-1.313793891	3.953162749	-8.982850496	1.33E-10	1.45E-09	13.89347131
CD34	-1.060194546	6.889085323	-8.976715293	1.35E-10	1.47E-09	13.87652252
NR2F1	-1.914269919	5.850110374	-8.974393543	1.36E-10	1.48E-09	13.87010734
MALL	1.21701024	6.247590502	8.972007683	1.37E-10	1.49E-09	13.86351431
COCH	-1.168670629	4.575445129	-8.958649356	1.42E-10	1.54E-09	13.82658696
SYNGR2	1.303471407	8.27677576	8.958401074	1.42E-10	1.54E-09	13.82590041
KRT7	1.527211768	5.289057216	8.95242016	1.44E-10	1.56E-09	13.80935948
WIPI1	1.500420586	6.49466384	8.92662217	1.55E-10	1.66E-09	13.73796053
RAP1GAP	-1.211853153	7.039788563	-8.923378213	1.56E-10	1.67E-09	13.72897658
AMZ2	1.02046644	7.498091807	8.922298885	1.57E-10	1.67E-09	13.72598716
SNRPB	1.042357996	7.792878714	8.917902628	1.59E-10	1.69E-09	13.71380928
ANKMY2	1.122856788	7.352101041	8.911352723	1.61E-10	1.72E-09	13.69566117
FZD7	-1.234204862	5.024476936	-8.900739655	1.66E-10	1.76E-09	13.66624367
ACTR3	1.198953014	8.485432462	8.892537967	1.70E-10	1.80E-09	13.6435004
PC	-1.379057949	7.029413143	-8.888352557	1.72E-10	1.81E-09	13.63189102
BIRC3	1.554245699	4.425923994	8.88026306	1.76E-10	1.85E-09	13.60944637
CDH5	-1.537507148	4.841390001	-8.877955014	1.77E-10	1.86E-09	13.6030411
GCDH	-1.091209912	6.427663174	-8.856668582	1.88E-10	1.95E-09	13.54393578
DDB2	1.358389085	6.159106438	8.841819827	1.96E-10	2.02E-09	13.50267225
ENO2	1.43197543	6.034931517	8.831959507	2.01E-10	2.08E-09	13.475256
GBAS	1.015503095	7.65030946	8.829766999	2.02E-10	2.09E-09	13.46915817
ADAMDEC1	1.263358289	3.75856991	8.812306342	2.12E-10	2.17E-09	13.42057503
CAV2	1.414843995	5.498587082	8.795404188	2.22E-10	2.27E-09	13.37350979

LZTFL1	1.071239718	6.415596295	8.771755012	2.37E-10	2.40E-09	13.30759751
C5orf28	1.897312969	4.037172899	8.771036639	2.38E-10	2.40E-09	13.30559425
UBA2	1.178564392	6.519220702	8.770892028	2.38E-10	2.40E-09	13.30519099
SNX10	1.727036787	6.858436969	8.762941115	2.43E-10	2.45E-09	13.2830147
PSMB3	1.092964405	8.388095938	8.761950818	2.44E-10	2.45E-09	13.28025207
TRAM1	1.429322095	7.563336875	8.760724724	2.45E-10	2.45E-09	13.27683146
PPARG	1.167068109	5.369642016	8.749268586	2.53E-10	2.53E-09	13.2448617
PLS1	1.634085949	8.118355328	8.746232097	2.55E-10	2.54E-09	13.23638528
SOCS2	-1.485179854	5.594424992	-8.732959788	2.64E-10	2.62E-09	13.19932203
IRX5	2.311054764	5.320236177	8.727174929	2.69E-10	2.66E-09	13.18316087
ALCAM	1.12267699	6.808048725	8.72510832	2.70E-10	2.67E-09	13.17738638
NPEPPS	1.001644736	6.101949039	8.715869844	2.77E-10	2.73E-09	13.15156595
FAAH	-1.412753809	6.516036569	-8.715034742	2.78E-10	2.73E-09	13.14923143
LBR	1.106278567	7.937242647	8.70570865	2.85E-10	2.79E-09	13.12315451
GSTM3	-2.314536716	8.389138055	-8.701892402	2.88E-10	2.81E-09	13.11248072
VEZF1	1.26521261	7.04110596	8.678038251	3.08E-10	2.99E-09	13.04572173
HSPB8	1.531879675	7.705635015	8.677414859	3.08E-10	2.99E-09	13.04397615
WASL	1.131878175	5.395671248	8.671168705	3.14E-10	3.04E-09	13.02648346
PLOD2	1.646871747	6.774086192	8.66660829	3.18E-10	3.07E-09	13.01370875
RAB11FIP2	1.100008289	6.889366245	8.652023417	3.31E-10	3.18E-09	12.9728363
TUBA1B	1.006869711	10.80776552	8.647579711	3.35E-10	3.21E-09	12.96037815
SLC35F2	1.0153594	6.566384855	8.646957128	3.36E-10	3.21E-09	12.95863251
CYP4F2	-1.942120661	7.030935943	-8.645658313	3.37E-10	3.22E-09	12.95499066
CAPN1	1.299286182	7.100912744	8.64194948	3.40E-10	3.25E-09	12.94459005
HAVCR1	2.688829279	5.011168095	8.628080135	3.54E-10	3.37E-09	12.90568161
GULP1	1.12996644	6.180421661	8.626483296	3.55E-10	3.38E-09	12.90120041
C16orf80	1.053142244	7.327283893	8.622336573	3.60E-10	3.41E-09	12.88956202
AOC3	-2.127375579	5.513490783	-8.621890245	3.60E-10	3.41E-09	12.88830921
CALB1	-5.241278368	7.489473823	-8.615843773	3.66E-10	3.47E-09	12.87133481
FDXR	1.609190088	4.978968274	8.610758103	3.71E-10	3.51E-09	12.85705426
KDELC1	2.060213392	4.746709742	8.603415858	3.79E-10	3.58E-09	12.83643169
FABP5	1.926033654	4.766607352	8.601914356	3.81E-10	3.59E-09	12.83221353
HLA-DMB	1.204039593	8.131677952	8.601682614	3.81E-10	3.59E-09	12.83156247
GALNT7	1.590905277	5.945300972	8.600776739	3.82E-10	3.60E-09	12.82901745
SSPN	1.036640916	4.569998709	8.598549681	3.84E-10	3.62E-09	12.82276018
GIMAP4	-1.408581637	5.980018605	-8.598394826	3.84E-10	3.62E-09	12.82232506
ITGA8	-1.259899939	5.877967814	-8.581322481	4.03E-10	3.77E-09	12.77433731
PON2	1.760162979	7.918124314	8.577249195	4.08E-10	3.81E-09	12.76288271
HIST1H2BE	1.003842901	4.471266918	8.56970777	4.17E-10	3.88E-09	12.74166994
ACSF2	-2.331253101	7.173967977	-8.53299006	4.62E-10	4.25E-09	12.63829099
FAIM	1.529281586	5.00540073	8.526527948	4.70E-10	4.32E-09	12.62008005
CASP1	1.22750791	5.299372462	8.511220673	4.91E-10	4.50E-09	12.57692242
EMX1	-1.12217781	5.604337634	-8.500530083	5.06E-10	4.60E-09	12.54676448
PLAG1	1.094243662	4.494959358	8.491324976	5.19E-10	4.71E-09	12.52078606

TVP23B	1.237446186	5.672321501	8.485696984	5.27E-10	4.77E-09	12.50489789
SAP30BP	1.054837906	5.864815525	8.468041179	5.54E-10	4.99E-09	12.45502987
BACH1	1.46916496	5.366468958	8.463342396	5.61E-10	5.06E-09	12.4417521
SIM2	-1.352375962	5.244279883	-8.462960044	5.62E-10	5.06E-09	12.44067154
SUSD4	-1.028039344	7.184555532	-8.459370197	5.68E-10	5.11E-09	12.43052545
MEIS2	-2.595423641	6.166279275	-8.450828408	5.81E-10	5.22E-09	12.40637736
CD37	1.141236622	4.831004324	8.431832223	6.13E-10	5.50E-09	12.352643
SPAG6	1.221575208	4.798786144	8.430950799	6.15E-10	5.51E-09	12.35014868
FCGR2B	1.400613563	5.322569569	8.413407026	6.46E-10	5.76E-09	12.30048284
SLC34A1	-1.957214348	5.924959713	-8.409315245	6.53E-10	5.81E-09	12.2888939
CD151	1.153279952	7.271755624	8.391670702	6.87E-10	6.08E-09	12.23889747
TMPRSS2	-1.199293706	6.114152878	-8.384321326	7.01E-10	6.19E-09	12.21806191
IL10RB	1.107866465	5.374432552	8.381136989	7.07E-10	6.24E-09	12.2090323
CYP4A11	-2.788818016	7.461924955	-8.36566389	7.39E-10	6.47E-09	12.16513926
ANXA3	1.64148879	5.721322171	8.356644458	7.58E-10	6.63E-09	12.13954056
IFNGR1	1.127464288	7.193444299	8.349191077	7.74E-10	6.74E-09	12.11837937
C8orf33	1.046687356	5.547102952	8.34681511	7.79E-10	6.77E-09	12.1116323
CORO1C	1.15239021	7.661282503	8.34025093	7.94E-10	6.87E-09	12.09298844
KRT8	2.412617712	8.011293867	8.337439881	8.00E-10	6.91E-09	12.08500284
CACNB4	-1.0175558	5.042291718	-8.303137717	8.82E-10	7.53E-09	11.98748319
SKAP2	1.157212074	5.960579879	8.298643447	8.93E-10	7.61E-09	11.97469599
SCEL	3.237409214	4.373548887	8.29142323	9.11E-10	7.76E-09	11.95414795
TESC	1.662947801	6.377100247	8.287615382	9.21E-10	7.83E-09	11.94330874
MEX3C	1.093582146	4.518500361	8.268898762	9.71E-10	8.22E-09	11.89000659
SPTLC1	1.051485989	6.674267861	8.268269055	9.73E-10	8.23E-09	11.88821257
FCN3	-1.457046508	5.896286339	-8.239624475	1.06E-09	8.82E-09	11.80655648
CORO1A	1.116877598	5.223306498	8.23877078	1.06E-09	8.83E-09	11.80412143
UPK1B	1.425870821	6.195143591	8.2360144	1.07E-09	8.89E-09	11.79625865
EPB41L2	1.105039873	6.552896529	8.234518992	1.07E-09	8.91E-09	11.79199252
SLC10A2	-1.35665569	5.932733864	-8.23310394	1.08E-09	8.94E-09	11.7879554
NTRK2	-1.70209379	5.778619948	-8.231793382	1.08E-09	8.97E-09	11.78421619
ANG	1.120944686	6.083285004	8.227413194	1.09E-09	9.06E-09	11.77171745
ADH1C	-1.072916067	4.002784792	-8.223448332	1.11E-09	9.16E-09	11.76040193
EFHD1	-1.493969254	9.225254779	-8.217224959	1.12E-09	9.31E-09	11.74263708
RRN3	1.056234512	5.476587735	8.208492207	1.15E-09	9.52E-09	11.71770164
NOV	1.439198543	5.881302991	8.205300357	1.16E-09	9.60E-09	11.70858547
TNFRSF11B	2.439892235	5.851329232	8.198360848	1.19E-09	9.78E-09	11.68876167
CDKN1C	-1.540596819	6.064117242	-8.190967562	1.21E-09	9.97E-09	11.66763553
AQP3	-2.065181442	7.589596731	-8.178086797	1.26E-09	1.03E-08	11.63081413
NEDD4L	-1.032713657	6.879384573	-8.173559614	1.27E-09	1.04E-08	11.61786808
FCGR3B	1.42070017	5.33138678	8.169022385	1.29E-09	1.05E-08	11.60489096
KHK	-2.077866791	6.965802258	-8.166588657	1.30E-09	1.06E-08	11.59792919
CSGALNACT1	-1.828031759	5.663431696	-8.165419251	1.30E-09	1.06E-08	11.59458382
SLC12A3	-2.130123774	6.647300492	-8.15499878	1.34E-09	1.09E-08	11.56476666

SLC22A6	-3.212565431	6.687322076	-8.145129379	1.38E-09	1.12E-08	11.53651497
CARHSP1	1.043077713	7.741724614	8.144265431	1.38E-09	1.12E-08	11.53404135
CNTN6	1.225020654	3.746051443	8.12383816	1.47E-09	1.18E-08	11.47553019
UGCG	2.325783072	6.626258283	8.118428891	1.49E-09	1.19E-08	11.46002818
KDM3A	1.151409828	6.84095316	8.088662141	1.62E-09	1.29E-08	11.37466305
TGIF1	1.531377432	7.590549385	8.084818817	1.64E-09	1.30E-08	11.36363391
GPD1	-1.814196362	6.80024936	-8.073299146	1.69E-09	1.34E-08	11.33056617
KRT19	2.557871446	7.252412236	8.066692218	1.73E-09	1.36E-08	11.31159399
SERPINB1	1.171978721	6.513174745	8.062647391	1.75E-09	1.38E-08	11.29997663
ABCG2	-1.098549939	5.12619026	-8.045884141	1.83E-09	1.44E-08	11.25181061
SCARA3	1.267727743	6.313090843	8.035887274	1.89E-09	1.48E-08	11.22307166
KIAA1598	1.104330824	6.176573725	8.032341712	1.91E-09	1.49E-08	11.21287623
MEAF6	1.151429949	6.883295479	8.029743546	1.92E-09	1.50E-08	11.2054042
KRT18	1.926438384	9.632214892	8.024330172	1.95E-09	1.52E-08	11.18983356
SOX4	1.153661462	6.418262959	7.991115168	2.14E-09	1.66E-08	11.09422551
PLG	-3.138609518	6.573345011	-7.978800865	2.22E-09	1.71E-08	11.05874849
MAP3K2	1.072494475	5.20928262	7.960279185	2.34E-09	1.79E-08	11.00535703
EZR	1.983118378	7.544449797	7.955785203	2.37E-09	1.82E-08	10.99239683
ERGIC2	1.195166502	6.262360978	7.953828896	2.39E-09	1.82E-08	10.98675435
CASD1	1.024645689	5.468115812	7.949439827	2.42E-09	1.84E-08	10.97409365
ADH6	-1.40205608	5.367840568	-7.946057759	2.44E-09	1.86E-08	10.96433631
NR3C2	-1.214793484	7.034339352	-7.943544206	2.46E-09	1.87E-08	10.95708386
EMP3	1.581517384	7.073379465	7.939898618	2.48E-09	1.89E-08	10.94656388
CLEC7A	1.084129831	4.932368865	7.935713571	2.51E-09	1.91E-08	10.93448542
RIT1	1.319962359	4.767001115	7.92440757	2.60E-09	1.96E-08	10.9018457
SH3BP4	-1.633173022	7.596337421	-7.923638716	2.60E-09	1.97E-08	10.89962556
CLDN5	-1.170457788	6.12463814	-7.92137865	2.62E-09	1.98E-08	10.89309905
SLC7A9	-2.664801583	7.592586408	-7.920896894	2.62E-09	1.98E-08	10.89170779
ABI3BP	2.058723541	4.849293336	7.917403333	2.65E-09	1.99E-08	10.88161798
SLC13A3	-2.601501741	7.724239337	-7.91309395	2.68E-09	2.01E-08	10.86917016
KDR	-1.282615269	5.395486384	-7.912239244	2.69E-09	2.02E-08	10.86670108
CD200	1.517410588	5.916892584	7.896833771	2.81E-09	2.10E-08	10.82218403
NPHS2	-2.941749835	8.210159928	-7.889786118	2.87E-09	2.13E-08	10.80181
POLD2	1.25240743	6.672669111	7.888117214	2.88E-09	2.14E-08	10.79698459
KCTD3	1.292593303	4.451573319	7.882346727	2.93E-09	2.17E-08	10.78029772
SLC16A7	-1.612620879	5.696830262	-7.856546233	3.16E-09	2.33E-08	10.70564537
MAST4	-1.143364254	6.314001668	-7.852562665	3.19E-09	2.35E-08	10.69411282
DDX41	1.033052433	6.956295967	7.840349028	3.31E-09	2.43E-08	10.65874345
GAS1	-1.103511022	5.132902258	-7.836962644	3.34E-09	2.45E-08	10.64893406
KMO	-1.971786045	5.889519026	-7.831373588	3.39E-09	2.49E-08	10.63274149
LYN	1.060774439	5.650032809	7.830881236	3.40E-09	2.49E-08	10.6313149
NUPR1	1.262650205	7.079542855	7.827855931	3.43E-09	2.51E-08	10.62254848
PFKP	1.764804433	7.827600504	7.824872368	3.46E-09	2.53E-08	10.61390207
TOMM70A	1.110797092	7.641258905	7.823365755	3.47E-09	2.54E-08	10.60953552

UGT2A3	2.608300252	5.457876134	7.821201714	3.49E-09	2.55E-08	10.60326317
SEC61G	1.028572745	7.814898043	7.816819417	3.54E-09	2.58E-08	10.59055981
FEM1C	1.018126081	5.648865709	7.812955863	3.58E-09	2.61E-08	10.57935851
SLC7A8	-1.792184281	6.86597887	-7.812654232	3.58E-09	2.61E-08	10.57848395
SELENBP1	-1.237004976	7.156917942	-7.812198974	3.59E-09	2.61E-08	10.57716394
DCAF6	1.029874053	6.335133558	7.78950811	3.83E-09	2.77E-08	10.51134466
CRIP1	2.301546133	6.324722988	7.784759806	3.88E-09	2.80E-08	10.49756446
APOM	-2.01968694	7.716624647	-7.782575801	3.91E-09	2.82E-08	10.49122541
CXCR4	1.717024273	6.940872793	7.777806549	3.96E-09	2.86E-08	10.47738097
CASK	1.140720244	6.378549857	7.763620954	4.13E-09	2.96E-08	10.43618828
MAP3K1	1.063003549	4.018755302	7.743871164	4.37E-09	3.12E-08	10.37880334
CHIT1	1.787294915	4.58986173	7.730846746	4.54E-09	3.23E-08	10.34093761
CTSS	1.194937733	5.142772442	7.723067664	4.64E-09	3.30E-08	10.31831327
ASPN	-1.61031518	5.849509643	-7.721310484	4.66E-09	3.31E-08	10.3132019
HIST1H2BK	1.25420043	6.699176381	7.717992158	4.71E-09	3.33E-08	10.30354854
DPY19L2P2	1.325414372	4.275302215	7.70524864	4.89E-09	3.45E-08	10.26646583
EIF1AY	-1.060211466	3.987776136	-7.702434642	4.93E-09	3.47E-08	10.25827507
PTPRE	1.141790565	6.105261153	7.702138027	4.93E-09	3.47E-08	10.25741166
C3AR1	1.218045693	6.195124637	7.69881452	4.98E-09	3.50E-08	10.24773672
GPC3	-1.815945744	6.168387513	-7.698352927	4.98E-09	3.50E-08	10.24639291
TMX1	1.364100407	6.748831935	7.684446794	5.19E-09	3.63E-08	10.20589844
RELN	2.036380415	6.17699211	7.671969451	5.38E-09	3.75E-08	10.16954791
SPON1	1.946647278	6.100615023	7.665574518	5.48E-09	3.81E-08	10.15091131
CCL8	-1.798976656	6.058077735	-7.661249261	5.55E-09	3.85E-08	10.13830398
AGPS	1.465154412	5.416425049	7.659550487	5.58E-09	3.87E-08	10.13335185
ASAP2	-1.104355694	7.633305771	-7.659247189	5.58E-09	3.87E-08	10.13246767
TNFSF10	1.911134149	8.250642886	7.650200344	5.73E-09	3.96E-08	10.10608987
APOH	-1.61015123	5.580662885	-7.64911668	5.75E-09	3.97E-08	10.10292969
BZW1	1.221695914	7.495730802	7.643664542	5.84E-09	4.03E-08	10.08702839
LAPTM5	1.253922535	7.942619016	7.643014012	5.85E-09	4.03E-08	10.08513091
PLSCR1	1.369235966	7.572974688	7.636006289	5.97E-09	4.11E-08	10.06468788
CDH6	1.665893398	6.095902321	7.629185022	6.09E-09	4.19E-08	10.04478407
TENC1	-1.043327132	6.903217622	-7.618970314	6.28E-09	4.30E-08	10.01496987
ECM2	-1.854072599	6.561095821	-7.609452874	6.45E-09	4.41E-08	9.987181489
ABCC4	1.514027951	7.19684626	7.587604193	6.88E-09	4.68E-08	9.923355315
HSPB2	1.044340708	6.002313922	7.576231278	7.11E-09	4.82E-08	9.890113226
LAMB1	1.224445074	7.762901725	7.55562686	7.55E-09	5.09E-08	9.829855994
TUBA1A	1.453471888	8.365784255	7.534057547	8.04E-09	5.39E-08	9.766732722
SLCO4C1	1.164999606	5.099368213	7.529091932	8.15E-09	5.46E-08	9.752194324
HK1	1.019723588	7.489063645	7.513096569	8.54E-09	5.70E-08	9.705346742
GMNN	-1.308023366	6.072277894	-7.513056733	8.54E-09	5.70E-08	9.705230039
DPYS	-3.352434885	7.056523269	-7.506409851	8.71E-09	5.79E-08	9.685755258
NLGN1	1.770406675	4.10998671	7.504207974	8.77E-09	5.82E-08	9.67930302
SPTBN2	-1.372516632	6.156060295	-7.503384246	8.79E-09	5.83E-08	9.6768891

ACOX2	-2.041512558	6.791761134	-7.499212293	8.90E-09	5.90E-08	9.664662282
RHOBTB3	-1.311876294	5.672334262	-7.4926738	9.07E-09	6.00E-08	9.645496465
GHR	-2.03620667	5.472892931	-7.47661926	9.50E-09	6.25E-08	9.59841971
ATHL1	2.318456023	6.611435482	7.471780545	9.64E-09	6.33E-08	9.584226352
CXCL1	2.116425661	5.350460416	7.447075052	1.04E-08	6.75E-08	9.511723605
CYFIP2	-1.590746382	8.52895289	-7.444019697	1.05E-08	6.80E-08	9.502753136
MSH2	1.062736616	4.689257626	7.440671868	1.06E-08	6.86E-08	9.492922968
ASNS	1.032673172	5.79999469	7.429043484	1.09E-08	7.07E-08	9.458770643
PERP	2.387694106	6.532781662	7.423408771	1.11E-08	7.18E-08	9.442217091
OXR1	2.001236629	4.95101388	7.422930339	1.11E-08	7.19E-08	9.440811425
FOLH1B	-1.60764378	5.105525675	-7.416261069	1.13E-08	7.30E-08	9.421214474
PLEKHA1	1.404820731	7.645098924	7.412683881	1.15E-08	7.36E-08	9.410701586
IL6	-2.032238894	5.11344484	-7.400360436	1.19E-08	7.60E-08	9.374475568
HLA-C	1.127988557	10.68079756	7.396144657	1.20E-08	7.70E-08	9.362079655
ELF3	1.54858441	7.424433507	7.391553122	1.22E-08	7.78E-08	9.348577029
OLFML3	-1.133964377	7.172537046	-7.391540871	1.22E-08	7.78E-08	9.348541
GPX1	1.826109423	8.721096206	7.363869062	1.32E-08	8.41E-08	9.267123974
CXCL6	2.67999219	4.968609201	7.361557975	1.33E-08	8.46E-08	9.260321062
SLC16A5	-1.130059179	5.319285135	-7.34551174	1.40E-08	8.83E-08	9.213074119
LYZ	2.626640923	7.149544192	7.342625708	1.41E-08	8.89E-08	9.204573957
APPL2	1.283795297	5.220925131	7.331666934	1.45E-08	9.16E-08	9.172290542
RCN1	1.121849087	7.379923938	7.313372749	1.53E-08	9.61E-08	9.118373922
CUBN	-2.845578734	7.547911911	-7.297335433	1.61E-08	1.00E-07	9.071084371
CYBA	1.734152096	8.159521677	7.292898035	1.63E-08	1.01E-07	9.05799573
LTBP2	-1.2494626	6.457681747	-7.292672207	1.63E-08	1.01E-07	9.057329578
SFN	1.485225638	7.060455176	7.291218509	1.64E-08	1.02E-07	9.053041315
CST6	2.301994873	4.549357982	7.280598546	1.69E-08	1.05E-07	9.021707884
CPD	1.247791338	6.446832091	7.256808881	1.81E-08	1.12E-07	8.95148258
CCT2	1.183105365	7.656227227	7.253376287	1.83E-08	1.13E-07	8.941345777
LAPTM4B	1.112844304	9.021558121	7.252372283	1.83E-08	1.13E-07	8.938380657
SNAI2	-1.920137023	5.258746113	-7.237307316	1.92E-08	1.18E-07	8.893878987
SLC22A8	-3.16673088	6.989540851	-7.231625233	1.95E-08	1.20E-07	8.877089161
SH3BP5	-1.866637939	7.052931606	-7.221034824	2.01E-08	1.23E-07	8.845788522
PDGFA	1.105880667	5.541425708	7.198093743	2.15E-08	1.31E-07	8.777952123
PPFIBP1	1.018693052	5.68346678	7.171955432	2.33E-08	1.40E-07	8.700607816
NRN1	-1.005200304	4.935781845	-7.167726236	2.35E-08	1.42E-07	8.688088123
CD24	1.113899876	10.32418866	7.164921818	2.37E-08	1.43E-07	8.679785386
MVP	1.204713451	8.005929466	7.161815297	2.40E-08	1.44E-07	8.670587492
VIM	1.7304672	10.46334963	7.161538783	2.40E-08	1.44E-07	8.669768741
TPSAB1	1.418924185	6.08866996	7.128297683	2.65E-08	1.57E-07	8.571297124
M6PR	1.477977727	6.214412597	7.12317817	2.69E-08	1.59E-07	8.556123379
ADNP	1.17935962	7.312911528	7.115041508	2.75E-08	1.63E-07	8.532002748
ARL6IP1	1.071074191	8.628148907	7.114167801	2.76E-08	1.63E-07	8.529412383
DCTN4	1.026378083	5.563115082	7.087541575	2.98E-08	1.76E-07	8.450441714

DRAM1	1.012354925	7.738737823	7.085986421	3.00E-08	1.76E-07	8.445827544
SLC22A7	-1.417828847	5.572286858	-7.056538643	3.27E-08	1.91E-07	8.358419632
RALYL	-1.185624799	6.475132469	-7.055428293	3.28E-08	1.91E-07	8.355122529
UGT8	-1.307895163	5.903255883	-7.050351364	3.33E-08	1.94E-07	8.34004574
CXADR	1.447650595	8.521091447	7.022626065	3.62E-08	2.09E-07	8.257675816
MME	-2.697668028	7.566175623	-7.021327319	3.63E-08	2.09E-07	8.253815892
PCK1	-4.021338115	8.888630895	-7.007900454	3.78E-08	2.17E-07	8.213903219
EPS8	1.193892857	8.700641566	7.005016698	3.81E-08	2.18E-07	8.205329194
CITED2	1.519686867	6.698858843	7.000029462	3.87E-08	2.21E-07	8.190499601
GAS2	-1.128449058	4.520860526	-6.990063866	3.98E-08	2.27E-07	8.160861218
RIN2	1.014230367	8.095672439	6.984101406	4.06E-08	2.31E-07	8.143124903
EDNRB	1.468774353	6.309507935	6.982040305	4.08E-08	2.32E-07	8.136993206
GATAD1	1.161866258	6.580074842	6.961034855	4.34E-08	2.45E-07	8.074484898
ODC1	1.416811162	9.253672729	6.956024339	4.41E-08	2.48E-07	8.059569751
VIL1	-1.005889617	5.186536588	-6.935510899	4.68E-08	2.61E-07	7.998486981
AQP2	-1.789988476	7.721664189	-6.927396372	4.80E-08	2.67E-07	7.974316018
GUCY1B3	1.076223184	5.988654272	6.926473038	4.81E-08	2.68E-07	7.97156536
FAT1	1.101723209	8.605411227	6.922237138	4.87E-08	2.71E-07	7.95894562
LGALS3	1.213623917	9.71655719	6.902597239	5.17E-08	2.85E-07	7.900417112
ALPL	-1.292010372	4.799953615	-6.892553462	5.32E-08	2.93E-07	7.870475342
EPAS1	-1.169083224	7.210047141	-6.880760448	5.51E-08	3.03E-07	7.835309898
CPA3	1.502285719	6.927945851	6.877027792	5.58E-08	3.06E-07	7.824177531
TIMP3	-1.805482299	7.299579276	-6.875410387	5.60E-08	3.07E-07	7.819353441
LHFP	-2.138317848	6.61396707	-6.867412771	5.74E-08	3.14E-07	7.795497031
MGAT4B	1.312089587	7.098411148	6.865380388	5.77E-08	3.15E-07	7.789433854
DLD	1.036038493	8.682761217	6.862280862	5.83E-08	3.18E-07	7.780186542
BDKRB2	-1.487123843	6.190191943	-6.845753545	6.12E-08	3.32E-07	7.73086694
IFI16	1.163198645	6.00581787	6.84403871	6.15E-08	3.33E-07	7.725748599
SCRN1	1.362844326	8.075600951	6.839376914	6.24E-08	3.38E-07	7.711833337
MGP	-2.072295602	9.206778478	-6.838865253	6.25E-08	3.38E-07	7.710305963
TSPO	1.046720687	7.527348616	6.829289459	6.43E-08	3.47E-07	7.681717738
PRKACB	1.157230684	7.260686177	6.819289185	6.62E-08	3.56E-07	7.651855739
SORD	-1.815821042	7.524967204	-6.811220491	6.78E-08	3.64E-07	7.627756857
FLRT3	2.012281838	7.692758629	6.806494067	6.88E-08	3.69E-07	7.613638398
KLK1	-1.861633303	6.601810771	-6.806099432	6.89E-08	3.69E-07	7.612459502
CD14	1.215210266	7.02898871	6.801098657	6.99E-08	3.74E-07	7.597519805
VCAM1	1.913801906	9.201434285	6.739141175	8.41E-08	4.44E-07	7.412290615
GPNMB	2.0231671	8.38761679	6.726079071	8.75E-08	4.60E-07	7.373209296
H2BFS	1.151250447	5.250601778	6.722200545	8.85E-08	4.65E-07	7.361602887
CLEC2B	1.146473025	4.575113551	6.718894995	8.94E-08	4.69E-07	7.351710378
AFM	-2.119456961	5.402933203	-6.687378318	9.82E-08	5.10E-07	7.257357617
AIDA	1.877172101	5.820598688	6.679153604	1.01E-07	5.22E-07	7.232725291
EFHC1	1.202194604	5.909460749	6.672331232	1.03E-07	5.31E-07	7.212289871
PCK2	-2.266323959	7.534206505	-6.658776558	1.07E-07	5.50E-07	7.171680854

TNFRSF12A	1.738480741	8.032661803	6.642490004	1.12E-07	5.74E-07	7.122873434
SYNPO	-1.40891166	7.192355782	-6.630762532	1.16E-07	5.92E-07	7.087719408
HPD	-3.58693374	6.618032059	-6.627358143	1.18E-07	5.96E-07	7.07751305
CXCL12	-1.64288932	8.087548853	-6.625017159	1.18E-07	6.00E-07	7.070494409
PPP2R3A	1.07906345	7.455504762	6.62346392	1.19E-07	6.02E-07	7.065837385
PEMT	1.047939132	6.419183512	6.611259814	1.23E-07	6.22E-07	7.029241637
TPD52	-1.154516468	5.582918233	-6.608262044	1.24E-07	6.27E-07	7.020251156
ABCC3	1.068586713	6.764232082	6.605261051	1.26E-07	6.32E-07	7.011250523
TBL1XR1	1.608420611	5.339639719	6.598911257	1.28E-07	6.42E-07	6.992204508
DDX3Y	-1.863116017	5.025314793	-6.597415514	1.29E-07	6.44E-07	6.987717758
TPBG	1.183558491	7.461179281	6.582881429	1.34E-07	6.72E-07	6.944113957
CA12	-1.665135974	8.58114022	-6.580726981	1.35E-07	6.76E-07	6.937649436
PTGDS	-1.954782988	7.745361365	-6.572183034	1.39E-07	6.92E-07	6.912010533
CYP3A5	1.584436659	6.601847499	6.556211667	1.45E-07	7.22E-07	6.8640731
CLDN4	1.295278957	6.983037591	6.534554509	1.55E-07	7.64E-07	6.799049293
BCL6	1.245696391	6.441972592	6.530343874	1.57E-07	7.73E-07	6.786404494
MSLN	2.216165784	4.203236673	6.526647102	1.59E-07	7.80E-07	6.775302136
DPP4	1.967605591	7.32096147	6.506580266	1.69E-07	8.24E-07	6.715024643
GPR183	1.180876889	4.52612461	6.499041041	1.73E-07	8.41E-07	6.692373041
RNASE6	1.245817858	5.671913544	6.491633792	1.77E-07	8.58E-07	6.670115339
YWHAH	1.078977458	7.416792488	6.490378601	1.77E-07	8.61E-07	6.666343416
SH3BGRL3	1.22824672	7.722516398	6.485233963	1.80E-07	8.73E-07	6.650882714
FECH	-1.04868259	6.540967036	-6.470751812	1.88E-07	9.08E-07	6.607354278
TMED2	1.000214087	7.291791765	6.468646201	1.89E-07	9.13E-07	6.601024728
PTH1R	-2.126025426	8.753095093	-6.453779319	1.98E-07	9.48E-07	6.556328581
TMEM243	1.040515906	7.145501651	6.436200817	2.09E-07	9.93E-07	6.503467504
KITLG	1.278116116	3.888805633	6.434927131	2.09E-07	9.97E-07	6.499636825
FRZB	-1.459564206	5.791510518	-6.428244849	2.14E-07	1.01E-06	6.479538352
TM7SF3	1.152913341	5.350523935	6.411580492	2.25E-07	1.06E-06	6.429408255
SMAGP	1.047505074	6.926672297	6.406672065	2.28E-07	1.07E-06	6.414640395
ADH1B	-1.873198961	5.646724276	-6.404194167	2.30E-07	1.08E-06	6.40718483
SLC39A8	1.078280353	5.060756297	6.402956052	2.31E-07	1.08E-06	6.403459459
SNX7	1.113797461	7.691267373	6.386531107	2.42E-07	1.13E-06	6.354032469
PLK2	2.044800754	5.401482169	6.376559356	2.50E-07	1.16E-06	6.324019565
FAM69A	-1.018179464	5.181649351	-6.352134877	2.69E-07	1.24E-06	6.250490572
ATRAID	1.07310465	8.652068055	6.350045168	2.70E-07	1.25E-06	6.244198527
CD63	1.084098011	10.7749028	6.343686309	2.76E-07	1.27E-06	6.225051199
CAPZA2	1.044660816	7.587167729	6.323710627	2.93E-07	1.34E-06	6.164892238
ABAT	-1.874589074	6.704508385	-6.317449199	2.98E-07	1.37E-06	6.146032306
C3	2.223550803	8.016222936	6.312491697	3.03E-07	1.39E-06	6.131098927
C6	1.762870213	5.712532518	6.310440695	3.05E-07	1.39E-06	6.124920485
SOX9	2.28016065	7.682293769	6.296649971	3.18E-07	1.45E-06	6.083373525
PROS1	1.594624984	7.355079169	6.277494528	3.37E-07	1.52E-06	6.02565381
EFEMP1	-2.349853016	6.441003554	-6.271326472	3.43E-07	1.55E-06	6.0070655

MPP6	1.059311169	4.527477379	6.265834231	3.49E-07	1.57E-06	5.990512831
HSPA13	1.011813522	5.393208748	6.256599067	3.58E-07	1.61E-06	5.962677499
PIGR	1.355454269	6.833694423	6.244634733	3.72E-07	1.66E-06	5.92661238
TUBB6	1.256867967	7.451888253	6.230319718	3.88E-07	1.74E-06	5.883455773
PLA2G16	1.283665614	8.238990941	6.226026831	3.93E-07	1.76E-06	5.870512504
NR1H4	1.241021116	7.138430849	6.223640826	3.96E-07	1.77E-06	5.863318348
RGN	-1.039294059	6.935605565	-6.222308978	3.98E-07	1.77E-06	5.859302563
MYLIP	1.035033123	6.741034671	6.221256915	3.99E-07	1.78E-06	5.856130348
PMP22	1.232804265	7.591309998	6.201494987	4.23E-07	1.88E-06	5.796537811
EML4	1.24696765	6.248887141	6.177029029	4.56E-07	2.00E-06	5.722745734
CR2	-1.049744769	4.76647412	-6.171058093	4.64E-07	2.04E-06	5.704734409
SLC7A7	-2.107007663	8.772383956	-6.170402559	4.65E-07	2.04E-06	5.702756938
ETNPPL	-1.13050541	4.742610612	-6.161048268	4.78E-07	2.09E-06	5.674537827
CLDN10	-1.317827023	8.505858859	-6.1530759	4.90E-07	2.14E-06	5.650485925
WDR91	1.184437256	7.107075321	6.150844226	4.93E-07	2.15E-06	5.643752906
SRPX	-1.878086462	5.090624942	-6.1478907	4.98E-07	2.17E-06	5.634841862
CAPRIN1	1.100486835	7.472204439	6.141254484	5.08E-07	2.21E-06	5.614819105
HLA-DPA1	1.047872414	7.684026652	6.135558624	5.17E-07	2.24E-06	5.597632801
TDG	1.030382807	5.780310342	6.112358675	5.54E-07	2.39E-06	5.5276237
DCN	-1.909727878	8.168926246	-6.109178375	5.60E-07	2.41E-06	5.518025838
TSPAN1	1.438429661	9.1854454	6.101835438	5.72E-07	2.45E-06	5.495864751
KIAA1462	-1.010919812	4.248556334	-6.08282564	6.06E-07	2.59E-06	5.438488198
RAB31	1.094465519	7.315336304	6.078147296	6.15E-07	2.62E-06	5.424366731
ASRGL1	1.317287723	8.730681191	6.075740332	6.19E-07	2.63E-06	5.417101219
ABI1	1.178636101	6.060045316	6.065126098	6.39E-07	2.71E-06	5.385060596
RGCC	-1.718929551	6.464755786	-6.064839021	6.40E-07	2.71E-06	5.384193985
ALDH6A1	-1.535227489	7.564545868	-6.064679814	6.40E-07	2.71E-06	5.38371338
SPHK1	1.001828207	6.486418172	6.057286653	6.55E-07	2.77E-06	5.3613949
RCAN2	-1.128158906	7.408526859	-6.043997634	6.82E-07	2.87E-06	5.321275876
PDGFC	1.061426226	8.483778896	6.024140688	7.24E-07	3.04E-06	5.26132382
ID2	1.103648251	8.07214237	6.018152512	7.37E-07	3.09E-06	5.243243302
KCTD12	1.239102329	8.090552103	6.017024337	7.40E-07	3.10E-06	5.239836874
SAMSN1	1.278259511	3.948540462	6.013464108	7.48E-07	3.12E-06	5.229086966
RNASE4	1.150662274	6.970312943	6.009873828	7.56E-07	3.15E-06	5.218246165
SERPINE2	1.649280551	8.730468969	5.98595268	8.13E-07	3.36E-06	5.146012915
ID4	1.561940829	6.96392125	5.965210853	8.65E-07	3.56E-06	5.083375517
PCP4	-2.065134578	7.481939806	-5.934806055	9.49E-07	3.87E-06	4.991551308
ZNF12	1.117027168	6.140246269	5.934662921	9.49E-07	3.87E-06	4.991119022
LGALS1	1.520047769	8.36738603	5.91574535	1.01E-06	4.08E-06	4.933984207
KLF4	1.220831564	6.853548011	5.880825536	1.12E-06	4.50E-06	4.828516346
MMP2	-1.02577096	7.147630339	-5.87965878	1.12E-06	4.52E-06	4.824992372
GIMAP6	-1.049780425	4.675556275	-5.875812131	1.13E-06	4.56E-06	4.813374268
ADAM9	1.166389131	7.477649482	5.868169952	1.16E-06	4.65E-06	4.790292443
MEIS1	-1.126049363	5.736664744	-5.857882489	1.20E-06	4.79E-06	4.759221066

ANKRD2	-1.413842225	5.881502231	-5.852536435	1.22E-06	4.86E-06	4.743074347
HSPA2	-2.097552537	7.991752074	-5.846205614	1.24E-06	4.94E-06	4.723953401
DNASE1	-1.266081714	5.809236164	-5.805454912	1.40E-06	5.55E-06	4.600877539
ACTR2	1.215924426	7.292065183	5.777397297	1.53E-06	6.00E-06	4.516143027
PTPRK	1.058114321	8.087648142	5.773473429	1.55E-06	6.07E-06	4.504293369
SH3GL2	-1.280038726	5.08826959	-5.770479373	1.56E-06	6.12E-06	4.495251739
QPRT	-1.415712576	6.833742613	-5.764715452	1.59E-06	6.21E-06	4.477845739
FXYD1	-1.06668063	5.880325439	-5.756955385	1.63E-06	6.33E-06	4.45441226
C1QB	1.296191241	6.07912493	5.756083328	1.63E-06	6.34E-06	4.451778902
PKM	1.119347291	7.970776851	5.752985951	1.65E-06	6.40E-06	4.442425794
SLC16A4	1.216042637	7.102437821	5.749239999	1.67E-06	6.46E-06	4.431114338
IL13RA2	-1.088957337	4.470310089	-5.744375517	1.69E-06	6.55E-06	4.416425549
POLR2J	1.079282619	8.155908159	5.720836349	1.82E-06	7.00E-06	4.345350815
C1QA	1.28383226	7.11039246	5.706563651	1.90E-06	7.27E-06	4.302259185
MYLK	-1.818055406	7.909764436	-5.697016686	1.95E-06	7.46E-06	4.273437054
GLUL	1.140673126	7.463626548	5.680487976	2.05E-06	7.81E-06	4.223540709
TKT	1.038355734	7.236705143	5.672374732	2.10E-06	8.00E-06	4.199050469
CLDN7	1.286614005	7.656796618	5.665757232	2.15E-06	8.14E-06	4.179076115
APH1A	1.145368174	7.673194394	5.665544448	2.15E-06	8.14E-06	4.178433858
ECI2	-1.08359485	7.931422504	-5.654344474	2.22E-06	8.39E-06	4.144629698
HSPB7	-1.116076306	6.504814115	-5.650307844	2.25E-06	8.49E-06	4.132446819
EVI2A	1.231010275	5.373959368	5.647742554	2.27E-06	8.54E-06	4.124704742
STC1	-1.193842584	6.576515398	-5.644397176	2.29E-06	8.61E-06	4.114608555
MMP7	1.893348337	9.142514856	5.631075286	2.38E-06	8.92E-06	4.074406172
SERPINA1	1.013503158	7.68942373	5.61232533	2.52E-06	9.40E-06	4.017830044
IFI44L	1.333290195	5.424134651	5.602387901	2.60E-06	9.64E-06	3.987848313
NEK7	1.042820491	7.591041794	5.557044614	2.98E-06	1.10E-05	3.851078974
PFN1	1.328046932	9.619270724	5.556661363	2.99E-06	1.10E-05	3.849923223
TMEM140	1.178872025	6.998519002	5.5392711	3.15E-06	1.15E-05	3.797485043
NUAK1	-1.01423014	6.658420056	-5.507779973	3.47E-06	1.26E-05	3.702552592
PPAP2C	1.349985306	5.004927109	5.493401366	3.62E-06	1.31E-05	3.659218662
SH3YL1	-1.383656834	9.067576769	-5.48230229	3.74E-06	1.35E-05	3.625773722
AZGP1	-2.441274358	6.028372323	-5.44991125	4.13E-06	1.47E-05	3.528196966
KDM5D	-1.511381659	5.840236137	-5.440848807	4.25E-06	1.51E-05	3.500904379
PPDPF	1.006420899	7.023193826	5.439310807	4.27E-06	1.52E-05	3.496272861
CLIC4	1.379305021	8.173084197	5.433997412	4.34E-06	1.54E-05	3.480272939
HLA-DPB1	1.050314006	9.300339603	5.428300891	4.41E-06	1.56E-05	3.463120694
GC	-1.86721992	4.6760781	-5.375784075	5.17E-06	1.81E-05	3.305062341
GBP2	1.086335549	6.660819524	5.370215089	5.26E-06	1.83E-05	3.288309312
APOD	-1.201933767	6.885909525	-5.365011164	5.34E-06	1.86E-05	3.272655899
TRIM22	1.001863801	7.663445739	5.36079113	5.41E-06	1.88E-05	3.25996305
CRISPLD2	-1.579323995	7.82678239	-5.343059229	5.71E-06	1.97E-05	3.206639883
RERGL	-1.041425564	4.349591785	-5.329651684	5.95E-06	2.05E-05	3.166332028
GCH1	-1.753099718	6.386403656	-5.329582389	5.95E-06	2.05E-05	3.16612373

ZDHHC4	1.090052886	7.250611936	5.320072463	6.12E-06	2.10E-05	3.137539536
SULT1C2	-1.275344473	7.607243919	-5.310265136	6.31E-06	2.16E-05	3.108066767
ITGA3	1.184915769	7.111624205	5.299359263	6.52E-06	2.23E-05	3.07529916
FABP3	-1.060950526	5.909351699	-5.294790144	6.61E-06	2.26E-05	3.061572923
MT1H	-1.836571107	11.33616278	-5.274597772	7.03E-06	2.38E-05	3.000927333
SLC38A1	1.084749711	7.934814949	5.273592149	7.05E-06	2.39E-05	2.997907702
BCHE	1.828790611	4.486629363	5.267198023	7.19E-06	2.43E-05	2.978709227
FAM129A	1.087325336	5.749192234	5.255377201	7.45E-06	2.51E-05	2.943223746
TGFBR3	-1.099587593	6.819112836	-5.251505736	7.54E-06	2.53E-05	2.931603745
CDH16	-1.972088551	9.488260253	-5.236829162	7.88E-06	2.64E-05	2.887561576
LAMB3	1.19269037	4.970909972	5.235666721	7.91E-06	2.65E-05	2.884073872
ASS1	-2.58891214	9.783254533	-5.219214393	8.31E-06	2.77E-05	2.834721155
S100A2	-2.30586064	8.005757827	-5.209572229	8.56E-06	2.84E-05	2.805805708
CHGB	-1.201497509	5.672031079	-5.191656001	9.04E-06	2.98E-05	2.752094755
LDHA	1.038661385	10.55451857	5.186813078	9.17E-06	3.01E-05	2.737580099
CTGF	-1.43357276	8.245231359	-5.165344815	9.79E-06	3.20E-05	2.673258436
TPSB2	1.440591922	6.862240582	5.164861001	9.80E-06	3.21E-05	2.671809259
ACSL1	-1.065869519	8.384779833	-5.161507501	9.90E-06	3.24E-05	2.661764947
HLA-B	1.378163576	10.88730957	5.138819861	1.06E-05	3.44E-05	2.593834015
PSENEN	1.016616441	6.355942134	5.128906712	1.09E-05	3.53E-05	2.564164771
SLC4A4	-1.260997787	6.734855142	-5.102121	1.18E-05	3.79E-05	2.484036673
NDNF	-1.384961298	6.508681784	-5.09964856	1.19E-05	3.82E-05	2.47664345
IL32	1.487379348	7.722523905	5.095550995	1.21E-05	3.86E-05	2.464391809
MT1X	-1.883971933	10.13512337	-5.078676262	1.27E-05	4.04E-05	2.413951564
PIK3CA	1.140265686	5.308639604	5.055092403	1.37E-05	4.30E-05	2.343498118
BIN1	1.156152962	7.606710965	5.051451617	1.38E-05	4.34E-05	2.332626141
HPGD	-1.653443625	5.462915835	-5.039390685	1.43E-05	4.49E-05	2.296618732
SLC44A4	1.469036674	6.909271149	5.003377428	1.60E-05	4.96E-05	2.189182203
A1CF	-1.207627832	7.013618155	-4.989882305	1.66E-05	5.15E-05	2.148954484
ZNF148	1.096862087	6.921612976	4.984895803	1.69E-05	5.22E-05	2.13409465
CYP26B1	-1.063175491	5.40601742	-4.957838505	1.83E-05	5.62E-05	2.053506531
BHMT	-2.257017482	9.701916254	-4.956964355	1.84E-05	5.63E-05	2.050904166
LSM7	1.095513784	6.171521233	4.951462216	1.87E-05	5.72E-05	2.034525975
ZGPAT	-1.043928651	6.4922239	-4.947165309	1.89E-05	5.79E-05	2.02173755
PDLIM2	-1.174922063	5.963962337	-4.944684657	1.91E-05	5.82E-05	2.014355518
VLDLR	1.02169993	5.823849298	4.90514473	2.15E-05	6.50E-05	1.896778245
DDC	-1.602978501	7.276956051	-4.898435546	2.19E-05	6.61E-05	1.876844255
CFD	1.197369296	5.877283457	4.893617087	2.22E-05	6.70E-05	1.862530937
MSRA	-1.166910533	8.659273292	-4.888933239	2.25E-05	6.79E-05	1.848619944
CNN1	-1.706833681	5.902460304	-4.850383186	2.53E-05	7.55E-05	1.734220551
ALDH1A1	1.275778265	8.859305854	4.846556203	2.56E-05	7.63E-05	1.722873094
FBP1	-1.517044379	8.430406499	-4.839257239	2.62E-05	7.78E-05	1.701235562
HILPDA	1.223522233	6.961569869	4.837203098	2.63E-05	7.82E-05	1.695147264
ABCA8	-1.686132314	6.316427451	-4.820783535	2.77E-05	8.17E-05	1.646499152

PRSS23	-1.050221231	7.405153955	-4.788881444	3.05E-05	8.92E-05	1.552072892
PLS3	-1.077481609	8.699852139	-4.750727418	3.41E-05	9.88E-05	1.439309721
EHD3	-1.137478575	6.646565097	-4.739236115	3.53E-05	0.000101757	1.405384492
CSRP2	-1.098689584	6.634907238	-4.735333435	3.58E-05	0.000102811	1.393866771
STK39	1.123474635	5.610165893	4.728416424	3.65E-05	0.000104746	1.373458046
PRUNE2	1.529178846	6.406834428	4.724673558	3.69E-05	0.000105757	1.362417351
LPL	-1.434526536	6.448586252	-4.719480162	3.75E-05	0.000107316	1.347101021
RGS5	-1.166037147	7.873186055	-4.703902968	3.93E-05	0.000111926	1.301182886
HLA-DRA	1.445651559	9.303332537	4.700444669	3.97E-05	0.000112962	1.290993106
PNMAL1	1.083809621	6.381926881	4.692177978	4.07E-05	0.000115422	1.266642258
ADAMTS3	1.001182563	4.087295568	4.686444478	4.14E-05	0.000117259	1.249758932
LACTB2	1.123461924	6.823246776	4.65003675	4.62E-05	0.000129508	1.142658865
MS4A4A	1.196215653	4.493109565	4.645486084	4.68E-05	0.000130986	1.12928571
AKR1B1	1.050361395	9.402560548	4.628302355	4.93E-05	0.000137176	1.078815015
VAMP3	1.059556122	7.592254957	4.613058518	5.15E-05	0.00014305	1.034078955
DIXDC1	1.031999579	6.61053738	4.605647568	5.27E-05	0.000145859	1.012342772
MT2A	-1.453546842	11.3577051	-4.594404551	5.45E-05	0.000150199	0.979383276
PTPRO	-1.106617492	8.776583727	-4.578478925	5.72E-05	0.000156878	0.932730014
HIBCH	-1.119119815	7.180090656	-4.56655666	5.92E-05	0.00016166	0.897830466
NEFL	1.432983683	4.706703193	4.56431839	5.96E-05	0.000162599	0.891280986
GALNT11	1.293064007	9.331260759	4.538858223	6.43E-05	0.000174373	0.816837815
ALDOA	1.147744388	10.70675368	4.533036351	6.54E-05	0.000177266	0.799830005
FBLN5	-1.128506543	8.107003023	-4.514405235	6.92E-05	0.000186312	0.74543937
GDE1	1.283994388	6.526828188	4.510498636	7.00E-05	0.000188324	0.734042006
MT1F	-1.602175515	10.01917321	-4.502369234	7.17E-05	0.000192432	0.710333007
TOB1	1.008421195	8.468718492	4.501603954	7.18E-05	0.000192807	0.70810168
PROM1	1.607455596	8.551271188	4.475757117	7.76E-05	0.00020692	0.632798912
BHMT2	-1.244652682	9.17082889	-4.421020504	9.12E-05	0.000239729	0.473714985
IL12RB2	1.660209917	4.453519516	4.404673968	9.58E-05	0.000250767	0.426311235
CAPN3	-1.247135138	6.400273494	-4.373540127	0.00010501	0.000272539	0.336163255
WFDC2	1.179656963	8.68877045	4.344458292	0.00011442	0.000295128	0.252123977
HSPB1	1.542969424	9.623694379	4.344365046	0.00011446	0.000295148	0.251854781
CXCL8	1.226316629	5.559996878	4.333174884	0.0001183	0.000304107	0.219562121
CPE	1.280864622	6.03767958	4.332738346	0.00011845	0.000304435	0.218302857
P4HB	1.4854414	8.597277818	4.326622529	0.0001206	0.000309399	0.20066477
RARRES1	1.27446301	5.262670559	4.322035269	0.00012225	0.000313353	0.187439945
DPEP1	-1.973329547	7.833364418	-4.319211468	0.00012327	0.000315537	0.179301165
MRC1	-1.365281542	6.433405228	-4.311921855	0.00012594	0.000321505	0.158298386
S100A9	1.104575014	5.194626465	4.2939555	0.00013279	0.000337102	0.106579738
AMIGO2	-1.099567084	5.416963796	-4.288682567	0.00013486	0.000341559	0.091413365
ENO1	1.013825835	8.638543007	4.284865823	0.00013638	0.000345111	0.080438951
ATP8B1	-1.071072681	6.943270321	-4.280741905	0.00013805	0.000349039	0.068584685
AP2M1	1.265451086	8.46754628	4.27971861	0.00013847	0.00035002	0.065643752
MYH11	-1.02634952	6.053714558	-4.272164534	0.00014158	0.000357154	0.043940196

ALDH4A1	-1.447257873	7.578238583	-4.256397126	0.00014829	0.000372428	-0.001322588
SPARCL1	-1.082816659	8.466828549	-4.244842969	0.00015341	0.000383477	-0.03445726
MT1HL1	-1.337421451	11.09213798	-4.222900706	0.0001636	0.000406542	-0.097303986
TAGLN	-2.086672913	8.343455853	-4.19030003	0.00018	0.000443024	-0.19048459
PLN	-1.24098552	4.819905478	-4.143272326	0.00020651	0.000502911	-0.324481509
F13A1	-1.182799842	6.898504683	-4.134624787	0.00021179	0.000514557	-0.349065859
CXCL14	-1.007403547	8.728139588	-4.133107536	0.00021273	0.000516731	-0.353377503
ID1	1.755725798	8.364248713	4.070019965	0.0002556	0.000611324	-0.532172302
KLF6	1.01486513	7.476670941	4.003347149	0.00031009	0.00072929	-0.72006485
IGFBP2	-1.401618076	7.16750657	-3.978469626	0.0003332	0.000778629	-0.789881859
YWHAE	1.230284866	8.148793296	3.947910494	0.00036389	0.000845743	-0.875420757
ADIRF	1.559805272	8.266013569	3.942474	0.00036963	0.000857325	-0.890612031
PSAT1	-1.016748657	6.141672006	-3.938865352	0.00037349	0.000865794	-0.900691324
EGR2	-1.069941251	5.61325298	-3.938152573	0.00037426	0.000867087	-0.902681767
CYP24A1	1.642297386	5.273302988	3.909976115	0.00040584	0.000932342	-0.981253761
NIT2	1.00191135	8.434523456	3.897483687	0.00042066	0.000962574	-1.016019777
LIPC	1.190362018	6.44917736	3.876221962	0.0004471	0.001015047	-1.075090127
REN	-1.357537168	6.541919885	-3.86981594	0.00045538	0.001032335	-1.09286263
PCOLCE2	-1.407694649	4.650370928	-3.861874704	0.00046585	0.001052999	-1.114878131
NAMPT	1.174047281	6.946850318	3.848310644	0.00048428	0.001091875	-1.152440003
LTBP3	1.438783319	6.628306327	3.844758615	0.00048922	0.00110102	-1.162267595
ASPA	-1.08588018	7.141300202	-3.834766468	0.00050339	0.001130445	-1.18989378
UCHL1	-1.206211301	7.945125887	-3.814119728	0.00053394	0.001192578	-1.246885208
SPINK1	-1.8624585	6.019539444	-3.773498454	0.00059938	0.001323992	-1.358642734
ADI1	-1.029868238	8.931994989	-3.705998012	0.00072569	0.001577234	-1.54323363
CA2	-1.045351647	8.972995465	-3.699903973	0.00073829	0.001601253	-1.559828364
CST3	1.232554464	8.908567512	3.571133115	0.00105969	0.002227983	-1.907635416
NOX4	-1.65469858	7.607457258	-3.563690726	0.00108191	0.002270093	-1.927565612
SLC27A2	-1.320976054	7.471143693	-3.561122124	0.00108968	0.002284089	-1.934439668
BSG	1.25637349	8.109234448	3.549696961	0.00112491	0.002351591	-1.964987554
NQO2	-1.199343513	8.386808748	-3.483745217	0.00135075	0.002775161	-2.140417326
FTCD	-1.120680431	7.311609429	-3.436607538	0.00153825	0.003126299	-2.26482938
TMEM176B	1.361215161	9.503141746	3.3995973	0.00170275	0.003431997	-2.361925994
REG1A	1.039816349	5.672648515	3.396512646	0.0017172	0.003459433	-2.36999494
TXNIP	1.015221009	10.20623818	3.279435659	0.00236114	0.004616817	-2.673473002
DCXR	-1.096892381	8.501142534	-3.213510698	0.00281931	0.005415592	-2.841891881
FMO1	-1.385933441	8.504625525	-3.111227542	0.0037013	0.006939272	-3.099486222
IL17RB	-1.124156742	8.053844349	-3.09659841	0.00384705	0.007177935	-3.135947802
ENPEP	-1.26590341	6.792915316	-3.058325562	0.00425454	0.007854537	-3.230875235
PPIB	1.001365345	8.409420354	2.95543475	0.00556172	0.009990366	-3.482656359
HBB	-1.401658472	9.385071337	-2.908571087	0.00627498	0.011122117	-3.595630461
SLPI	1.402040456	8.122099851	2.877705499	0.00679074	0.011956158	-3.669437608
COX7A1	-1.130240223	7.181698752	-2.872377938	0.00688369	0.012102668	-3.682128192
BBOX1	-1.398802899	8.827055055	-2.782193817	0.00864943	0.014885064	-3.894722259

SLC13A1	-1.30904256	6.152762802	-2.74746632	0.0094357	0.016128782	-3.975440028
FOSB	-1.259859951	7.229522278	-2.715214461	0.010225	0.017332584	-4.049818531
TNC	1.082721654	6.005532385	2.707112629	0.01043269	0.017658162	-4.068413215
LUM	-1.021008176	7.347418497	-2.649259053	0.01203339	0.020096594	-4.200132997
DDIT4	1.044556029	7.70744886	2.624810507	0.01277578	0.021236595	-4.255229702
GPX3	-1.509829728	11.18579778	-2.598989604	0.01360558	0.022465736	-4.313047323
SOSTDC1	1.223586646	6.665112638	2.560172507	0.01494681	0.024433757	-4.399237634
PDK4	-1.008444888	5.92706561	-2.553231939	0.01519906	0.024803721	-4.414555501
C8orf4	-1.180447501	6.214109897	-2.49876513	0.01731847	0.0279071	-4.533769169

Table S2. DEGs of KIRP in GSE15641

	logFC	AveExpr	t	P.Value	adj.P.Val	B
CLDN8	-6.008183342	4.351895987	-45.30728228	5.42E-25	1.11E-20	45.22784017
TMEM52B	-6.056993115	4.373454423	-41.76206865	3.83E-24	3.92E-20	43.68318044
GRHL2	-2.988212878	3.923027071	-39.34680321	1.60E-23	1.09E-19	42.51945472
CA10	-2.859189512	3.940282827	-36.31182848	1.09E-22	5.57E-19	40.91014706
CYP4X1	-2.978254091	3.694292516	-34.91171309	2.78E-22	9.44E-19	40.10611171
EMCN	-4.981072565	5.496129972	-34.73352048	3.14E-22	9.44E-19	40.00077002
ACPP	-2.546053493	3.836987669	-34.69507346	3.23E-22	9.44E-19	39.97794969
MPPED2	-3.609725359	3.918488235	-33.89636684	5.62E-22	1.44E-18	39.4963824
CALB1	-7.358439944	4.518851877	-31.46115195	3.31E-21	7.53E-18	37.93452594
CRHBP	-3.670340687	4.218762875	-31.17048792	4.12E-21	8.45E-18	37.73807052
FLJ35700	-3.371958144	4.189800891	-29.90854995	1.10E-20	2.05E-17	36.85842736
IRX1	-4.618656568	4.156981494	-28.73844418	2.82E-20	4.82E-17	36.0018907
TFAP2B	-2.988425869	3.737745306	-27.46914922	8.20E-20	1.28E-16	35.02485637
TSPAN7	-3.894168093	5.280928775	-27.3350689	9.21E-20	1.28E-16	34.91858308
ELF5	-3.312372252	4.189665815	-27.31315412	9.38E-20	1.28E-16	34.90115588
FABP4	-2.458463565	3.91366623	-26.83754145	1.42E-19	1.82E-16	34.51891367
NPHS2	-5.666400165	6.156338197	-26.70949552	1.59E-19	1.91E-16	34.4146738
PDZD2	-3.039219777	3.968260949	-26.37476849	2.14E-19	2.43E-16	34.13945678
TMEM207	-3.05299008	4.342129178	-26.03324945	2.90E-19	3.01E-16	33.85452667
TCEAL2	-4.238854465	4.11150325	-25.98982605	3.02E-19	3.01E-16	33.81799495
MYCT1	-2.262866607	4.024301954	-25.96643754	3.08E-19	3.01E-16	33.79828978
RERGL	-3.091927385	3.647462254	-25.70434828	3.91E-19	3.64E-16	33.5760954
RALYL	-4.175667949	4.345786144	-24.91436409	8.13E-19	7.24E-16	32.89066123
SLC26A7	-2.267869456	3.420141911	-24.84422201	8.69E-19	7.42E-16	32.82863374
FAM3B	-3.989151898	4.098106043	-23.67015129	2.69E-18	2.09E-15	31.76077139
SOST	-5.281028675	4.151419896	-23.65790595	2.73E-18	2.09E-15	31.74933067
CLDN19	-2.614649464	5.914231726	-23.64791611	2.75E-18	2.09E-15	31.73999247
KCNJ1	-6.38694518	4.96216544	-22.98140718	5.36E-18	3.92E-15	31.10713291
CA4	-3.231039169	4.679578752	-22.7729525	6.63E-18	4.68E-15	30.90514285
SEMA3G	-3.009063682	5.259304094	-22.71178044	7.05E-18	4.82E-15	30.84549254
GPC5	-4.281837461	3.86255615	-21.8836184	1.67E-17	1.07E-14	30.02072177
JAM2	-2.165659515	4.462991289	-21.88244968	1.67E-17	1.07E-14	30.01953469
NDNF	-3.904659764	4.672620538	-21.64771094	2.15E-17	1.33E-14	29.7797515
AVPR1A	-1.423160238	3.768588157	-21.47680475	2.58E-17	1.55E-14	29.60345639
SPINK1	-5.030182172	4.33962939	-21.39957782	2.80E-17	1.64E-14	29.52331429
CLIC5	-2.797864264	4.920253634	-20.8782426	4.96E-17	2.82E-14	28.9743293
RNF150	-2.931238446	4.824056299	-20.58638264	6.86E-17	3.80E-14	28.66079011
GJA5	-1.994885698	3.927808143	-20.04454597	1.27E-16	6.82E-14	28.06649035
FOLH1B	-2.900499189	3.749231355	-18.86085484	5.10E-16	2.68E-13	26.70996123
SCNN1G	-2.505635585	4.390412675	-18.75865128	5.77E-16	2.96E-13	26.58890279
CNKSR1	-1.832857931	4.533423635	-18.52962034	7.64E-16	3.81E-13	26.31525642
ADH1C	-4.444573207	4.099044381	-18.50805811	7.84E-16	3.82E-13	26.28932392

SGIP1	-2.362351695	3.875822138	-18.3502527	9.53E-16	4.54E-13	26.09863423
TEK	-2.123601781	5.274941028	-18.24809198	1.08E-15	5.03E-13	25.9743337
VAT1L	-3.232758481	4.979961453	-18.0260924	1.43E-15	6.50E-13	25.70188665
ZNF385D	-1.667753078	3.830432684	-17.79758151	1.91E-15	8.49E-13	25.41805459
PTGER3	-3.204149139	4.039886633	-17.77778337	1.95E-15	8.49E-13	25.39329923
RASL11B	-1.938234998	4.908414325	-17.76355905	1.99E-15	8.49E-13	25.37549703
MYZAP	-1.828354953	4.829058853	-17.60109481	2.45E-15	1.02E-12	25.17119668
HMGCS2	-2.862282495	4.662451502	-17.3083109	3.58E-15	1.47E-12	24.7984495
KDR	-3.746222696	5.392130191	-17.27077648	3.76E-15	1.50E-12	24.75023307
SYT6	-2.007565475	3.96108839	-17.25927004	3.81E-15	1.50E-12	24.73543218
SLC12A1	-4.972666847	4.667241221	-17.17366473	4.27E-15	1.65E-12	24.62502414
FGF1	-2.852723319	4.729658327	-17.11917984	4.58E-15	1.74E-12	24.55448319
FGF9	-3.775097456	4.595035147	-17.07321342	4.87E-15	1.81E-12	24.49480682
FAM83B	-2.010170307	3.439447583	-16.74249459	7.56E-15	2.76E-12	24.06096379
TMEM213	-4.475295653	4.988633282	-16.64833189	8.58E-15	3.08E-12	23.93598002
KIF26A	-2.524476319	4.42785908	-16.50666124	1.04E-14	3.67E-12	23.74669957
SCNN1B	-3.406509561	4.234586702	-16.34185456	1.30E-14	4.51E-12	23.52461399
CNTN1	-1.94712602	3.640137499	-16.2949244	1.39E-14	4.73E-12	23.46099676
ANGPT1	-2.221585419	3.91233078	-16.06637927	1.90E-14	6.38E-12	23.14877202
PIK3C2G	-2.257723905	3.661307943	-16.04694545	1.95E-14	6.45E-12	23.12203623
CEL	-3.495155384	4.171014728	-16.02975449	2.00E-14	6.50E-12	23.0983615
PPP2R2B	-1.769922856	4.067263695	-15.96738677	2.18E-14	6.98E-12	23.012277
LCN12	-1.8785866	4.121154576	-15.77474598	2.86E-14	9.00E-12	22.74444425
TIE1	-1.745115091	3.826890877	-15.60476891	3.63E-14	1.13E-11	22.5056623
PTPRB	-1.944654325	4.234633419	-15.55522914	3.90E-14	1.19E-11	22.43563028
SLC9A3R2	-1.628654141	3.531978547	-15.51355383	4.14E-14	1.25E-11	22.37656123
COL4A6	-1.338106845	3.928227659	-15.43930506	4.60E-14	1.37E-11	22.27097189
DAAM2	-2.500224959	5.348929413	-15.3828753	4.99E-14	1.46E-11	22.19042004
TCF21	-4.593855459	5.199018972	-15.26727604	5.89E-14	1.70E-11	22.02458248
BMP5	-1.189065654	3.507902845	-15.24737356	6.07E-14	1.72E-11	21.99591836
FAM162B	-2.715583676	4.376739749	-15.24145149	6.12E-14	1.72E-11	21.98738284
ZNF582-AS1	-1.67046056	4.653390204	-15.17973209	6.69E-14	1.85E-11	21.89825128
ANXA2P3	1.505110824	6.593602878	15.11431471	7.36E-14	2.01E-11	21.80342953
PRDM16	-1.677557239	4.222867745	-15.07332052	7.81E-14	2.11E-11	21.74382455
APLNR	-3.531622883	5.203051852	-15.06050164	7.96E-14	2.12E-11	21.72515683
KNG1	-5.504174456	4.484551443	-15.01376185	8.53E-14	2.22E-11	21.65697274
LYPD6B	-2.33493108	4.547743602	-15.01180481	8.55E-14	2.22E-11	21.65411375
C14orf37	-1.865179012	4.939415688	-14.99796083	8.73E-14	2.23E-11	21.63388005
FAM184A	-1.377197883	4.010953039	-14.98524045	8.89E-14	2.25E-11	21.61527414
ECSCR	-2.322911667	5.029076549	-14.81465943	1.14E-13	2.86E-11	21.36442387
PCDH9	-2.783751314	3.823589497	-14.66299219	1.43E-13	3.51E-11	21.13926644
CLDN5	-2.487662836	5.872250982	-14.65990136	1.44E-13	3.51E-11	21.13465699
AMPH	-3.111403328	4.541940135	-14.64651643	1.47E-13	3.52E-11	21.11468594
LOC100129447	-1.13202975	3.726519217	-14.64204014	1.48E-13	3.52E-11	21.10800354

CPAMD8	-2.039603749	5.57416627	-14.4936457	1.85E-13	4.35E-11	20.88546974
CHRM3	-1.604026099	3.877886096	-14.46492971	1.93E-13	4.49E-11	20.84218057
MYOCD	-2.791362066	3.970593056	-14.41554688	2.08E-13	4.79E-11	20.76756346
MILR1	1.751161964	4.75031724	14.29736935	2.49E-13	5.67E-11	20.58810566
KGFLP2	-1.72209368	3.998432205	-14.09931815	3.38E-13	7.60E-11	20.28450319
CLUL1	-1.608842177	3.892610392	-14.06700783	3.55E-13	7.90E-11	20.23463057
TMEM178A	-4.838865786	4.665094333	-13.96271743	4.17E-13	9.19E-11	20.07299093
ELTD1	-2.990282691	4.597493857	-13.80804316	5.31E-13	1.16E-10	19.8313838
SPTSSB	-1.401017073	3.319575213	-13.78809014	5.48E-13	1.18E-10	19.80005175
ITGA8	-1.776150511	4.962878953	-13.75286538	5.79E-13	1.23E-10	19.74464622
OXGR1	-1.050225782	3.510518827	-13.75081942	5.81E-13	1.23E-10	19.74142446
FLRT1	-2.016728975	4.837882032	-13.73424883	5.96E-13	1.25E-10	19.7153162
THSD7B	-1.507897912	3.386320432	-13.71409273	6.16E-13	1.27E-10	19.68352334
DUSP9	-2.906042206	5.141827797	-13.70919541	6.20E-13	1.27E-10	19.67579278
TMEM45B	-2.899083826	5.010547212	-13.67403613	6.56E-13	1.33E-10	19.62022534
SOX18	-1.633665769	3.57204203	-13.66123218	6.69E-13	1.34E-10	19.59995989
CALCA	-1.187547581	4.516580574	-13.61081518	7.25E-13	1.44E-10	19.52000905
PACRG	-1.441544544	5.020756379	-13.60309936	7.34E-13	1.45E-10	19.50775175
SLC4A1	-2.085413829	5.375554974	-13.57263554	7.70E-13	1.50E-10	19.45930087
ERG	-1.334262613	4.150933966	-13.56094359	7.84E-13	1.52E-10	19.44068168
SHISA6	-1.142510197	3.306322338	-13.50486242	8.58E-13	1.64E-10	19.35118899
CD34	-2.065863895	6.240541747	-13.45664323	9.27E-13	1.76E-10	19.27399723
PLA2R1	-1.435120039	3.862208971	-13.44620661	9.42E-13	1.77E-10	19.25725984
LDB2	-2.209945866	4.519468433	-13.36317636	1.08E-12	2.00E-10	19.12372184
TFAP2A	-2.315759284	4.087126919	-13.34681289	1.11E-12	2.04E-10	19.09732432
PCDH12	-1.774984192	5.911775439	-13.32666499	1.14E-12	2.09E-10	19.0647854
SHISA3	-4.643702566	5.692650258	-13.31931579	1.16E-12	2.09E-10	19.05290643
KLHL3	-1.531956864	4.608000501	-13.30838159	1.18E-12	2.11E-10	19.03522289
TMPRSS4	-1.999749311	5.155610182	-13.26822826	1.25E-12	2.23E-10	18.9701824
AGTR1	-3.443329373	4.776030271	-13.24638293	1.30E-12	2.30E-10	18.93472996
FXYD1	-2.113415232	5.440283944	-13.23100314	1.33E-12	2.33E-10	18.9097418
APELA	-1.867088938	3.361773474	-13.15216431	1.51E-12	2.63E-10	18.78127787
FCN3	-2.971904054	4.530167272	-13.11433351	1.61E-12	2.77E-10	18.71941296
LOC285556	-2.627096104	3.507800483	-13.10680837	1.63E-12	2.78E-10	18.70708987
PAPPA2	-1.284112177	4.98430424	-13.08399765	1.69E-12	2.87E-10	18.66970035
ATP6V0A4	-4.339007779	5.292100356	-13.05928182	1.76E-12	2.96E-10	18.62912876
ST6GALNAC3	-4.236567191	4.74640636	-12.92221757	2.21E-12	3.68E-10	18.4030079
LOC100507530	-1.409638426	4.875363837	-12.91461248	2.24E-12	3.69E-10	18.39040527
TSPAN2	-2.064211824	3.602869926	-12.87107206	2.40E-12	3.94E-10	18.31813875
IGF2-AS	-1.501896943	5.226521991	-12.75018344	2.94E-12	4.78E-10	18.11646747
OR5V1	-1.346538552	3.611643837	-12.73065668	3.04E-12	4.90E-10	18.08374993
STON1	-2.136038202	4.613024487	-12.65963659	3.42E-12	5.47E-10	17.96441855
SSBP3	-1.692389641	5.838520711	-12.65041969	3.47E-12	5.51E-10	17.94889317
ITPR1	-2.129805181	5.447503877	-12.57845972	3.92E-12	6.17E-10	17.82737339

C1QTNF7	-1.912082386	3.85061648	-12.57189417	3.96E-12	6.19E-10	17.81625889
TMTC1	-2.557062194	4.868245648	-12.56300337	4.02E-12	6.24E-10	17.80120084
MYOM1	-1.250822878	4.36897309	-12.55256811	4.09E-12	6.30E-10	17.78351631
BMPR1B	-3.091603029	4.405847478	-12.53289319	4.23E-12	6.47E-10	17.7501421
UMOD	-7.431174377	5.924577946	-12.52550617	4.28E-12	6.50E-10	17.73760104
CLDN14	-1.763838094	4.744713601	-12.50862621	4.41E-12	6.64E-10	17.70892195
ATP1B2	-1.711027955	4.980009088	-12.47331044	4.68E-12	7.00E-10	17.64882242
TBX3	-1.304626919	4.408514862	-12.44944969	4.87E-12	7.23E-10	17.6081416
OSBPL6	-1.187367416	3.996977263	-12.42775551	5.06E-12	7.42E-10	17.57110189
S1PR1	-2.534265706	5.090188529	-12.42568294	5.07E-12	7.42E-10	17.56756064
ATP1A2	-1.091035727	3.744780436	-12.4102329	5.21E-12	7.57E-10	17.54114779
KCNJ10	-2.611794571	4.163194268	-12.3995663	5.30E-12	7.65E-10	17.52289763
ABCG2	-1.854764869	4.648721588	-12.38752261	5.41E-12	7.75E-10	17.50227667
KCNE2	-1.547688446	4.946559947	-12.34551602	5.82E-12	8.27E-10	17.43023193
GPC3	-2.678930182	4.497718891	-12.28912681	6.40E-12	8.99E-10	17.33322098
PROX1	-1.89285925	4.724623212	-12.28506319	6.45E-12	8.99E-10	17.32621672
DLL1	-1.235157904	4.668219171	-12.28463951	6.45E-12	8.99E-10	17.32548635
SOX17	-2.124827685	4.140143418	-12.24999483	6.85E-12	9.48E-10	17.26569696
HOXD10	-3.295051425	4.772552416	-12.19738969	7.50E-12	1.03E-09	17.1746624
CALML3	-1.356747113	4.480690134	-12.12582787	8.48E-12	1.14E-09	17.05033821
CDH5	-2.88441608	5.242723042	-12.10817393	8.75E-12	1.17E-09	17.01958183
CRABP1	-1.441518066	4.107817532	-12.0216275	1.02E-11	1.35E-09	16.86830602
GATA3	-4.438200082	4.910521299	-12.00608036	1.04E-11	1.38E-09	16.84104333
SLC16A10	-2.784149603	4.351606782	-11.97420459	1.10E-11	1.45E-09	16.78506381
TMPRSS2	-1.555480839	5.161873295	-11.944672	1.16E-11	1.52E-09	16.73309865
TBX2	-1.886382738	5.226166358	-11.93730593	1.18E-11	1.53E-09	16.72012228
PDE3A	-1.671666167	4.608009269	-11.93164212	1.19E-11	1.53E-09	16.71014059
CLDN16	-2.014830643	4.788714754	-11.89208907	1.28E-11	1.63E-09	16.64033393
BVES	-1.059977275	3.736782889	-11.88684125	1.29E-11	1.64E-09	16.631059
MRGPRF	-2.854020812	5.465995846	-11.88178403	1.30E-11	1.64E-09	16.62211801
PVALB	-3.929317648	4.562898275	-11.83993543	1.40E-11	1.76E-09	16.54802124
TMEM72	-4.056210112	5.901999581	-11.82637581	1.43E-11	1.79E-09	16.52397054
LRRN2	-1.290736918	3.932676243	-11.80496579	1.49E-11	1.85E-09	16.48595353
LOC101060400	1.270370978	4.842751973	11.79276734	1.52E-11	1.87E-09	16.4642701
MIR143HG	-1.476475324	4.666901471	-11.77805901	1.56E-11	1.90E-09	16.43810292
ANGPTL1	-2.482036796	3.976784618	-11.77772642	1.56E-11	1.90E-09	16.43751092
LRRC2	-2.130632195	3.385000663	-11.7690061	1.58E-11	1.92E-09	16.421985
PCAT19	-1.691941481	4.464133857	-11.74594439	1.65E-11	1.99E-09	16.38088381
GATA3-AS1	-1.084899069	3.956024794	-11.71124261	1.75E-11	2.10E-09	16.31892395
DACH1	-4.283921324	4.471041678	-11.70742423	1.77E-11	2.10E-09	16.31209793
DMRT2	-3.418081646	3.700937599	-11.70167941	1.78E-11	2.11E-09	16.30182492
PROM2	-1.314134594	5.391807052	-11.6663324	1.90E-11	2.24E-09	16.23853418
HS3ST3A1	-2.381137803	3.602997108	-11.64484527	1.97E-11	2.31E-09	16.19999081
SCN4A	-1.525156237	4.479004575	-11.6133019	2.09E-11	2.43E-09	16.14331325

EYA4	-2.08435528	3.706092985	-11.60995755	2.10E-11	2.43E-09	16.13729742
HAVCR1	4.931606711	7.622512635	11.47559484	2.67E-11	3.08E-09	15.89454334
CCBE1	-2.183645802	4.335625516	-11.47177881	2.69E-11	3.08E-09	15.88761853
LOC284578	-3.388854609	4.340231054	-11.42545095	2.93E-11	3.33E-09	15.80341456
SNAI1	-1.968771678	5.457775256	-11.40553702	3.03E-11	3.43E-09	15.76714318
KCNN3	-1.366872366	4.095812087	-11.37412674	3.21E-11	3.61E-09	15.70983859
TMEM88	-2.043630229	5.854173204	-11.35069375	3.35E-11	3.75E-09	15.66701283
GATA2	-1.130567963	5.530197885	-11.32569849	3.51E-11	3.90E-09	15.62126127
DPT	-2.117933079	4.072968257	-11.30382378	3.65E-11	4.04E-09	15.58116166
CKMT2	-3.158299049	4.428124761	-11.28309813	3.79E-11	4.17E-09	15.54311675
FLT1	-1.149171259	5.166389067	-11.27164794	3.87E-11	4.24E-09	15.52207668
TCF7L1	-3.19306177	5.392943232	-11.25625637	3.98E-11	4.33E-09	15.49376993
LOC100130691	-3.149273809	4.610384917	-11.23225416	4.16E-11	4.51E-09	15.44957171
GRAMD1B	-2.214212851	5.482973914	-11.1902512	4.49E-11	4.84E-09	15.37206321
PAMR1	-2.481257019	5.793595248	-11.16107992	4.74E-11	5.05E-09	15.31811053
LINC00645	-4.548014913	4.282588208	-11.11861715	5.12E-11	5.41E-09	15.23939494
NTNG1	-1.826274133	4.427959806	-11.0869918	5.43E-11	5.68E-09	15.18063016
HEPACAM2	-3.342814799	3.4982646	-11.08633164	5.43E-11	5.68E-09	15.17940222
TNNT2	-1.38938087	4.665346363	-11.06182751	5.68E-11	5.88E-09	15.13378598
LYVE1	-2.91864722	4.967524456	-11.01871524	6.16E-11	6.34E-09	15.05335541
RNF212B	-2.556944462	4.391025287	-11.00032484	6.37E-11	6.52E-09	15.01897851
ANXA4	1.906053632	11.41923801	10.97427629	6.69E-11	6.80E-09	14.97021698
TNFRSF12A	2.792700383	9.34173834	10.97278389	6.70E-11	6.80E-09	14.96742082
IRX2	-2.480133111	4.874619547	-10.95194977	6.97E-11	7.00E-09	14.92835807
ATP6V1B1	-1.636001257	4.987785533	-10.93325389	7.21E-11	7.21E-09	14.89325999
PYGM	-1.226967409	4.530504527	-10.88250551	7.93E-11	7.89E-09	14.79777686
WBSCR17	-1.637933778	4.779868612	-10.80843584	9.11E-11	9.01E-09	14.65785582
CXCL12	-3.552718726	7.348885834	-10.78964494	9.44E-11	9.25E-09	14.62225308
LAMC3	-1.130766648	4.996025715	-10.77063156	9.78E-11	9.54E-09	14.58618509
ELAC2	1.071296211	6.347811697	10.72292191	1.07E-10	1.04E-08	14.49548688
PRKG1	-1.177588243	4.272462156	-10.71649223	1.08E-10	1.05E-08	14.48324253
LYPD6	-1.578649684	4.068448	-10.69808701	1.12E-10	1.08E-08	14.44816458
SOAT1	1.788308649	7.859607098	10.66158635	1.20E-10	1.15E-08	14.37847638
SMCO3	-1.687451813	3.742020247	-10.62602438	1.29E-10	1.22E-08	14.3104231
ARPC1B	2.445205516	9.679624807	10.61778741	1.31E-10	1.24E-08	14.29463822
ALDOB	-3.159000668	4.78164569	-10.61653956	1.31E-10	1.24E-08	14.29224618
LINC00202-2	1.119778262	5.342437292	10.61403642	1.31E-10	1.24E-08	14.28744725
LENEP	-1.093133584	5.030383486	-10.58966551	1.38E-10	1.29E-08	14.24068398
HRG	-3.75054511	4.553080111	-10.58675355	1.38E-10	1.29E-08	14.23509156
CYP39A1	-1.486173591	4.298597514	-10.55364545	1.47E-10	1.37E-08	14.17143394
ADAMTS4	-1.310303782	4.738259304	-10.54049753	1.51E-10	1.40E-08	14.14611659
LRRTM2	-1.329115396	3.3210256	-10.52739221	1.55E-10	1.42E-08	14.12085997
PAK6	-1.755086783	4.868770367	-10.5153306	1.59E-10	1.45E-08	14.097596
SLC14A1	-2.711328361	3.784045512	-10.47626799	1.71E-10	1.56E-08	14.02212972

RBP7	-2.55044887	5.959483942	-10.46959092	1.73E-10	1.57E-08	14.00921112
ROBO4	-1.394074488	5.696850333	-10.4611583	1.76E-10	1.59E-08	13.992888
PDZRN4	-1.925925728	3.374166811	-10.43493398	1.85E-10	1.66E-08	13.94206876
APCDD1L	-1.304389901	5.761813974	-10.43252698	1.86E-10	1.66E-08	13.93740002
HOXD9	-1.168814756	5.497387883	-10.4079218	1.95E-10	1.74E-08	13.88963315
RHOJ	-1.555102882	4.383030013	-10.40026526	1.98E-10	1.75E-08	13.87475385
RAB25	-3.760833443	5.088656324	-10.37741003	2.07E-10	1.83E-08	13.83029466
SPATA16	1.154752719	5.406038411	10.35289628	2.17E-10	1.91E-08	13.78253661
RPRM	-1.945955041	4.840271521	-10.3450723	2.20E-10	1.93E-08	13.76727799
PCDH19	-1.187267136	3.395109258	-10.32871872	2.27E-10	1.98E-08	13.73535982
DDX25	-1.677744481	3.781485284	-10.3146285	2.33E-10	2.03E-08	13.70783227
SORCS2	-1.71990977	4.894664146	-10.28732732	2.46E-10	2.13E-08	13.65442398
SIM2	-1.273474778	4.203472563	-10.28184129	2.49E-10	2.14E-08	13.64368055
PLVAP	-2.289786449	7.216587785	-10.27708138	2.51E-10	2.15E-08	13.63435604
SLFN13	2.638854213	5.241290792	10.25658265	2.61E-10	2.23E-08	13.59416707
HOXD8	-2.808931377	7.146640774	-10.23877838	2.70E-10	2.30E-08	13.5592178
NRK	-2.643474767	3.926435549	-10.22488092	2.78E-10	2.35E-08	13.53190969
GJA4	-1.864188744	5.651585549	-10.21971614	2.81E-10	2.36E-08	13.52175484
GAS2	-3.067115724	4.665767385	-10.11236067	3.46E-10	2.89E-08	13.30991086
GABRB3	-1.657106661	3.817432944	-10.10025261	3.54E-10	2.95E-08	13.28592633
EHD3	-1.133098647	5.628985853	-10.0956225	3.57E-10	2.96E-08	13.27674975
SCUBE3	-1.4760072	5.131748142	-10.08920005	3.62E-10	2.98E-08	13.26401633
SMIM5	-2.81514571	6.043833759	-10.07461238	3.73E-10	3.05E-08	13.23507471
CDH13	-1.329602299	3.576144636	-10.03561715	4.02E-10	3.28E-08	13.15757588
LINC00284	-1.631844954	3.544596482	-10.0124891	4.21E-10	3.42E-08	13.11151967
MECOM	-2.67012923	4.238429523	-10.00904522	4.24E-10	3.43E-08	13.10465585
PKNOX2	-1.200483391	5.251931091	-9.951967411	4.74E-10	3.79E-08	12.99067554
FAM222A	-1.466063338	5.486771317	-9.951905428	4.74E-10	3.79E-08	12.99055154
ALOX5	2.123713551	6.222410291	9.943332794	4.83E-10	3.83E-08	12.97339647
HSPA12B	-1.257311169	4.774637063	-9.905520748	5.20E-10	4.11E-08	12.89761655
LINC00982	-2.525464169	4.837323682	-9.888427417	5.38E-10	4.24E-08	12.86329911
GNA14	-1.2136279	4.699603813	-9.851737631	5.79E-10	4.53E-08	12.78951181
GHR	-3.679098875	4.917549127	-9.795929458	6.47E-10	5.04E-08	12.67694228
CCDC178	-1.599892075	4.396199071	-9.75086366	7.08E-10	5.45E-08	12.58574704
NEFH	-2.005286142	3.946424139	-9.744812546	7.17E-10	5.50E-08	12.57348195
AK311120 LSAMP	-1.005326942	3.880375578	-9.708022	7.72E-10	5.90E-08	12.49880846
NR2F1-AS1	-2.151988757	4.00309868	-9.699480925	7.85E-10	5.98E-08	12.48144757
TMEM55A	-1.386454153	6.012475719	-9.696860044	7.89E-10	5.99E-08	12.47611837
SH2D3C	-1.20958739	4.864450991	-9.684256674	8.09E-10	6.12E-08	12.4504787
LINGO2	-2.24842893	4.127470103	-9.676888771	8.22E-10	6.19E-08	12.43548024
LOC101927943	-1.094880271	4.006792314	-9.67247094	8.29E-10	6.22E-08	12.42648369
SEMA3D	-1.131763508	3.426900867	-9.669262456	8.34E-10	6.24E-08	12.41994829
DCHS1	-2.079050562	5.498957019	-9.646058218	8.74E-10	6.49E-08	12.37264341
USP46	-1.016201602	5.113215683	-9.603819388	9.52E-10	7.04E-08	12.28635386

EMP2	-1.740244215	5.110333945	-9.602374969	9.55E-10	7.04E-08	12.28339895
TBC1D24	-1.505674004	5.66904536	-9.588762538	9.81E-10	7.20E-08	12.25553799
LILRA6	2.058807409	5.789816593	9.587653694	9.84E-10	7.20E-08	12.25326742
NTF3	-1.324038501	5.596423609	-9.574932508	1.01E-09	7.36E-08	12.22720688
WNK3	-1.815685846	4.251904582	-9.562813617	1.03E-09	7.49E-08	12.20236051
KIF5C	-1.053771333	4.181805085	-9.535922763	1.09E-09	7.86E-08	12.1471598
RAMP2	-2.19336778	5.895034926	-9.523130254	1.12E-09	8.03E-08	12.12086649
TNMD	-1.212057634	3.757555035	-9.513520929	1.14E-09	8.16E-08	12.1011017
EBF1	-2.538133241	4.293076434	-9.499429962	1.18E-09	8.37E-08	12.07209703
GPR20	-1.233702396	4.445081536	-9.491629686	1.20E-09	8.47E-08	12.05602984
GALNT15	-1.362300557	3.929343531	-9.473495889	1.24E-09	8.76E-08	12.01864656
NRN1	-2.180580041	4.428892501	-9.462081259	1.27E-09	8.88E-08	11.99509288
PNPLA3	-1.825957356	4.707850168	-9.454242136	1.29E-09	8.96E-08	11.97890721
ASPRV1	-1.328148735	4.809748423	-9.454036758	1.29E-09	8.96E-08	11.97848306
PODXL	-4.099443235	7.594602199	-9.406384187	1.42E-09	9.84E-08	11.87991829
PLAC9	-1.384526216	5.580050224	-9.404869435	1.43E-09	9.84E-08	11.87678027
KLK6	-2.093222671	4.767414406	-9.395846431	1.45E-09	9.99E-08	11.85808161
CYYR1	-3.498392437	5.312667671	-9.394258146	1.46E-09	9.99E-08	11.85478904
KIAA2022	-1.040406645	3.340930252	-9.373363035	1.52E-09	1.04E-07	11.81144188
SLC34A1	-2.316159191	4.727619934	-9.342035927	1.62E-09	1.10E-07	11.74634556
ATP12A	-1.281388032	5.242886127	-9.284788225	1.83E-09	1.23E-07	11.62705268
DLC1	-1.261294117	5.339750023	-9.27291465	1.87E-09	1.26E-07	11.60225626
SFRP1	-2.802011245	4.747339673	-9.256461822	1.94E-09	1.30E-07	11.56786582
SELP	-1.75027134	5.040070785	-9.254149827	1.95E-09	1.30E-07	11.56303032
AHNAK2	2.517075051	6.794325536	9.247701635	1.97E-09	1.31E-07	11.54954027
GPX1	1.699307433	11.21624273	9.203839877	2.16E-09	1.42E-07	11.45763238
TNFSF9	2.195846111	5.293681322	9.195370846	2.20E-09	1.43E-07	11.439857
PLCG2	-1.302159227	4.37997	-9.191834919	2.21E-09	1.44E-07	11.43243274
TENM2	-2.902069979	4.068333248	-9.180770033	2.27E-09	1.47E-07	11.40918948
NRIP2	-1.850277344	5.118371536	-9.168919953	2.32E-09	1.49E-07	11.3842788
NOL3	1.374298607	6.11303534	9.167511851	2.33E-09	1.49E-07	11.38131751
HLA-F	1.80315881	8.76882509	9.167491213	2.33E-09	1.49E-07	11.38127411
FREM1	-3.068823593	4.491837743	-9.158334324	2.37E-09	1.51E-07	11.36201042
NAT8L	-1.281557349	5.553472314	-9.141732458	2.46E-09	1.56E-07	11.32705605
WT1	-3.786607741	4.374356889	-9.131986893	2.51E-09	1.58E-07	11.30652021
S100A11	1.773043438	11.12969879	9.131662422	2.51E-09	1.58E-07	11.30583627
GSTA3	-1.982738835	4.546760053	-9.103146902	2.66E-09	1.67E-07	11.24567465
LPHN3	-2.533197511	3.796123638	-9.085388728	2.76E-09	1.73E-07	11.20815407
SERPINI1	-2.440368863	5.345837473	-9.062870636	2.90E-09	1.81E-07	11.16051608
PC	-1.656639882	6.379817543	-9.055146706	2.94E-09	1.83E-07	11.14416023
EHF	-1.936647755	3.997289002	-9.038051068	3.05E-09	1.89E-07	11.10793099
DNM3	-1.266180433	3.953904722	-9.003585774	3.28E-09	2.01E-07	11.03477339
UBE2QL1	-2.845721985	4.118771284	-8.981990512	3.43E-09	2.10E-07	10.98885358
ZBED6CL	2.096177915	6.853915766	8.974912628	3.48E-09	2.12E-07	10.97378975

PEG3	-2.935697676	4.24105161	-8.961028504	3.59E-09	2.16E-07	10.94422079
OLFML1	-3.13948247	4.857530078	-8.94384263	3.72E-09	2.23E-07	10.90758447
NTRK3	-1.019434423	4.910705219	-8.939571957	3.75E-09	2.25E-07	10.89847426
LMOD1	-1.674744546	4.444952942	-8.929157277	3.84E-09	2.29E-07	10.87624743
ARL15	-1.467416801	5.747193944	-8.89933524	4.09E-09	2.42E-07	10.81252159
LNX1	-2.283931438	5.384504618	-8.896802895	4.11E-09	2.42E-07	10.80710482
TSPAN5	-2.010744651	4.742785287	-8.890220761	4.17E-09	2.44E-07	10.7930214
SH3BP4	-1.178553177	5.876808566	-8.889503578	4.17E-09	2.44E-07	10.79148654
C10orf95	-1.21267004	5.291937384	-8.885043477	4.21E-09	2.46E-07	10.7819398
ARHGEF15	-1.188447064	5.444744595	-8.873329833	4.32E-09	2.51E-07	10.75685435
HS3ST3B1	-1.574377689	4.39292435	-8.846948616	4.56E-09	2.65E-07	10.70029024
DENND2A	-1.588767715	4.927453093	-8.83536757	4.68E-09	2.69E-07	10.67542985
LOC100506388	-1.181390758	4.087542835	-8.831625202	4.72E-09	2.71E-07	10.66739247
CLCNKB	-3.077883633	6.270778921	-8.81698651	4.86E-09	2.78E-07	10.63593538
WNK4	-2.187495369	4.618081777	-8.813448471	4.90E-09	2.80E-07	10.62832818
LOC100505985	-3.70887086	3.978507693	-8.806862753	4.97E-09	2.83E-07	10.61416365
NEGR1	-1.210289898	3.339249352	-8.783040135	5.23E-09	2.97E-07	10.5628776
DPF3	-1.06593757	4.172186126	-8.736084772	5.78E-09	3.25E-07	10.46156807
RCN1	1.68183149	9.239995328	8.72787524	5.88E-09	3.29E-07	10.44382508
TNFRSF10B	1.736391472	7.210626919	8.727668537	5.88E-09	3.29E-07	10.44337822
SHANK3	-1.187285154	5.666294562	-8.726038369	5.90E-09	3.29E-07	10.43985388
GSTM3	-2.653877445	7.168804249	-8.684240042	6.46E-09	3.57E-07	10.34936616
C10orf82	-1.051601015	4.079598671	-8.679892288	6.52E-09	3.59E-07	10.33994041
LOC101929040	-2.392003571	3.874894774	-8.679288652	6.53E-09	3.59E-07	10.33863155
LINC00622	-1.934125819	3.834382893	-8.660311142	6.80E-09	3.71E-07	10.29745789
ACBD3	1.228855259	7.998721738	8.659846016	6.80E-09	3.71E-07	10.29644815
PARM1	-2.861382447	5.551025001	-8.65935775	6.81E-09	3.71E-07	10.29538814
EFS	-2.343644215	4.653471941	-8.637290385	7.14E-09	3.88E-07	10.24744716
SLC51A	-1.679669584	3.755209983	-8.630576026	7.24E-09	3.92E-07	10.23284737
TIMP1	2.112312131	11.32722457	8.62188053	7.38E-09	3.97E-07	10.21393079
CNN1	-2.78662261	5.290029314	-8.591009838	7.89E-09	4.21E-07	10.14669132
HIC1	-1.554650187	4.368955312	-8.55204348	8.58E-09	4.54E-07	10.06163614
SLC12A3	-2.384335877	4.751837467	-8.549330044	8.63E-09	4.56E-07	10.0557057
BHLHE41	1.671903406	5.869724073	8.545921568	8.69E-09	4.58E-07	10.0482548
HSPB1	2.013797909	12.06025793	8.528117379	9.04E-09	4.73E-07	10.00930965
COL14A1	-1.531707972	4.343381745	-8.525428653	9.09E-09	4.75E-07	10.0034246
LY6K	-1.035629177	3.921785579	-8.520699865	9.18E-09	4.79E-07	9.993071925
RASIP1	-1.06034665	4.668699065	-8.517945572	9.24E-09	4.80E-07	9.987040603
REN	-3.736789179	5.141913751	-8.515188612	9.29E-09	4.82E-07	9.981002425
SEPT6	-1.304511037	5.324505603	-8.508120825	9.43E-09	4.88E-07	9.965518188
CYP26B1	-1.532832302	4.738879433	-8.502223956	9.56E-09	4.93E-07	9.952594093
ITGA9-AS1	-1.051972513	3.897372539	-8.494751269	9.71E-09	5.00E-07	9.936209594
PRSS35	-2.897991127	3.536400475	-8.488122545	9.85E-09	5.06E-07	9.921669287
SALL3	-1.87219139	3.823516993	-8.482862752	9.97E-09	5.08E-07	9.910127576

LRRTM1	-1.733938975	4.490771138	-8.451681945	1.07E-08	5.38E-07	9.841630437
AQP2	-3.105714265	5.837670923	-8.440045165	1.09E-08	5.49E-07	9.816033659
IL13RA2	-1.640305927	3.508239156	-8.436295251	1.10E-08	5.51E-07	9.807781313
TTC36	-1.634959324	5.155188815	-8.428436215	1.12E-08	5.59E-07	9.790480001
LINC01018	-1.004402041	3.613740966	-8.415353357	1.15E-08	5.74E-07	9.761660305
TLN2	-1.280425625	5.743608076	-8.40437162	1.18E-08	5.85E-07	9.737451363
PPP1R16B	-2.093619884	4.456317934	-8.396143668	1.20E-08	5.91E-07	9.719302468
HRC	-1.393760124	4.806114276	-8.391866871	1.21E-08	5.95E-07	9.70986529
CAPG	1.890185641	9.223524898	8.364017194	1.29E-08	6.28E-07	9.64835223
LOC728485	-1.10697356	4.61476936	-8.359550225	1.30E-08	6.33E-07	9.638476122
NRGN	-1.71983889	5.351868582	-8.356304721	1.31E-08	6.36E-07	9.631298898
CCL11	-1.793360269	4.404668404	-8.34144921	1.36E-08	6.55E-07	9.598428862
PSCA	-1.977510993	4.135347733	-8.333454281	1.38E-08	6.65E-07	9.580726646
HLA-G	1.70212531	10.24820843	8.313876092	1.44E-08	6.93E-07	9.537340836
DGKK	-1.619049892	4.999354037	-8.310493177	1.45E-08	6.96E-07	9.529839001
PANK1	-1.88516817	7.725190969	-8.301577777	1.48E-08	7.08E-07	9.510061169
CHP2	-1.096081372	4.502878564	-8.294717345	1.50E-08	7.17E-07	9.494834811
S1PR3	-1.745105212	5.930231613	-8.285894703	1.53E-08	7.28E-07	9.475244167
SEL1L3	1.285005769	6.272393003	8.279191176	1.55E-08	7.37E-07	9.460352043
SYNPO2	-1.896913057	5.066088904	-8.246260377	1.67E-08	7.89E-07	9.387107728
CCSER1	-1.182108173	3.445390482	-8.236883495	1.71E-08	8.03E-07	9.366225227
LOC101927824	1.013929424	5.304311075	8.231246954	1.73E-08	8.11E-07	9.353666876
LDHA	1.177275738	12.17617436	8.229182324	1.73E-08	8.13E-07	9.349065767
OCIAD2	1.649704427	10.89731023	8.215796802	1.79E-08	8.36E-07	9.319221774
CASP5	1.108833117	4.252503315	8.213950957	1.79E-08	8.37E-07	9.315104449
GAS6-AS1	2.415802024	5.257738977	8.202177452	1.84E-08	8.53E-07	9.288831854
LINC01187	-1.483287368	3.755998292	-8.200893458	1.85E-08	8.54E-07	9.285965498
SLC2A12	-1.659825976	4.071696131	-8.177653065	1.94E-08	8.97E-07	9.234046142
PDGFRA	-1.418711149	4.576465989	-8.138586673	2.12E-08	9.67E-07	9.146608699
FXYD3	-1.062431059	6.019101798	-8.122442946	2.20E-08	9.97E-07	9.110416707
DDN	-1.515237837	7.369627837	-8.110053358	2.26E-08	1.02E-06	9.08261739
OPCML	-1.297238865	4.194859395	-8.106351704	2.28E-08	1.03E-06	9.074307778
HMCN1	-3.200855982	3.930595468	-8.090189185	2.36E-08	1.06E-06	9.038004143
MGAM	-3.221396542	4.666924553	-8.08839599	2.37E-08	1.07E-06	9.033974191
RANBP3L	-1.582953948	3.162950729	-8.072102973	2.46E-08	1.10E-06	8.997338303
AFAP1L2	-2.624056363	4.493613902	-8.062913725	2.51E-08	1.11E-06	8.976660094
SLC13A2	-2.085891731	5.331670457	-8.03813073	2.65E-08	1.16E-06	8.920835832
PPP1R1B	-1.289642724	5.37552645	-8.038130165	2.65E-08	1.16E-06	8.920834559
NTRK2	-2.486173589	4.381147539	-8.030231863	2.70E-08	1.18E-06	8.903026275
PRIMA1	-1.131469187	5.131706188	-8.015313171	2.79E-08	1.22E-06	8.8693665
ANKRD35	-1.368017869	4.920996985	-7.999712198	2.89E-08	1.26E-06	8.834135702
PSMB8	2.08097488	8.904376172	7.984055952	2.99E-08	1.29E-06	8.798747573
ADAMTS5	-2.402721122	4.505192911	-7.982999444	3.00E-08	1.29E-06	8.796358354
MLKL	1.999259919	7.232220476	7.970258155	3.08E-08	1.33E-06	8.767533153

CD248	-2.16771445	6.245262639	-7.962854403	3.13E-08	1.35E-06	8.750773409
LOC100130278	-1.441941182	3.906039955	-7.954137624	3.20E-08	1.37E-06	8.73103207
SLIT3	-1.011919419	4.820705508	-7.949836866	3.23E-08	1.38E-06	8.721288207
ARL6IP5	1.462496957	10.79622613	7.934460912	3.34E-08	1.42E-06	8.686432161
EPN3	-1.554514777	4.195562445	-7.933501704	3.35E-08	1.42E-06	8.684256676
CXCL16	2.808577671	8.036697263	7.92896769	3.38E-08	1.43E-06	8.673971875
SLC26A4	-1.692551454	3.354662539	-7.926320441	3.40E-08	1.44E-06	8.667965686
SCN7A	-1.21248273	3.188399495	-7.925024922	3.41E-08	1.44E-06	8.665026021
DNM3OS	-2.247052325	3.610105312	-7.91651763	3.48E-08	1.46E-06	8.645716585
FOXC2	-1.91813212	5.105500263	-7.897075551	3.63E-08	1.52E-06	8.601551933
HTATIP2	1.844765647	6.748789498	7.890871758	3.68E-08	1.53E-06	8.587448867
DPP6	-2.013599525	4.293962494	-7.883432109	3.74E-08	1.56E-06	8.57052962
TMEM178B	1.57788079	5.98660312	7.880189628	3.77E-08	1.56E-06	8.563153281
ADAMTS19	-1.098163342	3.099281943	-7.880026353	3.77E-08	1.56E-06	8.562781808
SLC4A9	-1.118185949	4.395405451	-7.872656702	3.84E-08	1.58E-06	8.546011221
PP7080	-2.744998094	6.658023663	-7.862857096	3.92E-08	1.61E-06	8.523699846
LOC101930114	-1.134669158	5.109819958	-7.856423815	3.98E-08	1.63E-06	8.509045901
GJA3	-1.364602624	4.179049212	-7.846610986	4.07E-08	1.66E-06	8.486683383
SLC26A10	-1.333914343	4.565653234	-7.818072786	4.34E-08	1.77E-06	8.421575329
TPPP3	-1.787415432	5.848317887	-7.814332453	4.38E-08	1.78E-06	8.413034046
MIR205	-1.14265626	3.615732166	-7.805424211	4.46E-08	1.81E-06	8.392684105
LOC100131043	-1.092103895	5.135638454	-7.797225661	4.55E-08	1.84E-06	8.37394615
ERP27	-3.101824302	6.234515532	-7.79230656	4.60E-08	1.86E-06	8.362699199
CXXC4	-1.337607855	4.170929704	-7.791361439	4.61E-08	1.86E-06	8.360537926
LEF1	-1.402834361	4.410910861	-7.780460515	4.72E-08	1.90E-06	8.335601538
IRAK4	1.299488916	5.974994145	7.774914385	4.78E-08	1.92E-06	8.322908504
LRP6	-1.041914023	7.017774286	-7.774550763	4.79E-08	1.92E-06	8.322076168
HLA-J	1.890097378	10.20480259	7.773522396	4.80E-08	1.92E-06	8.319722124
ABRACL	1.643920088	8.062978958	7.762540888	4.92E-08	1.96E-06	8.294575598
OGN	-3.760257777	3.871305338	-7.753780346	5.02E-08	1.98E-06	8.274503503
TUBAL3	-1.496862033	3.737838781	-7.751607321	5.04E-08	1.99E-06	8.269523122
CACHD1	-1.529958925	5.517095043	-7.724354497	5.36E-08	2.10E-06	8.207009506
ERICH4	-2.025336658	4.216060046	-7.719306368	5.43E-08	2.12E-06	8.195419223
ALX1	-2.512176744	5.05028185	-7.714273222	5.49E-08	2.14E-06	8.18386002
TFPI2	4.827141781	9.259143547	7.708271137	5.56E-08	2.16E-06	8.170071201
RAMP3	-2.042650149	5.988078725	-7.704212041	5.61E-08	2.18E-06	8.160743413
PIK3AP1	2.140680247	7.387356246	7.7015288	5.65E-08	2.18E-06	8.154576152
DACT3	-1.284178801	4.972207261	-7.699609811	5.67E-08	2.18E-06	8.150164901
BDKRB2	-1.885881391	5.8012701	-7.694479424	5.74E-08	2.20E-06	8.138369119
SLC7A8	-1.926795321	5.926544034	-7.686408501	5.85E-08	2.24E-06	8.1198055
CYP3A7-CYP3AP1	1.607483826	7.443951343	7.680873869	5.92E-08	2.26E-06	8.10707059
PGAM2	-1.757356055	4.558710969	-7.676778711	5.98E-08	2.28E-06	8.09764526
RNF126P1	1.034253114	5.902803326	7.676242332	5.98E-08	2.28E-06	8.09641058
C3orf70	-1.754836983	3.554087963	-7.675055768	6.00E-08	2.28E-06	8.093679117

LOC375196	-1.050735266	3.581198954	-7.674079565	6.01E-08	2.28E-06	8.091431765
SCN4B	-1.252070711	3.79960272	-7.64697628	6.40E-08	2.41E-06	8.028986724
GATA5	-1.438376928	3.772984273	-7.644963457	6.42E-08	2.42E-06	8.024345431
GDPD3	-1.554004174	5.257004029	-7.608194895	6.99E-08	2.63E-06	7.939469396
LOC441179	-1.791010583	3.610508386	-7.601914561	7.09E-08	2.66E-06	7.924954394
GPR116	-3.163648291	6.640902638	-7.59638806	7.18E-08	2.68E-06	7.9121774
FRZB	-1.360244796	4.543380272	-7.594570123	7.21E-08	2.69E-06	7.907973557
LINC00948	-1.453395879	3.703492847	-7.592296508	7.25E-08	2.70E-06	7.902715387
LINC01314	-1.009051098	4.832938445	-7.58550097	7.36E-08	2.73E-06	7.886995409
TUBB1	-1.023736488	3.900020954	-7.583560655	7.39E-08	2.73E-06	7.882505821
HSD11B2	-4.691984351	5.587645071	-7.574439082	7.55E-08	2.79E-06	7.861393372
KANK4	-2.594819153	4.818853232	-7.572273046	7.59E-08	2.79E-06	7.856378365
EPHB3	-1.172089482	5.867119314	-7.564404252	7.72E-08	2.84E-06	7.838154688
MRO	-1.595792719	4.295881404	-7.562301998	7.76E-08	2.85E-06	7.833284632
PCDH17	-3.063631369	4.721124658	-7.534360421	8.27E-08	3.02E-06	7.768501259
LINC00924	-2.210294975	4.399483146	-7.530319225	8.35E-08	3.04E-06	7.759123279
VGLL3	-1.525143833	3.361427083	-7.529896272	8.36E-08	3.04E-06	7.758141657
RASSF9	-2.107757786	3.614848287	-7.522174726	8.51E-08	3.08E-06	7.7402168
ESRRB	-1.383396122	4.59288066	-7.511624947	8.72E-08	3.15E-06	7.715714036
OLFML3	-2.689610794	7.153554655	-7.505881841	8.83E-08	3.19E-06	7.702369157
TMEM30B	-2.021936	4.749822909	-7.496693587	9.02E-08	3.24E-06	7.681010183
XPNPEP2	-3.382501167	5.476331956	-7.460088754	9.81E-08	3.50E-06	7.595811066
CMTM4	-1.320664234	7.224866775	-7.454997616	9.93E-08	3.52E-06	7.583947636
GSTM5	-1.407146815	5.920382718	-7.443149076	1.02E-07	3.61E-06	7.556325179
BMF	-1.036385437	5.9363241	-7.440758189	1.03E-07	3.62E-06	7.550749135
LOC145837	-1.603498888	3.833769334	-7.439570199	1.03E-07	3.62E-06	7.547978225
TFCP2L1	-1.938743064	6.432039206	-7.439200449	1.03E-07	3.62E-06	7.54711577
SFXN2	-1.275381834	5.588725608	-7.423613998	1.07E-07	3.74E-06	7.510743919
FAM174B	-1.448333758	4.712245079	-7.423400953	1.07E-07	3.74E-06	7.510246552
MNX1	1.539051025	6.421667524	7.415571911	1.09E-07	3.80E-06	7.491965129
MUC20	-1.875246968	4.898765085	-7.398723882	1.13E-07	3.93E-06	7.45259716
MEIS2	-4.166396219	6.453898642	-7.363428138	1.23E-07	4.23E-06	7.370006213
ABHD17C	-1.552676918	6.581421655	-7.362737387	1.23E-07	4.23E-06	7.3683883
ATP6V1G3	-3.130803148	4.005720694	-7.361081537	1.23E-07	4.24E-06	7.364509632
TNNI1	-1.60863987	4.358709076	-7.357967409	1.24E-07	4.26E-06	7.357214146
PPP1R35	1.144820935	7.654619274	7.356968036	1.25E-07	4.27E-06	7.354872649
SLC5A12	-3.391505542	3.749624062	-7.342572359	1.29E-07	4.40E-06	7.321130029
APOBEC3C	1.110947036	6.841053106	7.330527141	1.32E-07	4.51E-06	7.292876594
ARHGEF26	-1.028611773	3.376992198	-7.325748934	1.34E-07	4.56E-06	7.281663683
HEPH	-1.657584211	3.863228589	-7.321571026	1.35E-07	4.59E-06	7.271857117
NCAM1	-1.072849131	4.340253549	-7.319105375	1.36E-07	4.61E-06	7.266068603
RAPGEF4	-1.509730911	3.208165295	-7.317726952	1.36E-07	4.62E-06	7.262832195
ANKRD29	2.771294327	6.495946926	7.316819929	1.37E-07	4.62E-06	7.26070246
LPGAT1	1.651327153	7.378796553	7.30307027	1.41E-07	4.75E-06	7.228404883

TRIM56	1.175857517	8.288293142	7.300206969	1.42E-07	4.77E-06	7.22167607
PELI2	-1.008589135	5.430698408	-7.297036527	1.43E-07	4.79E-06	7.214224266
TMSB10	1.299021931	13.09697867	7.279603404	1.49E-07	4.97E-06	7.173226876
LRRC17	-2.107024915	4.447935524	-7.271563021	1.52E-07	5.05E-06	7.154305482
MMP7	3.248035412	10.59221012	7.261181243	1.56E-07	5.16E-06	7.129862099
SNCAIP	-1.148626712	4.228276101	-7.259688475	1.56E-07	5.16E-06	7.12634634
CATSPER1	1.301181694	4.856206452	7.246621091	1.61E-07	5.28E-06	7.0955582
CD36	-1.25587829	3.873802944	-7.21315119	1.74E-07	5.67E-06	7.016602273
HGFAC	1.177272902	4.890542366	7.212916027	1.74E-07	5.67E-06	7.016047025
MMRN2	-1.877405255	4.624003407	-7.210248561	1.75E-07	5.70E-06	7.009748353
LOC102723864	-1.65356491	5.04692443	-7.198153913	1.80E-07	5.85E-06	6.981178226
GAS1	-2.24009116	5.033005571	-7.196910593	1.81E-07	5.86E-06	6.978240208
MEIS1	-3.270063043	5.06694987	-7.191672546	1.83E-07	5.91E-06	6.965860379
MYOM2	-1.305746112	5.37165692	-7.18729038	1.85E-07	5.96E-06	6.955500759
ZNF521	-3.044471802	3.804385669	-7.18466748	1.86E-07	5.99E-06	6.949298979
FAM46C	-1.665451547	5.022382741	-7.181161907	1.88E-07	6.03E-06	6.941008807
TTTY15	-1.953686817	3.784272715	-7.17888549	1.89E-07	6.05E-06	6.935624594
LGI2	-1.27371246	6.012129905	-7.178353711	1.89E-07	6.05E-06	6.934366731
NPY	-1.254860726	4.557183608	-7.167870332	1.94E-07	6.19E-06	6.90956233
SLC16A5	-1.743337901	5.13908485	-7.165579622	1.95E-07	6.22E-06	6.904140542
INSM2	-1.315966332	4.37796472	-7.152420441	2.01E-07	6.38E-06	6.872982047
PLAT	-3.372572276	6.934894694	-7.152136847	2.01E-07	6.38E-06	6.872310315
IGFBP2	-3.287096453	6.4217219	-7.150482904	2.02E-07	6.39E-06	6.868392515
ST3GAL6	-1.104868184	4.243762124	-7.149527249	2.02E-07	6.40E-06	6.86612864
FBN3	-1.036755713	4.421104181	-7.136415491	2.08E-07	6.56E-06	6.835056504
LPPR1	-1.751896902	4.223735218	-7.132137541	2.10E-07	6.62E-06	6.824914073
HSD17B2	-1.722082835	4.220249998	-7.123517998	2.15E-07	6.73E-06	6.804471483
EMX1	-1.45337163	5.765845355	-7.123375019	2.15E-07	6.73E-06	6.80413231
ZNF442	-1.082238876	3.830212887	-7.122958713	2.15E-07	6.73E-06	6.803144736
HEXB	1.413847346	10.24887896	7.112762562	2.20E-07	6.88E-06	6.778950491
CCR5	1.949619565	6.498491053	7.111363083	2.21E-07	6.89E-06	6.775628699
FABP1	-2.198048222	4.319164242	-7.103755878	2.25E-07	7.00E-06	6.7575681
FAM169A	-3.012589155	4.324425383	-7.10176173	2.26E-07	7.02E-06	6.752832533
NFASC	-1.231976194	4.630104175	-7.095235646	2.29E-07	7.11E-06	6.737331429
CLIC1	1.092059231	10.64622961	7.09205272	2.31E-07	7.15E-06	6.729769281
GPR124	-1.93004176	5.310405172	-7.068685136	2.44E-07	7.51E-06	6.674213577
MUC15	-3.42022272	3.911184115	-7.047403111	2.57E-07	7.89E-06	6.623558349
TYRP1	-4.153919079	3.794988858	-7.044539146	2.59E-07	7.92E-06	6.616737373
ITGA1	-1.412559437	4.928345581	-7.043935427	2.59E-07	7.92E-06	6.615299394
TNIK	1.748917017	6.584403505	7.035511942	2.64E-07	8.06E-06	6.595231178
TEP1	1.188742667	5.282028503	7.017722304	2.75E-07	8.38E-06	6.552820688
SULF2	2.108109995	8.902931417	7.008297972	2.82E-07	8.55E-06	6.530337593
WLS	-1.681512193	6.89618952	-6.99742206	2.89E-07	8.76E-06	6.504378239
HLA-A	1.528353762	12.61987158	6.996427679	2.90E-07	8.76E-06	6.502004074

ATP6V0D2	-2.897235298	4.766841439	-6.987743146	2.96E-07	8.91E-06	6.481263988
LSP1	1.826331727	6.237021945	6.981748203	3.00E-07	9.02E-06	6.466941799
TMEM174	-1.788885626	5.174221995	-6.976406353	3.04E-07	9.12E-06	6.454176245
NPAS2	1.267884644	6.328846468	6.973359537	3.06E-07	9.17E-06	6.446893661
ANXA2	1.200538741	10.000505	6.965907146	3.11E-07	9.32E-06	6.42907606
NR2F1	-4.094333558	5.842355207	-6.957936786	3.17E-07	9.47E-06	6.410012717
SPATA18	2.498297935	6.105847174	6.957694596	3.17E-07	9.47E-06	6.409433333
SERPINA5	-3.450500891	6.384608229	-6.953973217	3.20E-07	9.53E-06	6.40052991
SECTM1	1.698289129	6.760234767	6.949291231	3.24E-07	9.63E-06	6.389325878
OSMR	1.613387141	6.846851068	6.941661366	3.30E-07	9.77E-06	6.371061948
APBB1IP	2.336073306	6.40851271	6.937720867	3.33E-07	9.83E-06	6.361626692
PEAR1	-1.546789958	5.752571297	-6.937625423	3.33E-07	9.83E-06	6.361398135
LINC00577	1.008936951	5.663773148	6.924864416	3.43E-07	1.01E-05	6.330829961
SLC34A2	3.791886931	7.819532029	6.918960983	3.48E-07	1.02E-05	6.316682123
PLAC1	-1.975548342	4.544221125	-6.915257471	3.51E-07	1.03E-05	6.307804384
COPG2IT1	-1.982468627	5.23748812	-6.913421383	3.53E-07	1.03E-05	6.303402471
SOCS2	-1.589133204	4.982169779	-6.912311578	3.54E-07	1.03E-05	6.300741584
PPBP	-3.37301467	4.188442292	-6.910484057	3.55E-07	1.03E-05	6.296359572
KCNMB2	-2.338304454	4.451865243	-6.906852588	3.58E-07	1.04E-05	6.287650897
EGF	-4.989683633	4.616588872	-6.89824236	3.66E-07	1.05E-05	6.266996364
NPHS1	-1.886886644	5.235818898	-6.886727472	3.76E-07	1.07E-05	6.239360343
TSHZ2	-1.094791291	4.262609268	-6.872762468	3.88E-07	1.11E-05	6.20582303
TYRO3	-1.614811551	5.587122167	-6.871649601	3.89E-07	1.11E-05	6.203149465
SQRDL	1.751373947	9.861960686	6.87048744	3.90E-07	1.11E-05	6.20035732
C7	-3.060159879	6.047859018	-6.860341847	4.00E-07	1.13E-05	6.175975341
TMEM74B	1.611811663	5.38248369	6.858723107	4.02E-07	1.13E-05	6.172084054
LCN2	3.937987242	8.257343782	6.854134249	4.06E-07	1.14E-05	6.161051238
MYH10	-1.075799386	8.213756581	-6.852904839	4.07E-07	1.14E-05	6.158094996
GAD1	-1.256208828	3.764474154	-6.851585918	4.08E-07	1.14E-05	6.15492332
SKI	-1.365100104	5.93941685	-6.823857109	4.36E-07	1.21E-05	6.08819542
C19orf33	2.785797155	9.018800369	6.819772372	4.41E-07	1.22E-05	6.078358152
MAFG-AS1	1.044813705	5.851223135	6.816733948	4.44E-07	1.23E-05	6.071039462
ANGPTL3	-1.402434442	3.320118294	-6.810081939	4.51E-07	1.24E-05	6.055012945
GATSL3	-1.057590584	5.056159681	-6.809238121	4.52E-07	1.24E-05	6.052979588
CGN	-1.425257502	5.52334332	-6.809101304	4.52E-07	1.24E-05	6.052649891
CYP4A11	-2.984992273	5.208099504	-6.805042915	4.56E-07	1.25E-05	6.042869137
ITPR1-AS1	-1.697458652	4.589276887	-6.799056951	4.63E-07	1.27E-05	6.02843943
SLC7A13	-3.914456771	3.750473704	-6.789358716	4.74E-07	1.29E-05	6.005052167
PIPOX	-3.083306876	5.174092496	-6.781431217	4.83E-07	1.32E-05	5.98592697
LOC149703	-1.84580523	5.349304044	-6.778134069	4.87E-07	1.32E-05	5.977970425
C2orf40	-3.259026088	5.586156884	-6.773891116	4.92E-07	1.34E-05	5.967729661
LOC100505942	-1.143230576	4.411675414	-6.772377243	4.93E-07	1.34E-05	5.964075286
ADH1B	-2.529410985	4.349010521	-6.772157184	4.94E-07	1.34E-05	5.963544059
LOC101927609	1.508165528	4.734875597	6.771253986	4.95E-07	1.34E-05	5.961363658

SOX7	-1.875376945	3.832031265	-6.761658905	5.06E-07	1.36E-05	5.938194501
MIR34A	1.285827011	5.171172541	6.745036011	5.27E-07	1.40E-05	5.898030385
TAGLN2	1.518505468	9.665895358	6.74496462	5.27E-07	1.40E-05	5.897857824
RAB11FIP3	-1.611736466	6.423005732	-6.742028486	5.31E-07	1.41E-05	5.890760253
HSPB8	1.693598876	7.392468976	6.740273778	5.33E-07	1.41E-05	5.886518094
SLC43A1	-1.122205925	5.650429335	-6.733975062	5.41E-07	1.43E-05	5.871287515
ESAM	-2.030450304	6.382924826	-6.728157529	5.48E-07	1.44E-05	5.857216445
SCRN1	1.400463575	9.79087123	6.722848387	5.56E-07	1.45E-05	5.844371682
LINC00271	-1.073597869	3.93175431	-6.721650248	5.57E-07	1.46E-05	5.8414725
MST1L	-2.516205386	4.112680655	-6.719044024	5.61E-07	1.46E-05	5.835165562
CSF3R	1.31081644	5.91437944	6.712694132	5.69E-07	1.48E-05	5.8197959
MAN1C1	-2.078050773	5.748274904	-6.70748258	5.76E-07	1.50E-05	5.80717813
HS6ST2	-3.894149968	4.603652578	-6.707057842	5.77E-07	1.50E-05	5.806149655
ATP6V1C2	-1.822603078	4.088173966	-6.68386529	6.10E-07	1.58E-05	5.749959506
PPIC	1.204771394	8.759418238	6.682481833	6.12E-07	1.58E-05	5.746605796
LOC102723493	-2.083281496	4.084126452	-6.674957148	6.23E-07	1.61E-05	5.728361058
BSND	-1.307008115	5.034446327	-6.674430497	6.24E-07	1.61E-05	5.727083874
ARHGAP28	-1.481166344	4.030186669	-6.673908762	6.25E-07	1.61E-05	5.725818583
EMILIN1	-1.999979077	5.125626571	-6.671419158	6.29E-07	1.61E-05	5.719780469
AHRR	-1.341907349	3.838485489	-6.671266946	6.29E-07	1.61E-05	5.719411283
THRB	-1.151419351	5.036894326	-6.659342442	6.47E-07	1.65E-05	5.69048059
PGAP1	-1.633525304	3.897694417	-6.655871404	6.52E-07	1.66E-05	5.682056327
RAPH1	-1.267306165	5.274823325	-6.655464772	6.53E-07	1.66E-05	5.681069337
PNMA2	1.467873031	6.444985277	6.655382414	6.53E-07	1.66E-05	5.680869432
SLC5A3	-2.15675449	6.6671846	-6.653302663	6.56E-07	1.67E-05	5.675821086
SLC13A3	-1.790721038	4.741153592	-6.652841316	6.57E-07	1.67E-05	5.674701157
VAT1	1.277461041	10.08344477	6.645903475	6.68E-07	1.69E-05	5.65785654
ERBB4	-2.17668183	4.370447541	-6.633307394	6.89E-07	1.74E-05	5.627260401
IGFBP5	-1.971245305	5.771382831	-6.624386284	7.04E-07	1.77E-05	5.605580195
ARMC10	1.050296568	8.347473825	6.623894062	7.05E-07	1.77E-05	5.604383734
ITM2C	-1.817789158	8.544783602	-6.613694581	7.22E-07	1.81E-05	5.579585468
DSG2	1.614799136	9.368980102	6.609688947	7.29E-07	1.82E-05	5.569843325
GSTT2	-1.527611127	4.573867095	-6.608458906	7.31E-07	1.82E-05	5.566851376
PLP2	1.658815734	9.437767986	6.603726086	7.40E-07	1.83E-05	5.555337716
APOC1	2.635745358	8.770914909	6.584606531	7.75E-07	1.91E-05	5.508800006
TNFRSF14	1.036676522	7.144631821	6.579559765	7.84E-07	1.93E-05	5.496509309
CDKN1C	-1.962273877	6.969144749	-6.571791546	7.99E-07	1.96E-05	5.477585457
AFP	-3.371161544	4.354048663	-6.563637346	8.15E-07	1.98E-05	5.457714252
RUNX1T1	-1.166785499	4.055490614	-6.553745142	8.35E-07	2.02E-05	5.433597954
ASPN	-2.405992087	4.808832757	-6.540811552	8.61E-07	2.08E-05	5.402051033
PLOD3	1.690904233	9.243412541	6.532621576	8.78E-07	2.12E-05	5.382065135
GNB2	1.212054549	8.749174867	6.528215786	8.88E-07	2.14E-05	5.371310749
SEMA6D	-1.833808489	5.088218572	-6.522004246	9.01E-07	2.16E-05	5.356145043
LTBP1	-2.024493605	6.522064667	-6.52191219	9.01E-07	2.16E-05	5.355920255

PLG	-2.520011795	4.74177724	-6.52121144	9.03E-07	2.16E-05	5.35420908
MIR100HG	-2.944802471	3.929402102	-6.511863348	9.24E-07	2.20E-05	5.331376766
C9orf40	-1.002009498	4.553417018	-6.506845621	9.35E-07	2.22E-05	5.319117323
HLX	-1.376448251	4.935499389	-6.502170996	9.46E-07	2.24E-05	5.307693733
C1orf168	-1.44616481	3.753454516	-6.499722476	9.51E-07	2.25E-05	5.301709247
GPR183	2.350826939	5.858159715	6.48645957	9.82E-07	2.32E-05	5.269281962
PCDH18	-2.807031202	5.593195378	-6.481512385	9.94E-07	2.34E-05	5.257181502
RARRES2	2.372933505	10.09968257	6.4759611	1.01E-06	2.36E-05	5.243600369
SYT17	-1.614390846	5.296138163	-6.471982331	1.02E-06	2.37E-05	5.233864366
NOTCH3	-1.40567876	5.714763512	-6.466750208	1.03E-06	2.40E-05	5.221058876
KCNE4	-1.309335385	4.069126514	-6.462816616	1.04E-06	2.42E-05	5.211429605
GSTA1	-2.92893963	5.072851591	-6.461988266	1.04E-06	2.42E-05	5.209401631
NES	-1.858342348	5.70362177	-6.451553872	1.07E-06	2.48E-05	5.183849868
GMPR	-1.726803603	5.542164649	-6.445906562	1.08E-06	2.51E-05	5.170015956
CDH3	-1.994301112	4.978941066	-6.443941289	1.09E-06	2.51E-05	5.165200949
STAP1	-2.035425813	3.676881985	-6.439253173	1.10E-06	2.54E-05	5.153713224
PF4	-1.838614291	5.787353084	-6.437480349	1.11E-06	2.55E-05	5.14936851
SLC22A8	-2.852559765	6.2688328	-6.430161924	1.13E-06	2.59E-05	5.13142955
APCDD1	-3.665608575	5.389892691	-6.42471706	1.14E-06	2.61E-05	5.118079453
SLC5A2	-1.329461351	6.040376603	-6.416054573	1.17E-06	2.66E-05	5.096833814
GC	-2.708652707	3.719788117	-6.415367891	1.17E-06	2.66E-05	5.095149324
ASPHD1	2.316463554	6.302225448	6.409690868	1.18E-06	2.69E-05	5.081221218
S100A12	-1.58507521	3.845243196	-6.406154633	1.19E-06	2.71E-05	5.072543673
AGXT	-1.200039865	4.422216703	-6.395134596	1.23E-06	2.77E-05	5.045493395
ZNF662	-1.921370508	4.502732509	-6.3924482	1.23E-06	2.78E-05	5.038897353
OR51E1	-1.194351145	3.929214536	-6.391971312	1.24E-06	2.78E-05	5.037726347
SALL2	-1.401039451	7.331150073	-6.388509049	1.25E-06	2.80E-05	5.029224015
ZNF10	-1.117338012	5.185537037	-6.383922746	1.26E-06	2.83E-05	5.017959468
SNAI2	-3.870936222	5.668049644	-6.381895219	1.27E-06	2.84E-05	5.012978913
SPP1	1.86165857	8.579088159	6.380851045	1.27E-06	2.84E-05	5.010413769
IGSF9	-1.09438837	5.039778817	-6.380783396	1.27E-06	2.84E-05	5.010247577
UPP2	-2.765538804	4.434224888	-6.380729733	1.27E-06	2.84E-05	5.010115745
CAV2	1.707104542	7.8876394	6.378100794	1.28E-06	2.85E-05	5.003656901
KCNE1	-1.067740783	3.6652675	-6.377911291	1.28E-06	2.85E-05	5.0031913
HS3ST1	2.19519425	6.445316921	6.375757196	1.29E-06	2.86E-05	4.997898496
NELL1	-1.330079183	4.22506203	-6.37538094	1.29E-06	2.86E-05	4.996973953
BAX	1.618497613	6.392521651	6.347592901	1.38E-06	3.05E-05	4.92865286
TCEAL7	-2.999364523	3.83308046	-6.345106413	1.39E-06	3.06E-05	4.922535639
MN1	-2.378597068	3.873727484	-6.339430959	1.40E-06	3.10E-05	4.90857063
CDC42EP3	-1.833637774	6.424660451	-6.337164198	1.41E-06	3.11E-05	4.902992136
BHLHB9	-1.44546491	5.229855545	-6.332714779	1.43E-06	3.14E-05	4.892040622
MYH11	-1.23614869	5.233435641	-6.331936294	1.43E-06	3.14E-05	4.890124303
TRIM63	-1.193520617	3.88049306	-6.329212974	1.44E-06	3.16E-05	4.883420103
P2RX7	1.398715097	4.750380107	6.318368133	1.48E-06	3.23E-05	4.856715178

PMS2P8	1.055016649	6.220959637	6.317608007	1.48E-06	3.23E-05	4.854842959
DTX3L	1.45946872	8.593900694	6.312686569	1.50E-06	3.26E-05	4.842719891
DNMT3L	-1.133604063	4.093751276	-6.30905971	1.51E-06	3.29E-05	4.833784235
APOD	-2.114338318	6.117681524	-6.303672858	1.53E-06	3.32E-05	4.820509992
MPP6	1.331476579	4.867441579	6.300478657	1.55E-06	3.34E-05	4.812637501
BEND5	-1.295708277	5.323383945	-6.296753249	1.56E-06	3.37E-05	4.803454511
PHGDH	-2.292010483	6.669260523	-6.296059457	1.56E-06	3.37E-05	4.801744188
CASR	-1.12696554	5.009643818	-6.28496568	1.60E-06	3.45E-05	4.774389549
FAM189A1	3.041965075	6.712793118	6.282135767	1.62E-06	3.46E-05	4.767409706
MYOF	1.230119375	7.916275556	6.281906825	1.62E-06	3.46E-05	4.766844996
STARD3NL	1.045016052	9.746219547	6.274146018	1.65E-06	3.52E-05	4.747699127
RNF186	-1.994814759	4.978226041	-6.270744958	1.66E-06	3.54E-05	4.739306866
TREH	-1.51479523	4.402362679	-6.268966079	1.67E-06	3.55E-05	4.734916956
SLC25A34	-1.339161721	5.137140123	-6.264277708	1.69E-06	3.59E-05	4.723345526
LINC00472	-2.1297295	4.815840143	-6.257889425	1.71E-06	3.64E-05	4.707575067
ZNF614	-1.04110287	4.626304997	-6.256371794	1.72E-06	3.65E-05	4.703827975
CDKN2A	1.167829099	5.548289786	6.251468555	1.74E-06	3.69E-05	4.691720153
MFAP4	-1.929146758	5.814906886	-6.249277055	1.75E-06	3.70E-05	4.686307812
PLN	-2.606511576	4.431935903	-6.245673981	1.77E-06	3.72E-05	4.677408298
STC1	-2.481520639	5.921424286	-6.237350358	1.80E-06	3.79E-05	4.656844326
SERPINB1	1.25440784	7.355806119	6.221110642	1.88E-06	3.92E-05	4.616703962
MRPS6	-1.559198885	9.043828135	-6.207019436	1.94E-06	4.03E-05	4.581853661
ATP8B3	1.216263581	4.315749376	6.205473036	1.95E-06	4.04E-05	4.57802796
HLA-B	1.876839981	12.02148603	6.196892197	1.99E-06	4.12E-05	4.556795334
THEMIS2	2.253419974	6.465562859	6.19681504	1.99E-06	4.12E-05	4.556604384
DIO1	-3.989131304	4.091107471	-6.176767329	2.09E-06	4.32E-05	4.506970556
EGFL7	-1.393045086	6.206287106	-6.175524407	2.10E-06	4.33E-05	4.503892101
ITGA3	2.105775013	8.52123097	6.17496452	2.10E-06	4.33E-05	4.502505333
CASQ2	-1.148992803	4.729371824	-6.174782964	2.10E-06	4.33E-05	4.502055634
TPK1	3.055355827	8.206779896	6.173286361	2.11E-06	4.34E-05	4.498348569
IL18	3.001822065	6.378578778	6.172182776	2.12E-06	4.34E-05	4.495614869
HOXB-AS3	-1.189774645	4.698089939	-6.171452133	2.12E-06	4.34E-05	4.493804925
IQGAP2	-1.186830517	5.119597808	-6.155984824	2.20E-06	4.50E-05	4.455477682
ANKRD45	1.478969261	4.653295358	6.154642954	2.21E-06	4.51E-05	4.452151541
CDC42EP2	-1.183406247	6.16143463	-6.150756055	2.23E-06	4.53E-05	4.442516005
PAQR6	1.284753529	5.648672909	6.148203444	2.25E-06	4.54E-05	4.436187377
EGFLAM	-1.430088423	5.042299348	-6.142884858	2.27E-06	4.60E-05	4.422999191
SCUBE2	-1.23842874	4.296134849	-6.131452737	2.34E-06	4.71E-05	4.394642793
CCDC3	-1.669605954	6.82762404	-6.120308924	2.41E-06	4.82E-05	4.366989959
NSUN5	1.095738071	8.130105326	6.109525773	2.47E-06	4.93E-05	4.340221288
TDRD7	1.266941689	6.970127509	6.10721021	2.48E-06	4.95E-05	4.334471631
GBAP1	1.024278157	8.185233597	6.103105006	2.51E-06	5.00E-05	4.324277011
GCAT	-1.022717058	6.553323972	-6.100548376	2.53E-06	5.02E-05	4.317927255
TNFRSF10A	1.123492277	5.853786648	6.076959852	2.68E-06	5.28E-05	4.259314007

RSPO3	-3.870486657	4.596296621	-6.07393964	2.70E-06	5.32E-05	4.251805723
PRNP	1.173696734	9.036317746	6.073214023	2.70E-06	5.32E-05	4.250001707
FOXI1	-1.795828865	4.429029371	-6.072010619	2.71E-06	5.33E-05	4.247009725
ABCB4	1.606364832	4.928133095	6.062626399	2.77E-06	5.45E-05	4.223673635
LINC01094	2.28372061	4.871150878	6.058236011	2.80E-06	5.50E-05	4.212753214
MME	-3.999664395	6.092086709	-6.050490581	2.86E-06	5.58E-05	4.19348348
LHX1	-1.215526758	5.447184345	-6.04760861	2.88E-06	5.61E-05	4.186312122
NAP1L5	-2.551042886	5.237784964	-6.047214725	2.88E-06	5.61E-05	4.185331941
PALM3	-2.108331933	6.194239314	-6.042241617	2.92E-06	5.67E-05	4.172955208
SLC16A2	-1.714670685	5.75652421	-6.042201386	2.92E-06	5.67E-05	4.172855074
TENC1	-1.329861807	7.042343371	-6.03907048	2.94E-06	5.71E-05	4.165061967
RARS	1.284384041	8.678723586	6.036572415	2.96E-06	5.74E-05	4.15884344
CTH	-1.055196442	4.478202672	-6.027368761	3.03E-06	5.86E-05	4.135927749
MET	2.917744561	8.660547253	6.018475181	3.09E-06	5.96E-05	4.1137771
HLA-C	1.623577692	11.48645658	6.016150642	3.11E-06	5.99E-05	4.107986398
ALG1	1.355233525	5.701431916	6.014032192	3.13E-06	6.01E-05	4.10270868
KRT80	2.093143965	5.727996268	6.006427795	3.19E-06	6.11E-05	4.083760583
ADH6	-2.217323166	4.604208973	-5.991845157	3.30E-06	6.33E-05	4.047410731
PDE8B	-1.41365559	4.054710593	-5.982267192	3.38E-06	6.47E-05	4.023526074
MAGI2-IT1	-1.861764436	3.563205359	-5.981169544	3.39E-06	6.48E-05	4.020788363
NCEH1	2.641337306	7.57715367	5.968852225	3.50E-06	6.64E-05	3.990060015
ARC	-1.327035832	4.37285072	-5.966523469	3.52E-06	6.67E-05	3.984248972
ANXA2P2	1.327259039	9.593295659	5.963327203	3.55E-06	6.71E-05	3.976272453
FST	-1.88447299	4.371165511	-5.96192172	3.56E-06	6.72E-05	3.972764696
DAPL1	-2.108492181	4.5605938	-5.959091476	3.58E-06	6.77E-05	3.965700569
RHOBTB3	-1.543868006	5.976651057	-5.952090616	3.65E-06	6.87E-05	3.948223952
MGAT4B	1.212391622	8.990378542	5.950082935	3.66E-06	6.89E-05	3.943211322
C4orf17	-1.014466332	5.713246645	-5.949138961	3.67E-06	6.90E-05	3.940854362
LOC727944	-1.490384935	4.540033677	-5.94653404	3.70E-06	6.92E-05	3.934349883
MCC	-1.98711023	5.61120255	-5.945661185	3.71E-06	6.93E-05	3.932170241
TREM2	1.904185839	7.489173108	5.943536888	3.72E-06	6.96E-05	3.926865311
YARS2	1.066652316	7.342376985	5.941916933	3.74E-06	6.98E-05	3.922819605
GRN	1.097098344	9.745721393	5.938506499	3.77E-06	7.03E-05	3.914301618
ATF7IP2	-1.107776849	5.52874184	-5.933987112	3.81E-06	7.09E-05	3.903012404
C14orf132	-2.154026844	5.469463958	-5.932696934	3.83E-06	7.11E-05	3.899789293
ELFN1	-1.197523317	6.452070447	-5.930912464	3.84E-06	7.13E-05	3.895331124
GLCE	-1.642487773	5.221508098	-5.922938616	3.92E-06	7.26E-05	3.875406746
ERVH-4	-1.021827154	4.546067434	-5.915509294	3.99E-06	7.37E-05	3.856838323
LIPA	1.6478515	10.2955236	5.912862783	4.02E-06	7.41E-05	3.850222703
CD86	1.713949227	5.423508608	5.912598485	4.02E-06	7.41E-05	3.849561992
UGCG	1.89740547	9.487142383	5.905032641	4.10E-06	7.53E-05	3.830645977
FECH	-1.024162315	6.741586624	-5.902569847	4.12E-06	7.56E-05	3.824487542
AFM	-2.529260342	3.693632103	-5.901863857	4.13E-06	7.57E-05	3.82272206
CUBN	-3.238358459	5.778766441	-5.891849852	4.24E-06	7.73E-05	3.797675567

CSTA	2.338539664	6.219060754	5.880524564	4.36E-06	7.92E-05	3.769339733
EGFL6	-3.084484564	4.046539685	-5.877880584	4.39E-06	7.96E-05	3.762723038
SLC10A2	-1.789384631	4.036963612	-5.873903657	4.43E-06	8.01E-05	3.752769538
SFXN3	1.268989611	6.73581164	5.871770038	4.45E-06	8.04E-05	3.747428977
CCDC43	1.087310367	7.302889527	5.858169587	4.61E-06	8.28E-05	3.713377943
DNM1	2.02564173	8.226806253	5.85037779	4.70E-06	8.43E-05	3.69386336
SMOC2	-1.44783896	4.459141037	-5.84731568	4.73E-06	8.49E-05	3.686193005
ALB	-1.56903344	3.328044221	-5.847086604	4.73E-06	8.49E-05	3.685619159
EPDR1	1.105989109	8.926225384	5.845512881	4.75E-06	8.51E-05	3.681676792
ACTA2	-1.277502845	5.675991749	-5.838064861	4.84E-06	8.65E-05	3.663016015
LOC644090	-1.260809271	4.604922969	-5.836213036	4.86E-06	8.68E-05	3.658375663
OLIG1	-1.345682156	3.328516362	-5.834905247	4.88E-06	8.70E-05	3.655098413
ZNF580	-1.107266954	7.476938665	-5.832202928	4.91E-06	8.74E-05	3.648326132
DDB2	2.062394746	7.421087303	5.830375778	4.94E-06	8.77E-05	3.643746797
NTS	-1.653097133	3.71353223	-5.830281616	4.94E-06	8.77E-05	3.643510794
RHCG	-2.643964152	5.190536326	-5.819958532	5.07E-06	8.97E-05	3.617633482
CLDN1	2.709229646	8.0587331	5.817898279	5.09E-06	9.01E-05	3.61246799
MT1G	-1.873516787	6.133670029	-5.815706377	5.12E-06	9.05E-05	3.606972076
MIR31HG	1.688141871	5.641131219	5.809254695	5.20E-06	9.17E-05	3.590793207
ALOX5AP	2.410722179	8.391219422	5.802896987	5.29E-06	9.31E-05	3.574846941
CD300A	1.374445671	5.058123856	5.792543399	5.42E-06	9.51E-05	3.548871841
TSHZ3	-1.519195748	5.077749213	-5.789989552	5.46E-06	9.56E-05	3.542463523
TEAD3	-1.232096144	5.787263395	-5.780454043	5.59E-06	9.76E-05	3.518532006
SLC30A2	-1.23910706	5.548643646	-5.776234868	5.65E-06	9.85E-05	3.507940901
IFI35	1.62102699	7.403817647	5.775896625	5.66E-06	9.85E-05	3.507091777
TIMP3	-2.434203499	6.800326256	-5.766795859	5.79E-06	0.00010051	3.484242102
KCNIP1	-1.186080622	4.355820896	-5.763794592	5.83E-06	0.00010118	3.476705377
WEE1	-1.522982257	6.887617837	-5.76077549	5.87E-06	0.00010174	3.469123207
LRRC32	-1.739354861	7.319930856	-5.754733267	5.96E-06	0.00010306	3.453946803
NMI	1.391201245	8.491122139	5.745028595	6.11E-06	0.00010524	3.429565856
ARHGAP30	1.299665939	6.118747448	5.731552497	6.32E-06	0.00010848	3.395698888
ISG20	1.670440724	6.012801	5.731234642	6.32E-06	0.00010848	3.394899928
BACH2	-1.459509975	3.823485456	-5.730323542	6.34E-06	0.00010864	3.392609746
RGS1	2.293519128	5.793222825	5.729780894	6.35E-06	0.00010869	3.391245696
C3	3.990359687	10.31485108	5.726196861	6.40E-06	0.00010958	3.382236012
PTGDS	-2.30715408	6.622089658	-5.725530698	6.41E-06	0.00010967	3.380561285
LOC285043	-1.116691904	3.905569693	-5.725162463	6.42E-06	0.00010968	3.379635533
EVI2B	2.56440972	7.008541752	5.709670143	6.67E-06	0.00011317	3.340678905
DACH2	-2.206560529	3.330204066	-5.705575673	6.74E-06	0.00011424	3.330380271
G6PC	-2.313180417	3.795710292	-5.700862608	6.82E-06	0.00011512	3.318524288
ENOX1	-1.492108406	4.746443612	-5.695032744	6.92E-06	0.00011653	3.303856832
TPRG1L	-1.185917683	7.522878674	-5.691528282	6.98E-06	0.00011727	3.29503878
ADTRP	-1.448995521	4.294158688	-5.674818404	7.28E-06	0.00012179	3.252981396
MAGEH1	-1.467497818	7.666934751	-5.673722931	7.30E-06	0.00012202	3.250223526

COL4A5	-1.219591109	5.322389999	-5.671841525	7.34E-06	0.0001225	3.245486874
RARRES3	2.047245591	8.846041243	5.655222531	7.65E-06	0.0001273	3.203636485
CPED1	-1.4912919	6.132820635	-5.653111634	7.69E-06	0.00012787	3.198319469
ACSF2	-2.630542358	6.686012316	-5.65086631	7.73E-06	0.00012849	3.192663535
GGT6	-1.980276697	4.992216759	-5.642405732	7.90E-06	0.00013072	3.171348524
UPK1A	-2.09256823	4.215686945	-5.640670708	7.93E-06	0.00013118	3.166976849
CDH6	1.912418656	7.233884672	5.63662196	8.02E-06	0.00013241	3.156774615
ARAP3	-1.241312795	5.169991659	-5.63056566	8.14E-06	0.00013374	3.14151169
SUSD5	-1.471801761	3.313584187	-5.623306579	8.29E-06	0.00013593	3.123214468
MTTP	-1.124931014	3.424645338	-5.618155577	8.40E-06	0.00013759	3.110228837
MOXD1	-1.994259978	5.41479218	-5.610566027	8.56E-06	0.00014001	3.091092605
PTPN6	1.106932056	7.361013622	5.593514669	8.93E-06	0.000145	3.048086369
CD37	1.080005432	4.93006916	5.593468987	8.93E-06	0.000145	3.047971127
LMO4	-1.117914765	5.041740469	-5.588097483	9.05E-06	0.00014674	3.034419617
LINC00494	1.551203858	5.625047658	5.580938642	9.22E-06	0.00014905	3.016356188
PCDHGA8	1.234412757	4.45352424	5.574551435	9.37E-06	0.00015087	3.000237156
SPRY4	-1.589449433	6.703006578	-5.573559435	9.39E-06	0.00015101	2.997733482
TRIB2	-2.165167255	6.673995987	-5.571272414	9.45E-06	0.00015176	2.991961124
FAM155B	1.46105328	6.310527139	5.570918707	9.45E-06	0.00015178	2.991068353
DLK1	-1.632027377	4.351379321	-5.569059531	9.50E-06	0.00015225	2.986375592
CHODL	-2.606810307	4.987377206	-5.561537323	9.68E-06	0.00015444	2.967386626
LACTB2	1.871889063	9.022342788	5.559914051	9.72E-06	0.00015495	2.963288418
HECW2	-1.150073835	3.636281052	-5.559049383	9.74E-06	0.00015517	2.961105365
GPR65	1.986081515	5.40219148	5.553573357	9.88E-06	0.0001572	2.947278846
PECAM1	-1.306518599	6.355161926	-5.552719332	9.90E-06	0.00015741	2.945122344
SCARA5	-1.025433653	4.759767533	-5.548166921	1.00E-05	0.00015886	2.933626318
LOC100287590	1.2079563	5.376609117	5.544684884	1.01E-05	0.00016001	2.924832449
GPR155	-1.72905267	4.629720592	-5.544227533	1.01E-05	0.00016007	2.923677358
PPAPDC1A	-1.554439049	5.488470847	-5.538255908	1.03E-05	0.00016224	2.90859426
HSPB6	-1.256969702	5.174158988	-5.537649681	1.03E-05	0.00016234	2.907062938
LEAP2	-2.261293469	5.594266045	-5.530202522	1.05E-05	0.00016519	2.888249816
IL33	-1.087701204	3.350516336	-5.528936385	1.05E-05	0.00016546	2.885050966
LPXN	1.004745752	5.723792988	5.526459169	1.06E-05	0.00016624	2.878792109
SPECC1L	-1.335369623	6.913583985	-5.525317764	1.06E-05	0.00016659	2.875908153
HSPA2	-3.041381577	7.58137385	-5.519720157	1.08E-05	0.00016831	2.861763771
FZD10	-1.468906874	3.410206323	-5.518637869	1.08E-05	0.00016864	2.859028777
SIRPA	1.180884004	7.090066541	5.508936358	1.11E-05	0.00017255	2.834509653
AHSP	-1.186686811	5.250118634	-5.504110216	1.12E-05	0.00017427	2.822310355
CHGB	-1.958455184	5.279335403	-5.498636515	1.13E-05	0.00017615	2.808472639
ZNF788 ZNF788	-1.510993196	4.228251155	-5.491969041	1.15E-05	0.00017873	2.791614811
CXorf38	1.301650218	6.157839816	5.487364683	1.17E-05	0.00018017	2.779971894
ARNTL	1.269900901	5.962688103	5.48728944	1.17E-05	0.00018017	2.77978162
PSMB10	1.851454904	7.872334919	5.477928393	1.20E-05	0.00018296	2.756107046
APOLD1	-1.796768142	5.890641901	-5.468855819	1.22E-05	0.00018644	2.733157599

AQP3	-1.957777706	6.924232327	-5.468632488	1.22E-05	0.00018644	2.732592621
PWAR6	-2.687883512	6.026911147	-5.465185067	1.23E-05	0.00018774	2.723871053
ADAP2	1.273589878	7.095428425	5.46319385	1.24E-05	0.00018834	2.718833229
AOC3	-2.13810472	5.518127884	-5.461098588	1.25E-05	0.00018906	2.713531945
TMEM163	2.643237729	6.615784778	5.460308248	1.25E-05	0.00018916	2.711532224
HBB	-2.55634856	8.129322152	-5.4586447	1.25E-05	0.00018982	2.707323003
VPS25	1.064512534	9.358717065	5.454501022	1.27E-05	0.00019111	2.696837766
NGF	-1.030218429	5.247377081	-5.45118285	1.28E-05	0.00019257	2.688440772
FANK1	1.133382688	5.890109878	5.445584119	1.30E-05	0.00019488	2.674271302
BLVRA	1.013978627	7.259785008	5.433615109	1.34E-05	0.00020028	2.643974392
ITGAL	1.533505347	5.171139374	5.432754791	1.34E-05	0.00020057	2.641796408
IDH2	-1.326747844	7.495363969	-5.426880313	1.36E-05	0.00020312	2.626923599
TOX	-1.747578956	3.987017964	-5.426060528	1.36E-05	0.00020325	2.624847959
PPP1R14A	-2.326070107	4.721049465	-5.42389745	1.37E-05	0.00020421	2.619371034
FBLN5	-2.246378392	7.843576422	-5.420421684	1.38E-05	0.00020556	2.610569895
CYFIP2	-1.83150069	8.542599985	-5.419072314	1.39E-05	0.00020603	2.607152939
LPL	-2.830169124	5.579980998	-5.412685847	1.41E-05	0.00020856	2.590979539
USP51	-1.170442369	5.439865802	-5.412206482	1.41E-05	0.00020866	2.589765493
NIP7	1.10220883	6.998778547	5.411478308	1.41E-05	0.00020875	2.587921288
FLOT2	1.273830125	7.892277386	5.407793589	1.43E-05	0.00021025	2.578588819
PTGER4	-1.442947558	5.817392917	-5.404519174	1.44E-05	0.00021138	2.570295003
HLA-DPA1	2.55617036	9.482745602	5.403133229	1.44E-05	0.00021197	2.566784369
LY86	1.899586614	7.603481626	5.393191971	1.48E-05	0.00021644	2.541600251
ZMAT3	1.296869272	7.264964135	5.388945895	1.50E-05	0.00021862	2.530842287
TCF4	-1.682203561	6.368537071	-5.377289447	1.54E-05	0.00022452	2.501304955
PGBD5	2.068503318	7.263761849	5.374325063	1.55E-05	0.00022573	2.493792247
TNFAIP8L1	-1.363205297	6.24268992	-5.373698433	1.56E-05	0.00022593	2.492204112
CRYAA	-1.851610168	4.929353142	-5.373167085	1.56E-05	0.00022607	2.490857448
PTH1R	-1.995621703	7.897616824	-5.370853359	1.57E-05	0.00022724	2.484993321
NNAT	-1.136788693	6.014497482	-5.365764241	1.59E-05	0.0002297	2.472094134
PMS2P5	2.100208202	5.837286516	5.361450366	1.60E-05	0.00023189	2.461159023
ANKEF1	1.029864251	4.990557721	5.361356421	1.60E-05	0.00023189	2.460920876
REEP1	-1.941682533	4.956927986	-5.358214324	1.62E-05	0.0002333	2.452955541
SNF8	1.029023846	8.882153055	5.352959806	1.64E-05	0.00023576	2.439634171
SNX10	1.470904334	9.506556984	5.351305923	1.65E-05	0.00023659	2.435440964
FBLN1	-1.743617093	5.376005301	-5.349639992	1.65E-05	0.00023725	2.431217089
HOXD11	-2.440736993	4.455299218	-5.348287246	1.66E-05	0.0002379	2.427787188
RDH12	-1.353194077	4.991198684	-5.34570942	1.67E-05	0.00023929	2.42125087
EBF2	-1.026990308	3.535919426	-5.339617302	1.70E-05	0.00024267	2.40580261
TMEM71	1.930659979	5.010925505	5.336311628	1.71E-05	0.00024386	2.397419493
CTSS	2.451122479	7.272102823	5.324344443	1.76E-05	0.00024963	2.367067163
DSERG1	-1.542316689	5.700487554	-5.323913518	1.76E-05	0.00024973	2.365974098
JAM3	-1.139634242	5.014658383	-5.323002707	1.77E-05	0.00025013	2.363663753
RAB42	1.406646222	6.108140798	5.322486568	1.77E-05	0.00025029	2.362354508

NFKBIZ	3.057274944	9.321539529	5.321800958	1.77E-05	0.00025055	2.360615368
NLGN1	2.301478868	5.810384267	5.321177934	1.78E-05	0.00025077	2.359034966
RAC2	1.790779386	6.891037228	5.318376628	1.79E-05	0.00025157	2.351928807
ZBTB7C	-1.046827048	4.827153495	-5.315191002	1.80E-05	0.00025286	2.343847344
FLJ22763	-2.772403714	3.916553767	-5.310583015	1.82E-05	0.00025531	2.332156837
LINC01159	-1.659101901	6.427552544	-5.309647709	1.83E-05	0.00025574	2.329783853
GPM6A	-1.73418758	4.260038059	-5.309020305	1.83E-05	0.00025597	2.328192036
LOC100507073	-1.865407666	4.894556669	-5.303726113	1.86E-05	0.00025855	2.314759248
ZNF782	-1.236599966	4.062562117	-5.290970937	1.92E-05	0.00026632	2.282391433
PLP1	-1.331784755	3.267956836	-5.289936275	1.92E-05	0.00026684	2.279765576
DAAM1	-1.329552831	6.13918357	-5.283867464	1.95E-05	0.00027024	2.264362792
CD40	1.402586967	6.493830747	5.279934059	1.97E-05	0.00027222	2.254378976
STX3	1.10238964	6.933230122	5.272937432	2.01E-05	0.00027616	2.236618627
ABCC4	1.773466062	6.556310541	5.268186858	2.03E-05	0.00027884	2.224558667
ITGB2	1.521863674	6.584897257	5.266604323	2.04E-05	0.00027932	2.220541011
OGFRL1	1.566535798	6.889777282	5.254843307	2.10E-05	0.00028647	2.190679944
C1orf109	-1.132626375	4.93074354	-5.253972265	2.11E-05	0.00028689	2.188468184
TPST2	-1.830824619	6.456861785	-5.248274505	2.14E-05	0.00029087	2.17399971
ITIH5	-2.16159805	4.842817536	-5.244805994	2.16E-05	0.00029305	2.165191478
GPR110	-1.904414499	4.501710376	-5.239725232	2.18E-05	0.00029607	2.152288211
MT1F	-2.649323893	8.353865221	-5.239183216	2.19E-05	0.00029619	2.150911638
FOXD1	-3.138152725	4.916726286	-5.237397266	2.20E-05	0.00029721	2.146375742
RINT1	1.241092716	6.173446519	5.237170732	2.20E-05	0.00029721	2.145800391
TM4SF5	-1.797138592	5.268972321	-5.222526559	2.28E-05	0.00030605	2.108603483
FXYD4	-3.70105641	4.790097603	-5.220057937	2.30E-05	0.00030757	2.102332372
PALM	-1.033954176	7.733301269	-5.219576066	2.30E-05	0.00030774	2.101108239
PCDHB7	-1.562492805	4.206051212	-5.213847906	2.33E-05	0.00031104	2.08655599
HTR2B	-2.739092082	4.72489672	-5.206675945	2.38E-05	0.00031532	2.068334337
LOC100507535	-1.042204952	4.459479579	-5.203817701	2.39E-05	0.000317	2.061072009
PLA2G4A	-2.002966047	4.581671999	-5.18421329	2.51E-05	0.00033148	2.01125377
CRYBG3	-1.257130168	6.905874395	-5.180926127	2.54E-05	0.00033383	2.002899405
LOC100506325	1.336319439	4.861909744	5.174771901	2.58E-05	0.00033844	1.987257532
DBNDD1	1.662088289	6.569324867	5.174491734	2.58E-05	0.00033846	1.986545421
C16orf89	-2.186713351	5.653790995	-5.168877026	2.61E-05	0.00034245	1.97227384
C9orf116	1.250396669	7.054084142	5.160742296	2.67E-05	0.00034827	1.951595272
TNNC1	-1.747911645	4.268247887	-5.158856605	2.68E-05	0.0003495	1.946801573
NNMT	1.667853493	4.953838229	5.158295622	2.69E-05	0.00034968	1.945375454
ETNK2	-1.36312601	6.083660397	-5.158157369	2.69E-05	0.00034968	1.945023988
SEPP1	-1.839522299	7.625020687	-5.153613601	2.72E-05	0.00035262	1.933472594
EMP3	1.350648319	9.016120443	5.153001445	2.72E-05	0.00035295	1.931916299
PRSS23	-1.152331951	6.219485758	-5.150738496	2.74E-05	0.00035431	1.926163081
KDELR3	1.03090656	6.836865122	5.150263395	2.74E-05	0.00035452	1.924955188
SLC41A2	1.506364136	5.2879056	5.149884682	2.74E-05	0.00035464	1.923992348
GLRX2	1.164591699	7.280974581	5.148024873	2.76E-05	0.00035587	1.919263913

DCN	-1.879437296	6.033297896	-5.143508935	2.79E-05	0.00035975	1.907782085
ECM2	-1.160045887	5.410856222	-5.136356321	2.84E-05	0.00036544	1.889595426
SUCNR1	-3.356995629	5.738323351	-5.13609033	2.84E-05	0.00036546	1.888919077
CPN2	-1.16427809	4.21458203	-5.126863351	2.91E-05	0.00037297	1.865456099
DDIT4L	-1.881362127	5.070305474	-5.124522405	2.93E-05	0.00037496	1.85950306
GABBR2	-1.063758147	4.414715388	-5.121419035	2.95E-05	0.0003777	1.851610975
SOD2	1.3780002	7.982208795	5.114396031	3.00E-05	0.00038331	1.83375017
STARD8	-1.129588183	6.841861361	-5.112927229	3.01E-05	0.00038427	1.830014592
VMP1	2.865836655	8.509052628	5.111137408	3.03E-05	0.00038578	1.825462505
PHF13	-1.009198347	7.543630903	-5.101499229	3.10E-05	0.00039335	1.800948329
ZNF518B	-1.096513795	5.028837096	-5.099934255	3.12E-05	0.00039425	1.796967714
RNF24	-1.052092376	5.28733976	-5.098556196	3.13E-05	0.00039515	1.793462487
BTN3A2	1.288368758	7.174981972	5.094230905	3.16E-05	0.00039903	1.782460431
SERPINE2	1.456274176	7.376363654	5.079044249	3.29E-05	0.00041147	1.743827784
ARG2	-1.990162272	5.594207445	-5.076849062	3.30E-05	0.00041343	1.738243173
FCER1G	2.026156175	7.505828837	5.07658758	3.31E-05	0.00041343	1.737577948
FAM151A	-2.873769931	4.919163577	-5.076456685	3.31E-05	0.00041343	1.737244945
ARRB2	1.075439251	6.404612648	5.076211443	3.31E-05	0.00041344	1.736621037
GPR137B	1.477375637	9.16852316	5.07479607	3.32E-05	0.00041468	1.733020225
ESRRG	-2.695545462	6.518485457	-5.067879613	3.38E-05	0.00042102	1.715423727
FAM43A	-1.093650415	5.809672316	-5.066494437	3.39E-05	0.000422	1.711899529
GPM6B	-1.921764493	5.42866508	-5.06471029	3.41E-05	0.00042341	1.707360206
SLC22A6	-2.376715486	5.131751189	-5.063726689	3.42E-05	0.00042421	1.704857652
BIRC7	1.01250669	5.81002261	5.057822457	3.47E-05	0.00042908	1.689835286
ANK2	-1.842560241	4.683891592	-5.0554347	3.49E-05	0.00043083	1.683759852
HBE1	-1.03714492	4.377087385	-5.053248847	3.51E-05	0.00043226	1.67819806
ENPP6	-2.614472093	3.550444124	-5.052500076	3.52E-05	0.00043259	1.676292834
ZNF649	-1.127822494	4.878847777	-5.051709302	3.52E-05	0.0004332	1.674280719
GALNT5	1.024790099	4.662816005	5.050300582	3.54E-05	0.0004345	1.670696225
LOC284219	1.02307588	5.635098016	5.049041697	3.55E-05	0.00043563	1.66749296
ZNF423	-2.789821776	4.997399806	-5.042085923	3.61E-05	0.00044236	1.64979335
CRTAP	1.061079475	8.588319679	5.034861744	3.68E-05	0.00044978	1.631409962
AHR	1.864496593	9.423791678	5.034501322	3.68E-05	0.00044992	1.630492773
PGM5-AS1	-1.231538271	5.543760743	-5.029838406	3.73E-05	0.00045424	1.618626597
RBPMS2	-1.190135716	6.526654893	-5.025290763	3.77E-05	0.00045842	1.607053468
RHBDF2	1.325523964	6.874547953	5.022925182	3.79E-05	0.00046092	1.601033275
LOC254057	-1.088716432	4.970932423	-5.022447849	3.80E-05	0.00046121	1.599818493
MAMDC2	-1.535295789	3.669709349	-5.0212245	3.81E-05	0.00046238	1.596705142
C9orf24	-1.354451677	5.142997888	-5.019803093	3.82E-05	0.00046378	1.593087715
HAO2	-3.388592822	5.579530245	-5.019535155	3.82E-05	0.00046382	1.592405822
MR1	1.163288075	5.918566245	5.015471885	3.86E-05	0.0004681	1.582064795
FADS3	1.006881758	7.128592994	5.013824061	3.88E-05	0.00046979	1.577871021
TRIL	-2.46312619	5.887296501	-5.010810712	3.91E-05	0.00047314	1.570201845
ENO2	2.171481768	7.041521566	5.003095772	3.99E-05	0.00048083	1.550566291

FAM19A5	-1.006735388	4.014300526	-5.002519589	3.99E-05	0.00048126	1.549099799
VEGFA	-1.566381203	7.223106628	-5.001461438	4.00E-05	0.00048227	1.546406601
LRRN4	2.640206225	7.063078942	5.00087308	4.01E-05	0.00048271	1.544909112
DOCK11	1.344793526	6.254157277	5.000191329	4.02E-05	0.00048327	1.543173911
AGTR2	-2.410919858	3.662879293	-4.997672427	4.04E-05	0.00048554	1.536762732
C1QTNF4	-1.373024362	3.814310547	-4.995605298	4.07E-05	0.00048752	1.531501364
GTF2IRD2	1.003542922	5.786177982	4.991359756	4.11E-05	0.00049168	1.520695239
NYNRIN	-1.271319018	6.28669869	-4.990896962	4.11E-05	0.00049197	1.519517283
HSPA12A	-1.136586126	5.580102195	-4.985568312	4.17E-05	0.00049755	1.505954044
ADRA2A	-1.843258086	4.130185135	-4.983547751	4.19E-05	0.00049953	1.500810949
ROR2	-1.379255743	3.831791922	-4.982247053	4.21E-05	0.0005009	1.497500158
LAIR1	1.306469215	6.792021098	4.980038381	4.23E-05	0.00050315	1.491878178
ASS1	-2.411053082	9.315472544	-4.979334414	4.24E-05	0.00050376	1.490086282
KCNJ8	-1.288653149	6.21630046	-4.978718582	4.24E-05	0.00050397	1.488518723
AGPAT9	-2.798920098	6.641789971	-4.974934415	4.29E-05	0.00050797	1.478886305
GIMAP8	-1.238344862	4.546269534	-4.96291645	4.42E-05	0.00052288	1.448294336
P4HB	1.001443165	9.765835264	4.959443883	4.46E-05	0.00052663	1.439454637
SETBP1	-1.762932561	7.380569219	-4.95738901	4.48E-05	0.00052879	1.43422375
SELENBP1	-1.701233385	7.119623721	-4.949715775	4.57E-05	0.00053677	1.414690497
MFSD4	-2.055293977	5.899414948	-4.94881631	4.58E-05	0.00053739	1.412400764
ZNF615	-1.165278542	6.498731304	-4.946775126	4.60E-05	0.00053896	1.40720458
PILRA	1.249366059	5.47716514	4.946050511	4.61E-05	0.00053965	1.405359943
NR3C2	-2.101630318	6.527810192	-4.942974429	4.65E-05	0.00054328	1.397529192
MTUS1	-1.043245281	6.285650499	-4.938427009	4.70E-05	0.00054744	1.385952773
FAS	2.176563478	7.376904282	4.932348327	4.78E-05	0.00055474	1.370478043
ALDH4A1	-1.565516416	6.807694717	-4.931428504	4.79E-05	0.00055515	1.368136401
ERVMER34-1	-1.487516886	4.60362623	-4.924032356	4.88E-05	0.0005644	1.349307502
B4GALT5	1.588856241	9.290210742	4.922583578	4.90E-05	0.00056617	1.345619222
FXYD6	-1.670234683	7.362539942	-4.91774235	4.96E-05	0.00057257	1.333294435
TNFAIP2	1.175171398	6.736042303	4.91671051	4.97E-05	0.00057376	1.330667571
GGACT	-1.122282744	4.791317606	-4.910513716	5.05E-05	0.00058127	1.314891676
KCNS3	1.88016162	7.043942526	4.905362302	5.12E-05	0.00058765	1.301777063
DRAM1	1.465583629	8.753583487	4.902967234	5.15E-05	0.00058993	1.295679619
ANKRD46	-1.373130874	7.183990502	-4.901843191	5.16E-05	0.0005913	1.292817988
MEX3B	-1.590537024	4.258879249	-4.897247016	5.23E-05	0.00059694	1.281116868
SYT1	-1.378245195	3.882727059	-4.894104561	5.27E-05	0.00059941	1.273116674
WASF3	-1.631806512	7.118301941	-4.889993291	5.32E-05	0.0006044	1.262650025
NEXN	-1.712375789	4.631798098	-4.885950231	5.38E-05	0.00061033	1.252357032
CACNA2D2	-1.007757826	5.646598414	-4.885409827	5.39E-05	0.00061084	1.25098125
FLII	1.003895654	8.614925339	4.883548701	5.41E-05	0.00061341	1.246243119
GSTM1	-1.069972198	7.641585428	-4.883140104	5.42E-05	0.00061371	1.245202896
USP44	-1.027959993	3.757162249	-4.876188574	5.51E-05	0.00062367	1.227505442
JAZF1	1.172127028	6.473157538	4.875119984	5.53E-05	0.00062503	1.224784992
LAPTM5	1.976033207	9.340395623	4.869467558	5.61E-05	0.00063232	1.210394911

OLFML2A	-1.916876171	6.262104212	-4.869287451	5.61E-05	0.00063232	1.209936393
PIK3R3	-1.228103732	5.144574659	-4.866431123	5.65E-05	0.00063625	1.202664713
TKT	1.476832652	7.978918907	4.861650084	5.72E-05	0.00064266	1.190493126
CORO1C	1.265305159	8.906053135	4.860227394	5.74E-05	0.00064429	1.186871252
NEURL1B	-2.175660652	6.149952361	-4.858222754	5.77E-05	0.00064654	1.181767863
AGMAT	-1.956850488	5.316983986	-4.844113155	5.98E-05	0.00066882	1.145848274
TMEM150C	-1.266800439	5.736414827	-4.841721291	6.02E-05	0.00067255	1.139759264
SLC6A19	-1.86476655	6.055356681	-4.837884596	6.08E-05	0.00067844	1.129992192
NAT2	-1.723808128	5.205512976	-4.836546524	6.10E-05	0.00067967	1.126585883
PTN	-2.513041149	5.31302586	-4.832747873	6.16E-05	0.00068516	1.116915775
ASAP2	-1.924403029	7.814412752	-4.832291402	6.17E-05	0.00068559	1.115753757
HTRA4	1.042051308	4.110890849	4.831719662	6.18E-05	0.00068603	1.114298305
AKR1C3	2.090206487	10.56827444	4.830359889	6.20E-05	0.00068712	1.110836805
MFGE8	-1.364133668	6.144420214	-4.826722795	6.26E-05	0.00069204	1.101578111
CLDN11	-1.431689325	4.298850544	-4.818808159	6.38E-05	0.00070314	1.081430716
SLC23A3	-1.086991064	4.885988169	-4.809122117	6.54E-05	0.0007173	1.056774736
CLU	1.499847519	8.472790204	4.804390573	6.62E-05	0.00072526	1.044730815
GSTM2	-1.23548389	8.035293365	-4.793532647	6.81E-05	0.0007425	1.017093314
MUC3B	1.014341761	6.597892291	4.782197882	7.01E-05	0.00076009	0.988243447
GRB14	-2.52958032	6.179901971	-4.781750618	7.02E-05	0.00076035	0.987105079
CASP1	1.862216267	6.531226575	4.779238596	7.06E-05	0.00076363	0.98071156
PHACTR3	-1.254347039	3.399626565	-4.778664205	7.07E-05	0.00076435	0.97924965
COL9A3	-1.023705678	5.374627728	-4.776691474	7.11E-05	0.00076781	0.974228788
HBD	-1.002280903	4.53188601	-4.769930593	7.23E-05	0.00078038	0.957021833
FCGR2B	2.624414034	6.619959809	4.754308474	7.53E-05	0.00080751	0.917264794
CD300LF	1.847646826	5.530870677	4.74917762	7.63E-05	0.00081732	0.904207979
SORD	-1.603649081	5.959613142	-4.742368127	7.76E-05	0.00083038	0.886880077
TMEM100	-2.916812481	4.038626508	-4.741432879	7.78E-05	0.00083127	0.884500241
OTUD3	-1.169670992	5.201382083	-4.741375383	7.78E-05	0.00083127	0.884353937
RAB4B	-1.149661536	7.222915532	-4.740204098	7.81E-05	0.00083281	0.881373501
TMEM200A	1.736832101	8.310722025	4.73907928	7.83E-05	0.00083434	0.878511328
COL21A1	-3.104639119	3.792754196	-4.738528887	7.84E-05	0.00083465	0.877110826
PSMB9	1.927021282	8.190318056	4.730618887	8.00E-05	0.00084859	0.856984011
ATG4C	-1.10589896	5.136410588	-4.729271534	8.03E-05	0.00085023	0.853555813
UTY	-1.078944747	4.190639472	-4.728350082	8.05E-05	0.0008518	0.851211295
DDR2	-1.730173575	5.964390861	-4.727656479	8.06E-05	0.00085283	0.849446522
LOC646903	-1.446558351	4.031661804	-4.727472751	8.06E-05	0.00085283	0.848979053
PSPH	1.363342623	5.034449132	4.725775176	8.10E-05	0.00085548	0.844659857
TES	1.031514198	7.578179596	4.72450297	8.13E-05	0.00085756	0.841422976
HOXC6	-1.490680247	8.41996623	-4.717375388	8.28E-05	0.00086931	0.823288809
TRAM1	1.126355971	9.595750327	4.715463696	8.32E-05	0.00087268	0.818425203
C15orf27	-1.14385464	4.320591863	-4.712773495	8.37E-05	0.00087736	0.811581091
KIAA1586	-1.308829434	3.811332823	-4.707059411	8.50E-05	0.0008871	0.797044439
CCR7	1.127660205	4.365073278	4.705806458	8.52E-05	0.00088904	0.793857011

GPIHBP1	-1.068949642	5.593429232	-4.702297301	8.60E-05	0.00089569	0.784930135
SLFN5	1.221497616	6.37345443	4.689434859	8.89E-05	0.00092097	0.75221193
RHPN2	1.627184971	8.472566254	4.689139073	8.90E-05	0.00092121	0.751459582
CDH10	-1.318908891	3.717567213	-4.681996964	9.06E-05	0.00093583	0.73329388
MAN1A1	-1.557450598	7.100956668	-4.678255268	9.15E-05	0.00094293	0.723777497
SGPP2	1.927101277	6.479392485	4.67770869	9.16E-05	0.00094377	0.722387395
MGC24103	-1.36627942	5.284678584	-4.676023361	9.20E-05	0.0009469	0.718101175
TLR1	1.969165102	5.198941565	4.673181424	9.27E-05	0.00095234	0.710873564
LRRN3	-1.135320576	4.066476625	-4.670906168	9.32E-05	0.0009565	0.705087283
NR0B2	-1.038202992	5.967660294	-4.665090351	9.46E-05	0.00096841	0.690297481
SUSD4	-1.375947481	5.458335313	-4.663892191	9.49E-05	0.0009709	0.687250632
FAM101B	-1.840429964	6.062871698	-4.663466269	9.50E-05	0.00097147	0.686167549
CLDN10	-2.918334691	8.046498112	-4.658993296	9.61E-05	0.0009807	0.674793432
MRAP2	-1.168288043	5.266979513	-4.658450709	9.62E-05	0.00098157	0.67341375
FUT8	1.342816187	6.710410906	4.653854851	9.74E-05	0.0009917	0.661727784
PDE2A	-1.197845287	5.86749214	-4.639926684	0.0001009	0.00101806	0.626316004
SLITRK6	-1.31497377	3.269135045	-4.62963813	0.0001036	0.0010388	0.600161413
LOC101928820	-1.191558276	4.073172888	-4.625561416	0.0001047	0.00104793	0.589798862
ANGPT2	-1.518628719	4.333202637	-4.622172357	0.0001056	0.00105551	0.581184647
BTK	1.112840362	5.916173503	4.620965412	0.0001059	0.00105826	0.578116956
ROBO2	-1.832455864	3.556486174	-4.618246956	0.0001066	0.0010646	0.571207626
RASD1	-2.481548744	7.007773718	-4.618019119	0.0001067	0.00106471	0.570628557
HCG4	1.543599137	5.334379275	4.613263884	0.000108	0.00107512	0.558543078
LIMCH1	-1.521076556	6.550470852	-4.6085719	0.0001093	0.001086	0.546619075
HENMT1	-1.498270096	4.964910607	-4.603907386	0.0001106	0.00109744	0.534765608
HAVCR2	1.172207929	5.617346093	4.603682279	0.0001107	0.00109755	0.534193586
IL32	2.679320161	9.774223073	4.598021469	0.0001123	0.0011098	0.519809366
GSS	1.103894499	9.165782126	4.594094564	0.0001134	0.00111831	0.509831677
FAM198B	-1.745404712	4.942127277	-4.590094901	0.0001146	0.00112763	0.499669678
AGA	1.212962047	7.627721795	4.589964967	0.0001147	0.00112763	0.499339562
TNFAIP8	1.386128966	7.305878962	4.5894034	0.0001148	0.00112816	0.497912831
NLRC5	1.121202391	6.564274052	4.588469906	0.0001151	0.00112963	0.495541195
CTSC	1.753529242	9.347035366	4.584800815	0.0001162	0.00113757	0.486219799
IGSF6	1.460653785	4.31401557	4.583070818	0.0001167	0.00114152	0.48182488
BCL6	1.116692727	6.96842441	4.578695621	0.000118	0.00115218	0.470710524
RNASET2	1.16182773	6.763387344	4.577292841	0.0001184	0.00115522	0.467147181
ASPHD2	-1.263991113	4.828954019	-4.57531875	0.000119	0.00116041	0.462132713
RNF213	1.176925911	6.931888805	4.559042351	0.0001241	0.0012013	0.420794054
OLR1	2.518701033	5.137276262	4.54671009	0.0001281	0.00123631	0.389479644
CNRIP1	-1.753102281	6.136190868	-4.543552076	0.0001291	0.00124157	0.381461728
KLHL14	-1.178436035	4.743966996	-4.543397228	0.0001292	0.00124157	0.381068595
DHDH	-1.370263841	5.554368973	-4.541707845	0.0001297	0.00124637	0.376779583
MS4A6A	1.485659336	6.773787584	4.540720223	0.0001301	0.00124835	0.37427226
NANOS1	-1.241165284	4.395854958	-4.538757822	0.0001307	0.00125288	0.369290342

MIF4GD	1.132442757	8.023453474	4.537230669	0.0001312	0.00125661	0.365413494
SLC17A2	1.563779228	4.841010094	4.536627937	0.0001314	0.00125796	0.363883419
ASMTL	-1.150701929	6.941779399	-4.535352059	0.0001319	0.00126089	0.36064457
BTG2	-1.254597088	7.999865249	-4.532497871	0.0001328	0.00126836	0.353399394
CCNJL	-1.053372133	4.816711234	-4.526330503	0.0001349	0.00128674	0.337745118
GPRC5B	-1.514004094	5.694445875	-4.525843906	0.0001351	0.00128774	0.33651009
SPTBN2	-1.115791315	5.932411657	-4.521477593	0.0001366	0.00129798	0.325428432
ECI2	-1.023351113	9.05078345	-4.513160693	0.0001396	0.00132104	0.304322591
TRIM22	2.071902278	8.963855981	4.510116049	0.0001406	0.00132949	0.296596968
GPD1	-1.215678635	4.805143751	-4.508407574	0.0001413	0.00133347	0.292261992
MVP	1.688759235	8.744798183	4.507835977	0.0001415	0.00133419	0.290811688
HAS2	-1.761385589	3.569051355	-4.501383624	0.0001438	0.00135019	0.274441291
RAP1GAP	-1.106118987	6.826981215	-4.498302213	0.000145	0.00135839	0.266624074
PARP3	1.149730973	6.52566565	4.496151133	0.0001458	0.00136401	0.261167277
DKK2	-1.068656153	3.078719612	-4.489612144	0.0001482	0.00138198	0.24458073
BSPRY	-1.420822934	5.661725718	-4.489157323	0.0001484	0.00138296	0.243427126
ZNF112	-1.28647625	4.91650117	-4.486682082	0.0001493	0.00138984	0.237149124
NBPF20	1.191657067	6.253680531	4.480711664	0.0001516	0.00140677	0.222007488
PARP12	1.397906822	7.212088348	4.480358869	0.0001518	0.0014074	0.221112816
C15orf48	4.114702838	6.912694864	4.479288172	0.0001522	0.00141062	0.218397617
GAPT	1.675670578	4.337093281	4.47868283	0.0001524	0.00141217	0.216862546
C6orf203	-1.022335762	6.602647041	-4.47787758	0.0001527	0.00141444	0.214820563
HACE1	-1.332181934	5.579830524	-4.471117685	0.0001554	0.00143717	0.197679856
CYP3A5	2.619673043	6.931814828	4.469107659	0.0001562	0.00144393	0.192583592
PLCL1	-2.05014876	4.761061024	-4.468368662	0.0001565	0.00144471	0.190709975
EFHD1	-2.211151495	8.308249846	-4.465264798	0.0001578	0.0014536	0.1828409
TMEM132E	-1.064580731	5.014725363	-4.464936735	0.0001579	0.0014538	0.182009206
DAGLB	1.020246663	5.965498123	4.46097681	0.0001595	0.00146371	0.171970603
GPNMB	2.62137014	7.955666822	4.459077892	0.0001603	0.00146952	0.167157045
PAPLN	1.784478264	6.975261774	4.452879987	0.0001628	0.00149034	0.151447347
C8orf4	-3.796033865	5.935700902	-4.45139603	0.0001634	0.00149468	0.1476863
LGR5	-1.146954896	3.879083527	-4.45052764	0.0001638	0.001496	0.145485445
RETSAT	1.132608873	7.906576678	4.446937901	0.0001653	0.00150743	0.136388015
LINC00597	-1.279637257	3.816220193	-4.446854006	0.0001654	0.00150743	0.136175408
IKBIP	1.59240592	5.851086616	4.444556702	0.0001663	0.00151497	0.130353762
CST3	1.041501186	7.145565992	4.440960141	0.0001679	0.00152558	0.121240224
BIRC3	2.466707402	6.710374727	4.438270413	0.000169	0.0015334	0.114425035
F2R	-1.164367515	5.197846954	-4.437644198	0.0001693	0.0015348	0.112838398
MTURN	-1.21258298	5.85414643	-4.436427982	0.0001698	0.00153724	0.109756946
LGALS3BP	1.538826115	10.42898459	4.435022223	0.0001704	0.00154005	0.106195364
TLR2	1.382249777	7.172474693	4.431621882	0.0001719	0.00155009	0.097580839
NEU1	1.520253259	9.68032903	4.42790001	0.0001736	0.00156079	0.088152492
COL4A3	-1.111720008	4.720515088	-4.420446355	0.0001769	0.00158735	0.069273096
MYO1F	1.118167389	6.534021071	4.413300989	0.0001802	0.00161099	0.051177618

EGR3	-1.830999004	5.014684757	-4.413051748	0.0001803	0.00161131	0.050546476
TYROBP	1.633015918	8.161218558	4.411087888	0.0001812	0.00161872	0.045573603
ENG	-1.12413838	6.286457799	-4.408931428	0.0001822	0.00162625	0.040113299
TRAM1L1	-1.250078544	6.12850867	-4.400394952	0.0001862	0.00165565	0.018501114
PMM2	1.103875044	7.060392543	4.398828889	0.000187	0.00166086	0.014536722
EML6	-1.228693083	4.74370093	-4.38993289	0.0001913	0.00168915	-0.007980056
LOC153684	1.302420866	7.239050483	4.388249472	0.0001921	0.00169392	-0.012240421
TET1	-1.921898765	4.322239452	-4.36698593	0.0002028	0.00177028	-0.06603818
ITGAM	2.050377761	6.584387452	4.360713791	0.0002061	0.00179234	-0.08190133
BAZ1A	1.40468007	6.841451401	4.358392148	0.0002073	0.00179964	-0.087772435
MICU3	-1.21359272	4.269420546	-4.355766617	0.0002087	0.00181099	-0.094411589
CTXN3	-2.535876873	4.148883599	-4.352614664	0.0002104	0.00182358	-0.102381286
NKD2	-1.108266652	5.494811825	-4.352108406	0.0002107	0.00182491	-0.103661292
HIBCH	-1.296920068	7.560691459	-4.351385173	0.0002111	0.00182675	-0.105489861
LIFR	-1.28451119	5.361986371	-4.336234034	0.0002194	0.00188534	-0.143788582
KIAA1919	1.279322455	5.769003832	4.333281345	0.0002211	0.00189644	-0.151250451
PKIB	1.422058695	4.203779271	4.331695016	0.000222	0.00190255	-0.155259074
MAP3K14	1.417153698	6.168942942	4.330028112	0.0002229	0.00190908	-0.159471116
NOD2	1.064301822	4.345398376	4.323519295	0.0002267	0.00193786	-0.175916111
SOWAHA	-1.877385541	4.923758007	-4.321457565	0.0002279	0.00194728	-0.18112458
TMEM170B	-1.182888873	4.733822749	-4.320565967	0.0002284	0.0019501	-0.183376893
NME1	1.622501843	9.882263549	4.320281862	0.0002286	0.0019507	-0.184094576
FERMT3	1.14410696	6.249808776	4.317389946	0.0002302	0.0019626	-0.191399538
IGSF11	-1.083325347	3.902930945	-4.313855435	0.0002323	0.00197724	-0.20032686
CSRP2	-1.793620015	7.749739732	-4.307223714	0.0002363	0.00200511	-0.217074481
CTSD	1.461744033	8.892785636	4.305562475	0.0002373	0.00201206	-0.221269227
KLF8	1.081220651	4.834354953	4.299795982	0.0002408	0.00203855	-0.235828411
CHAMP1	-1.112432688	7.388604524	-4.293568665	0.0002447	0.00206867	-0.251548233
DHRS2	-1.773805154	4.739573594	-4.289341011	0.0002474	0.00208597	-0.262218535
FDXR	1.298533525	6.25593332	4.279943315	0.0002534	0.00213314	-0.285932669
TRIM38	1.01851219	8.104518895	4.275979546	0.0002559	0.00214866	-0.295932749
COL12A1	-1.540935819	4.634018929	-4.274908815	0.0002566	0.00215366	-0.29863385
CRYAB	2.494128307	12.00686834	4.271600195	0.0002588	0.00216927	-0.306979829
SIM1	-1.983314445	5.763067649	-4.270337573	0.0002597	0.00217539	-0.310164554
TMEM158	-1.875460229	5.106031542	-4.269523278	0.0002602	0.00217903	-0.312218391
TLR7	1.06655087	4.925217697	4.269226357	0.0002604	0.00217979	-0.312967279
CORO2B	-1.711020748	5.41780454	-4.267415342	0.0002616	0.00218721	-0.317534837
HLA-DMB	1.343378353	9.439780341	4.265494119	0.0002629	0.00219618	-0.322380058
TMEM61	-1.442818987	5.706145868	-4.264807209	0.0002634	0.00219824	-0.324112335
HLA-DRA	2.181323831	10.93551393	4.25821132	0.0002678	0.00223376	-0.340744147
ESRP1	-2.3888199	5.318455086	-4.252770808	0.0002716	0.00225949	-0.354459927
ESPL1	-1.076555889	6.125810075	-4.249179091	0.0002741	0.00227433	-0.363513457
NUDT11	-2.032624755	4.708637454	-4.248983713	0.0002742	0.00227433	-0.364005909
PERP	1.051444759	6.425246866	4.234654661	0.0002844	0.00234077	-0.400113625

TEAD4	1.100295355	6.654013288	4.234097868	0.0002848	0.00234092	-0.401516333
ASB9	-1.48156443	5.087001822	-4.23332542	0.0002854	0.00234402	-0.403462284
KRT18	1.58580987	11.31665199	4.232616552	0.0002859	0.00234638	-0.40524802
SIGLEC10	1.165765224	5.093679504	4.230442052	0.0002875	0.00235661	-0.410725614
CD93	-1.445943634	6.699965391	-4.220170042	0.0002951	0.00240956	-0.436595363
TSPAN8	-4.032724862	6.276162001	-4.212439922	0.000301	0.00245365	-0.45605729
PON3	-1.255917417	5.079754361	-4.210610339	0.0003024	0.00245961	-0.460662801
LBX2-AS1	1.041126236	6.744207322	4.210555731	0.0003025	0.00245961	-0.460800259
KCNJ13	-1.13699502	3.537251035	-4.209803747	0.000303	0.00246238	-0.462693098
LOC101927809	1.047005927	4.078685565	4.199766	0.0003109	0.0025123	-0.487954469
SNCA	-1.171395472	5.680504608	-4.198860409	0.0003116	0.00251712	-0.49023306
SERTAD4-AS1	1.885148815	6.859204558	4.196657512	0.0003134	0.00252912	-0.495775539
HCP5	1.323641888	6.589660059	4.196532556	0.0003135	0.00252912	-0.496089914
MYO5C	-1.368913977	7.841236522	-4.19097308	0.000318	0.00255919	-0.510075473
PDE10A	-1.328702747	4.059890761	-4.190521303	0.0003183	0.00256114	-0.511211849
QPCT	2.39441676	7.069461881	4.187882371	0.0003205	0.0025754	-0.517849295
S100A10	1.477343773	9.388715981	4.186183577	0.0003219	0.00258557	-0.522121761
KLHL23	-1.443952819	5.738734083	-4.182562764	0.0003249	0.00260547	-0.531227196
SLC7A9	-2.454929775	6.002795442	-4.171044796	0.0003345	0.00266746	-0.560183724
EPAS1	-1.248598457	7.282059571	-4.160852421	0.0003433	0.00272601	-0.585797019
SEZ6L2	1.164942971	7.084891357	4.158905615	0.0003451	0.00273745	-0.590688164
STS	1.024774078	5.937977411	4.157441426	0.0003463	0.00274663	-0.594366538
PPAP2B	-1.494110763	8.92270233	-4.156432335	0.0003472	0.00275161	-0.59690148
MST1	-1.471203739	8.059755388	-4.145226827	0.0003573	0.00282038	-0.625044097
KBTBD7	-1.041459544	5.239054446	-4.143800683	0.0003586	0.00282956	-0.628624957
SPR	1.071656428	8.329989257	4.143305665	0.0003591	0.00282986	-0.629867831
TM6SF1	1.430611137	5.222814757	4.141078538	0.0003611	0.00284378	-0.63545933
C5	-1.64649894	5.179605128	-4.138898627	0.0003631	0.00285742	-0.640931799
HBM	-1.283400961	4.22949897	-4.124870334	0.0003763	0.00293769	-0.676136948
FCGR1B	2.522622913	6.379841105	4.124613793	0.0003766	0.00293849	-0.676780569
ITM2A	-2.241825259	6.85565905	-4.12309791	0.000378	0.0029468	-0.680583545
KIAA1462	-1.749551125	4.059667412	-4.111528665	0.0003893	0.00302311	-0.709599998
CPEB4	-1.199024196	6.494360378	-4.109416743	0.0003914	0.00303826	-0.714895316
GBP2	1.828605453	7.129320163	4.108669806	0.0003922	0.00304059	-0.716768032
PPP1R36	-1.36609657	5.637365274	-4.100697145	0.0004002	0.00309124	-0.736753321
SPAG4	1.530009344	6.253382446	4.100477545	0.0004004	0.0030918	-0.737303699
PLA2G16	1.621990444	7.878618429	4.099579109	0.0004014	0.00309766	-0.739555385
CYP4F2	-1.857795227	4.981286958	-4.097657524	0.0004033	0.00310822	-0.744371019
EDNRA	-2.397233631	5.150846653	-4.095545501	0.0004055	0.00312263	-0.749663445
ZNF300P1	-1.771304771	4.669805143	-4.094687068	0.0004064	0.00312781	-0.751814416
SLC6A20	2.172488836	5.893088794	4.094599651	0.0004065	0.00312781	-0.752033451
MOB1B	-1.404903988	7.709806614	-4.09400251	0.0004071	0.00313139	-0.753529653
TIMD4	1.095134823	4.773407052	4.082305184	0.0004194	0.00320795	-0.782830654
CXCL6	4.027262711	6.130702351	4.081074225	0.0004207	0.00321562	-0.785913244

EIF4E3	1.042579935	6.34245623	4.078356231	0.0004236	0.00323395	-0.792719097
ZNF14	-1.074529678	6.148280932	-4.076205785	0.000426	0.00324753	-0.798103226
RBP1	-1.24541362	5.519839333	-4.075255324	0.000427	0.00325266	-0.800482755
SLC25A25	-1.025522828	6.515217606	-4.073879765	0.0004285	0.00325995	-0.80392636
HOXA11	-1.100997911	5.259655455	-4.073353987	0.0004291	0.00325995	-0.805242547
TAF5	-1.308475033	4.533364107	-4.062356615	0.0004412	0.00334006	-0.832765277
WFDC1	-1.2667431	6.569900621	-4.060170626	0.0004437	0.00335002	-0.838234433
WISP1	-1.624057804	4.507572823	-4.056751024	0.0004476	0.0033743	-0.84678888
DKK1	-2.188010932	3.74832031	-4.053590487	0.0004512	0.00339654	-0.854694053
FPGT	-1.069876184	5.596678302	-4.049350203	0.0004561	0.0034233	-0.865298085
DUSP10	1.150591922	6.292946313	4.046973059	0.0004588	0.00343716	-0.871241889
ZNF542P	-1.090294928	5.3694536	-4.045024925	0.0004611	0.00344978	-0.876112508
LOC101929480	-1.401338512	4.828449242	-4.039613644	0.0004675	0.00349246	-0.889639152
SPAG5	-1.368132125	6.473229402	-4.038381077	0.000469	0.00350078	-0.892719733
NUAK1	-1.561909608	6.925283654	-4.037880593	0.0004696	0.00350154	-0.893970552
LOC101927365	-1.365570608	4.566789541	-4.035907715	0.0004719	0.00351516	-0.898900914
FYB	1.247999892	5.265785655	4.033210799	0.0004752	0.0035277	-0.905639947
S100A16	1.006276774	10.27373853	4.029194981	0.0004801	0.00355893	-0.915673037
FGD5	-1.308287126	5.849239966	-4.028324623	0.0004811	0.00356192	-0.917847278
DMGDH	-1.144498794	4.833301758	-4.027410528	0.0004822	0.00356736	-0.920130679
PLCB1	-1.295256998	5.10028984	-4.01994629	0.0004915	0.00361999	-0.938772511
ADORA3	1.127840647	5.096707266	4.015435222	0.0004971	0.00365646	-0.950035555
LOC102723721	1.327337601	5.347556719	4.01445119	0.0004984	0.00366298	-0.952492117
LRRK2	2.685657396	8.83905308	4.012992855	0.0005002	0.00366737	-0.956132518
2019 3 1	1.025690954	4.388510841	4.011467213	0.0005022	0.00367897	-0.959940658
RTP4	2.182947807	7.248512615	4.009644366	0.0005045	0.00369077	-0.964490269
MPZL2	1.388873824	5.875742779	4.007481163	0.0005073	0.00370185	-0.96988884
TMEM176B	1.241185624	8.132064996	4.005477925	0.0005099	0.00371676	-0.974887677
PTPRC	1.290556969	5.071342805	4.002104207	0.0005143	0.0037418	-0.983305253
ABCC3	1.253574354	7.242594678	3.995456837	0.000523	0.00378964	-0.999886578
C7orf31	1.122093872	5.888416971	3.994781548	0.0005239	0.00379314	-1.001570719
ITGB8	1.625098014	6.51513306	3.984636076	0.0005376	0.00387463	-1.026866154
STMN2	-1.844565503	3.764395174	-3.979431861	0.0005447	0.00392063	-1.039836586
KCNJ3	-1.095720645	4.631107862	-3.975810369	0.0005498	0.00394712	-1.048860352
CNDP1	-1.257370225	4.505103541	-3.974761774	0.0005512	0.00395487	-1.051472846
HRH1	1.49377842	6.243041045	3.973234667	0.0005534	0.00396467	-1.055277261
MEOX1	-1.393494837	3.533153491	-3.963280523	0.0005675	0.00404345	-1.080068139
C1orf162	1.501173699	6.699876988	3.963012931	0.0005679	0.00404478	-1.080734401
VASN	-1.121350747	6.859835846	-3.961828894	0.0005696	0.00405554	-1.083682347
ALDOA	1.07601347	10.61358857	3.95475142	0.0005799	0.00411327	-1.101299566
SLC16A6	1.586804042	5.06305877	3.948814626	0.0005887	0.00416749	-1.116072267
BCL2A1	2.867218777	5.622748713	3.948778798	0.0005888	0.00416749	-1.116161403
PRELP	-1.381610396	6.154803624	-3.948273904	0.0005895	0.00417129	-1.117417527
ZFPM2	-2.191375615	4.415835204	-3.946387819	0.0005924	0.0041855	-1.122109602

PCDHB2	2.370127322	5.838706839	3.941806863	0.0005993	0.00422565	-1.133503796
LOC340340	2.047533258	4.656689507	3.938807082	0.0006038	0.00424767	-1.140963597
MALT1	1.293242237	7.221365256	3.937634702	0.0006056	0.00425885	-1.14387872
WBSCR27	1.189606821	6.504821966	3.936879432	0.0006068	0.00426356	-1.145756599
CFI	1.699785633	9.470717317	3.936792714	0.0006069	0.00426356	-1.145972207
AP1S3	1.53447886	4.742665805	3.934264547	0.0006108	0.00428215	-1.152257562
NT5E	1.78593758	6.23076013	3.933807545	0.0006115	0.00428418	-1.153393637
S100P	-1.590433551	4.369000931	-3.925791826	0.0006241	0.00435868	-1.173315464
NEDD4L	-1.197542099	7.056066158	-3.92262268	0.0006291	0.00438784	-1.181189435
SAMHD1	1.132267584	6.095864144	3.921999633	0.0006301	0.00439178	-1.182737277
LOC285812	-1.007135043	4.442671929	-3.921461941	0.000631	0.00439627	-1.184073026
FAM107A	-1.808638308	6.120005972	-3.915604654	0.0006404	0.00445744	-1.198621248
RNFT1	1.171162174	4.908195573	3.912454477	0.0006455	0.00448988	-1.206443612
ABCA17P	1.133005906	4.220523738	3.91106389	0.0006478	0.00450135	-1.209896205
OMD	-1.124729533	3.485333121	-3.910739149	0.0006484	0.00450352	-1.210702443
PLA2G7	2.353478038	4.922519539	3.910338166	0.000649	0.00450657	-1.211697948
CWH43	-1.79754233	4.285672616	-3.905099555	0.0006577	0.00455748	-1.224701539
KMO	-1.947113568	5.19292916	-3.903161609	0.0006609	0.00457835	-1.229511035
POU5F1P4	1.092429175	5.728239883	3.898216175	0.0006692	0.00462348	-1.241781933
C12orf75	1.444053774	9.66442856	3.896801046	0.0006716	0.00463699	-1.245292588
MS4A7	1.459875508	6.487950856	3.89076029	0.000682	0.00468946	-1.260275265
NAPSB	1.159395678	5.614914084	3.888824429	0.0006853	0.00470933	-1.265075597
SLC22A12	-1.998296346	6.045304364	-3.885889934	0.0006904	0.00473649	-1.27235119
LOC101928047	-1.954463858	4.326574424	-3.885300108	0.0006915	0.0047406	-1.273813413
KRT222	1.066186405	4.035809005	3.884039636	0.0006937	0.00475076	-1.276938051
CDC42EP5	-2.176616138	4.937484864	-3.883031246	0.0006955	0.00475972	-1.279437626
PDGFRL	-1.331428829	4.697410145	-3.881209223	0.0006987	0.00477692	-1.283953637
LINC01268	2.258207627	5.288449669	3.880345929	0.0007002	0.00478417	-1.2860932
DEPDC1B	-1.509643173	3.353063708	-3.880178281	0.0007005	0.0047846	-1.286508683
TNFAIP6	3.924444245	6.928130571	3.877513802	0.0007052	0.00481375	-1.293111498
ZNF439	-1.360031689	4.676470309	-3.876108549	0.0007077	0.00482446	-1.296593419
SULT1E1	-2.890997088	3.553883141	-3.875101167	0.0007096	0.00483516	-1.299089319
SRXN1	1.166238793	8.708922546	3.872092003	0.000715	0.00486563	-1.306543962
ERMP1	-1.110196814	7.746872709	-3.86623332	0.0007256	0.00493007	-1.321053901
LINC00113	-1.149867168	4.132120318	-3.863076246	0.0007315	0.00495695	-1.328870759
WIPI1	1.390270578	8.382780611	3.859365326	0.0007384	0.00498829	-1.338057017
PRTG	-1.234252264	4.497360816	-3.857096758	0.0007426	0.00501367	-1.34367176
COL2A1	-1.706849051	3.544747016	-3.854538312	0.0007474	0.00504288	-1.350003021
HLA-DMA	1.768691792	8.518462816	3.854060141	0.0007483	0.00504565	-1.351186217
CHST9	2.049960857	5.955291559	3.853698052	0.000749	0.0050475	-1.352082155
FPR3	1.677166848	5.420818263	3.85298943	0.0007504	0.00505267	-1.353835481
ECHDC3	-2.077551456	7.028969697	-3.849445708	0.0007571	0.00508976	-1.362602479
CCDC109B	1.289488547	6.683189018	3.848420624	0.0007591	0.00509794	-1.365138132
UGT8	-2.039865341	7.152381445	-3.847629595	0.0007606	0.00510647	-1.367094716

TWIST1	-1.975980364	3.84757047	-3.842867599	0.0007698	0.00514803	-1.37887132
PYGL	1.383446897	7.076838857	3.842230495	0.000771	0.00515296	-1.380446641
FAT4	-2.071941961	5.795116194	-3.837509818	0.0007803	0.0052012	-1.392117154
DNMBP	-1.062077216	7.560962865	-3.833302727	0.0007886	0.00524686	-1.402515065
MAGI2-AS3	-1.191421708	5.278505397	-3.829053963	0.0007971	0.00528285	-1.413013175
CYB5R2	-1.275555402	5.188178934	-3.826504258	0.0008023	0.00530806	-1.41931179
EZR	1.542408075	8.948277303	3.825494121	0.0008043	0.00531989	-1.42180688
EVI2A	1.458849137	6.422419493	3.82380841	0.0008078	0.00533436	-1.425970315
THSD7A	-2.079743392	7.169498638	-3.823416834	0.0008086	0.00533581	-1.42693738
MIR10A	-1.111243088	5.987793131	-3.819281292	0.000817	0.00537276	-1.437149351
CHRNB1	1.045844199	5.4316436	3.796159245	0.0008661	0.0056358	-1.494194753
LHFP	-1.411625384	6.512837308	-3.794875342	0.0008689	0.00565049	-1.497359796
EXTL2	-1.437355545	6.969762447	-3.792085445	0.0008751	0.00568362	-1.504236449
LOC100505501	-1.265906303	4.189700959	-3.789962672	0.0008798	0.00571004	-1.509467894
CALCRL	-1.2657636	4.685073288	-3.787835245	0.0008845	0.00573559	-1.514710065
RPS6KA2	-1.033439054	6.459808785	-3.783596813	0.000894	0.00578774	-1.525151725
CHEK2	1.012763024	5.775230669	3.767978299	0.0009299	0.00598223	-1.563603229
EFEMP1	-1.777292993	5.314655004	-3.767781922	0.0009303	0.00598266	-1.564086435
CENPH	1.296061219	5.205767305	3.759161888	0.0009507	0.0060772	-1.58529043
CRNDE	1.780351476	7.575849454	3.758660077	0.0009519	0.00608225	-1.58652442
CIB1	1.020484369	9.301866883	3.753566349	0.0009642	0.00614159	-1.599047862
NRTN	1.281829356	4.751844811	3.753422106	0.0009646	0.00614191	-1.599402433
DOK6	-1.278393202	4.236330904	-3.748905662	0.0009756	0.00618713	-1.610502739
PTGFRN	1.261558384	7.913395652	3.746704607	0.000981	0.00620804	-1.615911116
CYTIP	1.688811199	5.575508418	3.746134169	0.0009824	0.00621312	-1.617312648
NAP1L3	-1.513359368	4.956057628	-3.745987798	0.0009828	0.00621349	-1.617672263
IL17D	-1.132977098	3.909364372	-3.742135151	0.0009924	0.00626049	-1.627136402
FBN2	-1.163167487	3.497383964	-3.735755996	0.0010084	0.00634029	-1.642801319
NDRG2	-1.049962489	6.479239757	-3.734701344	0.0010111	0.00635323	-1.645390481
ARSE	1.322602215	8.031585404	3.734283071	0.0010122	0.00635712	-1.646417284
HOXA1	1.197499989	6.39724372	3.734214169	0.0010123	0.00635712	-1.646586426
C2CD4A	1.269420588	4.076645168	3.732514957	0.0010167	0.00638044	-1.650757426
ERV3-2	2.116419634	7.436329917	3.732253079	0.0010174	0.00638268	-1.651400204
COL16A1	-1.159174263	5.939135622	-3.728909899	0.0010259	0.0064346	-1.659604965
CORO1A	1.167362373	6.54846964	3.720894188	0.0010468	0.00654155	-1.679268922
FCGR3B	1.945012585	6.133358315	3.719761613	0.0010498	0.0065582	-1.68204641
RECQL	1.46429645	7.386349833	3.716373489	0.0010588	0.00660421	-1.690353966
ACOX2	-2.079396027	6.473478712	-3.715644654	0.0010607	0.0066143	-1.692140776
DDX39A	1.063972901	8.663426719	3.711321467	0.0010723	0.00667436	-1.702737534
SH3BP5	-1.556423309	7.488028755	-3.709421407	0.0010774	0.00670343	-1.707393794
C15orf52	1.091687768	6.288829251	3.709349679	0.0010776	0.00670343	-1.707569557
IGFBP6	2.595131535	9.32988001	3.704721805	0.0010902	0.00676849	-1.71890778
MAGI2	-1.001500902	5.155177066	-3.698673483	0.0011069	0.00685232	-1.733720235
TRIM71	-1.913481632	4.093780342	-3.68966274	0.0011323	0.00698724	-1.755775343

C1QC	2.173781771	8.535384395	3.68942958	0.0011329	0.00698786	-1.75634584
C11orf96	-2.363972249	7.722996818	-3.688015154	0.0011369	0.00700638	-1.75980644
EOMES	1.06487614	4.35304387	3.687253403	0.0011391	0.00701556	-1.761670023
CNTN3	-2.532923815	5.189207458	-3.686073496	0.0011425	0.00703425	-1.764556389
GALNT3	-1.3133732	4.674244384	-3.685849268	0.0011431	0.0070361	-1.765104884
TMEM54	1.3973952	7.011022298	3.681908879	0.0011545	0.0070826	-1.774742099
SLC16A14	-1.587632959	4.704464816	-3.666633868	0.0011996	0.00730995	-1.812073651
DPY19L2P2	1.733758913	5.666285839	3.666574664	0.0011998	0.00730995	-1.812218257
SLC5A11	-1.012201808	5.103047235	-3.664861798	0.0012049	0.00733486	-1.816401687
CLIP3	-1.156859136	5.513958554	-3.664648552	0.0012056	0.0073366	-1.816922471
RABGEF1	1.220585184	6.310607744	3.653311229	0.0012403	0.00753239	-1.844597762
DUSP23	1.407819735	8.384827753	3.652559939	0.0012427	0.00753987	-1.846430855
NPY1R	-2.006480185	7.183109945	-3.648173302	0.0012564	0.00760741	-1.857131762
PDE1A	-2.796837548	4.676258626	-3.647220039	0.0012594	0.00762334	-1.859456693
BEX5	-1.798334422	6.779557649	-3.644945708	0.0012666	0.00766236	-1.865002897
PCDH7	-1.119182208	3.925501119	-3.643831482	0.0012701	0.00767924	-1.867719694
CEACAM1	-1.091645759	5.310700895	-3.638094478	0.0012885	0.00777657	-1.881704318
RBPMS-AS1	1.689147356	6.933436542	3.635209769	0.0012978	0.00781681	-1.888733718
CDK5RAP3	1.139603847	9.006539461	3.634106976	0.0013014	0.00783612	-1.891420555
TENM4	-1.83765154	5.185086574	-3.632502697	0.0013067	0.00785621	-1.895328782
C1QB	2.102890747	8.095275065	3.628695293	0.0013192	0.00790941	-1.904602082
CETP	-1.29848912	4.479429168	-3.624424304	0.0013333	0.00796741	-1.915001099
TAGLN	-1.86764509	7.697690079	-3.624225436	0.001334	0.00796905	-1.915485216
MT1HL1	-1.730623441	9.423306303	-3.613338959	0.0013708	0.00814625	-1.941974885
DACT1	-1.746179296	5.425367484	-3.612328854	0.0013743	0.00815975	-1.944431542
ERICH5	1.330180514	5.297014233	3.609454202	0.0013842	0.00821146	-1.951421808
HPD	-3.230298533	5.111024097	-3.607197506	0.001392	0.00824835	-1.956908235
ISLR	-1.855240951	5.49593708	-3.604919886	0.0014	0.00828346	-1.962444494
CDO1	-1.007613226	4.003026221	-3.602974903	0.0014068	0.00831902	-1.967171377
GABRA2	-1.316918154	3.48480118	-3.600071755	0.0014171	0.00836992	-1.974225467
PLA2G12B	-1.325753997	4.668283332	-3.593546885	0.0014403	0.00847813	-1.990073399
ACVR2A	-1.159833191	6.851907789	-3.592823567	0.0014429	0.00848858	-1.991829696
ZNF184	-1.021531362	5.89032961	-3.587884763	0.0014608	0.00857161	-2.003818813
MEF2C	-1.133177966	6.360975655	-3.584213091	0.0014743	0.00864327	-2.012728681
UGT3A1	-1.966321443	4.770602147	-3.58131221	0.001485	0.00869913	-2.019766148
BHMT	-3.117167015	8.232118187	-3.573528953	0.0015141	0.00884645	-2.038639535
TNFSF13	1.944163611	9.162356803	3.571770691	0.0015208	0.00888028	-2.04290135
IFI27	2.939794174	9.259013547	3.568236224	0.0015343	0.00894122	-2.051466522
EPS8	1.165388124	10.24419383	3.568229748	0.0015343	0.00894122	-2.051482213
PPP1R1A	-1.807534057	7.162358958	-3.56570161	0.001544	0.00898548	-2.057607112
VSTM2L	1.565290351	6.713473308	3.564432317	0.0015489	0.00900447	-2.060681712
CLMN	-1.166777344	6.270641968	-3.559250525	0.001569	0.00910223	-2.073230016
FERMT2	-1.030649414	7.629093578	-3.552809165	0.0015944	0.00924169	-2.0888206
CADPS2	-1.086008728	7.663581403	-3.549829521	0.0016063	0.00929741	-2.09602951

HEY1	-1.413037954	6.506463076	-3.545092181	0.0016254	0.00938921	-2.107487066
DPP10	-1.19013246	3.128960937	-3.544480409	0.0016279	0.00939558	-2.108966327
HLA-DRB1 HLA-DRB1	1.490620204	11.14390006	3.543481109	0.0016319	0.00941634	-2.111382454
OAS1	1.285038905	6.851914371	3.538746629	0.0016513	0.00950393	-2.12282665
KCNN2	-1.454154131	4.096715047	-3.538215587	0.0016534	0.00951383	-2.124109984
MT1E	-1.906801564	8.785183761	-3.536939328	0.0016587	0.00953951	-2.127193987
IL7	1.144607119	4.331323957	3.535314396	0.0016654	0.00957039	-2.13112003
MAN2B1	1.058745866	7.824520267	3.53194371	0.0016795	0.00963647	-2.139262213
KLRG2	-1.051989353	5.276130376	-3.531615004	0.0016808	0.00964165	-2.140056098
A1CF	-2.355458408	5.379621695	-3.530022845	0.0016875	0.00967452	-2.143901127
CPXM1	-2.252854006	4.990634554	-3.529386516	0.0016902	0.00968172	-2.145437691
TGFB1I1	-3.028272863	6.226388342	-3.528013186	0.001696	0.00970196	-2.148753619
TMEM140	1.371400362	8.305664251	3.526484816	0.0017024	0.00973011	-2.152443407
PEG10	-1.496334893	6.749018394	-3.525360502	0.0017072	0.00974849	-2.1551574
CNTN6	2.790886562	6.112643988	3.525166083	0.001708	0.00974849	-2.155626681
SNAP25	1.009692491	5.817055895	3.524737327	0.0017099	0.00975346	-2.15666157
SH3BGRL2	-1.293241662	6.600573715	-3.524061484	0.0017127	0.00976443	-2.158292768
CYP8B1	-1.951599548	4.940305068	-3.515828856	0.0017482	0.00992297	-2.178154803
SRPX	-2.577669552	5.136759872	-3.515799389	0.0017483	0.00992297	-2.178225868
SULT1C4	2.745358482	6.615856136	3.513313911	0.0017591	0.00997067	-2.184219383
NID1	-1.009220793	6.401141464	-3.513059433	0.0017602	0.00997422	-2.184832957
SPINK13	1.102188994	4.429135251	3.512369139	0.0017633	0.00998583	-2.18649726
UPP1	1.728618422	7.119452182	3.511579515	0.0017667	0.00999989	-2.188400919
FYN	-1.815747088	6.683200551	-3.509081589	0.0017777	0.01005387	-2.194422115
AGXT2	-2.310082022	7.029031964	-3.497751662	0.0018285	0.01028561	-2.221715201
LST1	1.06637504	7.078961335	3.489193168	0.0018678	0.01047028	-2.242312984
BTBD11	-1.715053732	4.756636929	-3.488045317	0.0018731	0.01048606	-2.245074266
SOX9	2.020095897	9.304903441	3.485594474	0.0018845	0.01053277	-2.250969038
CTGF	-2.039183547	8.550936492	-3.482470359	0.0018992	0.01060896	-2.258481186
DNER	-3.034448001	4.79470109	-3.478635796	0.0019173	0.01068416	-2.267698611
MT1X	-1.938840592	8.30737009	-3.47337203	0.0019425	0.01079935	-2.280346038
MRVI1	-1.109107188	5.425516973	-3.466221194	0.0019773	0.01094082	-2.297517377
MPP5	-1.097876956	7.671242046	-3.458431246	0.0020158	0.01110908	-2.316209941
PCK1	-3.614512763	6.658795329	-3.453311509	0.0020416	0.01121466	-2.328487432
TMEM176A	1.646569892	11.23123653	3.447822153	0.0020695	0.01133169	-2.341644464
PCOLCE	-1.345522083	6.84269688	-3.442340183	0.0020978	0.01145589	-2.354776707
NAGS	-1.070731486	5.532216768	-3.441922062	0.0021	0.0114647	-2.355778039
ZDHHC23	1.005025582	6.101671584	3.440610963	0.0021068	0.0114989	-2.358917638
AKR7A3	-1.420089643	7.534584689	-3.437961216	0.0021206	0.01155607	-2.365261556
LOC400043	-1.417449286	8.643236939	-3.434329793	0.0021398	0.01164177	-2.373953052
SLAMF8	1.396257068	5.222884173	3.432768714	0.0021481	0.01167132	-2.377688393
C17orf62	1.059785164	7.218478284	3.429418242	0.0021659	0.01175596	-2.385703412
SPARCL1	-1.959193873	7.823971607	-3.424649776	0.0021916	0.01186395	-2.397105923

MT1H	-1.861081614	9.328333364	-3.42352725	0.0021977	0.01189063	-2.399789349
BNC1	1.114884629	3.950482614	3.420515613	0.0022141	0.01194792	-2.406987235
IL2RG	1.580432625	6.259462316	3.418013865	0.0022279	0.01200169	-2.412964807
PPAP2A	-1.034420782	8.819793268	-3.417175686	0.0022325	0.01200898	-2.414967179
TRIM47	1.130393155	7.82780831	3.414873461	0.0022452	0.01206481	-2.42046621
PPAP2C	2.147142912	6.060746493	3.414136438	0.0022493	0.01207796	-2.42222637
LOC100130232	-2.136982633	5.205244628	-3.413353914	0.0022537	0.01209115	-2.424095051
PBX1	-1.191167847	7.860173839	-3.412727626	0.0022572	0.0121067	-2.425590533
KIF23	-1.636136425	4.134968189	-3.409959446	0.0022726	0.012177	-2.432199376
LGALS3	1.161628922	7.697473694	3.402786197	0.0023132	0.01234937	-2.449316308
APOM	-1.851723997	7.321810919	-3.401956822	0.002318	0.01236501	-2.451294562
LINC01279	-2.148700944	4.780242605	-3.396050276	0.002352	0.01253025	-2.46537816
GMNN	-1.321146991	7.773221239	-3.394187225	0.0023629	0.01256511	-2.469818641
SCD	1.024580561	6.599169533	3.392965637	0.00237	0.01259322	-2.472729763
SNHG19	-1.402388232	7.890318656	-3.392574878	0.0023723	0.0126021	-2.473660888
ZNF667	-1.269949495	4.48912657	-3.392203225	0.0023745	0.01261038	-2.474546449
RAB31	1.35214159	9.184043517	3.385662619	0.0024131	0.01279553	-2.490125576
NEFL	2.156384295	7.974529763	3.385066995	0.0024166	0.01280296	-2.491543767
ALDH6A1	-1.40098766	7.669006468	-3.37002133	0.0025079	0.01318569	-2.527338239
TMEM159	1.027793737	6.39711127	3.366693759	0.0025285	0.01326354	-2.535246994
PDK4	-1.41380235	5.489531519	-3.356730522	0.0025913	0.0135339	-2.55891011
UNC5CL	2.061348298	6.965180938	3.356156186	0.002595	0.01354268	-2.560273407
DIRAS3	-1.159285761	4.516542841	-3.354825386	0.0026035	0.01357326	-2.563431995
PKIA	-1.469382731	5.388112112	-3.354610023	0.0026048	0.01357569	-2.563943107
DYRK2	1.038677113	7.019414674	3.354036654	0.0026085	0.01358579	-2.565303797
CD52	1.33086693	6.303255315	3.352855141	0.0026161	0.01361841	-2.56810744
FOLR3	-1.003136418	5.127085888	-3.340812621	0.0026947	0.01392503	-2.596662874
C1orf116	-1.034973103	5.767331891	-3.337738972	0.0027152	0.01399178	-2.603945134
COLEC11	-1.075052918	6.48418582	-3.329905081	0.0027679	0.01422069	-2.622494464
C1orf53	1.328254101	5.379272103	3.328998627	0.0027741	0.01424166	-2.624639755
SYNDIG1	-1.713612423	5.270087432	-3.324840883	0.0028025	0.01435082	-2.634477052
GSAP	1.404363963	7.679266173	3.323286352	0.0028133	0.01438318	-2.638153931
PRKAR2B	-1.67598604	7.025042576	-3.322240637	0.0028205	0.01440247	-2.640626965
RASSF6	1.1521444	5.647187307	3.322176759	0.0028209	0.01440247	-2.640778021
AKR1C1	1.275034682	7.298581549	3.322118615	0.0028213	0.01440247	-2.640915519
IRF8	1.049864323	7.61058994	3.321720118	0.0028241	0.01441171	-2.641857843
SLC25A33	-1.432437531	6.868455837	-3.313809123	0.0028794	0.01464299	-2.660556237
SLC25A43	1.344662501	6.571002942	3.311764361	0.0028939	0.0146948	-2.665386519
MRPL10	1.050134789	8.805972736	3.308500385	0.0029172	0.01479088	-2.6730946
VEPH1	-1.041038666	5.806724185	-3.304834515	0.0029435	0.01489536	-2.681748375
AZGP1	-2.894943819	5.615867837	-3.277090378	0.0031504	0.01574343	-2.747124211
RRAD	1.48829766	6.086100797	3.276792965	0.0031527	0.0157472	-2.747823893
GIMAP6	-1.496175753	5.826288402	-3.275116215	0.0031656	0.01579656	-2.751768082
TMEM171	1.167670465	6.641795241	3.271431533	0.0031943	0.01591313	-2.760432768

IL1R2	-1.469371028	5.280936805	-3.250081728	0.0033653	0.01657063	-2.810563429
FXYD5	1.124848711	7.774897962	3.247353506	0.0033877	0.01667329	-2.816960255
GLT8D2	-1.792437316	4.720055105	-3.246410967	0.0033955	0.01669162	-2.819169726
RNASE6	1.419948105	6.959967103	3.239280794	0.0034551	0.0169397	-2.835875905
TMEM38B	-1.190370519	6.616543925	-3.238295135	0.0034634	0.01697233	-2.838184194
HLA-DQB1	1.352549151	5.903038511	3.236322913	0.0034801	0.01703782	-2.842802058
CDH11	-2.083523098	5.224630275	-3.234176821	0.0034983	0.01710265	-2.847825769
DPYS	-2.536348052	6.523656889	-3.232258097	0.0035147	0.01716641	-2.852316129
DPY19L3	-1.132759158	6.959074894	-3.232013644	0.0035168	0.01717255	-2.852888144
GLRB	1.503515982	5.903302325	3.225759676	0.0035708	0.01739057	-2.867516449
ACADSB	-1.079588922	6.472090671	-3.221133147	0.0036113	0.01752508	-2.878330863
FAM26F	1.217552404	5.134228745	3.214750061	0.0036678	0.01774062	-2.893241039
CST7	1.041927973	5.579690926	3.214143965	0.0036732	0.01775738	-2.894656198
FBN1	-1.838429924	5.563737236	-3.209377592	0.0037161	0.01793588	-2.905781358
ATRNL1	-1.120787332	4.382372746	-3.207219031	0.0037356	0.01799212	-2.910817463
KHK	-1.233827747	6.085327509	-3.191954495	0.0038768	0.01850235	-2.946391904
TAPBPL	1.10218061	7.098539409	3.191575446	0.0038804	0.0185148	-2.947274415
SOX11	-1.919366506	3.960524171	-3.190916778	0.0038866	0.01853608	-2.94880784
TMEM255A	1.476789589	4.533874759	3.186750351	0.0039261	0.01865982	-2.958504588
FBP1	-1.555977436	7.610828039	-3.182206649	0.0039696	0.01880081	-2.969073507
EPB41L5	-1.027161796	6.072594071	-3.179866932	0.0039922	0.01889036	-2.974513425
FBXO6	1.208090352	6.134400045	3.179187461	0.0039988	0.01889836	-2.976092911
PIGR	1.638844226	7.916411243	3.170291958	0.004086	0.01923189	-2.996758458
STMN3	1.02178579	6.022523011	3.166531061	0.0041234	0.01937236	-3.005488407
NIT2	1.007357085	9.787931376	3.159853983	0.0041906	0.01958044	-3.02097697
CD74	1.229611908	8.338601709	3.155183675	0.0042383	0.01976562	-3.031802453
DFNA5	1.438393604	9.283812782	3.147064461	0.0043224	0.02006227	-3.050606429
ANXA13	2.135083529	7.048310571	3.146611126	0.0043271	0.02007611	-3.051655751
ARMCX2	-1.113483901	9.125600472	-3.136952018	0.0044293	0.02045781	-3.07399836
BLNK	-1.420296088	7.314184895	-3.126727914	0.0045401	0.02087516	-3.097616382
LOC284825	1.819607338	7.623679315	3.126481972	0.0045428	0.02088287	-3.098184115
GSTO2	-1.154995729	6.421531942	-3.123955315	0.0045706	0.02099649	-3.104015564
OSR2	-1.311361636	3.992500393	-3.118674634	0.0046292	0.02122306	-3.116196757
EYA1	-1.603809266	4.751117206	-3.118578883	0.0046302	0.02122306	-3.11641755
SRGN	1.279589935	7.708148286	3.115472871	0.0046651	0.02134921	-3.123578148
SLC51B	-1.201937054	5.137263533	-3.112257364	0.0047014	0.02146264	-3.130987971
KDM5D	-2.073054533	4.961225783	-3.108879432	0.0047398	0.02161885	-3.13876857
PRSS8	-1.435295484	6.984865007	-3.106603629	0.0047659	0.02170783	-3.14400853
DAO	-1.799750914	5.493871766	-3.106528721	0.0047667	0.02170783	-3.144180973
PLAUR	1.156470804	6.578057147	3.099490181	0.0048482	0.02198199	-3.160376387
VCAN	2.717383841	9.815631135	3.097793755	0.0048681	0.0220371	-3.164277437
PDZK1IP1	1.90105573	10.65878474	3.090948988	0.0049489	0.02232374	-3.180008101
CLEC4A	1.162261597	4.689830794	3.09086633	0.0049499	0.02232374	-3.180197974
C1QA	1.793826943	8.31283304	3.089388482	0.0049676	0.02237862	-3.183592356

CLMP	-1.765348763	5.091173432	-3.082079015	0.0050557	0.02269561	-3.200370689
TNFSF10	2.022754192	9.715074602	3.07952595	0.0050868	0.0227804	-3.206226989
TRBC1	1.424960669	6.428801362	3.076099999	0.0051288	0.02293863	-3.214082219
KLF6	1.003778855	9.003407553	3.074143258	0.005153	0.02302158	-3.218567041
PRAME	1.390942583	5.392556155	3.073491662	0.0051611	0.02304256	-3.220060214
NPY6R	1.167106083	6.276118692	3.073285639	0.0051636	0.02304895	-3.220532299
PLA1A	1.711375482	6.684520478	3.071054811	0.0051914	0.02314755	-3.225643181
ZNF667-AS1	-2.316863532	6.237623229	-3.070726891	0.0051955	0.02316074	-3.226394319
PCK2	-1.23508921	7.579017236	-3.063154746	0.0052907	0.02349863	-3.24372934
TFPI	1.583847473	6.594263465	3.058810685	0.0053462	0.02367295	-3.253665769
TGFBR3	-2.19498602	7.492490147	-3.054477678	0.005402	0.02386506	-3.263570713
SPOCK2	-1.005055332	7.076661439	-3.05388839	0.0054097	0.02389204	-3.264917305
P2RY13	1.440604325	5.218284266	3.046782879	0.0055026	0.02419283	-3.281145158
PON2	1.231289653	8.291664146	3.039499691	0.0055994	0.02453424	-3.29776136
IRX3	1.330600974	9.512540985	3.038958374	0.0056066	0.02455615	-3.298995637
UBP1	-1.015306574	7.536704758	-3.028736478	0.0057454	0.02506348	-3.322284523
DCXR	-1.145919401	8.051738543	-3.027114227	0.0057678	0.02514301	-3.325977316
WDR72	-1.115007466	6.342297706	-3.022024278	0.0058384	0.02537517	-3.337557991
CERK	-1.036390159	6.789430136	-3.021369361	0.0058475	0.02540413	-3.339047424
AGT	1.735784179	9.310779299	3.020962148	0.0058532	0.0254111	-3.339973447
QRFPR	1.18583574	4.196775768	3.020351678	0.0058618	0.02543361	-3.341361581
ARNT2	-1.541367414	7.098144324	-3.017513868	0.0059016	0.02557412	-3.347812756
ANXA1	2.058294974	7.803821507	3.016921457	0.00591	0.02560491	-3.349159133
EPHA4	-1.001384856	5.298397383	-3.013316883	0.0059611	0.02576457	-3.357348703
CLEC5A	1.221559092	4.101746303	3.003196307	0.0061068	0.02625762	-3.380318834
C3AR1	1.357380599	7.942588886	3.00092801	0.0061399	0.02634474	-3.385462243
PRODH2	-1.109202454	6.62037739	-3.000379785	0.0061479	0.02636815	-3.386705088
IL7R	1.196822597	5.674475534	3.000152276	0.0061513	0.02637694	-3.387220828
SERPINB9P1	1.16387443	5.090629049	2.999931779	0.0061545	0.02638528	-3.387720656
HCK	1.224004618	6.35272781	2.999401043	0.0061623	0.02640709	-3.388923671
DNAJC12	-1.248552643	5.036858157	-2.997053177	0.0061969	0.0264925	-3.394244398
FSTL3	1.283018528	7.311210143	2.995412054	0.0062212	0.02657097	-3.397962374
ZCCHC24	-1.065441717	7.050484705	-2.994689453	0.0062319	0.02660567	-3.399599137
EPHX2	-1.539480701	7.55446015	-2.994086905	0.0062408	0.02662725	-3.400963829
CHRDL1	-1.940127427	4.812691504	-2.99367601	0.006247	0.02664223	-3.40189438
TMEM130	1.298577911	5.13673347	2.99149493	0.0062795	0.02673649	-3.406832877
P2RX5	1.164681415	5.108177288	2.989839338	0.0063043	0.02682287	-3.410580438
TNFSF13B	1.612873948	6.631290002	2.987648326	0.0063373	0.02693552	-3.415538498
ANKRD33B	1.291466835	6.535216523	2.982982137	0.0064081	0.02715412	-3.426092087
GALNT7	1.178520926	7.164830247	2.982663096	0.0064129	0.02716912	-3.42681339
GPR34	1.499634552	6.845488618	2.97573188	0.0065196	0.02750446	-3.442474996
APOL1	1.470820407	7.758496377	2.973548033	0.0065535	0.02761612	-3.447406069
ERAP2	1.273171363	4.619162212	2.969837869	0.0066116	0.02779785	-3.455779683
ZNF703	-1.078851623	5.488428732	-2.967466128	0.0066489	0.02792402	-3.461130012

SLC27A3	1.246871	7.372359282	2.95502804	0.0068482	0.02855379	-3.489156025
SLC46A3	1.556249514	7.577266602	2.952163918	0.0068949	0.02871215	-3.49560178
C1orf226	-1.062098928	5.753611646	-2.948248498	0.0069592	0.02893294	-3.504408754
ACOT4	-1.438270924	5.334728273	-2.944627286	0.0070192	0.02911147	-3.51254907
CCDC110	1.00406728	4.457578528	2.943484453	0.0070382	0.02917836	-3.515117128
LTF	-1.105188488	5.658055054	-2.941622803	0.0070694	0.02927797	-3.519299431
CXCL8	2.79430266	6.840588318	2.935107833	0.0071793	0.0295956	-3.533925836
SMIM24	-2.726497464	8.311235616	-2.929863608	0.007269	0.02987505	-3.545688195
LY96	1.897191474	7.037451205	2.928386172	0.0072944	0.02994961	-3.549000158
COL15A1	-2.011798501	6.769828259	-2.925205937	0.0073495	0.03011065	-3.556126581
CXCL1	2.243718237	6.407302748	2.925187909	0.0073498	0.03011065	-3.55616697
SUSD2	-1.303440871	6.034274895	-2.924922892	0.0073544	0.03011748	-3.556760663
OXCT1	-1.121110195	8.587488541	-2.923101999	0.0073861	0.03021723	-3.560839159
FLVCR1-AS1	1.024464188	6.175918865	2.919296754	0.0074529	0.03042342	-3.569358358
EBI3	1.002537072	5.155424808	2.91371911	0.0075517	0.03074121	-3.581836022
P2RY12	1.142485505	3.953498229	2.913210125	0.0075608	0.03076244	-3.582974096
PLS3	-1.404505954	9.434781875	-2.90518565	0.0077054	0.0312364	-3.600903928
HSPA6	2.281558218	6.432357772	2.902212077	0.0077597	0.0314072	-3.607542013
VTCN1	-1.954629515	5.57012491	-2.896267659	0.0078692	0.03176202	-3.620802253
KDELC1	1.480316403	7.532838902	2.892726108	0.0079351	0.0319904	-3.628696152
EXO1	-1.106106908	4.881565708	-2.884409047	0.0080921	0.03246979	-3.647215924
SCEL	2.311334825	4.533513487	2.877551905	0.0082237	0.03290124	-3.662465319
RPL22L1	1.19122242	8.85089381	2.876502455	0.008244	0.03296323	-3.664797595
COX7A1	-2.09213678	7.208233271	-2.870694013	0.0083574	0.03336433	-3.677698619
CSF1R	1.197805542	7.900512587	2.869901157	0.008373	0.03340702	-3.679458624
TMEM125	-1.26948365	6.483484084	-2.861556771	0.0085387	0.03388341	-3.697967241
NR4A2	-1.378088297	5.182179356	-2.852946458	0.0087129	0.03442817	-3.71703782
CCL18	2.80372037	6.435766557	2.849847384	0.0087765	0.0346124	-3.72389485
BEX1	-2.243214781	6.450773427	-2.849811435	0.0087772	0.0346124	-3.723974368
HAMP	1.218188387	6.46661909	2.84586721	0.0088587	0.03485633	-3.732695975
GDA	1.509061241	7.024346713	2.842234641	0.0089344	0.03508682	-3.740723121
LAT	1.087041465	5.789968421	2.838669223	0.0090093	0.03529682	-3.748596908
LOC102724356	-1.324476129	7.120469911	-2.838623178	0.0090103	0.03529682	-3.74869856
THY1	-1.937691095	6.967413999	-2.836009137	0.0090656	0.03545243	-3.754468166
LOC100132891	1.408178535	4.71236846	2.833452243	0.00912	0.03565156	-3.760109067
MNDA	1.620858172	5.718663969	2.833037459	0.0091289	0.03567065	-3.761023905
SHISA2	-2.607112833	5.963958154	-2.832396876	0.0091426	0.03569201	-3.762436626
DDC	-1.787186102	7.023477858	-2.824487755	0.0093132	0.03620639	-3.779865947
LCP1	1.203497262	7.639564195	2.824110092	0.0093214	0.03623063	-3.78069759
EIF1AY	-1.900607427	4.158234695	-2.823957836	0.0093247	0.03623063	-3.781032854
ELF3	1.346236704	8.088090197	2.822780425	0.0093504	0.03630978	-3.783625173
GJC1	-1.196948778	4.388508278	-2.81849754	0.0094444	0.03660547	-3.793050256
HCLS1	1.048664994	7.276965518	2.813621398	0.0095526	0.03691281	-3.803772087
ACADM	-1.007211197	9.157488545	-2.808478468	0.0096679	0.03723197	-3.815070355

ZNF516	-1.022732527	5.676115717	-2.807413861	0.0096919	0.03728948	-3.817407834
IFI16	1.102693388	8.2546438	2.804462693	0.0097588	0.03750469	-3.823885137
KLHL13	-1.556506212	7.555686229	-2.794656348	0.0099844	0.03817088	-3.84538345
PBLD	-1.436606818	7.533192	-2.794494334	0.0099881	0.03817816	-3.845738309
GALNT12	1.248632004	6.819260155	2.791701074	0.0100533	0.03834136	-3.851854715
CLDN3	1.685974881	8.951681958	2.789598745	0.0101026	0.03848641	-3.856456125
TREM1	1.091764429	5.607631917	2.787884929	0.010143	0.0385971	-3.860205874
ATP1A1	-1.094670673	10.57633673	-2.784285395	0.0102282	0.03884942	-3.868077636
MICB	1.467504198	5.055788457	2.760078216	0.0108195	0.04068866	-3.920879513
LOC100506119	-1.17175985	6.551126718	-2.757696177	0.0108794	0.0408307	-3.926062406
MATR3	1.115209945	4.560162924	2.7513557	0.0110404	0.04130614	-3.939846792
LINC01116	-1.572869101	6.215280459	-2.738481902	0.0113741	0.04221558	-3.967783706
L1CAM	-1.257836296	5.966054768	-2.73179111	0.0115513	0.04275704	-3.982275922
RBM20	1.949539988	5.536942748	2.726843824	0.011684	0.04312351	-3.992979681
KIT	-1.101339436	6.370435907	-2.722831737	0.0117927	0.04343065	-4.001652542
ARNTL2	1.101334897	4.959720308	2.719902889	0.0118726	0.04369695	-4.00797951
LOC100507642	1.236835175	5.853424458	2.719256891	0.0118903	0.04371942	-4.009374524
MYH8	-1.426520695	4.327144655	-2.714053582	0.0120337	0.04416759	-4.020604494
PLS1	1.475776922	9.256164554	2.708976372	0.0121753	0.04455138	-4.03155127
EGR2	-1.321871395	5.609499586	-2.705021655	0.0122866	0.04483063	-4.040070304
NAP1L2	-1.128826745	4.246977689	-2.686659775	0.0128162	0.04635037	-4.079536951
FAM110C	1.267451391	7.985169755	2.686073627	0.0128335	0.04638794	-4.080794421
BEX4	-1.129066373	8.15213013	-2.68113471	0.0129797	0.04681743	-4.091384058
BCHE	1.736051815	6.784984712	2.6766117	0.013115	0.04718085	-4.101072684
KRT8	1.529007144	9.172245434	2.673371596	0.0132127	0.04748243	-4.108007777
ANG	1.284314392	7.520344589	2.666990567	0.0134072	0.0480549	-4.121652325
PRUNE2	1.685045486	6.899414327	2.664886139	0.013472	0.04823356	-4.126148328
FZD1	1.102423028	7.010781419	2.664622969	0.0134801	0.04823356	-4.126710442
TUBB2B	-1.841597158	6.915697028	-2.660356529	0.0136123	0.04856888	-4.135819038
CD14	1.354143375	8.742051269	2.656284761	0.0137396	0.04890385	-4.144504577
SPRY2	-1.181386463	8.056991767	-2.655212109	0.0137733	0.04898132	-4.146791453
OXTR	1.080234306	5.121395385	2.654632669	0.0137916	0.04903771	-4.148026598
POSTN	-1.557695034	4.70533198	-2.64457537	0.0141119	0.04997732	-4.169441345

Table S3. DEGs of KIRP in TCGA database

Gene Symbol	Gene ID	Median (Tumor)	Median (Normal)	Log2(Fold Change)	adjp
A1CF	ENSG00000148584.14	0.37	1.96	-1.111	2.71E-08
A2M	ENSG00000175899.14	86.085	197.149	-1.186	5.28E-07
ABCA3	ENSG00000167972.13	18.96	6.745	1.366	1.04E-18
ABCB1	ENSG00000085563.14	63.195	28.444	1.124	2.00E-03
ABCC3	ENSG00000108846.15	46.741	9.465	2.19	5.40E-13
ABCC6P2	ENSG00000255277.3	12.58	5.597	1.042	4.82E-03
ABHD11	ENSG00000106077.18	100.558	32.99	1.579	3.02E-13
ABHD11-AS1	ENSG00000225969.1	4.84	0.63	1.841	1.68E-15
ABI3BP	ENSG00000154175.16	41.878	14.455	1.472	5.02E-06
ABO	ENSG00000175164.13	2.55	7.642	-1.284	7.61E-06
ABR	ENSG00000159842.14	47.23	21.695	1.088	1.83E-13
AC000123.3	ENSG00000279265.1	2.995	0.95	1.035	1.69E-07
AC005255.3	ENSG00000256210.3	3.995	1.18	1.196	8.76E-07
AC006126.4	ENSG00000267045.1	0.56	8.035	-2.534	5.41E-38
AC007283.5	ENSG00000234431.2	5.095	1.045	1.576	2.84E-15
AC009065.4	ENSG00000279473.1	3.175	0	2.062	3.15E-09
AC013271.3	ENSG00000186148.11	0.405	2.285	-1.225	6.33E-12
AC013275.2	ENSG00000231013.1	0.445	3.084	-1.499	8.21E-10
AC013463.2	ENSG00000236283.3	2.44	6.95	-1.209	1.88E-06
AC019117.1	ENSG00000236318.1	2.06	0.08	1.502	3.55E-07
AC019181.2	ENSG00000233255.1	0.815	3.53	-1.32	5.38E-08
AC025171.1	ENSG00000215068.7	2.025	0.47	1.041	1.37E-13
AC026471.6	ENSG00000260740.2	0.14	3.424	-1.956	7.58E-23
AC027612.6	ENSG00000143429.9	73.094	34.44	1.064	1.10E-06
AC069213.1	ENSG00000224769.1	0.92	5.208	-1.693	1.36E-19
AC074286.1	ENSG00000213963.6	1.5	9.66	-2.092	1.27E-25
AC079630.2	ENSG00000223914.1	28.494	2.275	3.171	1.24E-18
AC079630.4	ENSG00000225342.2	17.319	4.155	1.829	2.05E-14
AC079767.4	ENSG00000224137.1	1.18	0.065	1.033	1.26E-09
AC092580.4	ENSG00000235576.1	1.885	0.31	1.139	5.25E-05
AC093326.1	ENSG00000223985.1	0.1	9.219	-3.216	3.00E-34
AC093673.5	ENSG00000232533.1	17.775	5.385	1.556	3.80E-23
AC104534.3	ENSG00000268083.5	1.51	0	1.328	6.45E-06
AC108142.1	ENSG00000177822.7	1.91	0.395	1.061	6.08E-11
AC110615.1	ENSG00000279909.1	3.15	0.555	1.416	5.52E-04
AC113188.2	ENSG00000279535.1	10.045	3.64	1.251	1.93E-05
AC124789.1	ENSG00000276170.4	0.39	3.795	-1.786	6.99E-29
AC124944.5	ENSG00000223783.1	0	1.699	-1.432	3.45E-56
AC132217.4	ENSG00000240801.1	1.615	17.157	-2.796	1.35E-19
AC144831.1	ENSG00000261888.1	0.76	2.84	-1.126	2.06E-15
AC147651.5	ENSG00000229380.1	12.59	1.93	2.214	8.21E-08

AC234582.1	ENSG00000279487.1	1.59	10.399	-2.138	3.36E-08
AC245100.1	ENSG00000280649.1	10.83	4.74	1.043	4.61E-13
ACACB	ENSG00000076555.15	6.41	13.95	-1.013	3.25E-10
ACADSB	ENSG00000196177.12	9.19	22.95	-1.233	7.41E-11
ACE2	ENSG00000130234.10	39.084	11.054	1.734	2.04E-04
ACHE	ENSG00000087085.13	5.715	1.59	1.375	3.13E-06
ACOT11	ENSG00000162390.17	8.13	20.102	-1.209	1.03E-04
ACOT12	ENSG00000172497.8	0	1.305	-1.204	2.74E-32
ACPP	ENSG00000014257.15	0.275	14.762	-3.628	3.54E-64
ACRBP	ENSG00000111644.7	4.67	1.54	1.158	1.20E-13
ACSF2	ENSG00000167107.12	22.36	80.649	-1.805	2.29E-15
ACSL1	ENSG00000151726.13	50.676	124.012	-1.275	4.37E-05
ACSM1	ENSG00000166743.9	5.5	0.66	1.969	4.88E-16
ACSM5	ENSG00000183549.10	21.625	9.465	1.112	3.11E-06
ACTA2	ENSG00000107796.12	55.595	157.046	-1.482	8.10E-07
ACTG2	ENSG00000163017.13	0.52	2.4	-1.161	3.53E-04
ACVRL1	ENSG00000139567.12	5.73	14.865	-1.237	1.01E-11
ADAMTS4	ENSG00000158859.9	0.34	1.695	-1.008	1.25E-07
ADAMTS9	ENSG00000163638.13	26.549	10.325	1.283	2.00E-05
ADAMTSL1	ENSG00000178031.15	0.18	2.815	-1.693	4.02E-56
ADAMTSL2	ENSG00000197859.9	1.73	13.935	-2.452	6.76E-30
ADCK2	ENSG00000133597.9	28.59	12.074	1.178	8.36E-27
ADCY1	ENSG00000164742.14	0.09	1.46	-1.174	9.71E-24
ADCY2	ENSG00000078295.15	2.93	0.385	1.505	1.21E-10
ADCY4	ENSG00000129467.13	1.19	13.325	-2.709	2.70E-53
ADCY7	ENSG00000121281.12	4.02	1.225	1.174	6.41E-12
ADGRA2	ENSG00000020181.17	1.065	5.585	-1.673	4.87E-09
ADGRF1	ENSG00000153292.15	4.66	23.125	-2.092	1.80E-13
ADGRF5	ENSG00000069122.18	2.375	35.584	-3.438	1.87E-41
ADGRG6	ENSG00000112414.14	8.94	2.679	1.434	1.48E-07
ADGRL4	ENSG00000162618.12	2.19	10.828	-1.891	9.21E-17
ADGRV1	ENSG00000164199.15	0.11	1.46	-1.148	3.48E-47
ADH1B	ENSG00000196616.12	1.06	36.023	-4.168	3.70E-34
ADH1C	ENSG00000248144.5	0.14	9.94	-3.263	1.42E-47
ADH6	ENSG00000172955.17	1.605	8.79	-1.91	3.60E-10
ADM	ENSG00000148926.9	12.63	36.235	-1.45	6.65E-03
ADORA2A	ENSG00000128271.19	1.52	4.6	-1.152	2.66E-16
ADORA2A-AS1	ENSG00000178803.10	0.42	2.08	-1.117	2.56E-14
ADRA2B	ENSG00000274286.1	0.19	1.785	-1.227	7.40E-40
ADRA2C	ENSG00000184160.7	1.23	8.87	-2.146	1.12E-24
ADTRP	ENSG00000111863.12	0.37	3.789	-1.806	3.63E-34
AEN	ENSG00000181026.14	11.865	4.94	1.115	1.36E-24
AF127577.10	ENSG00000229047.1	20.955	6.565	1.537	8.52E-05
AF186192.5	ENSG00000255085.8	0.135	1.665	-1.231	1.59E-24

AFAP1L2	ENSG00000169129.14	0.955	16.435	-3.157	9.28E-36
AFM	ENSG00000079557.4	0.02	3.646	-2.187	4.74E-42
AFP	ENSG00000081051.7	0.3	1.945	-1.18	1.09E-13
AGMAT	ENSG00000116771.5	3.245	27.614	-2.753	1.04E-16
AGPAT9	ENSG00000138678.10	3.92	19.271	-2.043	1.67E-19
AGR2	ENSG00000106541.11	0.385	3.89	-1.82	8.10E-06
AGTR1	ENSG00000144891.17	0.295	5.4	-2.305	2.68E-42
AGXT	ENSG00000172482.4	0.21	5.295	-2.379	2.94E-39
AGXT2	ENSG00000113492.13	9.27	38.646	-1.949	1.80E-05
AHNAK2	ENSG00000185567.6	43.181	1.01	4.458	2.30E-41
AHR	ENSG00000106546.12	16.855	6.172	1.316	1.84E-09
AK8	ENSG00000165695.9	6.445	2.66	1.024	9.86E-14
AKAP2	ENSG00000241978.9	9.1	19.898	-1.049	1.86E-05
AKR1B1	ENSG00000085662.13	222.875	90.036	1.298	1.46E-06
AKR1C1	ENSG00000187134.12	217.338	43.142	2.306	3.49E-09
AKR1C2	ENSG00000151632.16	47.753	7.42	2.534	1.76E-13
AL035610.1	ENSG00000232079.6	22.554	6.51	1.649	3.14E-05
ALB	ENSG00000163631.16	0.42	33.065	-4.584	1.90E-50
ALDH1A1	ENSG00000165092.12	408.671	185.535	1.135	2.37E-11
ALDH1A2	ENSG00000128918.14	2.82	14.185	-1.991	7.69E-04
ALDH3B1	ENSG00000006534.15	41.215	15.395	1.365	6.65E-14
ALDH4A1	ENSG00000159423.16	37.349	106.788	-1.491	2.10E-06
ALDH6A1	ENSG00000119711.12	15.995	43.967	-1.404	3.44E-11
ALDOA	ENSG00000149925.16	2317.172	1076.624	1.105	9.76E-17
ALDOB	ENSG00000136872.17	1.35	982.867	-8.71	9.73E-47
ALOX15B	ENSG00000179593.15	1.235	0.065	1.069	6.26E-08
ALOX5	ENSG00000012779.10	33.579	3.467	2.953	2.00E-26
ALOX5AP	ENSG00000132965.9	18.5	7.019	1.282	8.48E-09
ALPL	ENSG00000162551.13	5.3	24.213	-2.001	1.12E-12
ALS2CL	ENSG00000178038.16	9.565	23.725	-1.227	4.05E-12
AMACR	ENSG00000242110.7	261.179	45.391	2.499	9.99E-16
AMICA1	ENSG00000160593.17	7.02	2.465	1.211	3.29E-06
AMIGO2	ENSG00000139211.6	1.255	6.23	-1.681	8.11E-09
AMPH	ENSG00000078053.16	0.09	1.41	-1.145	4.90E-20
ANG	ENSG00000214274.9	27.225	10.524	1.292	1.74E-05
ANGPT1	ENSG00000154188.9	0.31	3.239	-1.694	3.26E-23
ANGPTL1	ENSG00000116194.12	0.73	5.924	-2.001	1.98E-21
ANGPTL3	ENSG00000132855.4	0.115	5.356	-2.511	1.14E-22
ANK2	ENSG00000145362.16	2.83	33.765	-3.182	8.98E-20
ANKRD13B	ENSG00000198720.12	4.74	1.48	1.211	1.35E-11
ANKRD22	ENSG00000152766.5	0.51	2.11	-1.042	8.06E-08
ANKRD29	ENSG00000154065.16	10.04	3.01	1.461	2.19E-13
ANKRD46	ENSG00000186106.11	6.29	13.626	-1.005	3.75E-09
ANKRD65	ENSG00000235098.8	0.495	3.13	-1.466	4.75E-09

ANO1	ENSG00000131620.17	1.07	3.28	-1.048	8.30E-04
ANXA1	ENSG00000135046.13	243.824	42.457	2.494	4.92E-23
ANXA13	ENSG00000104537.16	20.534	1.539	3.084	7.12E-12
ANXA2	ENSG00000182718.16	702.059	237.37	1.56	4.11E-21
ANXA2P2	ENSG00000231991.4	13.865	4.63	1.401	9.12E-20
ANXA4	ENSG00000196975.14	246.833	106.879	1.2	1.54E-15
ANXA9	ENSG00000143412.9	3.115	15.59	-2.011	4.03E-11
AOAH	ENSG00000136250.11	4.37	1.54	1.08	4.80E-08
AOC3	ENSG00000131471.6	1.98	15.609	-2.479	3.68E-20
AOX1	ENSG00000138356.13	12.395	26.71	-1.049	3.63E-04
AP000322.53	ENSG00000243627.4	0.01	1.505	-1.31	4.08E-33
AP000349.2	ENSG00000280178.1	3.455	0.305	1.771	2.03E-13
AP000696.2	ENSG00000231324.1	0.02	1.615	-1.358	7.10E-40
AP000783.1	ENSG00000254667.2	0.2	1.745	-1.194	3.82E-26
AP000892.6	ENSG00000280143.1	1.025	4.695	-1.492	8.69E-16
AP002518.1	ENSG00000280653.1	89.384	7.122	3.476	5.92E-07
APBA2	ENSG00000034053.14	2.085	0.48	1.06	5.07E-11
APBB1IP	ENSG00000077420.15	27.214	2.41	3.049	3.29E-27
APBB2	ENSG00000163697.16	4.995	14.605	-1.38	1.95E-10
APCDD1	ENSG00000154856.12	1.005	5.137	-1.614	7.23E-11
APCDD1L	ENSG00000198768.10	0.03	1.96	-1.523	1.92E-34
APCDD1L-AS1	ENSG00000231290.5	0.06	4.424	-2.355	1.01E-45
APLN	ENSG00000171388.11	1.07	4.269	-1.348	2.86E-08
APLNR	ENSG00000134817.10	0.48	3.884	-1.722	4.39E-11
APLP1	ENSG00000105290.11	0.88	4.335	-1.505	2.40E-08
APOBEC3C	ENSG00000244509.3	21.58	4.533	2.029	2.74E-20
APOBR	ENSG00000184730.10	3.715	0.705	1.467	1.43E-19
APOC1	ENSG00000130208.9	466.02	8.652	5.597	3.84E-45
APOC1P1	ENSG00000214855.9	1.49	0.035	1.267	7.56E-16
APOC2	ENSG00000234906.8	9.66	0.92	2.473	1.59E-15
APOC3	ENSG00000110245.11	0	2.035	-1.602	3.28E-16
APOC4-APOC2	ENSG00000224916.8	14.32	4.989	1.355	6.87E-10
APOD	ENSG00000189058.8	1.645	20.805	-3.043	2.86E-22
APOH	ENSG00000091583.10	0.285	1.88	-1.164	1.07E-13
APOL1	ENSG00000100342.20	78.034	26.376	1.53	1.48E-07
APOL3	ENSG00000128284.19	5.56	15.605	-1.34	1.66E-12
APOL4	ENSG00000100336.17	0.435	2.03	-1.078	1.05E-11
APOLD1	ENSG00000178878.12	2.155	6.694	-1.286	1.81E-09
AQP2	ENSG00000167580.7	0.02	409.437	-8.652	7.78E-102
AQP3	ENSG00000165272.14	12.865	143.563	-3.382	2.02E-28
AQP5	ENSG00000161798.6	0.11	1.355	-1.085	5.22E-14
AQP6	ENSG00000086159.12	0.09	19.982	-4.267	1.77E-76
AQP7	ENSG00000165269.12	6.11	18.954	-1.489	1.23E-10
AR	ENSG00000169083.15	6.7	1.835	1.442	8.05E-11

ARAP1-AS1	ENSG00000256007.1	10.765	0	3.556	1.76E-08
ARAP3	ENSG00000120318.15	1.14	7.815	-2.042	1.14E-34
ARC	ENSG00000198576.3	0.14	1.334	-1.034	5.30E-24
ARG2	ENSG00000081181.7	2.83	40.233	-3.428	1.05E-24
ARHGAP22	ENSG00000128805.14	3.245	0.665	1.35	5.38E-19
ARHGAP23	ENSG00000275832.4	3.08	9.135	-1.313	4.42E-06
ARHGAP27	ENSG00000159314.11	25.925	12.06	1.044	4.98E-10
ARHGAP28	ENSG00000088756.12	1.06	3.32	-1.068	2.16E-14
ARHGEF15	ENSG00000198844.10	1.095	9.475	-2.322	6.84E-45
ARHGEF17	ENSG00000110237.3	11.205	23.76	-1.021	4.04E-11
ARHGEF26	ENSG00000114790.12	0.46	1.985	-1.032	5.53E-21
ARL15	ENSG00000185305.10	7.43	20.025	-1.319	1.49E-06
ARL4C	ENSG00000188042.7	19.6	5.144	1.745	7.01E-11
ARL4D	ENSG00000175906.4	9.665	25.358	-1.305	1.78E-09
ARL6IP5	ENSG00000144746.6	145.391	48.765	1.557	9.11E-26
ARPC1B	ENSG00000130429.12	291.468	59.85	2.265	2.71E-29
ARSE	ENSG00000157399.14	40.834	12.671	1.614	6.57E-09
ASAP2	ENSG00000151693.9	4.725	14.265	-1.415	2.84E-06
ASB9	ENSG00000102048.15	2.57	6.36	-1.044	1.15E-08
ASF1B	ENSG00000105011.8	2.4	0.555	1.129	5.08E-17
ASPDH	ENSG00000204653.9	1.515	20.569	-3.1	8.09E-24
ASPG	ENSG00000166183.15	0.225	6.805	-2.672	2.84E-23
ASPHD1	ENSG00000174939.10	19.34	1.435	3.062	1.08E-30
ASPN	ENSG00000106819.11	0.57	3.195	-1.418	1.89E-11
ASS1	ENSG00000130707.17	99.105	325.711	-1.707	7.45E-12
ATF3	ENSG00000162772.16	13.1	47.852	-1.793	3.55E-06
ATHL1	ENSG00000142102.15	61.684	18.165	1.71	1.01E-03
ATP12A	ENSG00000075673.11	0	2.44	-1.782	1.19E-61
ATP1A1	ENSG00000163399.15	402.152	1027.697	-1.351	4.64E-06
ATP1A2	ENSG00000018625.14	0.235	2.11	-1.332	4.70E-44
ATP1B2	ENSG00000129244.8	0.485	6.603	-2.356	7.36E-47
ATP2C2	ENSG00000064270.12	0.39	1.92	-1.071	1.26E-15
ATP6V0A4	ENSG00000105929.15	0.675	53.828	-5.033	2.30E-44
ATP6V0D2	ENSG00000147614.3	0.35	36.719	-4.804	7.74E-53
ATP6V1B1	ENSG00000116039.11	0.895	94.549	-5.656	1.09E-58
ATP6V1C2	ENSG00000143882.9	0.58	16.791	-3.493	1.93E-88
ATP6V1G2-DDX39B	ENSG00000254870.5	2.035	5.123	-1.013	1.18E-07
ATP6V1G3	ENSG00000151418.11	0	25.804	-4.744	3.16E-90
ATP8B3	ENSG00000130270.16	2.33	0.295	1.363	3.32E-15
AURKB	ENSG00000178999.12	1.88	0.39	1.051	1.80E-12
AUTS2	ENSG00000158321.15	4.57	11.13	-1.123	1.77E-07
AVPR1A	ENSG00000166148.3	0.08	1.784	-1.366	5.80E-29
AVPR2	ENSG00000126895.13	0.105	6.978	-2.852	1.35E-29

AZGP1	ENSG00000160862.12	22.32	72.164	-1.65	8.21E-05
AZGP1P1	ENSG00000214313.8	0.08	1.163	-1.002	1.60E-12
B3GALT5	ENSG00000183778.17	1.38	0.115	1.094	2.90E-10
B3GNT3	ENSG00000179913.10	9.11	3.11	1.299	6.13E-03
B3GNTL1	ENSG00000175711.8	7.805	2.05	1.53	3.02E-25
B4GALNT3	ENSG00000139044.10	0.53	7.035	-2.393	9.30E-26
B4GALT5	ENSG00000158470.5	57.799	18.804	1.57	1.18E-21
BAHCC1	ENSG00000266074.8	1.59	4.819	-1.168	2.58E-12
BAMBI	ENSG00000095739.10	43.594	14.969	1.482	1.30E-08
BASP1P1	ENSG00000230535.1	1.545	0.12	1.184	1.83E-07
BAX	ENSG00000087088.19	154.428	50.709	1.588	1.44E-31
BAZ1A	ENSG00000198604.10	8.67	3.75	1.026	1.66E-13
BBC3	ENSG00000105327.15	17.725	3.23	2.146	1.48E-48
BCAM	ENSG00000187244.10	112.321	349.402	-1.629	9.51E-14
BCHE	ENSG00000114200.9	9.575	1.315	2.192	2.60E-12
BCL11A	ENSG00000119866.20	2.35	0.44	1.218	6.86E-10
BCL2A1	ENSG00000140379.7	4.405	0.825	1.566	8.67E-12
BCL2L2	ENSG00000129473.9	12.465	27.019	-1.057	2.50E-08
BCL6B	ENSG00000161940.10	0.88	5.342	-1.754	9.78E-24
BCO1	ENSG00000135697.9	4.405	1.065	1.388	1.99E-17
BCO2	ENSG00000197580.11	8.555	2.48	1.457	1.10E-12
BDKRB1	ENSG00000100739.10	0.105	1.95	-1.417	2.16E-27
BDKRB2	ENSG00000168398.6	0.46	10.585	-2.988	8.68E-30
BEX5	ENSG00000184515.10	4.325	15.01	-1.588	1.21E-17
BHLHE41	ENSG00000123095.5	19.52	2.984	2.365	3.22E-32
BHMT	ENSG00000145692.14	13.819	114.555	-2.963	7.02E-12
BIN1	ENSG00000136717.14	150.398	66.963	1.156	4.00E-10
BIRC7	ENSG00000101197.12	1.475	0.16	1.093	4.19E-09
BIVM	ENSG00000134897.13	29.885	12.844	1.158	2.86E-11
BLNK	ENSG00000095585.16	5.785	19.255	-1.578	1.03E-12
BLVRA	ENSG00000106605.10	95.746	41.35	1.192	2.28E-21
BMP1	ENSG00000168487.17	18.695	7.275	1.251	2.45E-11
BMP7	ENSG00000101144.12	0.815	4.229	-1.527	1.30E-03
BMPR1B	ENSG00000138696.10	0.06	4.15	-2.281	4.59E-31
BOLA2	ENSG00000183336.7	12.485	5.735	1.002	1.46E-13
BPI	ENSG00000101425.12	0.29	1.699	-1.065	4.46E-18
BRSK1	ENSG00000160469.16	5.1	0.89	1.69	1.56E-12
BSND	ENSG00000162399.6	0	10.414	-3.513	1.19E-66
BSPRY	ENSG00000119411.10	4.05	12.32	-1.399	5.14E-14
BST2	ENSG00000130303.12	25.55	77.724	-1.568	2.09E-06
BTBD11	ENSG00000151136.14	0.395	3.114	-1.56	2.01E-04
BTK	ENSG00000010671.15	3.545	0.75	1.377	2.15E-13
BTNL9	ENSG00000165810.16	0.945	6.999	-2.04	1.47E-35
C10orf11	ENSG00000148655.14	32.725	15.18	1.06	1.41E-11

C11orf96	ENSG00000187479.5	5.485	38.96	-2.623	4.22E-23
C12orf75	ENSG00000235162.8	168.709	76.359	1.133	4.35E-07
C15orf48	ENSG00000166920.10	12.25	1.975	2.155	6.27E-15
C15orf59	ENSG00000205363.5	4.695	14.039	-1.401	1.05E-06
C15orf59-AS1	ENSG00000260469.2	0.03	1.368	-1.201	5.50E-51
C16orf59	ENSG00000162062.14	3.005	0.745	1.199	4.56E-19
C16orf74	ENSG00000154102.10	5.235	1.805	1.152	3.40E-07
C16orf89	ENSG00000153446.15	0.39	20.448	-3.948	4.46E-37
C17orf62	ENSG00000178927.16	75.68	34.611	1.107	3.68E-20
C17orf97	ENSG00000187624.8	22.43	8.56	1.293	9.18E-12
C19orf33	ENSG00000167644.11	270.052	23.933	3.442	1.66E-15
C19orf81	ENSG00000235034.6	1.765	0.35	1.034	5.04E-09
C1orf168	ENSG00000187889.12	0.14	5.36	-2.48	6.94E-50
C1orf186	ENSG00000263961.6	135.026	16.795	2.934	1.83E-17
C1orf226	ENSG00000239887.4	1.26	6.885	-1.803	4.87E-24
C1orf95	ENSG00000203685.9	0.29	2.055	-1.244	8.07E-07
C1QA	ENSG00000173372.16	108.642	27.704	1.933	6.65E-13
C1QB	ENSG00000173369.15	114.245	27.532	2.014	1.46E-14
C1QC	ENSG00000159189.11	85.995	21.124	1.975	2.04E-15
C1QL1	ENSG00000131094.3	0.43	2.965	-1.471	1.06E-03
C1QTNF3	ENSG00000082196.20	10.359	4.295	1.101	8.95E-04
C1QTNF6	ENSG00000133466.13	6.84	2.81	1.041	7.98E-09
C1QTNF7	ENSG00000163145.12	0.11	1.955	-1.413	1.37E-24
C1RL	ENSG00000139178.10	35.71	12.219	1.474	6.85E-19
C2orf40	ENSG00000119147.9	1.38	30.706	-3.736	3.11E-23
C2orf54	ENSG00000172478.17	0.2	5.209	-2.371	1.57E-25
C2orf72	ENSG00000204128.5	0.19	1.805	-1.237	1.11E-20
C2orf88	ENSG00000187699.10	1.595	4.559	-1.099	1.23E-07
C3	ENSG00000125730.16	702.692	25.001	4.758	2.62E-28
C3AR1	ENSG00000171860.4	7.06	2.38	1.254	3.25E-14
C3orf67	ENSG00000163689.18	3.17	0.73	1.269	3.21E-15
C4A	ENSG00000244731.7	432.992	149.214	1.531	1.89E-09
C4B	ENSG00000224389.8	419.244	157.035	1.411	1.33E-09
C4orf48	ENSG00000243449.6	25.665	6.631	1.805	8.33E-11
C5AR2	ENSG00000134830.5	0.265	1.56	-1.017	2.50E-17
C5orf38	ENSG00000186493.11	0.4	6.16	-2.355	4.16E-18
C5orf49	ENSG00000215217.6	7.555	2.79	1.175	1.33E-08
C6	ENSG00000039537.13	4.509	1.085	1.402	6.39E-05
C6orf223	ENSG00000181577.15	0.05	1.495	-1.249	6.57E-13
C7	ENSG00000112936.18	7.739	238.599	-4.777	5.19E-28
C8orf4	ENSG00000176907.4	6.45	57.418	-2.971	1.67E-14
C9orf116	ENSG00000160345.12	24.995	8.194	1.499	3.33E-21
C9orf24	ENSG00000164972.12	1.24	4.485	-1.292	2.13E-10
CA10	ENSG00000154975.13	0.04	5.181	-2.571	3.12E-36

CA12	ENSG00000074410.13	33.905	178.119	-2.359	1.69E-10
CA2	ENSG00000104267.9	61.286	175.137	-1.5	7.53E-04
CA4	ENSG00000167434.9	0.37	29.545	-4.479	8.89E-54
CA8	ENSG00000178538.9	0.07	2.785	-1.823	1.75E-55
CA9	ENSG00000107159.12	2.2	0.215	1.397	1.05E-08
CACHD1	ENSG00000158966.13	0.85	2.903	-1.077	4.24E-11
CACNA1C	ENSG00000151067.20	0.23	1.795	-1.184	1.33E-25
CACNA1H	ENSG00000196557.10	1.23	10.154	-2.322	9.13E-22
CACNA2D2	ENSG00000007402.11	0.545	2.275	-1.084	9.64E-21
CACNB3	ENSG00000167535.7	19.059	6.09	1.5	8.51E-10
CALB1	ENSG00000104327.7	0.11	88.487	-6.333	9.31E-72
CALCA	ENSG00000110680.12	0.02	3.112	-2.011	6.93E-49
CALCRL	ENSG00000064989.12	3.3	7.923	-1.053	7.26E-03
CALML3	ENSG00000178363.4	0.04	2.77	-1.858	1.52E-32
CAMK1G	ENSG00000008118.9	0.435	2.205	-1.159	1.57E-20
CAMK2N1	ENSG00000162545.5	29.305	74.397	-1.315	6.06E-08
CAPG	ENSG00000042493.15	485.305	181.332	1.415	1.28E-12
CAPN12	ENSG00000182472.8	22.683	6.819	1.599	6.64E-03
CAPN13	ENSG00000162949.16	0.49	2.38	-1.182	1.07E-16
CAPS	ENSG00000105519.12	10.61	35.481	-1.652	2.01E-15
CARD11	ENSG00000198286.9	2.605	0.75	1.043	8.55E-10
CASP1	ENSG00000137752.22	16.015	6.77	1.131	1.05E-07
CASQ2	ENSG00000118729.11	0.08	1.31	-1.097	3.63E-26
CASR	ENSG00000036828.13	0.31	18.435	-3.891	1.08E-46
CASZ1	ENSG00000130940.14	2.405	6.215	-1.083	1.09E-15
CAV2	ENSG00000105971.14	39.675	14.18	1.422	2.22E-15
CBX7	ENSG00000100307.12	14.035	34.57	-1.242	1.14E-09
CCBE1	ENSG00000183287.13	0.04	1.45	-1.236	1.22E-26
CCDC109B	ENSG00000005059.15	6.655	1.495	1.617	1.06E-14
CCDC178	ENSG00000166960.16	0.41	2.315	-1.233	2.18E-20
CCDC3	ENSG00000151468.10	1.68	6.738	-1.53	1.85E-10
CCDC74A	ENSG00000163040.14	16.965	4.702	1.656	5.23E-12
CCDC74B	ENSG00000152076.18	8.3	2.755	1.309	3.32E-06
CCDC78	ENSG00000162004.16	3.755	1.1	1.179	2.59E-09
CCDC88B	ENSG00000168071.21	19.39	3.504	2.178	4.81E-36
CCDC88C	ENSG00000015133.18	2.98	7.705	-1.129	3.02E-10
CCL11	ENSG00000172156.3	0.06	1.299	-1.117	6.86E-29
CCL14	ENSG00000276409.4	3.68	33.476	-2.881	1.32E-24
CCL15-CCL14	ENSG00000275688.4	4.78	11.089	-1.065	8.01E-04
CCL18	ENSG00000275385.1	18.061	0.1	4.115	7.73E-17
CCL19	ENSG00000172724.11	0.44	4.67	-1.977	7.86E-07
CCL21	ENSG00000137077.7	0.995	13.385	-2.85	3.22E-12
CCL23	ENSG00000274736.4	0.29	1.715	-1.074	1.51E-24
CCL28	ENSG00000151882.11	10.97	2.31	1.855	4.92E-06

CCL3	ENSG00000277632.1	6.35	2.289	1.16	2.47E-06
CCL4L2	ENSG00000276070.4	5.504	1.625	1.309	6.09E-08
CCL5	ENSG00000271503.5	13.47	3.863	1.573	4.65E-08
CCM2L	ENSG00000101331.15	0.9	5.329	-1.736	1.15E-30
CCND2	ENSG00000118971.7	22.415	3.466	2.39	6.24E-18
CCNO	ENSG00000152669.8	4.32	1.42	1.137	6.99E-07
CCSER1	ENSG00000184305.14	0.875	3.94	-1.398	1.39E-14
CCZ1	ENSG00000122674.11	68.02	26.723	1.316	5.38E-21
CCZ1B	ENSG00000146574.15	44.62	17.211	1.325	2.23E-13
CD14	ENSG00000170458.13	47.78	22.826	1.034	9.83E-09
CD151	ENSG00000177697.17	450.957	206.757	1.121	3.73E-16
CD163	ENSG00000177575.12	14.57	6.319	1.089	4.59E-04
CD2	ENSG00000116824.4	3.8	0.85	1.376	7.73E-09
CD200	ENSG00000091972.18	17.062	5.47	1.481	7.54E-08
CD22	ENSG00000012124.14	3.275	1.083	1.037	1.38E-07
CD24	ENSG00000272398.5	1672.568	537.268	1.637	8.05E-14
CD248	ENSG00000174807.3	2.02	8.695	-1.683	3.30E-14
CD276	ENSG00000103855.17	51.805	18.845	1.412	5.83E-29
CD300A	ENSG00000167851.13	6.33	1.33	1.654	2.59E-16
CD300LF	ENSG00000186074.18	2.06	0.31	1.224	1.91E-14
CD300LG	ENSG00000161649.12	0.17	1.665	-1.188	2.27E-16
CD34	ENSG00000174059.16	6.2	45.079	-2.678	4.78E-24
CD37	ENSG00000104894.11	22.351	4.655	2.046	6.42E-15
CD3D	ENSG00000167286.9	4.035	1.189	1.202	1.11E-06
CD3E	ENSG00000198851.9	5.87	1.535	1.439	1.49E-06
CD4	ENSG00000010610.9	16.264	7.455	1.03	1.47E-09
CD48	ENSG00000117091.9	9.459	2.104	1.752	1.98E-10
CD52	ENSG00000169442.8	38.744	7.91	2.157	3.53E-11
CD53	ENSG00000143119.12	23.685	11.286	1.007	6.06E-08
CD63	ENSG00000135404.11	1693.86	657.341	1.364	5.48E-28
CD68	ENSG00000129226.13	171.04	28.526	2.543	1.10E-29
CD70	ENSG00000125726.10	6.285	0.17	2.638	3.67E-16
CD82	ENSG00000085117.11	14.975	32.318	-1.06	2.15E-03
CD86	ENSG00000114013.15	3.665	0.815	1.362	1.57E-18
CD93	ENSG00000125810.9	3.715	13.393	-1.61	1.03E-08
CDA	ENSG00000158825.5	1.205	11.339	-2.484	1.87E-31
CDC42EP2	ENSG00000149798.4	3.1	8.915	-1.274	7.75E-14
CDC42EP3	ENSG00000163171.7	6.69	17.304	-1.251	5.46E-08
CDCA7L	ENSG00000164649.19	6.96	2.865	1.042	1.38E-05
CDH1	ENSG00000039068.18	18.09	40.513	-1.121	9.54E-03
CDH11	ENSG00000140937.13	0.94	4.867	-1.596	1.19E-05
CDH13	ENSG00000140945.15	1.22	7.05	-1.858	1.23E-09
CDH16	ENSG00000166589.12	105.189	452.9	-2.096	1.43E-09
CDH2	ENSG00000170558.8	24.52	6.039	1.858	2.62E-08

CDH3	ENSG00000062038.13	0.34	9.92	-3.027	1.20E-25
CDH5	ENSG00000179776.17	3.105	15.32	-1.991	2.03E-17
CDH6	ENSG00000113361.12	57.199	10.104	2.39	1.53E-12
CDHR1	ENSG00000148600.14	3.99	0.5	1.734	3.01E-09
CDHR2	ENSG00000074276.10	18.67	6.804	1.334	5.28E-04
CDKL1	ENSG00000100490.9	6.765	15.034	-1.046	3.58E-03
CDKN1A	ENSG00000124762.13	172.397	55.589	1.615	2.24E-14
CDKN1C	ENSG00000129757.12	15.27	66.68	-2.057	1.26E-21
CDKN2A	ENSG00000147889.16	4.14	0.23	2.063	1.56E-19
CDO1	ENSG00000129596.4	0.395	2.08	-1.143	4.25E-04
CDR2	ENSG00000140743.7	37.715	16.635	1.134	8.20E-13
CDT1	ENSG00000167513.8	2.63	0.565	1.214	1.40E-21
CEACAM1	ENSG00000079385.21	2.2	19.918	-2.709	5.02E-39
CEACAM19	ENSG00000186567.12	1.435	3.885	-1.004	1.92E-04
CEBPA	ENSG00000245848.2	7.84	2.439	1.362	1.64E-14
CEL	ENSG00000170835.13	0.295	6.2	-2.475	1.94E-48
CENPH	ENSG00000153044.9	3.58	1.19	1.064	2.78E-17
CERS6-AS1	ENSG00000227617.8	11.885	4.85	1.139	1.30E-03
CES3	ENSG00000172828.12	1.545	4.27	-1.05	5.93E-08
CFB	ENSG00000243649.8	162.759	33.375	2.252	4.04E-11
CFD	ENSG00000197766.7	12.96	5.13	1.187	2.81E-03
CGN	ENSG00000143375.14	4.885	19.135	-1.775	5.61E-17
CGNL1	ENSG00000128849.10	4.035	46.787	-3.247	9.32E-21
CGREF1	ENSG00000138028.14	6.955	2.495	1.187	1.08E-06
CH17-13I23.3	ENSG00000277702.1	15.475	6.865	1.067	3.51E-05
CH507-152C13.3	ENSG00000276076.4	0.075	36.816	-5.137	2.30E-54
CH507-396I9.3	ENSG00000278961.1	0.01	1.505	-1.31	4.08E-33
CH507-42P11.8	ENSG00000275993.2	4.81	11.147	-1.064	4.30E-05
CH507-9B2.2	ENSG00000279493.1	0	1.165	-1.115	4.82E-27
CHAC1	ENSG00000128965.11	0.805	4.485	-1.603	4.50E-20
CHEK2	ENSG00000183765.20	8.34	2.6	1.375	4.17E-18
CHGB	ENSG00000089199.9	1.509	6.112	-1.503	3.13E-06
CHI3L1	ENSG00000133048.12	4.49	24.879	-2.237	7.74E-03
CHI3L2	ENSG00000064886.13	4.695	0.82	1.646	6.35E-07
CHIT1	ENSG00000133063.15	11.358	0.22	3.341	1.10E-14
CHPF	ENSG00000123989.13	83.395	36.108	1.185	2.10E-13
CHPF2	ENSG00000033100.14	27.739	13.158	1.021	2.83E-19
CHRD	ENSG00000090539.15	1.305	5.825	-1.566	1.26E-11
CHRDL1	ENSG00000101938.14	0.385	7.197	-2.565	1.84E-13
CHRDL2	ENSG00000054938.15	0.065	1.885	-1.438	1.45E-18
CHRNA4	ENSG00000101204.15	0	1.34	-1.226	1.25E-46
CHRNB1	ENSG00000170175.10	19.165	8.74	1.05	3.23E-07
CHST1	ENSG00000175264.7	0.44	1.915	-1.017	3.58E-10
CHST13	ENSG00000180767.9	6.77	1.919	1.412	4.94E-09

CHST2	ENSG00000175040.5	1.11	5.015	-1.511	2.25E-09
CHST9	ENSG00000154080.12	4.68	1.105	1.432	4.16E-05
CHTF18	ENSG00000127586.16	7.039	3.01	1.004	4.87E-06
CIT	ENSG00000122966.13	3.51	9.205	-1.178	2.65E-04
CITED4	ENSG00000179862.6	34.899	11.071	1.572	2.50E-08
CKLF	ENSG00000217555.12	40.126	14.905	1.371	9.81E-25
CKMT2	ENSG00000131730.15	0.36	13.699	-3.434	1.23E-28
CLCF1	ENSG00000175505.10	20.37	8.469	1.174	1.68E-10
CLCNKA	ENSG00000186510.11	0.65	46.269	-4.84	1.84E-76
CLCNKB	ENSG00000184908.17	2.555	169.959	-5.588	1.74E-59
CLDN1	ENSG00000163347.5	60.475	7.98	2.775	2.43E-19
CLDN10	ENSG00000134873.9	20.935	94.667	-2.125	2.23E-13
CLDN11	ENSG00000013297.10	0.285	4.679	-2.144	9.71E-21
CLDN14	ENSG00000159261.10	1.09	7.624	-2.045	3.77E-25
CLDN16	ENSG00000113946.3	1.01	13.445	-2.845	2.09E-26
CLDN19	ENSG00000164007.10	0.15	17.775	-4.029	1.08E-36
CLDN2	ENSG00000165376.10	95.025	43.086	1.123	6.02E-04
CLDN3	ENSG00000165215.6	193.299	41.871	2.18	9.23E-09
CLDN4	ENSG00000189143.9	279.605	90.612	1.615	1.19E-10
CLDN5	ENSG00000184113.9	5.675	22.72	-1.829	3.38E-23
CLDN8	ENSG00000156284.5	0	30.585	-4.981	2.52E-67
CLEC14A	ENSG00000176435.6	3.465	13.286	-1.678	6.91E-14
CLEC18A	ENSG00000157322.16	2.925	12.312	-1.762	2.62E-06
CLEC18B	ENSG00000140839.11	5.12	23.418	-1.996	8.99E-07
CLEC18C	ENSG00000157335.19	1.42	10.108	-2.199	2.72E-09
CLEC1A	ENSG00000150048.10	0.52	2.33	-1.131	6.71E-24
CLEC3B	ENSG00000163815.5	7.845	91.597	-3.388	2.77E-39
CLIC4	ENSG00000169504.14	139.444	68.707	1.011	4.07E-05
CLIC5	ENSG00000112782.15	0.55	26.884	-4.169	1.14E-48
CLIC6	ENSG00000159212.12	10.16	1.64	2.08	1.91E-05
CLMN	ENSG00000165959.11	5.365	17.79	-1.562	6.23E-21
CLMP	ENSG00000166250.11	0.29	1.733	-1.083	4.71E-09
CLNK	ENSG00000109684.14	0.04	2.05	-1.552	6.27E-46
CLRN3	ENSG00000180745.4	29.019	9.27	1.548	5.85E-04
CLU	ENSG00000120885.19	5140.346	1033.519	2.313	5.32E-20
CLUL1	ENSG00000079101.16	0.945	3.395	-1.176	2.27E-12
CMAHP	ENSG00000168405.14	3.93	13.515	-1.558	1.46E-14
CMTM3	ENSG00000140931.19	32.475	9.944	1.613	8.28E-15
CMTM7	ENSG00000153551.13	38.763	12.119	1.6	5.78E-18
CNFN	ENSG00000105427.9	6.07	1.5	1.5	6.15E-13
CNGA1	ENSG00000198515.13	0.21	1.875	-1.249	9.93E-24
CNKSR1	ENSG00000142675.17	0.27	7.449	-2.734	6.60E-24
CNKSR3	ENSG00000153721.17	30.228	14.61	1	3.89E-08
CNN1	ENSG00000130176.7	1.565	11.524	-2.288	1.02E-12

CNPY3	ENSG00000137161.16	58.781	25.656	1.165	1.14E-20
CNRIP1	ENSG00000119865.8	1.87	4.935	-1.048	6.71E-12
CNTFR	ENSG00000122756.14	0.06	1.25	-1.086	3.00E-23
CNTN1	ENSG00000018236.14	0.12	4.274	-2.235	4.10E-47
CNTN6	ENSG00000134115.12	8.76	0.12	3.123	6.14E-19
COBLL1	ENSG00000082438.15	25.37	52.225	-1.013	9.07E-03
COL12A1	ENSG00000111799.20	3.325	9.565	-1.288	2.72E-07
COL14A1	ENSG00000187955.11	1.455	10.89	-2.276	4.69E-13
COL16A1	ENSG00000084636.17	4.01	9.135	-1.016	1.52E-05
COL23A1	ENSG00000050767.15	13.895	1.855	2.383	3.04E-15
COL4A3	ENSG00000169031.18	6.555	21.05	-1.545	1.19E-14
COL4A4	ENSG00000081052.11	3.53	9.99	-1.279	1.44E-08
COL4A6	ENSG00000197565.15	0.16	2.73	-1.685	2.73E-31
COL6A3	ENSG00000163359.15	1.635	5.865	-1.381	2.88E-05
COL7A1	ENSG00000114270.15	1.15	4.993	-1.479	1.58E-13
COL8A2	ENSG00000171812.10	2.48	0.57	1.148	7.50E-13
COL9A2	ENSG00000049089.13	2.25	11.68	-1.964	1.09E-16
COLEC11	ENSG00000118004.17	5.38	15.305	-1.354	1.09E-05
COLGALT1	ENSG00000130309.10	33.075	15.595	1.038	3.24E-20
CORO1A	ENSG00000102879.15	24.609	7.83	1.536	6.25E-12
CORO1C	ENSG00000110880.10	46.226	19.09	1.233	1.01E-22
CORO2B	ENSG00000103647.12	2.675	6.84	-1.093	7.42E-05
CORO6	ENSG00000167549.18	3.26	0.98	1.106	6.10E-05
CORO7	ENSG00000262246.5	23.995	10.979	1.061	1.07E-16
COX6A2	ENSG00000156885.5	0.54	2.63	-1.237	4.47E-06
COX7A1	ENSG00000161281.10	12.985	88.122	-2.672	9.01E-06
CPA3	ENSG00000163751.3	5.185	1.045	1.597	3.85E-05
CPAMD8	ENSG00000160111.12	1.12	14.003	-2.823	1.19E-44
CPED1	ENSG00000106034.17	0.81	2.94	-1.122	3.57E-06
CPN2	ENSG00000178772.6	0.05	4.278	-2.33	9.33E-13
CPNE5	ENSG00000124772.11	0.39	2.035	-1.127	1.36E-05
CPNE7	ENSG00000178773.14	6.47	0.845	2.018	5.39E-10
CPT1A	ENSG00000110090.12	15.07	34.605	-1.148	1.41E-05
CPXM1	ENSG00000088882.7	1	3.865	-1.282	1.75E-07
CPXM2	ENSG00000121898.12	1.22	9.008	-2.173	2.02E-05
CRABP1	ENSG00000166426.7	0	8.165	-3.196	7.35E-39
CRABP2	ENSG00000143320.8	1.435	6.478	-1.619	1.02E-08
CRACR2A	ENSG00000130038.9	1.79	0.335	1.063	5.42E-15
CRB2	ENSG00000148204.11	0.06	3.133	-1.963	2.59E-18
CREB3L1	ENSG00000157613.10	0.43	2.615	-1.338	3.81E-03
CREB5	ENSG00000146592.16	21.6	3.15	2.445	7.08E-28
CRHBP	ENSG00000145708.10	0.31	20.199	-4.016	1.27E-76
CRISPLD2	ENSG00000103196.11	3.75	10.385	-1.261	1.27E-05
CRNDE	ENSG00000245694.8	38.654	6.334	2.435	3.05E-12

CRYAA	ENSG00000160202.7	0	29.204	-4.917	3.13E-55
CRYAB	ENSG00000109846.7	5188.852	934.466	2.472	1.78E-16
CRYBG3	ENSG00000080200.9	1.705	4.485	-1.02	5.47E-10
CSDC2	ENSG00000172346.14	5.71	21.096	-1.719	8.83E-08
CSF1R	ENSG00000182578.13	15.635	6.92	1.071	2.18E-10
CSF2RA	ENSG00000198223.14	6.25	1.77	1.388	7.06E-13
CSF3R	ENSG00000119535.17	7.37	2.12	1.424	9.72E-10
CSGALNACT1	ENSG00000147408.14	1.11	7.37	-1.988	9.69E-11
CSPG4	ENSG00000173546.7	0.725	2.985	-1.208	1.29E-09
CSRNP1	ENSG00000144655.14	8.04	17.999	-1.072	7.32E-06
CSRP1	ENSG00000159176.13	57.39	138.942	-1.261	2.04E-11
CSRP2	ENSG00000175183.9	12.8	45.727	-1.76	1.32E-11
CSTA	ENSG00000121552.3	4.025	0.939	1.374	8.74E-11
CTA-392C11.1	ENSG00000253802.1	0	1.365	-1.242	3.60E-55
CTA-963H5.5	ENSG00000250318.1	4.455	1.72	1.004	1.88E-07
CTB-171A8.1	ENSG00000266903.1	0.615	2.722	-1.204	4.09E-25
CTB-193M12.5	ENSG00000280206.1	4.73	1.47	1.214	5.47E-20
CTB-25B13.12	ENSG00000267317.2	13.285	6.06	1.017	7.12E-18
CTB-63M22.1	ENSG00000229119.3	164.477	29.479	2.441	3.42E-11
CTB-79E8.3	ENSG00000253683.1	0	1.119	-1.084	1.83E-08
CTC-425F1.4	ENSG00000267458.1	10.315	3.078	1.472	7.80E-04
CTC-510F12.6	ENSG00000267576.1	14.445	3.577	1.755	2.45E-08
CTD-2004A9.1	ENSG00000250885.1	0	1.135	-1.094	5.27E-35
CTD-2008P7.9	ENSG00000267259.1	0	1.255	-1.173	2.97E-41
CTD-2247C11.5	ENSG00000250529.1	0	3.102	-2.036	5.66E-46
CTD-2342N23.1	ENSG00000278898.1	0.01	2.435	-1.766	4.09E-59
CTD-2369P2.12	ENSG00000267303.1	14.215	6.47	1.026	2.47E-03
CTD-2377D24.6	ENSG00000244649.4	6.02	1.469	1.507	8.91E-06
CTD-2510F5.6	ENSG00000265303.1	1.265	0	1.179	7.29E-05
CTD-2515H24.2	ENSG00000276772.1	0.07	1.19	-1.033	3.08E-60
CTD-2531D15.5	ENSG00000255126.1	12.175	2.702	1.831	2.48E-03
CTD-2540B15.11	ENSG00000267580.1	13.385	2.209	2.164	1.70E-17
CTD-3157E16.2	ENSG00000276855.1	23	10.54	1.056	4.09E-08
CTGF	ENSG00000118523.5	33.704	156.703	-2.184	4.51E-11
CTSC	ENSG00000109861.15	481.602	121.511	1.978	1.86E-25
CTSS	ENSG00000163131.10	42.585	12.671	1.673	3.87E-11
CTSZ	ENSG00000101160.13	301.757	112.742	1.412	6.81E-17
CTXN1	ENSG00000178531.5	16.45	5.418	1.443	3.08E-08
CTXN3	ENSG00000205279.8	0.02	8.771	-3.26	1.45E-39
CUBN	ENSG00000107611.14	5.89	17.081	-1.392	4.42E-04
CUX1	ENSG00000257923.9	100.016	49.164	1.01	3.06E-13
CWH43	ENSG00000109182.11	0.51	5.673	-2.144	2.39E-08
CX3CR1	ENSG00000168329.13	2.44	0.62	1.086	6.57E-08
CXCL1	ENSG00000163739.4	12.93	3.594	1.6	4.55E-04

CXCL12	ENSG00000107562.16	13.995	85.029	-2.52	5.27E-12
CXCL16	ENSG00000161921.14	101.405	26.16	1.915	6.43E-25
CXCL6	ENSG00000124875.9	11.753	0.85	2.785	5.75E-09
CXCL8	ENSG00000169429.10	5.575	1.934	1.164	7.58E-06
CXCR4	ENSG00000121966.6	33.011	10.914	1.513	1.77E-09
CYB5A	ENSG00000166347.18	198.122	414.534	-1.061	8.73E-08
CYBA	ENSG00000051523.10	507.406	124.801	2.015	2.96E-28
CYBB	ENSG00000165168.7	7.48	2.538	1.261	4.22E-10
CYBRD1	ENSG00000071967.11	6.175	19.146	-1.489	3.55E-04
CYFIP2	ENSG00000055163.18	50.185	143.822	-1.5	1.35E-13
CYP17A1	ENSG00000148795.5	1.175	11.146	-2.481	8.27E-11
CYP1B1	ENSG00000138061.11	5.47	14.325	-1.244	6.14E-03
CYP27A1	ENSG00000135929.8	49.096	23.31	1.043	1.69E-06
CYP27B1	ENSG00000111012.9	1.61	11.059	-2.208	1.38E-15
CYP2B6	ENSG00000197408.8	0.01	3.774	-2.241	5.82E-46
CYP3A5	ENSG00000106258.13	30.965	5.123	2.384	5.26E-12
CYP4A11	ENSG00000187048.12	0.48	66.19	-5.505	3.14E-34
CYP4A22	ENSG00000162365.11	0.06	9.1	-3.252	2.70E-42
CYP4F2	ENSG00000186115.12	0.04	5.31	-2.601	6.72E-29
CYP4F3	ENSG00000186529.14	0.615	7.625	-2.417	3.84E-08
CYP4X1	ENSG00000186377.7	0.305	2.94	-1.594	3.17E-32
CYP8B1	ENSG00000180432.5	0.66	3.421	-1.413	1.16E-15
CYR61	ENSG00000142871.15	30.514	127.009	-2.022	1.12E-09
CYYR1	ENSG00000166265.11	1.435	9.05	-2.045	2.53E-24
DAAM2	ENSG00000146122.16	0.675	6.21	-2.106	5.56E-33
DACH1	ENSG00000276644.4	0.15	4.045	-2.133	1.71E-27
DACT3	ENSG00000197380.10	0.475	2.695	-1.325	1.91E-21
DAGLB	ENSG00000164535.14	17.47	6.195	1.36	1.13E-27
DAO	ENSG00000110887.7	2.695	10.504	-1.639	2.03E-08
DBH-AS1	ENSG00000225756.1	0.74	2.705	-1.09	3.53E-06
DBNDD1	ENSG00000003249.13	36.784	12.375	1.498	2.62E-18
DCBLD2	ENSG00000057019.15	18.175	8.213	1.058	8.74E-09
DCHS1	ENSG00000166341.7	0.725	3.189	-1.28	4.83E-12
DCN	ENSG00000011465.16	16.055	256.417	-3.916	8.75E-21
DCXR	ENSG00000169738.7	100.064	208.348	-1.051	4.78E-07
DDB2	ENSG00000134574.11	48.515	10.095	2.158	1.10E-33
DDC	ENSG00000132437.17	24.805	67.505	-1.409	1.47E-03
DDN	ENSG00000181418.7	0.03	8.335	-3.18	7.20E-57
DDO	ENSG00000203797.9	7.865	2.815	1.217	1.44E-08
DDR2	ENSG00000162733.16	0.475	3.125	-1.484	6.14E-15
DDX25	ENSG00000109832.12	0.14	1.56	-1.167	7.28E-31
DDX3Y	ENSG00000067048.16	0.94	18.395	-3.322	1.04E-15
DEF6	ENSG00000023892.10	4.99	1.84	1.077	3.32E-08
DENND2A	ENSG00000146966.12	1.38	7.16	-1.778	1.34E-12

DEPTOR	ENSG00000155792.9	9.68	29.954	-1.535	3.82E-10
DES	ENSG00000175084.11	0.32	6.285	-2.464	8.57E-23
DGAT2	ENSG00000062282.14	0.75	2.564	-1.026	5.31E-04
DGCR5	ENSG00000237517.8	5.155	0.81	1.766	1.83E-14
DGCR9	ENSG00000273032.1	1.905	0.385	1.068	9.01E-11
DGKA	ENSG00000065357.19	16.91	5.965	1.363	3.28E-06
DIO1	ENSG00000211452.10	0.72	15.931	-3.299	2.41E-12
DIO3OS	ENSG00000258498.6	0.07	1.645	-1.306	3.92E-25
DIRAS1	ENSG00000176490.4	0.54	2.915	-1.346	3.57E-06
DLC1	ENSG00000164741.14	4.025	11.115	-1.27	6.14E-09
DLGAP1	ENSG00000170579.14	4.17	0.57	1.72	3.30E-06
DLL1	ENSG00000198719.8	0.57	5.449	-2.038	8.79E-17
DLL4	ENSG00000128917.6	1.74	5.723	-1.295	4.47E-11
DMGDH	ENSG00000132837.14	12.375	27.538	-1.093	2.93E-07
DMRT2	ENSG00000173253.14	0.01	21.914	-4.504	1.76E-93
DNAAF1	ENSG00000154099.17	3.95	1.474	1.001	4.24E-03
DNAAF3	ENSG00000167646.13	11.375	3.128	1.584	7.26E-11
DNAJB13	ENSG00000187726.8	5.93	0.52	2.189	1.30E-11
DNAJC22	ENSG00000178401.14	14.75	5.189	1.348	3.58E-10
DNASE1	ENSG00000213918.10	5.365	36.344	-2.553	2.76E-44
DNASE1L3	ENSG00000163687.13	2.21	26.421	-3.095	9.36E-49
DNER	ENSG00000187957.7	0.665	8.635	-2.533	2.77E-13
DNM1	ENSG00000106976.18	34.97	6.3	2.301	2.86E-15
DNM3	ENSG00000197959.13	0.69	3.385	-1.376	6.21E-19
DOC2A	ENSG00000149927.17	8.37	1.005	2.225	1.69E-06
DOC2B	ENSG00000272636.3	0.32	5.395	-2.276	9.31E-21
DOCK11	ENSG00000147251.15	24.68	5.66	1.947	2.10E-19
DOCK2	ENSG00000134516.15	3.71	1.245	1.069	5.62E-10
DOK1	ENSG00000115325.13	10.05	4.37	1.041	1.77E-15
DPCD	ENSG00000166171.12	59.225	26.154	1.149	3.98E-16
DPEP1	ENSG00000015413.9	13.76	111.747	-2.933	1.36E-08
DPEP2	ENSG00000167261.13	3.055	0.965	1.045	9.91E-10
DPP4	ENSG00000197635.9	65.437	25.869	1.306	1.63E-03
DPP6	ENSG00000130226.16	0.05	2.71	-1.821	2.89E-17
DPT	ENSG00000143196.4	0.08	3.436	-2.038	4.68E-42
DPYS	ENSG00000147647.12	12.25	51.051	-1.974	6.92E-06
DPYSL3	ENSG00000113657.12	1.2	5.727	-1.612	5.42E-05
DRAIC	ENSG00000245750.7	0.635	4.665	-1.793	1.79E-31
DRC7	ENSG00000159625.14	5.72	0.79	1.909	3.21E-15
DTNA	ENSG00000134769.21	10.375	3.18	1.444	1.39E-20
DTX1	ENSG00000135144.7	2.415	7.055	-1.238	8.45E-12
DTX2	ENSG00000091073.19	13.805	6.275	1.025	2.62E-16
DUSP1	ENSG00000120129.5	58.116	192.557	-1.711	1.38E-05
DUSP10	ENSG00000143507.17	4.705	1.174	1.392	3.43E-15

DUSP15	ENSG00000149599.15	10.665	24.09	-1.105	4.32E-07
DUSP2	ENSG00000158050.4	2.17	5.34	-1	1.14E-06
DUSP26	ENSG00000133878.8	0.11	1.595	-1.225	4.68E-30
DUSP6	ENSG00000139318.7	18.115	44.944	-1.265	2.17E-03
DUSP9	ENSG00000130829.17	0.06	36.859	-5.159	2.10E-79
DYNC2LI1	ENSG00000138036.18	81.868	28.879	1.472	8.77E-09
DYNLT3	ENSG00000165169.10	11.675	25.178	-1.046	4.70E-08
DYSF	ENSG00000135636.13	3.415	10.36	-1.363	8.12E-04
E2F1	ENSG00000101412.12	2.55	0.445	1.297	1.68E-20
EBF1	ENSG00000164330.16	0.355	2.73	-1.461	9.59E-17
EBI3	ENSG00000105246.5	3.265	1	1.093	4.65E-12
ECHDC3	ENSG00000134463.14	16.13	50.067	-1.576	2.63E-10
EDA2R	ENSG00000131080.14	3.61	0.62	1.509	4.49E-25
EDIL3	ENSG00000164176.12	12.693	5.575	1.058	7.32E-03
EDNRA	ENSG00000151617.15	0.44	2.595	-1.32	3.40E-15
EEF1A2	ENSG00000101210.10	13.82	1.58	2.522	7.22E-08
EFCAB12	ENSG00000172771.11	3.405	1.135	1.045	6.47E-07
EFEMP1	ENSG00000115380.18	5.595	47.212	-2.87	5.63E-11
EFHD1	ENSG00000115468.11	32.959	198.293	-2.553	5.34E-18
EFNA5	ENSG00000184349.12	1.724	5.09	-1.16	4.45E-04
EFS	ENSG00000100842.12	0.23	3.415	-1.844	2.11E-21
EGF	ENSG00000138798.11	0.91	22.36	-3.612	7.13E-52
EGFL7	ENSG00000172889.15	7.52	58.415	-2.802	7.62E-33
EGFLAM	ENSG00000164318.17	0.97	3.89	-1.312	3.53E-12
EGR1	ENSG00000120738.7	39.968	109.781	-1.435	2.30E-04
EHD2	ENSG00000024422.11	40.5	17.677	1.152	9.14E-08
EHD3	ENSG00000013016.14	3.3	11.485	-1.538	1.52E-16
EHF	ENSG00000135373.12	0.53	8.489	-2.633	9.64E-20
EIF1AY	ENSG00000198692.9	1.295	20.682	-3.24	4.01E-14
ELF3	ENSG00000163435.15	304.615	104.673	1.532	1.09E-10
ELF5	ENSG00000135374.9	0.05	4.538	-2.399	1.48E-49
ELN	ENSG00000049540.16	2.205	10.434	-1.835	1.05E-09
EMCN	ENSG00000164035.9	2.48	47.502	-3.801	6.43E-53
EMID1	ENSG00000186998.15	2.355	8.235	-1.461	2.32E-16
EMILIN1	ENSG00000138080.13	5.55	17.751	-1.517	3.15E-10
EMILIN2	ENSG00000132205.10	10.08	2.994	1.472	3.57E-16
EMP3	ENSG00000142227.10	95.424	34.733	1.432	7.83E-14
EMX1	ENSG00000135638.13	21.338	62.911	-1.517	2.02E-11
ENG	ENSG00000106991.13	32.675	118.68	-1.829	2.70E-22
ENKD1	ENSG00000124074.11	42.07	19.47	1.073	3.97E-07
ENO2	ENSG00000111674.8	32.474	7.055	2.055	6.25E-18
ENOX1	ENSG00000120658.12	0.51	2.06	-1.019	3.17E-13
ENPP3	ENSG00000154269.14	6.385	1.825	1.386	1.31E-06
ENPP6	ENSG00000164303.10	0.16	3.807	-2.051	1.27E-22

HS3ST1	ENSG00000002587.9	13.12	2.83	1.882	3.05E-11
HECW1	ENSG00000002746.14	0.485	2.313	-1.158	3.49E-07
MAD1L1	ENSG00000002822.15	29.598	10.69	1.388	1.12E-15
TMEM176A	ENSG00000002933.7	559.017	200.028	1.478	3.71E-06
KLHL13	ENSG00000003096.13	4.645	12.19	-1.224	4.01E-03
TFPI	ENSG00000003436.14	56.895	26.899	1.053	3.85E-05
SLC7A2	ENSG00000003989.16	0.68	4.344	-1.669	4.79E-10
HSPB6	ENSG00000004776.11	3.369	24.524	-2.547	2.85E-21
PDK4	ENSG00000004799.7	9.405	46.993	-2.206	1.90E-14
ZMYND10	ENSG00000004838.13	20.92	3.424	2.309	2.14E-11
SLC4A1	ENSG00000004939.13	0.03	50.229	-5.636	4.17E-99
PRSS22	ENSG00000005001.9	0.21	8.013	-2.897	7.67E-31
HOXA11	ENSG00000005073.5	0.56	2.123	-1.001	4.55E-03
THSD7A	ENSG00000005108.15	1.69	5.63	-1.301	4.27E-08
WDR54	ENSG00000005448.16	35.484	16.26	1.08	1.76E-08
ITGA3	ENSG00000005884.17	220.211	70.585	1.628	4.77E-15
TMEM98	ENSG00000006042.11	102.479	28.284	1.821	4.15E-15
TMEM132A	ENSG00000006118.14	19.685	6.825	1.402	2.17E-09
TNFRSF12A	ENSG00000006327.13	354.956	68.279	2.361	2.83E-31
USH1C	ENSG00000006611.15	107.761	47.392	1.168	2.25E-05
TBXA2R	ENSG00000006638.11	0.52	3.384	-1.528	3.40E-29
UPP2	ENSG00000007001.12	0.57	4.62	-1.84	1.20E-20
SLC13A2	ENSG00000007216.14	0.815	10.968	-2.721	4.15E-14
RHBDF1	ENSG00000007384.15	28.374	13.46	1.022	3.76E-14
SELE	ENSG00000007908.15	0.07	1.64	-1.303	3.01E-25
FMO3	ENSG00000007933.12	1.015	4.256	-1.383	8.43E-10
TFAP2B	ENSG00000008196.12	0	8.7	-3.278	5.25E-81
MGST1	ENSG00000008394.12	241.394	115.555	1.056	2.77E-03
IL32	ENSG00000008517.16	903.354	205.444	2.131	1.14E-15
IYD	ENSG00000009765.14	1.13	3.43	-1.056	4.21E-07
ETV7	ENSG00000010030.13	2.895	0.83	1.09	2.50E-11
SEMA3G	ENSG00000010319.6	1.27	18.312	-3.089	1.05E-53
PRSS3	ENSG00000010438.16	0.04	1.79	-1.424	1.16E-18
LRRC23	ENSG00000010626.14	27.855	12.879	1.056	9.27E-18
MRC2	ENSG00000011028.13	6.47	15.877	-1.176	7.83E-04
NME2	ENSG00000011052.21	22.305	6.955	1.551	2.50E-15
SYT7	ENSG00000011347.9	0.08	4.755	-2.414	1.74E-44
PLAUR	ENSG00000011422.11	13.55	5.335	1.2	1.67E-09
TYROBP	ENSG00000011600.11	144.542	27.049	2.375	5.46E-22
TMEM159	ENSG00000011638.10	37.36	17.945	1.018	2.55E-18
KDM5D	ENSG00000012817.15	1.015	9.103	-2.326	4.70E-14
MVP	ENSG00000013364.18	264.816	104.412	1.334	1.44E-22
GPRC5A	ENSG00000013588.5	2.65	8.27	-1.345	8.89E-04
WAS	ENSG00000015285.10	6.5	2.252	1.205	3.29E-10

XYLT2	ENSG00000015532.9	25.094	11.575	1.053	7.65E-21
VSIG2	ENSG00000019102.11	0.13	2.74	-1.727	3.29E-13
MARCO	ENSG00000019169.10	3.695	0.66	1.5	8.49E-09
SNAI2	ENSG00000019549.8	0.75	5.375	-1.865	6.24E-30
HGF	ENSG00000019991.15	1.04	3.737	-1.215	1.93E-10
SLC7A9	ENSG00000021488.12	2.295	11.368	-1.908	8.52E-08
FHL1	ENSG00000022267.16	349.62	34.48	3.305	4.85E-10
NLRP2	ENSG00000022556.15	0.295	1.745	-1.084	1.82E-03
GRAMD1B	ENSG00000023171.14	0.67	4.095	-1.609	5.46E-17
TYMP	ENSG00000025708.12	56.629	14.431	1.901	5.91E-14
VIM	ENSG00000026025.13	1115.171	337.348	1.722	3.42E-24
FAS	ENSG00000026103.19	15.155	6.785	1.053	2.71E-09
RNASET2	ENSG00000026297.15	143.573	33.371	2.073	6.00E-23
SLAMF7	ENSG00000026751.16	1.795	0.345	1.055	1.42E-05
PRKCH	ENSG00000027075.13	3.3	7.94	-1.056	3.34E-07
INSRR	ENSG00000027644.4	0	1.455	-1.296	4.96E-57
HMGB3	ENSG00000029993.14	39.22	14.655	1.361	1.29E-18
GRN	ENSG00000030582.16	415.023	162.415	1.348	7.41E-25
FUT8	ENSG00000033170.16	16.845	7.535	1.064	4.89E-15
TMSB10	ENSG00000034510.5	4592.295	841.838	2.446	6.23E-33
SH3YL1	ENSG00000035115.21	23.575	57.35	-1.248	2.27E-12
STAP1	ENSG00000035720.7	0.16	4.168	-2.156	2.76E-44
USP2	ENSG00000036672.15	11.15	38.08	-1.685	2.39E-06
FLT4	ENSG00000037280.15	1.31	10.385	-2.301	7.98E-30
VCAN	ENSG00000038427.15	67.827	5.06	3.506	1.20E-17
FAM65C	ENSG00000042062.11	2.115	11.929	-2.053	1.68E-20
LMO3	ENSG00000048540.14	0.14	2.145	-1.464	3.29E-44
EPN3	ENSG00000049283.17	0.7	9.84	-2.673	1.77E-25
LTBP1	ENSG00000049323.15	1.61	9.929	-2.066	1.21E-11
RCN1	ENSG00000049449.8	86.064	30.343	1.474	2.51E-25
RFC2	ENSG00000049541.10	21.51	10.06	1.025	5.92E-20
LAMC3	ENSG00000050555.17	2.135	11.892	-2.04	1.20E-15
PTGER3	ENSG00000050628.20	0.06	53.46	-5.683	4.08E-63
FSTL4	ENSG00000053108.16	0.11	1.935	-1.403	3.49E-23
NRIP2	ENSG00000053702.14	1.415	6.65	-1.663	1.37E-27
LAMA3	ENSG00000053747.15	17.57	2.578	2.376	4.78E-16
KCNQ1	ENSG00000053918.15	2.83	30.243	-3.028	3.66E-31
ENTPD2	ENSG00000054179.11	12.049	4.77	1.177	6.92E-07
KCNH2	ENSG00000055118.14	3.26	1.045	1.059	8.64E-08
MCOLN3	ENSG00000055732.12	0.425	3.612	-1.695	2.75E-13
LAMC2	ENSG00000058085.14	16.225	5.184	1.478	1.86E-04
YBX3	ENSG00000060138.12	267.834	131.152	1.025	8.01E-15
WNK1	ENSG00000060237.16	18.025	46.12	-1.308	6.95E-10
RIMBP2	ENSG00000060709.13	0.01	1.635	-1.383	4.47E-33

SPAG4	ENSG00000061656.9	19.71	4.29	1.969	2.47E-19
VMP1	ENSG00000062716.10	120.621	55.865	1.097	1.18E-09
POLD1	ENSG00000062822.12	17.78	7.605	1.126	3.81E-15
SPHK2	ENSG00000063176.15	10.065	23.845	-1.167	2.20E-10
LIMCH1	ENSG00000064042.17	7.635	18.894	-1.204	8.37E-05
ST3GAL6	ENSG00000064225.12	3.645	9.95	-1.237	1.20E-08
NGFR	ENSG00000064300.8	0.765	2.705	-1.07	2.32E-08
SLC9A3R2	ENSG00000065054.13	27.19	127.609	-2.19	3.40E-32
ERBB3	ENSG00000065361.14	59.089	25.995	1.154	1.59E-06
MYLK	ENSG00000065534.18	10.16	45.336	-2.054	1.14E-07
TIE1	ENSG00000066056.13	2.505	15.909	-2.27	7.07E-32
SLC9A3	ENSG00000066230.10	0.55	49.472	-5.025	4.99E-53
SPI1	ENSG00000066336.11	21.475	5.008	1.903	1.72E-18
MPPED2	ENSG00000066382.16	0.325	6.884	-2.573	3.68E-52
FGFR2	ENSG00000066468.20	8.315	17.774	-1.011	1.58E-03
KIF26A	ENSG00000066735.14	0.13	2.16	-1.484	3.36E-38
FECH	ENSG00000066926.10	10.67	26.172	-1.219	4.57E-09
PFKP	ENSG00000067057.16	109.758	40.866	1.404	2.02E-13
IDI1	ENSG00000067064.10	10.71	25.1	-1.156	4.49E-15
PPAP2A	ENSG00000067113.16	30.835	77.414	-1.301	3.10E-09
PKM	ENSG00000067225.17	1058.752	461.567	1.196	5.64E-14
ZFY	ENSG00000067646.11	0.13	1.604	-1.205	1.89E-12
RASGRP2	ENSG00000068831.18	2.39	7.304	-1.293	1.19E-18
TGFBR3	ENSG00000069702.10	4.195	12.632	-1.392	3.01E-12
SPTB	ENSG00000070182.17	0.19	1.64	-1.15	1.05E-56
WIPI1	ENSG00000070540.12	60.431	24.305	1.28	8.15E-25
SLC12A3	ENSG00000070915.9	0.1	72.143	-6.055	1.45E-66
TRIB2	ENSG00000071575.11	6.925	29.104	-1.926	1.88E-23
MYO3B	ENSG00000071909.18	0.02	2.769	-1.886	4.90E-39
LIMS2	ENSG00000072163.18	13.225	41.889	-1.592	9.35E-13
LNX1	ENSG00000072201.13	1.595	8.63	-1.892	5.75E-20
FCGR2B	ENSG00000072694.18	9.83	2.2	1.759	3.06E-10
MRVI1	ENSG00000072952.18	0.635	5.919	-2.081	5.36E-20
SCARB1	ENSG00000073060.15	33.445	7.96	1.943	4.82E-29
PANX2	ENSG00000073150.13	0.24	1.675	-1.109	5.26E-03
PTGS2	ENSG00000073756.11	0.27	2.355	-1.401	2.40E-19
ST6GAL1	ENSG00000073849.14	15.845	60.488	-1.868	6.22E-12
FRY	ENSG00000073910.19	5.48	15.103	-1.313	3.25E-09
NOTCH3	ENSG00000074181.8	2.915	19.429	-2.384	4.99E-10
NTN4	ENSG00000074527.11	25.64	66.995	-1.352	3.00E-05
SLC12A1	ENSG00000074803.17	0.23	201.515	-7.363	3.13E-68
SPAG5	ENSG00000076382.16	4.825	18.076	-1.711	1.53E-12
TPD52	ENSG00000076554.15	15.23	47.251	-1.572	2.60E-08
RAP1GAP	ENSG00000076864.19	15.725	65.673	-1.995	2.59E-19

UBE2T	ENSG00000077152.9	5.41	2.075	1.06	1.28E-17
PPP1R12B	ENSG00000077157.20	3.24	8.773	-1.205	3.29E-11
FBLN1	ENSG00000077942.17	5.91	33.276	-2.31	1.79E-14
ITGA8	ENSG00000077943.7	0.5	5.26	-2.061	1.96E-24
NEBL	ENSG00000078114.18	4.595	13.935	-1.416	2.52E-06
ITM2A	ENSG00000078596.10	3.27	15.69	-1.967	1.04E-06
SLC1A3	ENSG00000079215.13	3.02	0.77	1.184	3.24E-10
TNS1	ENSG00000079308.16	23.3	75.494	-1.654	1.77E-06
RAPGEF3	ENSG00000079337.15	16.08	50.748	-1.599	3.01E-18
SCGN	ENSG00000079689.13	4.96	0.72	1.793	1.71E-09
MOXD1	ENSG00000079931.14	3.31	11.945	-1.587	1.42E-07
SCTR	ENSG00000080293.9	0.53	4.77	-1.915	3.86E-33
MAGI3	ENSG00000081026.18	2.12	5.77	-1.118	4.83E-14
IL12RB2	ENSG00000081985.10	2	0.37	1.131	2.22E-06
TRPM3	ENSG00000083067.22	2.12	6.048	-1.176	5.49E-11
GRHL2	ENSG00000083307.10	0.02	3.928	-2.272	4.27E-36
FAT1	ENSG00000083857.13	145.764	60.761	1.249	8.94E-13
MECOM	ENSG00000085276.17	1.385	32.726	-3.822	7.09E-32
OVGP1	ENSG00000085465.12	4.8	1.79	1.056	8.04E-09
TTC39A	ENSG00000085831.15	31.41	10.755	1.463	3.63E-18
FOLH1	ENSG00000086205.16	0.54	4.775	-1.907	4.86E-16
SNX10	ENSG00000086300.15	45.044	17.875	1.287	2.17E-18
LAT2	ENSG00000086730.16	8.085	2.325	1.45	3.87E-18
HSD17B14	ENSG00000087076.8	9.779	29.82	-1.516	4.12E-03
MT3	ENSG00000087250.8	0.51	3.83	-1.677	1.14E-06
F11	ENSG00000088926.13	0.15	11.155	-3.402	2.02E-55
TESC	ENSG00000088992.17	43.487	12.729	1.696	2.04E-03
OAS1	ENSG00000089127.12	35.393	12.493	1.432	1.57E-17
FXYD5	ENSG00000089327.14	79.453	27.915	1.476	5.90E-11
FXYD3	ENSG00000089356.16	0.22	6.814	-2.679	9.72E-20
HEPH	ENSG00000089472.16	0.73	4.63	-1.702	1.51E-16
RGS1	ENSG00000090104.11	41.675	7.076	2.402	1.73E-17
LYZ	ENSG00000090382.6	44.079	9.253	2.136	3.41E-13
THPO	ENSG00000090534.17	0.15	1.795	-1.281	9.23E-04
NAT14	ENSG00000090971.4	38.1	14.543	1.331	3.82E-06
LAMB1	ENSG00000091136.13	254.134	95.772	1.399	7.45E-09
SLC26A4	ENSG00000091137.11	0.1	1.605	-1.244	6.65E-34
RAPGEF4	ENSG00000091428.17	0.44	3.069	-1.499	1.66E-26
SEL1L3	ENSG00000091490.10	66.576	12.64	2.309	1.07E-28
JPH4	ENSG00000092051.16	0.3	2.075	-1.242	2.34E-46
SLC7A8	ENSG00000092068.18	4.76	48.89	-3.115	6.04E-19
SEMA6A	ENSG00000092421.16	16.955	6.605	1.239	6.03E-10
TYRO3	ENSG00000092445.11	1.825	16.305	-2.615	1.27E-17
PHGDH	ENSG00000092621.11	13.265	47.911	-1.778	1.64E-08

EZR	ENSG00000092820.17	372.965	161.556	1.202	1.81E-15
FMO2	ENSG00000094963.13	1.535	5.975	-1.46	2.69E-06
EPB41L4B	ENSG00000095203.14	0.33	4.275	-1.988	9.26E-25
SH2D3C	ENSG00000095370.19	2.47	10.155	-1.685	8.72E-22
SMIM24	ENSG00000095932.6	96.085	230.687	-1.255	1.20E-05
TREM2	ENSG00000095970.16	26.695	0.82	3.928	3.74E-46
SCD	ENSG00000099194.5	19.654	4.95	1.796	1.90E-14
ERMP1	ENSG00000099219.13	9.94	25.595	-1.282	7.10E-06
MYO9B	ENSG00000099331.13	31.245	14.255	1.08	1.81E-12
HSD3B7	ENSG00000099377.13	51.69	19.705	1.348	3.59E-18
GADD45B	ENSG00000099860.8	32.919	102.764	-1.613	2.24E-08
PALM	ENSG00000099864.17	19.88	45.748	-1.163	1.43E-07
MMP11	ENSG00000099953.9	15.57	2.185	2.379	3.35E-21
SUSD2	ENSG00000099994.10	2.16	14.429	-2.288	2.87E-21
GGT5	ENSG00000099998.17	7.848	32.94	-1.94	3.60E-15
UPB1	ENSG00000100024.14	0.4	11.831	-3.196	8.65E-34
GGT1	ENSG00000100031.18	273.301	123.991	1.134	1.30E-05
LGALS1	ENSG00000100097.11	385.102	178.02	1.109	2.23E-10
KDELR3	ENSG00000100196.10	19.035	7.32	1.268	6.84E-13
TIMP3	ENSG00000100234.11	15.2	202.439	-3.651	2.54E-28
MIOX	ENSG00000100253.12	68.429	143.537	-1.058	7.15E-04
PDGFB	ENSG00000100311.16	4.715	13.16	-1.309	8.34E-11
PVALB	ENSG00000100362.12	0.44	49.371	-5.128	7.31E-31
KIAA0930	ENSG00000100364.18	34.35	16.635	1.003	1.66E-17
PYGL	ENSG00000100504.16	21.58	7.095	1.48	1.37E-15
TRIM9	ENSG00000100505.13	2.705	0.51	1.295	2.71E-17
SERPINA4	ENSG00000100665.11	0.245	2.425	-1.46	6.72E-15
PAPLN	ENSG00000100767.15	49.41	23.56	1.037	2.24E-06
REC8	ENSG00000100918.12	4.88	17.424	-1.648	3.02E-10
NFATC4	ENSG00000100968.13	7.055	15.419	-1.027	2.05E-07
PLTP	ENSG00000100979.14	105.094	43.767	1.245	8.84E-06
GSS	ENSG00000100983.9	97.369	35.96	1.412	9.74E-24
MMP9	ENSG00000100985.7	3.03	0.675	1.267	8.18E-07
SGK2	ENSG00000101049.14	16.115	39.852	-1.255	1.86E-08
MYBL2	ENSG00000101057.15	1.495	0.13	1.143	1.79E-12
SLC52A3	ENSG00000101276.14	0.47	8.65	-2.715	1.07E-21
SIRPB1	ENSG00000101307.15	2.585	0.52	1.238	2.13E-10
HCK	ENSG00000101336.12	7.725	2.275	1.414	1.46E-11
PPP1R16B	ENSG00000101445.9	0.975	6.828	-1.987	1.66E-20
SYNDIG1	ENSG00000101463.5	0.665	4.475	-1.717	3.07E-18
LIPG	ENSG00000101670.11	2.285	7.91	-1.44	1.07E-05
WDR13	ENSG00000101940.17	219.799	107.925	1.019	6.38E-14
SRPX	ENSG00000101955.14	0.585	2.585	-1.177	6.42E-10
SYP	ENSG00000102003.10	0.5	2.805	-1.343	3.78E-41

PLS3	ENSG00000102024.17	22.44	67.878	-1.555	6.26E-06
VGLL1	ENSG00000102243.12	0.035	1.304	-1.154	7.36E-18
TIMP1	ENSG00000102265.11	488.206	118.438	2.034	4.70E-20
GABRE	ENSG00000102287.16	0.55	3.085	-1.398	5.08E-08
SRPX2	ENSG00000102359.5	0.475	2.179	-1.108	1.50E-06
SYTL4	ENSG00000102362.15	3.384	8.205	-1.07	2.12E-14
FGF9	ENSG00000102678.6	0.26	7.745	-2.795	3.33E-44
FLT1	ENSG00000102755.10	2.715	14.162	-2.029	2.86E-16
RGCC	ENSG00000102760.12	7.715	22.614	-1.438	1.01E-13
OLFM4	ENSG00000102837.6	0.02	2.85	-1.916	5.24E-25
MSLN	ENSG00000102854.14	8.96	2.919	1.346	3.50E-03
HSF4	ENSG00000102878.15	30.424	11.895	1.285	1.27E-05
GDPD3	ENSG00000102886.14	2.27	7.208	-1.328	2.37E-11
VAC14	ENSG00000103043.14	50.59	24.16	1.036	1.20E-22
HCFC1R1	ENSG00000103145.10	299.237	123.716	1.267	2.37E-14
MPG	ENSG00000103152.11	61.479	29.248	1.047	7.97E-30
HAGHL	ENSG00000103253.17	15.799	5.214	1.435	4.52E-11
PYCARD	ENSG00000103490.13	29.258	7.665	1.804	7.01E-18
IGDCC4	ENSG00000103742.11	1.815	0.305	1.109	3.27E-12
FAM189A1	ENSG00000104059.4	7.75	1.18	2.005	3.89E-15
RHOV	ENSG00000104140.6	0.415	2.035	-1.101	3.57E-04
SFRP1	ENSG00000104332.11	0.3	78.825	-5.94	3.87E-64
PLAT	ENSG00000104368.17	5.695	61.284	-3.218	8.95E-37
WISP1	ENSG00000104415.13	0.56	3.315	-1.468	1.46E-17
SQLE	ENSG00000104549.11	3.195	9.305	-1.297	2.52E-07
TUSC3	ENSG00000104723.20	73.231	31.082	1.21	1.23E-18
MAN2B1	ENSG00000104774.12	75.53	27.759	1.412	3.58E-27
TUBB4A	ENSG00000104833.10	17.26	1.58	2.823	4.09E-20
IL4I1	ENSG00000104951.15	5.88	1.985	1.205	8.75E-11
TLE6	ENSG00000104953.18	0.485	2.232	-1.122	1.19E-12
NOVA2	ENSG00000104967.6	0.19	1.615	-1.136	7.99E-34
LILRB1	ENSG00000104972.14	2.62	0.8	1.008	1.11E-09
OLFM2	ENSG00000105088.8	8.03	2.25	1.474	2.10E-05
PRX	ENSG00000105227.14	1.17	3.935	-1.185	1.27E-25
SIGLEC8	ENSG00000105366.15	1.4	0.04	1.206	5.48E-15
RPS19	ENSG00000105372.6	2160.35	947.872	1.188	1.56E-17
MEIS3	ENSG00000105419.17	6.475	1.99	1.322	7.21E-05
LIG1	ENSG00000105486.13	19.56	7.989	1.194	5.90E-13
RASIP1	ENSG00000105538.8	1.82	9.06	-1.835	1.45E-19
PLEKHA4	ENSG00000105559.11	35.5	11.771	1.515	3.51E-13
JAK3	ENSG00000105639.18	4.155	1.235	1.206	1.60E-05
PDE4C	ENSG00000105650.21	0.325	1.764	-1.061	3.80E-16
HAMP	ENSG00000105697.7	8.67	0.19	3.023	2.80E-34
SCN1B	ENSG00000105711.10	11.735	5.1	1.062	8.73E-08

GRIK5	ENSG00000105737.9	0.335	6.175	-2.426	4.89E-18
TFPI2	ENSG00000105825.11	168.697	7.272	4.359	3.25E-14
PON3	ENSG00000105852.10	0.04	1.615	-1.33	4.08E-09
ITGB8	ENSG00000105855.9	62.515	14.475	2.037	9.26E-13
PTN	ENSG00000105894.11	3.575	11.91	-1.497	9.56E-03
MET	ENSG00000105976.14	81.848	14.052	2.461	2.50E-32
HOXA3	ENSG00000105997.22	18.425	4.96	1.705	2.48E-10
HSPB1	ENSG00000106211.8	1536.997	609.506	1.333	2.18E-14
NUDT1	ENSG00000106268.15	22.74	7.445	1.491	5.58E-26
SERPINE1	ENSG00000106366.8	4.529	16.686	-1.678	6.72E-05
PLOD3	ENSG00000106397.11	79.404	28.52	1.446	3.06E-24
TMEM176B	ENSG00000106565.17	721.046	271.978	1.403	2.72E-07
LIMK1	ENSG00000106683.14	19.68	8.199	1.169	8.32E-26
PRUNE2	ENSG00000106772.17	44.89	11.74	1.849	7.85E-12
OGN	ENSG00000106809.10	0.04	1.86	-1.459	6.84E-18
TYRP1	ENSG00000107165.12	0.03	3.465	-2.116	1.60E-43
SH3GL2	ENSG00000107295.9	2.605	6.445	-1.046	2.97E-04
PTGDS	ENSG00000107317.11	5.765	181.427	-4.753	2.15E-16
PDLIM1	ENSG00000107438.8	103.414	44.084	1.212	1.12E-10
GATA3	ENSG00000107485.15	0.445	21.56	-3.965	7.61E-36
UNC5B	ENSG00000107731.12	1.485	5.494	-1.386	5.56E-05
SPOCK2	ENSG00000107742.12	6.52	39.055	-2.413	7.99E-15
LIPA	ENSG00000107798.17	58.965	27.175	1.09	5.50E-16
PBLD	ENSG00000108187.15	17.865	43.629	-1.242	2.63E-07
4-Sep	ENSG00000108387.14	3.525	16.36	-1.94	2.33E-23
HOXB6	ENSG00000108511.9	7.4	23.93	-1.569	2.76E-10
PFN1	ENSG00000108518.7	688.254	339.495	1.017	1.85E-17
RASD1	ENSG00000108551.4	1.22	95.713	-5.445	1.98E-37
LGALS3BP	ENSG00000108679.12	442.121	194.549	1.18	4.49E-15
SGCA	ENSG00000108823.15	0.49	2.03	-1.024	1.28E-13
VAT1	ENSG00000108828.15	211.431	77.834	1.43	5.85E-33
FAM20A	ENSG00000108950.11	5.43	14.267	-1.247	5.14E-04
RAB34	ENSG00000109113.17	143.508	60.031	1.244	3.06E-08
USP46	ENSG00000109189.12	2.745	6.625	-1.026	6.53E-10
PF4V1	ENSG00000109272.3	3.895	0.36	1.848	3.99E-09
GLRB	ENSG00000109738.10	9.275	2.38	1.604	6.50E-11
HGFAC	ENSG00000109758.8	3.745	0.53	1.633	1.07E-07
HTATIP2	ENSG00000109854.13	53.509	21.79	1.258	6.43E-14
LPXN	ENSG00000110031.12	13.11	5.62	1.092	3.04E-13
UNC93B1	ENSG00000110057.7	29.431	13.45	1.074	7.77E-15
MS4A6A	ENSG00000110077.14	37.625	12.685	1.497	1.51E-11
MS4A4A	ENSG00000110079.16	7.16	2.649	1.161	1.16E-06
ST3GAL4	ENSG00000110080.18	14.305	31.715	-1.096	9.43E-04
FOLR3	ENSG00000110203.8	0.21	14.485	-3.678	1.26E-56

IL10RA	ENSG00000110324.9	6.335	2.425	1.099	2.36E-07
GALNT18	ENSG00000110328.5	8.015	21.62	-1.327	5.27E-09
SLC15A3	ENSG00000110446.9	42.845	16.53	1.323	5.15E-10
MDK	ENSG00000110492.15	99.95	20.175	2.253	1.00E-10
SELPLG	ENSG00000110876.9	10.01	2.19	1.787	1.54E-15
KRT18	ENSG00000111057.10	624.236	200.867	1.631	1.84E-16
TNS2	ENSG00000111077.17	20.624	63.846	-1.584	1.53E-20
TRPV4	ENSG00000111199.10	5.455	15.044	-1.314	1.53E-06
MGP	ENSG00000111341.9	69.84	429.555	-2.604	3.04E-12
SLC38A1	ENSG00000111371.15	31.806	13.19	1.209	2.76E-12
RERGL	ENSG00000111404.6	0.305	8.77	-2.904	1.09E-49
VDR	ENSG00000111424.10	3.435	9.12	-1.19	7.63E-07
GAPDH	ENSG00000111640.14	4130.64	2009.888	1.039	1.16E-18
PTPN6	ENSG00000111679.16	48.01	20.698	1.176	8.55E-19
NEDD9	ENSG00000111859.16	10.825	25.926	-1.187	3.17E-07
FAM184A	ENSG00000111879.18	0.795	3.185	-1.221	4.84E-23
MAN1A1	ENSG00000111885.6	13.125	33.935	-1.306	3.42E-05
NCOA7	ENSG00000111912.18	13.535	30.497	-1.116	5.81E-08
PHACTR1	ENSG00000112137.16	2.12	5.54	-1.068	1.27E-05
FHL5	ENSG00000112214.10	0.18	2.9	-1.725	1.07E-24
SIM1	ENSG00000112246.9	1.825	5.27	-1.15	4.68E-09
EYA4	ENSG00000112319.17	0.025	1.94	-1.52	2.60E-41
SLC17A2	ENSG00000112337.10	1.495	0.145	1.124	3.04E-12
PERP	ENSG00000112378.11	43.779	16.439	1.361	1.40E-08
PACRG	ENSG00000112530.11	2.545	7.584	-1.276	5.63E-13
PDE10A	ENSG00000112541.13	0.16	2.26	-1.491	1.79E-09
MDFI	ENSG00000112559.13	0.43	1.925	-1.032	4.26E-07
SMOC2	ENSG00000112562.18	1.73	5.025	-1.142	5.77E-07
VEGFA	ENSG00000112715.20	21.29	77.203	-1.811	8.60E-08
LY86	ENSG00000112799.8	9.95	1.42	2.178	1.18E-22
GHR	ENSG00000112964.13	0.595	4.1	-1.677	2.68E-28
HBEGF	ENSG00000113070.7	3.91	9.28	-1.066	1.24E-08
SLC4A9	ENSG00000113073.14	0.05	12.559	-3.691	5.42E-96
HAVCR1	ENSG00000113249.12	29.105	2.584	3.07	1.71E-17
RASGRF2	ENSG00000113319.11	0.39	3.14	-1.575	1.05E-24
PRLR	ENSG00000113494.16	0.45	2.8	-1.39	4.01E-08
PCDH12	ENSG00000113555.5	0.935	4.735	-1.567	3.98E-25
FGF1	ENSG00000113578.17	0.615	34.862	-4.473	3.83E-36
LIFR	ENSG00000113594.9	4.99	11.05	-1.008	1.83E-03
PDGFRB	ENSG00000113721.13	3.99	25.925	-2.432	5.27E-19
KNG1	ENSG00000113889.11	0.03	210.1	-7.679	9.48E-84
HRG	ENSG00000113905.4	0.01	14.468	-3.937	2.81E-64
NIT2	ENSG00000114021.11	152.439	59.071	1.353	1.09E-10
RBP2	ENSG00000114113.6	0	1.244	-1.166	2.16E-41

WNT5A	ENSG00000114251.13	5.06	1.29	1.404	4.18E-09
USP9Y	ENSG00000114374.12	0.44	8.53	-2.726	9.15E-15
HYAL1	ENSG00000114378.16	21.29	45.065	-1.047	1.36E-05
HHLA2	ENSG00000114455.13	16.335	1.915	2.572	9.11E-09
UPK1B	ENSG00000114638.7	9.295	2.247	1.665	3.60E-03
PLCH1	ENSG00000114805.16	3.3	0.805	1.252	1.43E-15
VIPR1	ENSG00000114812.12	0.24	1.53	-1.029	8.81E-17
TNNC1	ENSG00000114854.7	0.755	12.065	-2.896	2.14E-38
TFCP2L1	ENSG00000115112.7	4.435	39.759	-2.907	8.05E-26
FNDC4	ENSG00000115226.9	8.295	3.195	1.148	6.45E-04
PDE1A	ENSG00000115252.18	0.775	24.883	-3.866	1.68E-19
REEP6	ENSG00000115255.10	5.83	13.949	-1.13	1.56E-06
GRB14	ENSG00000115290.9	5.93	27.284	-2.029	9.75E-18
GALNT3	ENSG00000115339.13	2.945	9.108	-1.357	3.17E-07
IGFBP2	ENSG00000115457.9	16.935	123.023	-2.79	4.63E-19
IGFBP5	ENSG00000115461.4	15.38	227.142	-3.8	1.43E-27
PLCD4	ENSG00000115556.13	2.13	5.38	-1.027	2.32E-06
IL1R2	ENSG00000115590.13	0.925	4.67	-1.558	2.61E-12
IL1RL1	ENSG00000115602.16	0.615	15.832	-3.382	1.68E-29
SLC9A2	ENSG00000115616.2	0.235	2.669	-1.571	1.92E-19
QPCT	ENSG00000115828.15	17.035	4.68	1.667	5.13E-06
PLCL1	ENSG00000115896.15	1.58	11.153	-2.236	2.46E-18
PLEK	ENSG00000115956.9	5.645	1.565	1.374	1.12E-11
RND3	ENSG00000115963.13	19.82	8.54	1.126	5.56E-13
EPAS1	ENSG00000116016.13	23.72	111.766	-2.19	3.42E-18
PAPPA2	ENSG00000116183.10	0.03	3.467	-2.117	3.63E-20
NPHS2	ENSG00000116218.12	0	105.404	-6.733	1.04E-64
ERRFI1	ENSG00000116285.12	12.095	30.715	-1.276	4.85E-08
KCNC4	ENSG00000116396.13	1.155	6.285	-1.757	4.69E-24
NCF2	ENSG00000116701.14	6.1	1.805	1.34	5.44E-13
WLS	ENSG00000116729.13	24.535	68.741	-1.45	8.76E-07
OLFML3	ENSG00000116774.11	5.315	18.109	-1.597	7.65E-12
HAO2	ENSG00000116882.14	4.995	42.499	-2.859	6.26E-14
HPCAL4	ENSG00000116983.12	0.03	2.189	-1.63	3.10E-34
ST6GALNAC5	ENSG00000117069.14	2.035	0.145	1.406	8.17E-07
PLA2G2D	ENSG00000117215.14	1.15	0.03	1.062	2.81E-09
TSPAN1	ENSG00000117472.9	508.321	248.568	1.029	7.41E-03
F3	ENSG00000117525.13	1.34	10.08	-2.244	8.94E-18
HSD11B1	ENSG00000117594.9	0.385	2.072	-1.15	4.62E-15
MAN1C1	ENSG00000117643.14	2.33	29.38	-3.19	6.44E-26
TNNT2	ENSG00000118194.18	0.21	20.609	-4.159	2.44E-60
TTR	ENSG00000118271.9	0	1.525	-1.336	3.44E-16
SGIP1	ENSG00000118473.21	0.595	6.265	-2.187	3.89E-22
TCF21	ENSG00000118526.6	0.395	14.545	-3.478	3.29E-52

SLC16A7	ENSG00000118596.11	2.125	5.43	-1.041	4.84E-04
SPP1	ENSG00000118785.13	4342.168	1549.137	1.486	2.84E-10
SATB2	ENSG00000119042.16	10	2.84	1.518	4.30E-10
GDA	ENSG00000119125.16	39.86	10.95	1.774	3.52E-05
NEK6	ENSG00000119408.16	23.59	10.884	1.049	2.52E-13
NR4A3	ENSG00000119508.17	0.38	3.065	-1.558	3.98E-17
GALNT12	ENSG00000119514.6	8.649	3.745	1.024	4.63E-14
IFI27L2	ENSG00000119632.3	97.102	35.768	1.416	6.76E-13
LTBP2	ENSG00000119681.11	1.16	4.954	-1.463	1.28E-04
ESRRB	ENSG00000119715.14	0.125	11.374	-3.459	1.38E-69
EPCAM	ENSG00000119888.10	92.892	191.353	-1.035	1.33E-04
TEK	ENSG00000120156.20	0.765	16.284	-3.292	1.75E-54
MYCT1	ENSG00000120279.6	0.61	4.855	-1.863	2.50E-30
PLXDC2	ENSG00000120594.16	16.31	5.538	1.405	6.63E-20
IQSEC3	ENSG00000120645.11	0.34	2.705	-1.467	4.42E-12
TGFBI	ENSG00000120708.16	79.291	33.229	1.23	2.71E-08
PLS1	ENSG00000120756.12	27.83	9.857	1.409	4.10E-10
GLT8D2	ENSG00000120820.12	0.635	2.414	-1.062	3.12E-06
SOCS2	ENSG00000120833.13	3.25	17.273	-2.104	1.36E-24
TNFRSF10B	ENSG00000120889.12	42.299	15.341	1.406	1.85E-25
PDLIM2	ENSG00000120913.23	14.395	51.622	-1.773	2.31E-15
TBX2	ENSG00000121068.13	2.3	30.749	-3.266	1.53E-32
TNFSF10	ENSG00000121858.10	198.975	65.853	1.581	2.47E-06
TMEM54	ENSG00000121900.18	98.243	30.643	1.649	8.58E-20
HPCA	ENSG00000121905.9	2.05	0.325	1.203	5.94E-15
XPNPEP2	ENSG00000122121.10	0.245	18.674	-3.982	5.15E-34
SASH3	ENSG00000122122.9	5.315	1.545	1.311	6.42E-11
PLG	ENSG00000122194.18	0.64	36.486	-4.515	1.65E-44
HS3ST2	ENSG00000122254.6	1.93	0.065	1.46	1.38E-11
PTGFR	ENSG00000122420.9	0.22	1.875	-1.237	4.59E-09
RAMP3	ENSG00000122679.8	5.6	48.65	-2.911	4.99E-39
NUDT10	ENSG00000122824.10	0.15	1.619	-1.188	8.01E-24
ITIH5	ENSG00000123243.14	1.96	17.272	-2.626	1.15E-13
NCKAP1L	ENSG00000123338.12	4.34	1.16	1.306	2.55E-11
NR4A1	ENSG00000123358.19	10.09	172.349	-3.966	8.08E-18
HOXC11	ENSG00000123388.4	1.955	0.435	1.042	4.11E-08
TUBA1B	ENSG00000123416.15	509.344	224.216	1.18	2.60E-11
IL13RA2	ENSG00000123496.7	0.06	1.97	-1.486	5.62E-40
NRK	ENSG00000123572.16	0.06	3.22	-1.993	2.90E-21
TNFAIP6	ENSG00000123610.4	14.92	0.28	3.637	3.92E-18
G0S2	ENSG00000123689.5	16.774	63.742	-1.865	5.82E-09
PI3	ENSG00000124102.4	2.85	0.5	1.36	1.64E-07
KCNS1	ENSG00000124134.8	1.385	0.11	1.103	4.67E-07
SLC12A5	ENSG00000124140.12	3.24	0.66	1.353	1.31E-10

PTGIS	ENSG00000124212.5	0.65	2.735	-1.179	1.86E-04
SNAI1	ENSG00000124216.3	0.78	3.195	-1.237	1.50E-10
PCK1	ENSG00000124253.10	7.914	126.763	-3.841	2.57E-20
HIF3A	ENSG00000124440.15	0.36	2.46	-1.347	1.26E-22
F13A1	ENSG00000124491.15	2.185	7.932	-1.488	6.81E-09
TREM1	ENSG00000124731.12	2.275	0.585	1.047	5.02E-09
SOX4	ENSG00000124766.5	32.27	10.134	1.579	1.17E-14
NRN1	ENSG00000124785.8	0.815	4.605	-1.627	4.81E-33
SH3TC1	ENSG00000125089.16	10.295	25.439	-1.227	6.51E-12
MT1G	ENSG00000125144.13	6.565	1307.965	-7.435	4.16E-48
MT2A	ENSG00000125148.6	102.685	340.592	-1.72	4.17E-08
6-Sep	ENSG00000125354.22	10.635	22.755	-1.03	9.53E-07
SOX9	ENSG00000125398.5	37.724	7.758	2.145	1.57E-13
HS3ST3B1	ENSG00000125430.8	0.17	2.015	-1.366	3.77E-19
TNFSF9	ENSG00000125657.4	6.545	0.3	2.537	1.10E-40
FOSB	ENSG00000125740.13	1.7	6.774	-1.526	7.97E-04
SDCBP2	ENSG00000125775.14	2.24	5.49	-1.002	5.24E-09
FLRT3	ENSG00000125848.9	33.314	14.255	1.17	8.18E-04
LRRN4	ENSG00000125872.7	20.672	1.662	3.025	2.53E-18
MMP24	ENSG00000125966.9	15.85	6.583	1.152	7.89E-11
PROZ	ENSG00000126231.13	0.06	3.994	-2.236	8.21E-34
HCST	ENSG00000126264.9	17.3	6.26	1.334	7.48E-11
FLRT1	ENSG00000126500.3	0.06	1.49	-1.232	2.79E-47
WNK4	ENSG00000126562.16	0.76	26.109	-3.945	1.38E-52
RHOJ	ENSG00000126785.12	1.16	3.359	-1.013	2.82E-13
HSPA2	ENSG00000126803.9	4.365	31.26	-2.588	4.94E-23
EVI2A	ENSG00000126860.11	4.43	1.34	1.214	1.13E-12
FGD3	ENSG00000127084.17	2.24	6.089	-1.13	5.32E-10
TRAF2	ENSG00000127191.17	14.375	6.34	1.067	8.86E-19
MASP1	ENSG00000127241.16	15.11	1.809	2.52	8.31E-20
TSPAN8	ENSG00000127324.8	0.9	24.384	-3.74	9.22E-22
PTPRB	ENSG00000127329.14	1.41	13.373	-2.576	3.52E-37
LRRC61	ENSG00000127399.14	27.84	10.79	1.291	6.51E-18
PKMYT1	ENSG00000127564.16	3.975	0.565	1.669	8.12E-17
FBXL16	ENSG00000127585.11	27.474	8.249	1.622	1.58E-03
VIL1	ENSG00000127831.10	1.785	6.102	-1.351	1.03E-05
SHFM1	ENSG00000127922.9	232.099	88.401	1.383	4.13E-20
FGL2	ENSG00000127951.5	6.968	24.468	-1.676	3.90E-04
STEAP4	ENSG00000127954.12	0.38	1.765	-1.003	1.35E-11
RASL11B	ENSG00000128045.6	0.18	14.074	-3.675	6.44E-83
KDR	ENSG00000128052.8	1.33	10.883	-2.35	1.72E-22
MGAT3	ENSG00000128268.11	0.31	2.005	-1.198	1.25E-09
TPST2	ENSG00000128294.15	7.055	23.435	-1.601	5.66E-15
RAC2	ENSG00000128340.14	23.124	4.58	2.112	1.65E-16

KRT17	ENSG00000128422.15	4.395	1.33	1.211	6.83E-03
PODXL	ENSG00000128567.16	4.53	103.971	-4.247	1.31E-43
FLNC	ENSG00000128591.15	4.92	1.89	1.034	1.13E-06
LRRC4	ENSG00000128594.7	0.25	1.99	-1.258	1.44E-19
HOXD1	ENSG00000128645.12	0.765	3.265	-1.273	7.63E-08
HOXD3	ENSG00000128652.11	1.075	8.53	-2.199	7.71E-30
GAD1	ENSG00000128683.13	0.19	2.79	-1.671	7.10E-21
HOXD9	ENSG00000128709.11	5.215	17.168	-1.548	8.83E-17
HOXD10	ENSG00000128710.5	0.33	17.407	-3.791	3.87E-35
HOXD11	ENSG00000128713.12	0.16	3.29	-1.887	1.35E-19
ISLR	ENSG00000129009.12	3.59	13.209	-1.63	2.48E-06
PLD2	ENSG00000129219.13	20.935	8.39	1.224	1.10E-10
MTUS1	ENSG00000129422.13	6.44	16.449	-1.23	2.26E-10
RIPK3	ENSG00000129465.15	1.305	3.82	-1.064	5.40E-09
RNASE1	ENSG00000129538.13	42.47	192.738	-2.156	5.92E-12
RHBDF2	ENSG00000129667.12	30.09	8.27	1.746	6.01E-30
MAP7D3	ENSG00000129680.15	8.53	2.74	1.349	5.03E-21
RPS4Y1	ENSG00000129824.15	17.904	202.797	-3.43	1.99E-09
LBP	ENSG00000129988.5	0.055	1.339	-1.149	6.88E-11
TNNI3	ENSG00000129991.12	2.645	0.195	1.609	3.90E-07
NXNL2	ENSG00000130045.15	6.34	2.19	1.202	7.32E-06
STARD8	ENSG00000130052.13	3.26	12.635	-1.678	6.70E-18
SH3BP4	ENSG00000130147.15	6.735	23.205	-1.646	4.69E-12
LDLR	ENSG00000130164.11	0.915	9.598	-2.468	1.92E-38
EXOC3L2	ENSG00000130201.7	1.745	17.095	-2.721	7.71E-36
GADD45G	ENSG00000130222.10	5.265	11.916	-1.044	1.20E-10
PLVAP	ENSG00000130300.8	16.32	83.075	-2.279	7.09E-15
USHBP1	ENSG00000130307.11	0.57	4.41	-1.785	1.28E-52
PXDN	ENSG00000130508.10	28.789	4.765	2.369	5.10E-20
HRC	ENSG00000130528.11	0.43	2.725	-1.381	2.29E-32
LSP1	ENSG00000130592.13	30.011	4.444	2.51	2.44E-23
H19	ENSG00000130600.15	24.975	106.944	-2.055	2.85E-12
GATA5	ENSG00000130700.6	0.03	1.49	-1.274	2.63E-32
SMPDL3B	ENSG00000130768.14	15.17	4.874	1.461	8.69E-17
THEMIS2	ENSG00000130775.15	20.754	5.085	1.838	6.67E-15
ZNF331	ENSG00000130844.16	4.88	11.329	-1.068	6.64E-10
LRP3	ENSG00000130881.13	4.099	10.449	-1.167	9.88E-03
TXLNGY	ENSG00000131002.11	0.765	7.94	-2.341	7.80E-11
SYNE1	ENSG00000131018.22	21.41	45.686	-1.059	5.78E-05
GINS2	ENSG00000131153.8	2.38	0.505	1.167	1.29E-20
SLC34A1	ENSG00000131183.10	0.715	29.025	-4.13	1.65E-24
F12	ENSG00000131187.9	8.405	1.49	1.917	8.83E-17
PRR7	ENSG00000131188.11	3.45	0.68	1.405	3.45E-21
SH3BP5	ENSG00000131370.15	6.919	32.51	-2.081	1.39E-13

RFTN1	ENSG00000131378.13	4.625	11.295	-1.128	1.60E-03
GALNT15	ENSG00000131386.17	0.615	4.955	-1.883	2.59E-38
KCNC3	ENSG00000131398.13	2.515	7.798	-1.324	8.41E-13
NAPSB	ENSG00000131401.11	15.865	4.105	1.724	5.16E-12
PDLIM4	ENSG00000131435.12	2.985	12.666	-1.778	2.39E-05
PSMC3IP	ENSG00000131470.14	6.87	2.695	1.091	1.83E-11
RAMP2	ENSG00000131477.10	10.51	46.662	-2.05	4.23E-16
G6PC	ENSG00000131482.9	0.13	6.85	-2.796	3.17E-23
TMEM204	ENSG00000131634.13	3.63	26.094	-2.549	8.28E-31
THOC6	ENSG00000131652.13	37.054	15.088	1.242	3.06E-27
TOP2A	ENSG00000131747.14	3.29	0.415	1.6	1.48E-17
PPP1R1B	ENSG00000131771.13	0.08	4.445	-2.334	2.97E-24
NR0B2	ENSG00000131910.4	0.03	10.845	-3.524	8.16E-33
GCH1	ENSG00000131979.18	2.845	7.595	-1.16	1.67E-21
LGALS3	ENSG00000131981.15	223.672	97.615	1.188	8.27E-08
IQCA1	ENSG00000132321.16	10.48	4.51	1.059	5.91E-04
RAMP1	ENSG00000132329.10	1.315	9.905	-2.236	1.56E-16
ITGB4	ENSG00000132470.13	49.391	12.32	1.92	1.28E-10
XAF1	ENSG00000132530.16	3.465	8.548	-1.097	5.36E-05
MTSS1L	ENSG00000132613.14	5.65	15.4	-1.302	1.61E-05
HSPA12B	ENSG00000132622.10	0.8	5.71	-1.898	3.05E-46
SNAP25	ENSG00000132639.12	5.36	0.73	1.878	5.77E-15
RHBG	ENSG00000132677.12	0.19	19.072	-4.076	1.04E-46
NES	ENSG00000132688.10	3.22	23.516	-2.538	3.06E-33
RAB25	ENSG00000132698.13	0.5	40.065	-4.775	3.15E-18
VSTM2L	ENSG00000132821.11	8.529	0.535	2.634	2.18E-10
PRAM1	ENSG00000133246.11	3.475	0.69	1.405	1.07E-16
PDE6B	ENSG00000133256.12	17.27	6.991	1.193	6.74E-09
MYH11	ENSG00000133392.16	2.465	29.635	-3.144	1.51E-15
PDZD2	ENSG00000133401.15	0.405	3.915	-1.807	2.39E-33
GSTT2B	ENSG00000133433.10	2.595	9.958	-1.608	6.60E-09
FAM83F	ENSG00000133477.16	0.415	3.065	-1.522	7.99E-09
GIMAP6	ENSG00000133561.15	2.37	10.79	-1.807	1.13E-12
TMTC1	ENSG00000133687.15	0.85	7.225	-2.153	1.12E-30
LYVE1	ENSG00000133800.8	0.775	7.435	-2.249	5.41E-25
PEBP4	ENSG00000134020.7	0.215	1.553	-1.071	2.01E-11
MEIS2	ENSG00000134138.19	1.635	13.11	-2.421	2.84E-23
TSPAN2	ENSG00000134198.9	0.425	3.712	-1.726	2.97E-26
GSTM5	ENSG00000134201.10	0.4	3.26	-1.605	1.56E-22
GSTM3	ENSG00000134202.10	36.661	146.012	-1.965	4.29E-16
HMGCS2	ENSG00000134240.11	0.13	58.328	-5.714	7.05E-52
PTGFRN	ENSG00000134247.9	53.693	5.809	3.006	9.96E-35
VTCN1	ENSG00000134258.16	2.91	11.199	-1.642	1.35E-05
FST	ENSG00000134363.11	0.085	1.305	-1.087	7.10E-10

IL15RA	ENSG00000134470.19	2.32	6.424	-1.161	1.59E-03
FADS2	ENSG00000134824.13	3.46	9.611	-1.25	6.85E-03
PDGFRA	ENSG00000134853.11	0.45	8.355	-2.69	2.67E-31
GGACT	ENSG00000134864.10	3.45	11.256	-1.462	7.44E-21
KDELC1	ENSG00000134901.12	15.22	3.13	1.974	1.41E-25
SLC37A2	ENSG00000134955.11	4.43	0.67	1.701	4.13E-15
FAM189A2	ENSG00000135063.17	1.54	4.19	-1.031	6.93E-11
PSAT1	ENSG00000135069.13	7.675	31.333	-1.898	9.50E-08
SDS	ENSG00000135094.10	7.17	0.29	2.663	1.17E-27
TBX3	ENSG00000135111.14	0.67	5.69	-2.002	2.19E-36
RNFT2	ENSG00000135119.14	3.92	1.145	1.198	8.21E-18
UGT2A3	ENSG00000135220.10	42.111	13.104	1.612	2.83E-03
KCP	ENSG00000135253.13	18.285	7.264	1.223	4.54E-06
TES	ENSG00000135269.17	52.539	18.724	1.441	2.84E-13
MRAP2	ENSG00000135324.5	0.27	2.204	-1.335	5.08E-09
EPHA7	ENSG00000135333.13	8.04	2.47	1.382	7.87E-06
LMO2	ENSG00000135363.11	12.2	26.355	-1.051	8.55E-12
RDH5	ENSG00000135437.9	34.642	10.58	1.622	7.99E-09
PKIB	ENSG00000135549.14	6.09	1.695	1.396	4.28E-15
MDM2	ENSG00000135679.21	54.964	19.104	1.477	1.61E-27
ITM2C	ENSG00000135916.15	91.887	186.443	-1.013	1.50E-03
SERPINE2	ENSG00000135919.12	202.474	27	2.861	2.09E-21
SCEL	ENSG00000136155.16	3.705	0.175	2.002	2.21E-09
LCP1	ENSG00000136167.13	14.095	6.15	1.078	9.21E-09
GPNMB	ENSG00000136235.15	112.762	23.77	2.199	4.55E-12
RTP4	ENSG00000136514.2	7.704	2.53	1.302	4.04E-09
GALNT5	ENSG00000136542.8	2.01	0.03	1.547	5.78E-16
HS6ST1	ENSG00000136720.6	10.52	41.873	-1.896	5.59E-20
TMOD1	ENSG00000136842.13	1.255	4.955	-1.401	2.27E-05
LMX1B	ENSG00000136944.17	0.06	1.815	-1.409	8.30E-24
MYC	ENSG00000136997.14	28.023	12.475	1.107	2.73E-08
GMPR	ENSG00000137198.9	10.905	23.506	-1.042	3.69E-12
TFAP2A	ENSG00000137203.10	0.17	7.74	-2.901	8.86E-26
SLC22A7	ENSG00000137204.14	0.815	15.567	-3.19	7.96E-11
SLC22A23	ENSG00000137266.14	1.055	6.246	-1.818	6.91E-22
IER3	ENSG00000137331.11	36.395	92.825	-1.327	2.66E-05
NRM	ENSG00000137404.14	21.29	8.06	1.299	3.57E-16
SYTL2	ENSG00000137501.16	59.55	17.718	1.694	5.18E-07
LRRC32	ENSG00000137507.11	12.32	43.655	-1.745	1.48E-15
TMPRSS4	ENSG00000137648.16	0.25	13.551	-3.541	8.39E-29
MMP7	ENSG00000137673.8	382.521	81.965	2.209	3.78E-07
FXYD6	ENSG00000137726.15	13.82	47.535	-1.712	1.06E-13
THBS1	ENSG00000137801.10	19.845	59.51	-1.537	6.89E-04
ITGA11	ENSG00000137809.16	0.43	3.07	-1.509	2.32E-09

PAQR5	ENSG00000137819.13	11.13	29.568	-1.333	1.09E-05
PLCB2	ENSG00000137841.11	8.725	2.925	1.309	9.05E-08
PAK6	ENSG00000137843.11	0.11	4.205	-2.229	3.18E-42
STRA6	ENSG00000137868.18	0.16	3.005	-1.788	2.83E-15
SEMA6D	ENSG00000137872.15	0.97	6.709	-1.969	3.62E-20
KHK	ENSG00000138030.12	17.08	121.713	-2.763	2.75E-10
SLC3A1	ENSG00000138079.13	365.226	180.743	1.011	3.54E-04
PLCE1	ENSG00000138193.14	1.545	4.829	-1.196	5.02E-06
PLA2G12B	ENSG00000138308.5	0.125	1.81	-1.321	4.57E-06
LEF1	ENSG00000138795.9	0.895	4.53	-1.545	1.67E-13
HADH	ENSG00000138796.15	33.38	72.086	-1.088	3.22E-08
SLC39A8	ENSG00000138821.12	20.589	7.865	1.284	4.93E-07
MTTP	ENSG00000138823.12	0.06	1.49	-1.232	1.39E-32
PARVG	ENSG00000138964.16	9.175	2.125	1.703	1.47E-11
ERP27	ENSG00000139055.6	1.52	19.255	-3.007	5.31E-32
GABARAPL1	ENSG00000139112.10	94.506	195.299	-1.039	1.38E-07
PIK3C2G	ENSG00000139144.9	0	2.57	-1.836	2.17E-43
PRICKLE1	ENSG00000139174.10	8.28	3.143	1.163	4.73E-07
RBP5	ENSG00000139194.7	75.695	225.127	-1.56	3.45E-06
SLC38A4	ENSG00000139209.15	0.285	1.685	-1.063	1.42E-05
LUM	ENSG00000139329.4	18.189	95.666	-2.333	6.42E-09
SLC15A4	ENSG00000139370.10	24.285	10.78	1.102	4.20E-25
FAM222A	ENSG00000139438.5	1.015	6.829	-1.958	1.18E-32
ESYT1	ENSG00000139641.12	78.476	36.61	1.079	3.41E-19
JDP2	ENSG00000140044.12	7.23	16.925	-1.123	3.71E-09
FBLN5	ENSG00000140092.14	20.644	78.457	-1.876	1.09E-15
SORD	ENSG00000140263.13	8.135	27.22	-1.627	1.19E-09
FGF7	ENSG00000140285.9	0.21	3.333	-1.84	4.31E-25
GCNT3	ENSG00000140297.12	10.39	3.109	1.471	1.07E-06
PCSK6	ENSG00000140479.16	0.52	3.01	-1.399	4.83E-07
RHCG	ENSG00000140519.12	0.11	72.399	-6.047	1.39E-63
NTRK3	ENSG00000140538.16	0.465	2.634	-1.311	1.46E-14
MFGE8	ENSG00000140545.14	11.224	42.127	-1.819	9.90E-12
PMM2	ENSG00000140650.11	25.584	11.304	1.111	9.50E-22
SLC5A2	ENSG00000140675.12	0.26	23.564	-4.285	1.20E-28
ITGAX	ENSG00000140678.16	10.995	1.175	2.463	2.58E-26
TGFB1I1	ENSG00000140682.18	4.075	15.545	-1.705	2.65E-12
IGSF6	ENSG00000140749.8	4.48	0.789	1.615	8.24E-23
NOL3	ENSG00000140939.14	45.991	16.07	1.461	1.26E-21
RPS2	ENSG00000140988.15	1790.441	798.002	1.165	6.26E-20
LRRC46	ENSG00000141294.9	3.53	0.86	1.284	1.68E-17
SLC14A1	ENSG00000141469.16	0.485	5.693	-2.172	9.23E-31
ZMYND15	ENSG00000141497.13	3.815	1.275	1.082	4.86E-12
SLC16A3	ENSG00000141526.14	66.346	22.49	1.52	3.31E-12

NARF	ENSG00000141562.17	61.86	26.728	1.181	2.69E-15
RNF157	ENSG00000141576.14	9.91	3.895	1.156	3.31E-06
MAPK4	ENSG00000141639.11	0.335	3.81	-1.849	1.50E-18
P3H4	ENSG00000141696.12	23.495	8.955	1.299	2.66E-18
STAC2	ENSG00000141750.6	0.03	6.42	-2.849	1.52E-28
PPAP2C	ENSG00000141934.9	21.81	5.031	1.919	2.51E-05
VAV1	ENSG00000141968.7	3.395	0.885	1.221	1.41E-11
TRPM2	ENSG00000142185.16	3.775	0.97	1.277	7.13E-12
RNPEPL1	ENSG00000142327.11	92.539	44.674	1.034	8.14E-19
MYO1F	ENSG00000142347.16	8.2	2.414	1.43	1.26E-13
FBN3	ENSG00000142449.12	0	1.39	-1.257	4.88E-41
TM4SF5	ENSG00000142484.6	2.7	7.975	-1.278	3.05E-03
PRDM16	ENSG00000142611.16	0.05	3.87	-2.214	2.36E-64
SH3BGRL3	ENSG00000142669.13	158.913	77.511	1.026	1.24E-15
FCN3	ENSG00000142748.12	0.83	13.44	-2.98	8.70E-26
TMEM61	ENSG00000143001.4	1.385	12.965	-2.55	5.49E-34
PVRL4	ENSG00000143217.8	0.1	2.365	-1.613	1.64E-17
FCGR2A	ENSG00000143226.13	17.835	8.252	1.026	8.22E-08
RGS5	ENSG00000143248.12	12.605	45.822	-1.783	3.79E-04
NR1I3	ENSG00000143257.11	0.31	3.498	-1.78	7.67E-35
RGS16	ENSG00000143333.6	1.48	4.835	-1.234	1.33E-09
SELENBP1	ENSG00000143416.20	23.714	99.418	-2.023	4.47E-20
SUSD4	ENSG00000143502.14	2.12	10.674	-1.904	1.17E-15
SLC27A3	ENSG00000143554.13	36.126	16.795	1.061	1.60E-06
KCNN3	ENSG00000143603.18	0.14	1.395	-1.071	1.72E-16
PKLR	ENSG00000143627.17	2.015	7.858	-1.555	4.16E-06
ITPKB	ENSG00000143772.9	4.99	13.542	-1.28	1.09E-13
REN	ENSG00000143839.13	0.155	16.223	-3.898	1.20E-31
SOX13	ENSG00000143842.14	6.18	21.67	-1.659	1.03E-16
ETNK2	ENSG00000143845.14	12.5	39.8	-1.596	1.09E-15
MEIS1	ENSG00000143995.19	1.8	5.605	-1.238	1.17E-14
SFXN5	ENSG00000144040.12	6.74	15.237	-1.069	1.31E-13
FBLN7	ENSG00000144152.12	7.37	1.165	1.951	1.97E-14
FAHD2B	ENSG00000144199.11	4.705	10.835	-1.053	2.07E-08
LYG1	ENSG00000144214.9	62.841	12.51	2.24	2.28E-05
NXPH2	ENSG00000144227.4	0	1.1	-1.07	4.08E-33
ZNF385B	ENSG00000144331.18	20.485	6.56	1.507	2.68E-07
HES6	ENSG00000144485.10	7.255	2.91	1.078	8.01E-16
GRIP2	ENSG00000144596.11	0.105	1.22	-1.007	2.02E-26
ITGA9	ENSG00000144668.11	0.835	3.105	-1.162	2.54E-12
NCEH1	ENSG00000144959.9	24.86	9.619	1.284	7.69E-14
TMEM44	ENSG00000145014.17	12.635	5.035	1.176	1.25E-09
OCIAD2	ENSG00000145247.11	410.787	115.052	1.827	5.92E-19
GC	ENSG00000145321.12	0.04	1.129	-1.034	3.45E-10

UGT3A1	ENSG00000145626.11	1.055	17.114	-3.14	1.08E-09
PLK2	ENSG00000145632.14	23.52	10.644	1.074	2.16E-07
GZMA	ENSG00000145649.7	4.015	1.375	1.078	1.01E-06
LHFPL2	ENSG00000145685.13	5.08	1.875	1.081	1.40E-14
TNFAIP8	ENSG00000145779.7	26.27	7.929	1.611	9.12E-20
TIMD4	ENSG00000145850.8	4.765	1.334	1.304	1.77E-10
KLHL3	ENSG00000146021.14	0.625	4.11	-1.653	5.18E-26
PLA2G7	ENSG00000146070.16	3.375	0.125	1.959	4.22E-19
SCUBE3	ENSG00000146197.8	0.07	1.64	-1.303	3.42E-37
PRSS35	ENSG00000146250.6	0.07	1.149	-1.006	1.46E-15
FAXC	ENSG00000146267.11	5.475	1.865	1.176	8.16E-07
RSPO3	ENSG00000146374.13	0.11	1.42	-1.124	5.30E-18
SLC2A12	ENSG00000146411.5	0.14	2.275	-1.522	2.04E-36
SDK1	ENSG00000146555.18	7.02	2.575	1.166	1.28E-10
SSC4D	ENSG00000146700.8	1.515	4.365	-1.093	1.52E-07
TRIM50	ENSG00000146755.10	0.715	2.574	-1.059	8.81E-06
TMEM140	ENSG00000146859.6	69.746	22.975	1.561	2.59E-29
SLC16A2	ENSG00000147100.9	2.715	9.024	-1.432	1.08E-13
IL2RG	ENSG00000147168.12	8.585	3.585	1.064	1.23E-04
RIPPLY1	ENSG00000147223.5	0.185	1.615	-1.142	5.18E-04
GPC3	ENSG00000147257.13	2.015	43.815	-3.894	1.12E-29
TACC1	ENSG00000147526.19	12.35	31.14	-1.268	1.05E-08
LACTB2	ENSG00000147592.8	74.143	29.244	1.313	6.50E-13
SLC26A7	ENSG00000147606.8	0.13	13.535	-3.685	1.73E-72
NTRK2	ENSG00000148053.15	0.99	11.215	-2.618	1.00E-11
IDNK	ENSG00000148057.15	7.565	17.945	-1.145	1.04E-13
RP11-35N6.1	ENSG00000148123.14	0.435	9.479	-2.868	3.40E-28
UGCG	ENSG00000148154.9	42.01	16.492	1.298	1.32E-17
SLC25A25	ENSG00000148339.12	3.905	19.045	-2.031	2.22E-23
LCN2	ENSG00000148346.11	143.071	3.472	5.01	3.33E-16
RGS10	ENSG00000148908.14	24.12	9.42	1.27	8.08E-10
SLC5A12	ENSG00000148942.14	0.26	29.095	-4.578	3.45E-23
PAMR1	ENSG00000149090.11	0.64	2.429	-1.064	5.46E-10
SLC43A1	ENSG00000149150.8	0.62	7.48	-2.388	3.14E-34
SERPINH1	ENSG00000149257.13	96.018	42.395	1.161	2.23E-19
SLC22A8	ENSG00000149452.15	0.08	76.493	-6.165	1.70E-34
PLCH2	ENSG00000149527.17	1.465	7.55	-1.795	1.01E-13
FEZ1	ENSG00000149557.12	2.21	5.765	-1.075	7.65E-07
ESAM	ENSG00000149564.11	13.625	41.518	-1.54	1.11E-13
TAGLN	ENSG00000149591.16	42.171	277.452	-2.689	1.47E-14
FERMT3	ENSG00000149781.12	12.12	4.715	1.199	5.13E-09
MPP7	ENSG00000150054.18	2.02	5.98	-1.209	1.67E-11
FXYD4	ENSG00000150201.14	0.03	175.21	-7.419	3.71E-76
FCGR1A	ENSG00000150337.13	11.505	2.29	1.926	2.93E-17

LYPD6B	ENSG00000150556.16	0.23	5.969	-2.502	2.15E-25
GPM6A	ENSG00000150625.16	0.425	3.163	-1.547	9.23E-29
PRSS23	ENSG00000150687.11	9.87	29.229	-1.476	1.44E-05
IL18	ENSG00000150782.11	33.926	15.609	1.072	9.66E-09
FOXO1	ENSG00000150907.6	3.56	8.364	-1.038	1.87E-08
ITPR1	ENSG00000150995.17	3.925	15.71	-1.763	5.15E-15
THRB	ENSG00000151090.17	1.84	6.29	-1.36	8.94E-13
THRSP	ENSG00000151365.2	0.07	1.77	-1.372	1.34E-11
PTPRO	ENSG00000151490.13	4.1	16.965	-1.817	2.60E-05
NR3C2	ENSG00000151623.14	1.89	8.845	-1.768	9.82E-17
KCNJ1	ENSG00000151704.15	0.09	120.245	-6.797	1.96E-65
TMEM45B	ENSG00000151715.7	1.04	14.484	-2.924	1.12E-24
ZNF385D	ENSG00000151789.9	0.48	2.155	-1.092	4.08E-08
GABRA2	ENSG00000151834.15	0	2.69	-1.884	5.59E-39
SACS	ENSG00000151835.13	3.285	0.93	1.151	2.38E-18
TMEM163	ENSG00000152128.13	11.475	1.03	2.62	8.12E-15
HSPB8	ENSG00000152137.6	119.059	32.316	1.849	2.88E-32
TMEM178A	ENSG00000152154.10	1.39	20.014	-3.136	5.26E-63
SETBP1	ENSG00000152217.16	2.22	5.7	-1.057	1.45E-07
TCF7L1	ENSG00000152284.4	1.85	8.999	-1.811	4.82E-27
KCNK13	ENSG00000152315.4	0.6	3.57	-1.514	7.37E-16
ZSCAN1	ENSG00000152467.9	0.08	1.175	-1.01	2.77E-11
PLEKHH2	ENSG00000152527.13	15.41	4.72	1.521	3.33E-03
SPARCL1	ENSG00000152583.12	11.025	42.271	-1.847	5.68E-12
PANK1	ENSG00000152782.16	4.69	14.535	-1.449	5.24E-08
PLOD2	ENSG00000152952.11	39.726	15.234	1.327	3.84E-14
LGI2	ENSG00000153012.11	0.15	1.545	-1.146	2.63E-26
NR4A2	ENSG00000153234.13	1.35	8.048	-1.945	4.49E-16
PLA2R1	ENSG00000153246.11	2.35	16.442	-2.38	7.27E-22
FRMD1	ENSG00000153303.16	0.14	3.393	-1.946	1.99E-26
PID1	ENSG00000153823.18	0.5	2.87	-1.367	2.17E-21
KCTD15	ENSG00000153885.14	1.67	7.629	-1.692	3.83E-12
LGI4	ENSG00000153902.13	0.34	2.174	-1.244	2.98E-16
GRAP	ENSG00000154016.13	1.225	3.829	-1.118	9.86E-14
SLC5A10	ENSG00000154025.15	5.34	17.134	-1.516	6.06E-04
THY1	ENSG00000154096.13	11.324	147.974	-3.595	8.55E-18
ROBO4	ENSG00000154133.14	2.03	16.967	-2.568	1.53E-35
NRGN	ENSG00000154146.12	2.985	11.921	-1.697	4.38E-27
FAM134B	ENSG00000154153.13	58.25	25.94	1.137	2.94E-13
TNIK	ENSG00000154310.16	11.695	3.435	1.517	3.39E-20
FAM167A	ENSG00000154319.14	1.59	6.59	-1.551	1.12E-07
PGM5	ENSG00000154330.12	3.23	11.82	-1.6	2.12E-09
TMSB4Y	ENSG00000154620.5	0.08	1.58	-1.256	4.56E-18
PDE1C	ENSG00000154678.16	0.12	2.53	-1.656	3.70E-22

JAM2	ENSG00000154721.14	1.28	4.685	-1.318	1.10E-25
SLFN13	ENSG00000154760.13	13.27	2.027	2.237	1.61E-15
FGD5	ENSG00000154783.10	3.42	11.525	-1.503	2.36E-08
RAB6B	ENSG00000154917.10	14.39	3.465	1.785	8.67E-24
PROM2	ENSG00000155066.15	0.37	42.191	-4.978	1.43E-44
SLC7A7	ENSG00000155465.18	13.325	54.385	-1.951	2.22E-03
PIK3AP1	ENSG00000155629.14	18.475	5.35	1.617	7.63E-19
VSIG4	ENSG00000155659.14	11.375	5.03	1.037	4.63E-06
FZD7	ENSG00000155760.2	1.6	5.834	-1.394	2.53E-11
FMN2	ENSG00000155816.19	0.03	1.885	-1.486	4.38E-37
MCU	ENSG00000156026.14	33.69	10.78	1.558	7.49E-26
GNA14	ENSG00000156049.6	0.415	4.569	-1.977	6.25E-52
KCNMA1	ENSG00000156113.20	6.455	2.58	1.058	1.65E-06
TSPAN7	ENSG00000156298.12	2.73	42.545	-3.545	3.42E-37
SFXN2	ENSG00000156398.12	6.17	20.649	-1.594	2.05E-16
PCDH1	ENSG00000156453.13	2.185	24.902	-3.024	8.63E-19
SH3RF2	ENSG00000156463.17	3.295	0.98	1.117	2.13E-16
PPP2R2B	ENSG00000156475.18	0.265	3.16	-1.717	3.33E-21
TMEM171	ENSG00000157111.12	22.554	8.805	1.264	2.59E-03
MMP14	ENSG00000157227.12	86.659	26.954	1.649	3.63E-19
FZD1	ENSG00000157240.3	33.835	7.909	1.967	2.55E-19
SUSD3	ENSG00000157303.10	5.895	14.086	-1.13	2.29E-05
KIT	ENSG00000157404.15	1.1	7.485	-2.015	9.05E-14
HYDIN	ENSG00000157423.17	5.01	1.9	1.051	1.30E-11
ERG	ENSG00000157554.18	0.895	7.54	-2.172	6.79E-34
TSPAN18	ENSG00000157570.11	22.81	10.564	1.042	3.44E-06
SVOPL	ENSG00000157703.15	0.23	1.725	-1.148	2.35E-22
SLC34A2	ENSG00000157765.11	108.902	6.64	3.847	4.30E-07
FMNL2	ENSG00000157827.19	24.055	11.005	1.061	3.34E-14
SLC30A2	ENSG00000158014.14	1.335	11.855	-2.461	1.49E-07
GALNT14	ENSG00000158089.14	131.338	58.724	1.148	1.46E-10
HPD	ENSG00000158104.11	1.44	108.907	-5.493	7.11E-28
FAM46B	ENSG00000158246.7	0.74	3.995	-1.521	6.37E-13
SLC13A3	ENSG00000158296.13	1.445	63.465	-4.721	2.44E-18
NCF1	ENSG00000158517.13	7.335	1.605	1.678	1.46E-13
SLAMF8	ENSG00000158714.10	4.015	1.47	1.022	9.03E-10
NBL1	ENSG00000158747.13	212.894	42.366	2.302	5.56E-12
SLC5A11	ENSG00000158865.12	0.13	1.665	-1.238	4.13E-11
FCER1G	ENSG00000158869.10	69.293	15.885	2.058	1.08E-17
WNT9B	ENSG00000158955.10	0.02	1.557	-1.326	1.82E-50
IFNAR2	ENSG00000159110.19	18.66	7.435	1.221	2.65E-16
LAD1	ENSG00000159166.13	5.25	38.344	-2.654	3.79E-11
STC1	ENSG00000159167.11	1.735	16.554	-2.682	7.76E-17
TNNI1	ENSG00000159173.18	0.16	5.429	-2.471	1.53E-43

RCAN1	ENSG00000159200.17	26.435	68.164	-1.334	1.06E-08
SIM2	ENSG00000159263.15	0.46	8.017	-2.627	2.55E-38
SCUBE1	ENSG00000159307.18	0.09	1.925	-1.424	2.96E-44
IRX6	ENSG00000159387.7	2.49	0.72	1.021	9.36E-03
HK2	ENSG00000159399.9	9.53	0.795	2.552	4.46E-37
SPON2	ENSG00000159674.11	173.212	14.424	3.498	3.46E-18
TPPP3	ENSG00000159713.10	8.675	24.649	-1.407	1.95E-05
RGS12	ENSG00000159788.18	46.775	20.247	1.169	5.76E-11
ZYX	ENSG00000159840.15	154.332	67.834	1.174	5.40E-17
PTGIR	ENSG00000160013.8	0.735	3.889	-1.495	1.51E-20
TFF3	ENSG00000160180.15	0.69	3.895	-1.534	4.94E-14
TMPRSS3	ENSG00000160183.13	0.36	3.115	-1.597	7.51E-12
CBS	ENSG00000160200.17	9.215	3.695	1.122	1.18E-04
TRAPPC10	ENSG00000160218.12	6.885	15.01	-1.022	1.47E-05
ITGB2	ENSG00000160255.16	40.453	8.869	2.07	3.53E-20
FTCD	ENSG00000160282.13	19.349	64.536	-1.687	2.93E-04
ZNF208	ENSG00000160321.14	0.285	1.67	-1.055	4.12E-05
PTH1R	ENSG00000160801.13	66.942	271.893	-2.006	5.18E-08
PTGER1	ENSG00000160951.3	0.13	8.644	-3.093	6.11E-89
MGAT4B	ENSG00000161013.16	243.081	116.747	1.052	4.39E-20
NPHS1	ENSG00000161270.19	0.02	21.59	-4.469	1.10E-50
FDXR	ENSG00000161513.11	34.181	8.31	1.918	3.36E-37
ZNF577	ENSG00000161551.12	4.68	10.889	-1.066	7.05E-07
ZNF385A	ENSG00000161642.17	22.83	6.685	1.633	5.02E-26
NAGS	ENSG00000161653.10	1.265	5.545	-1.531	3.67E-20
PLCD3	ENSG00000161714.11	72.088	15.45	2.152	8.57E-19
GRASP	ENSG00000161835.10	3.185	13.11	-1.753	4.18E-24
IP6K3	ENSG00000161896.10	0.11	2.049	-1.458	8.27E-05
TNFSF13	ENSG00000161955.16	126.574	54.875	1.191	2.07E-08
PRR35	ENSG00000161992.5	0	7.452	-3.079	2.08E-78
WDR90	ENSG00000161996.17	25.905	11.526	1.103	9.30E-05
PAQR4	ENSG00000162073.13	6.735	1.444	1.662	2.68E-20
TAL1	ENSG00000162367.11	0.34	1.985	-1.155	3.01E-29
FAM151A	ENSG00000162391.11	0.205	38.897	-5.049	6.21E-30
PPAP2B	ENSG00000162407.8	27.295	62.085	-1.157	9.75E-05
SLC45A1	ENSG00000162426.14	0.665	3.765	-1.517	1.06E-36
SLC25A34	ENSG00000162461.7	0.93	3.465	-1.21	6.28E-17
LAPTM5	ENSG00000162511.7	79.605	15.747	2.267	9.56E-22
NTNG1	ENSG00000162631.18	0.23	4.625	-2.193	9.50E-25
GBP2	ENSG00000162645.12	42.555	13.862	1.551	5.23E-15
GBP4	ENSG00000162654.8	2.965	8.733	-1.296	1.55E-06
VCAM1	ENSG00000162692.10	140.209	41.7	1.726	1.96E-07
SNED1	ENSG00000162804.13	0.745	3.93	-1.498	3.45E-19
MT2P1	ENSG00000162840.4	0	1.07	-1.05	1.50E-16

PIGR	ENSG00000162896.5	469.945	110.902	2.073	6.53E-06
KCNJ3	ENSG00000162989.4	1.645	4.417	-1.034	3.72E-06
FRZB	ENSG00000162998.4	1.745	8.097	-1.729	2.94E-11
SPATA18	ENSG00000163071.10	11.785	3.225	1.597	2.20E-14
TNFAIP8L2	ENSG00000163154.5	4.32	0.98	1.426	1.54E-15
S100A11	ENSG00000163191.5	1300.634	368.915	1.815	9.68E-28
NIPAL1	ENSG00000163293.11	0.575	2.155	-1.002	1.26E-16
GPR155	ENSG00000163328.13	1.19	5.786	-1.632	1.59E-14
LINC01116	ENSG00000163364.9	2.08	9.995	-1.836	5.48E-09
LMOD1	ENSG00000163431.12	0.66	6.904	-2.251	1.64E-18
FBLN2	ENSG00000163520.13	2.1	7.205	-1.404	1.19E-03
GLB1L	ENSG00000163521.15	28.149	11.875	1.179	3.46E-11
NFASC	ENSG00000163531.15	0.915	19.203	-3.399	1.38E-49
SERPINI1	ENSG00000163536.12	3.89	11.92	-1.402	6.29E-10
NUAK2	ENSG00000163545.8	4.575	25.405	-2.244	3.58E-17
RPL22L1	ENSG00000163584.17	63.619	20.913	1.56	2.71E-20
FABP1	ENSG00000163586.9	0.19	163.364	-7.11	6.46E-46
PPM1K	ENSG00000163644.14	3.47	8.724	-1.121	5.70E-09
IL17RE	ENSG00000163701.18	0.86	5.205	-1.738	3.97E-11
PCOLCE2	ENSG00000163710.7	2.365	21.895	-2.766	2.95E-17
UCN	ENSG00000163794.6	2.075	0.415	1.12	4.70E-14
SLC6A20	ENSG00000163817.15	10.925	1.27	2.393	9.13E-13
LRRC2	ENSG00000163827.12	0.16	2.56	-1.618	8.79E-49
HEYL	ENSG00000163909.7	1.665	5.015	-1.174	2.84E-07
TKT	ENSG00000163931.15	177.416	80.568	1.129	2.61E-18
SPRY1	ENSG00000164056.10	6.325	18.938	-1.445	1.64E-06
HPGD	ENSG00000164120.13	1.81	11.45	-2.148	3.88E-19
FAM198B	ENSG00000164125.15	1.995	5.69	-1.159	3.17E-04
NPY1R	ENSG00000164128.6	3.595	14.664	-1.769	7.19E-11
NPY5R	ENSG00000164129.11	0.42	1.87	-1.015	1.52E-08
ITGA2	ENSG00000164171.10	2.82	6.78	-1.026	3.05E-03
RANBP3L	ENSG00000164188.8	0.08	5.385	-2.564	7.50E-53
SPINK1	ENSG00000164266.10	0.32	23.905	-4.238	1.02E-27
RHOBTB3	ENSG00000164292.12	3.67	15.07	-1.783	1.44E-14
GPX8	ENSG00000164294.13	17.825	5.433	1.549	6.98E-18
TMEM174	ENSG00000164325.7	0.645	12.061	-2.989	2.22E-31
ACSL6	ENSG00000164398.12	0.245	1.71	-1.122	2.89E-15
TMEM200A	ENSG00000164484.11	15.37	3.265	1.94	1.02E-13
RAET1E	ENSG00000164520.11	0.04	1.159	-1.054	8.63E-35
PI16	ENSG00000164530.13	0.04	1.464	-1.245	3.15E-30
PTTG1	ENSG00000164611.12	5.78	1.936	1.207	4.42E-15
FABP5	ENSG00000164687.10	17.334	5.057	1.598	1.28E-08
SHH	ENSG00000164690.7	6.42	2.615	1.037	2.78E-06
PGAM2	ENSG00000164708.5	0.39	5.356	-2.193	6.48E-35

SOX17	ENSG00000164736.5	0.46	2.16	-1.114	4.71E-25
KCNV1	ENSG00000164794.8	1.285	0.055	1.115	7.74E-17
GPR146	ENSG00000164849.8	2.91	6.92	-1.018	3.11E-08
NOS3	ENSG00000164867.10	1.635	9.18	-1.95	6.93E-30
TP53INP1	ENSG00000164938.13	13.18	5.745	1.072	2.43E-12
FREM1	ENSG00000164946.19	2	10.19	-1.899	2.32E-19
SVEP1	ENSG00000165124.17	0.275	2.309	-1.376	1.19E-21
TRPV6	ENSG00000165125.17	0.12	5.99	-2.642	4.57E-48
FBP1	ENSG00000165140.9	33.375	89.424	-1.395	2.70E-11
TMEM246	ENSG00000165152.8	12.32	4.46	1.287	6.03E-11
WBSCR27	ENSG00000165171.10	31.179	6.585	2.085	2.59E-15
NCF1C	ENSG00000165178.9	3.53	0.75	1.372	2.20E-11
NLGN4Y	ENSG00000165246.12	0.1	1.265	-1.042	1.42E-06
PHYHIPL	ENSG00000165443.11	30.41	11.783	1.297	1.60E-05
PKNOX2	ENSG00000165495.15	0.14	1.344	-1.04	2.67E-19
OXGR1	ENSG00000165621.8	0.01	2.055	-1.597	5.82E-59
ZNF503	ENSG00000165655.15	7.655	19.72	-1.259	1.36E-11
TMEM52B	ENSG00000165685.8	0.62	69.772	-5.449	5.02E-51
FRMD7	ENSG00000165694.9	0	1.055	-1.039	3.32E-66
FAM69B	ENSG00000165716.9	0.815	8.569	-2.398	2.79E-25
STOX1	ENSG00000165730.14	0.19	1.54	-1.094	2.36E-14
KIAA1462	ENSG00000165757.8	2.145	7.629	-1.456	5.04E-12
NDRG2	ENSG00000165795.20	58.535	136.48	-1.207	4.47E-11
PRAP1	ENSG00000165828.13	19.413	62.996	-1.648	3.28E-03
HSPA12A	ENSG00000165868.12	3.095	14.705	-1.939	8.26E-16
IFI27	ENSG00000165949.12	800.051	131.954	2.591	1.02E-11
NELL1	ENSG00000165973.17	0.02	5.627	-2.7	1.47E-55
LIPC	ENSG00000166035.10	26.023	7.913	1.6	7.30E-08
JAM3	ENSG00000166086.12	3.155	10.079	-1.415	1.26E-08
IKBIP	ENSG00000166130.14	20.005	5.344	1.727	2.59E-29
GABRB3	ENSG00000166206.13	0.215	2.923	-1.691	1.85E-08
WDR72	ENSG00000166415.14	9.85	21.615	-1.06	4.83E-08
PLD4	ENSG00000166428.12	6.375	0.915	1.945	1.42E-20
TMEM130	ENSG00000166448.14	6.505	2.432	1.129	7.94E-03
MFAP4	ENSG00000166482.11	7.41	31.989	-1.972	2.49E-12
NNMT	ENSG00000166741.7	451.082	41.29	3.418	2.51E-10
ZNF667-AS1	ENSG00000166770.10	10.934	22.942	-1.004	6.60E-07
KIAA0101	ENSG00000166803.10	5.575	1.31	1.509	1.95E-17
MESP1	ENSG00000166823.5	7.14	1.835	1.522	1.85E-15
SCNN1G	ENSG00000166828.2	0.01	30.874	-4.98	6.51E-74
RBPMS2	ENSG00000166831.8	4.32	11.55	-1.238	2.11E-08
NAV2	ENSG00000166833.19	3.445	9.336	-1.217	1.21E-06
GLYATL1	ENSG00000166840.13	27.672	61.239	-1.118	7.10E-05
ELFN2	ENSG00000166897.14	3.325	0.335	1.696	2.04E-16

SCG5	ENSG00000166922.8	4.485	0.435	1.934	4.64E-11
FAM102A	ENSG00000167106.11	16.47	37.62	-1.144	1.86E-03
GPRC5B	ENSG00000167191.11	8.93	42.015	-2.115	8.71E-11
SNX20	ENSG00000167208.14	1.785	0.375	1.018	2.28E-11
IGF2	ENSG00000167244.17	0.975	12.931	-2.818	5.05E-22
TUBA1A	ENSG00000167552.13	168.09	60.509	1.459	8.76E-20
LAIR1	ENSG00000167613.15	12.368	2.885	1.783	9.62E-15
PPP1R14A	ENSG00000167641.10	3.375	20.929	-2.326	2.27E-22
PSCA	ENSG00000167653.4	0.135	1.315	-1.028	2.90E-14
NXN	ENSG00000167693.16	16.285	7.41	1.039	2.91E-06
GPT	ENSG00000167701.13	5.36	18.85	-1.642	2.65E-08
GGT6	ENSG00000167741.10	0.345	41.732	-4.99	5.82E-45
KLK1	ENSG00000167748.10	0.18	32.026	-4.807	4.14E-21
KLK6	ENSG00000167755.13	0.06	9.628	-3.326	1.07E-18
KRT80	ENSG00000167767.13	8.26	2.074	1.591	2.50E-16
IGFBP6	ENSG00000167779.7	305.26	34.229	3.12	1.77E-14
TMEM88	ENSG00000167874.6	6.04	25.469	-1.911	2.88E-30
TK1	ENSG00000167900.11	17.145	6.534	1.268	1.04E-10
SOST	ENSG00000167941.2	0	4.343	-2.418	3.48E-48
NAALADL1	ENSG00000168060.14	1.5	4.73	-1.197	2.85E-06
SCARA3	ENSG00000168077.13	23.072	4.59	2.106	6.30E-05
FAM83B	ENSG00000168143.8	0.01	1.16	-1.096	2.70E-22
TTC39C	ENSG00000168234.12	6.43	2.695	1.008	2.01E-12
FOXI1	ENSG00000168269.8	0	23.965	-4.642	6.28E-91
FAM107A	ENSG00000168309.16	2.445	28.909	-3.118	6.32E-30
TAP1	ENSG00000168394.10	40.44	18.065	1.12	1.23E-18
SCNN1B	ENSG00000168447.10	0.06	27.752	-4.762	7.76E-79
RAB31	ENSG00000168461.12	20.69	9.775	1.009	9.64E-09
NPNT	ENSG00000168743.12	15.59	48.666	-1.582	2.10E-06
TSPAN5	ENSG00000168785.7	0.385	4.799	-2.066	2.28E-15
PLA2G4F	ENSG00000168907.13	0.11	12.379	-3.591	3.50E-51
LGALS9	ENSG00000168961.16	40.38	13.445	1.518	1.49E-12
KLK7	ENSG00000169035.11	0	5.539	-2.709	1.10E-44
PARM1	ENSG00000169116.11	3.085	27.133	-2.784	1.34E-21
RGS14	ENSG00000169220.17	56.541	24.21	1.191	1.50E-16
GPRIN1	ENSG00000169258.6	2.385	0.23	1.46	3.98E-22
SHE	ENSG00000169291.9	1.675	5.6	-1.303	5.12E-19
UMOD	ENSG00000169344.15	0.16	1766.344	-10.573	3.19E-108
GP2	ENSG00000169347.16	0	3.81	-2.266	2.32E-78
PTAFR	ENSG00000169403.11	4.515	1.5	1.141	2.35E-08
RNASE6	ENSG00000169413.2	10.28	3.875	1.21	7.20E-09
SLC38A11	ENSG00000169507.9	1.33	4.689	-1.288	3.12E-06
GPR183	ENSG00000169508.6	4.945	1.605	1.19	3.01E-08
MUC15	ENSG00000169550.12	0	7.9	-3.154	5.30E-68

SPNS1	ENSG00000169682.17	51.52	24.925	1.019	1.43E-22
LRRC45	ENSG00000169683.7	15.08	6.675	1.067	5.38E-12
MT1E	ENSG00000169715.14	64.536	269.267	-2.044	2.60E-09
LDB2	ENSG00000169744.12	2.325	17.52	-2.478	3.00E-32
NLGN1	ENSG00000169760.17	3.57	0.715	1.414	1.31E-15
LINGO1	ENSG00000169783.12	0.42	2.205	-1.174	1.77E-11
MUC3A	ENSG00000169894.17	21.97	0.53	3.908	6.12E-20
ITGAM	ENSG00000169896.16	4.38	1.51	1.1	2.12E-11
RNF150	ENSG00000170153.10	0.5	5.627	-2.143	4.19E-37
HOXD4	ENSG00000170166.5	1.415	7.325	-1.785	3.29E-22
SLC16A5	ENSG00000170190.15	2.275	24.853	-2.981	4.67E-28
HSPB2	ENSG00000170276.5	18.775	5.465	1.613	2.39E-13
FABP4	ENSG00000170323.8	0.505	5.86	-2.188	6.22E-23
FOS	ENSG00000170345.9	42.238	162.601	-1.92	2.88E-06
LRRN2	ENSG00000170382.11	0.62	9.925	-2.754	3.25E-21
KRT8	ENSG00000170421.11	784.564	257.066	1.606	8.86E-19
METTL7B	ENSG00000170439.6	49.334	8.1	2.468	5.63E-16
SLC23A1	ENSG00000170482.16	4.245	9.816	-1.044	4.76E-03
NPAS2	ENSG00000170485.16	30.728	14.341	1.048	3.54E-08
PFKFB3	ENSG00000170525.18	18.445	41.217	-1.118	1.69E-04
IRX1	ENSG00000170549.3	0	7.525	-3.092	1.32E-67
IRX2	ENSG00000170561.12	0.09	6.8	-2.839	5.20E-53
KCNS3	ENSG00000170745.11	11.005	3.674	1.361	3.53E-13
HTRA3	ENSG00000170801.9	0.675	2.45	-1.042	6.27E-11
GPR27	ENSG00000170837.2	0.44	2.975	-1.465	4.95E-04
MINA	ENSG00000170854.17	6.575	14.525	-1.035	3.97E-12
TMEM43	ENSG00000170876.7	64.861	25.277	1.326	2.07E-16
OSCAR	ENSG00000170909.13	3.575	0.52	1.59	1.82E-20
PRKCDBP	ENSG00000170955.9	37.339	15.668	1.202	1.21E-04
S1PR1	ENSG00000170989.8	3.125	14.77	-1.935	4.04E-17
HS6ST2	ENSG00000171004.17	0.06	5.061	-2.515	1.51E-34
SOX7	ENSG00000171056.7	0.485	2.775	-1.346	3.67E-26
GIMAP8	ENSG00000171115.3	1.34	6.015	-1.584	1.76E-18
NRTN	ENSG00000171119.2	6.655	2.075	1.316	1.52E-11
PRKCE	ENSG00000171132.13	1.86	5.46	-1.175	1.28E-15
TMEM37	ENSG00000171227.6	30.209	82.395	-1.418	4.53E-07
KRT13	ENSG00000171401.14	0.05	2.115	-1.569	3.25E-07
PDE7B	ENSG00000171408.13	1.395	4.463	-1.19	1.49E-12
GLOD5	ENSG00000171433.11	1.96	5.08	-1.038	1.08E-06
MCC	ENSG00000171444.17	1.31	3.649	-1.009	4.87E-07
HOPX	ENSG00000171476.21	2.76	8.617	-1.355	6.75E-06
PTGER4	ENSG00000171522.5	1.275	5.975	-1.616	3.46E-20
MAP6	ENSG00000171533.11	1.075	10.069	-2.415	1.29E-22
SLC25A33	ENSG00000171612.6	1.75	4.55	-1.013	3.71E-22

RGS19	ENSG00000171700.13	9.48	4.08	1.045	1.22E-15
VAT1L	ENSG00000171724.2	0.03	1.205	-1.098	1.47E-35
PAH	ENSG00000171759.8	0.475	65.89	-5.503	4.92E-15
TLN2	ENSG00000171914.14	11.935	28.424	-1.186	1.10E-06
SYNPO	ENSG00000171992.12	8.905	36.145	-1.907	2.95E-09
ISG20	ENSG00000172183.14	13.28	4.519	1.371	9.54E-12
TPSAB1	ENSG00000172236.16	21.935	2.744	2.615	1.33E-06
NEGR1	ENSG00000172260.13	0.12	2.128	-1.482	3.98E-36
SPTLC3	ENSG00000172296.12	16.42	7.554	1.026	3.45E-08
RCAN2	ENSG00000172348.14	6.725	20.795	-1.496	1.30E-06
SYNPO2	ENSG00000172403.10	0.82	5.54	-1.845	1.03E-13
TTC36	ENSG00000172425.10	0.27	2.429	-1.433	9.98E-34
PDE3A	ENSG00000172572.6	0.355	2.355	-1.308	9.47E-15
LRRC20	ENSG00000172731.13	11.505	2.725	1.747	2.62E-38
RPL38	ENSG00000172809.12	1660.669	790.597	1.07	3.49E-18
OVOL1	ENSG00000172818.9	1.485	4.97	-1.265	2.99E-10
SSH3	ENSG00000172830.12	34.226	15.134	1.127	9.27E-10
MRGPRF	ENSG00000172935.8	0.37	8.935	-2.858	1.71E-50
SLC22A13	ENSG00000172940.11	0.28	1.651	-1.051	2.72E-09
MIR4435-2HG	ENSG00000172965.14	42.774	17.534	1.24	8.18E-18
SYT12	ENSG00000173227.13	2.3	0.205	1.453	9.12E-12
MMRN2	ENSG00000173269.13	4.11	17.479	-1.854	1.49E-19
MZT2A	ENSG00000173272.13	94.006	43.534	1.093	2.31E-15
NDNF	ENSG00000173376.13	0.05	6.377	-2.813	6.13E-42
OLR1	ENSG00000173391.8	7.84	2.859	1.196	5.49E-08
PPP1R14B	ENSG00000173457.10	126.745	55.473	1.178	6.84E-18
TNFRSF10C	ENSG00000173535.13	14.835	3.023	1.977	2.37E-19
UGT2A1	ENSG00000173610.11	0.05	1.378	-1.18	3.53E-15
HSPB7	ENSG00000173641.17	0.34	9.185	-2.926	1.32E-48
HEG1	ENSG00000173706.12	4.245	10.669	-1.154	4.12E-04
RNF213	ENSG00000173821.19	84.779	35.351	1.239	9.45E-10
SPTBN2	ENSG00000173898.11	8.845	48.12	-2.319	2.17E-24
SLCO4C1	ENSG00000173930.8	25.98	11.434	1.118	1.18E-05
PIFO	ENSG00000173947.13	16.69	6.27	1.283	5.12E-13
RP11-23P13.6	ENSG00000174171.5	8.485	2.334	1.509	2.98E-04
SELP	ENSG00000174175.16	1.265	6.942	-1.81	1.76E-26
PRPF8	ENSG00000174231.16	137.729	65.919	1.052	6.69E-12
PHLDA3	ENSG00000174307.6	42.645	11.536	1.8	1.48E-28
SLC16A11	ENSG00000174326.11	0.46	3.234	-1.536	1.34E-18
SLC6A19	ENSG00000174358.15	0.849	17.969	-3.359	2.21E-12
SLC26A9	ENSG00000174502.18	0.47	2.795	-1.368	1.27E-07
MFSD4	ENSG00000174514.12	6.755	40.388	-2.416	1.32E-23
GOLT1A	ENSG00000174567.7	1.975	8.974	-1.745	5.66E-08
UGT8	ENSG00000174607.10	6.145	14.628	-1.129	1.69E-05

SLCO2A1	ENSG00000174640.12	10.54	28.497	-1.354	2.58E-05
SH3PXD2B	ENSG00000174705.11	4.215	1.53	1.044	1.02E-14
PCP2	ENSG00000174788.9	1.015	3.37	-1.117	4.84E-12
RIN1	ENSG00000174791.10	6.905	1.725	1.537	6.38E-12
SEZ6L2	ENSG00000174938.14	61.01	3.803	3.69	1.20E-33
FUT1	ENSG00000174951.10	0.21	1.445	-1.015	1.47E-22
UBE2C	ENSG00000175063.16	3.795	0.7	1.496	5.66E-15
TP53I11	ENSG00000175274.18	6.095	19.94	-1.561	2.91E-11
LPL	ENSG00000175445.14	1.98	8.643	-1.694	1.50E-15
NR2F1	ENSG00000175745.11	2.24	23.235	-2.903	1.14E-28
SFN	ENSG00000175793.11	15.624	2.835	2.116	3.32E-06
MSRA	ENSG00000175806.14	17.965	43.036	-1.215	2.36E-08
GAPT	ENSG00000175857.8	2.265	0.355	1.269	1.25E-15
HOXD8	ENSG00000175879.8	11.02	55.285	-2.227	1.09E-19
ORAI3	ENSG00000175938.6	37.335	15.864	1.185	1.99E-24
NUPR1	ENSG00000176046.8	141.832	59.071	1.25	4.51E-05
GPX2	ENSG00000176153.11	0.24	2.41	-1.459	1.34E-03
SPHK1	ENSG00000176170.13	11.235	2.221	1.926	2.37E-11
HSD11B2	ENSG00000176387.6	2.65	369.671	-6.666	9.54E-48
RIMS2	ENSG00000176406.20	1.975	0.16	1.359	1.70E-11
PLA2G16	ENSG00000176485.10	236.705	67.749	1.79	1.30E-15
PRR15	ENSG00000176532.3	5.015	14.813	-1.394	5.96E-12
RNF152	ENSG00000176641.10	1.51	13.573	-2.538	2.73E-23
FOXC2	ENSG00000176692.5	0.295	2.449	-1.413	6.11E-24
TTTY14	ENSG00000176728.7	0.155	5.606	-2.516	3.16E-18
IRX5	ENSG00000176842.14	9.96	2.079	1.832	1.43E-04
TYMS	ENSG00000176890.15	30.6	7.724	1.857	6.40E-24
MUC20	ENSG00000176945.16	5.59	57.318	-3.146	7.53E-33
LINC00982	ENSG00000177133.10	0.07	56.767	-5.755	3.16E-94
NUDT4P1	ENSG00000177144.6	4.635	13.356	-1.349	5.05E-04
RIMKLA	ENSG00000177181.14	2.46	0.53	1.177	2.00E-24
HIC1	ENSG00000177374.12	1.025	3.76	-1.233	3.75E-12
TGIF1	ENSG00000177426.20	99.019	43.314	1.174	4.27E-12
GPR4	ENSG00000177464.4	0.76	5.23	-1.824	7.79E-24
IRX3	ENSG00000177508.11	57.911	8.93	2.569	1.60E-06
FLII	ENSG00000177731.15	230.423	92.937	1.301	6.26E-15
MYOZ1	ENSG00000177791.11	0.32	2.12	-1.241	1.34E-28
KCNJ10	ENSG00000177807.6	0.1	13.4	-3.711	3.86E-68
ODF3B	ENSG00000177989.13	35.072	8.18	1.974	1.96E-13
RNF212	ENSG00000178222.12	1.73	8.32	-1.771	5.19E-12
GALNT11	ENSG00000178234.12	175.271	63.913	1.441	4.35E-09
SHISA3	ENSG00000178343.4	1.635	21.115	-3.069	4.82E-17
TUBAL3	ENSG00000178462.11	0.05	1.719	-1.373	7.58E-17
ERBB4	ENSG00000178568.13	0.02	3.463	-2.129	2.16E-38

FAM132B	ENSG00000178752.15	13.105	1.244	2.652	4.03E-09
RNF186	ENSG00000178828.6	0.55	11.713	-3.036	9.60E-13
MSC	ENSG00000178860.8	9.995	2.317	1.729	6.65E-12
EXOC3L1	ENSG00000179044.15	0.97	4.35	-1.441	1.54E-30
GIMAP7	ENSG00000179144.4	4.535	13.115	-1.351	5.45E-08
WSCD1	ENSG00000179314.13	9.81	3.625	1.225	2.09E-06
HLA-DQB1	ENSG00000179344.16	67.069	31.74	1.056	1.43E-04
GATA2	ENSG00000179348.11	1.175	14.715	-2.853	7.83E-37
GPC5	ENSG00000179399.13	0.02	1.334	-1.194	2.79E-39
LBX2	ENSG00000179528.15	4.264	0.77	1.573	5.01E-20
GRM8	ENSG00000179603.17	3.43	0.715	1.369	1.33E-08
PIPOX	ENSG00000179761.11	1.73	20.164	-2.955	6.92E-19
GIPC3	ENSG00000179855.5	0.39	2.075	-1.146	6.56E-25
ITLN1	ENSG00000179914.4	0.17	1.894	-1.306	1.04E-21
LYNX1	ENSG00000180155.18	3.42	10.475	-1.376	1.22E-07
RCC1	ENSG00000180198.15	12.89	5.515	1.092	1.00E-14
SLC9A4	ENSG00000180251.4	0.01	11.698	-3.652	1.68E-57
MTURN	ENSG00000180354.15	9.115	35.125	-1.836	6.60E-20
GAS1	ENSG00000180447.6	0.88	6.03	-1.903	1.54E-27
HMHA1	ENSG00000180448.10	32.504	12.139	1.35	2.25E-14
SSTR2	ENSG00000180616.8	0.12	2.239	-1.532	1.53E-34
SLC25A42	ENSG00000181035.13	6.94	16.837	-1.168	1.68E-12
F2R	ENSG00000181104.6	4.05	12.224	-1.389	1.41E-06
HIST3H2A	ENSG00000181218.5	8.675	1.435	1.99	6.82E-20
ZNF467	ENSG00000181444.12	4.23	11.339	-1.238	1.75E-08
RAP2B	ENSG00000181467.3	10.605	3.345	1.417	7.04E-24
SLX1B	ENSG00000181625.17	16.854	7.38	1.091	2.53E-08
TMEM252	ENSG00000181778.4	36.663	13.714	1.356	3.00E-03
IDH2	ENSG00000182054.9	89.421	213.035	-1.243	3.37E-07
TMEM30B	ENSG00000182107.6	0.38	9.345	-2.906	3.86E-25
FAM89A	ENSG00000182118.5	3.285	9.58	-1.304	1.10E-16
KCNIP1	ENSG00000182132.12	0.27	1.879	-1.181	2.96E-07
RGMA	ENSG00000182175.13	0.325	1.705	-1.03	9.03E-16
FAM153B	ENSG00000182230.11	0.47	2.635	-1.306	1.35E-11
GLTPD2	ENSG00000182327.7	0.8	5.456	-1.843	1.95E-12
SPNS3	ENSG00000182557.7	6.7	2.53	1.125	4.23E-06
EPHB3	ENSG00000182580.2	0.28	2.465	-1.437	1.01E-22
PLCB1	ENSG00000182621.16	1.79	5.095	-1.127	4.65E-06
PLCXD3	ENSG00000182836.9	0.09	1.885	-1.404	3.43E-30
GALNT9	ENSG00000182870.12	1.82	5.757	-1.261	8.48E-04
TCEAL7	ENSG00000182916.7	0.475	2.61	-1.291	8.86E-11
PYCR1	ENSG00000183010.16	7.29	2.219	1.365	1.95E-10
SPNS2	ENSG00000183018.8	30.615	68.8	-1.143	6.44E-05
SLC8A1	ENSG00000183023.18	0.83	4.78	-1.659	5.53E-17

PCP4	ENSG00000183036.10	8.819	21.168	-1.175	7.36E-04
GAS6	ENSG00000183087.14	34.018	79.704	-1.205	8.93E-08
GPC6	ENSG00000183098.10	6.96	2.695	1.107	2.63E-05
WT1-AS	ENSG00000183242.11	0.01	1.43	-1.266	3.20E-28
OVCH2	ENSG00000183378.11	0.01	2.524	-1.803	7.10E-30
FAM46C	ENSG00000183508.4	1.77	4.7	-1.041	1.23E-05
FAM167B	ENSG00000183615.5	6.255	21.19	-1.613	3.60E-18
FAM101B	ENSG00000183688.4	1.49	5.41	-1.364	1.44E-10
LHFP	ENSG00000183722.7	6.035	19.275	-1.527	6.27E-10
MACC1	ENSG00000183742.12	14.775	4.7	1.469	4.00E-08
OLFML1	ENSG00000183801.7	0.795	3.265	-1.249	5.25E-08
FAM162B	ENSG00000183807.7	0.15	1.615	-1.185	6.46E-38
FAM3B	ENSG00000183844.16	0.325	18.267	-3.862	4.90E-41
UTY	ENSG00000183878.15	0.17	2.995	-1.772	7.36E-16
SMTN	ENSG00000183963.18	10.72	24.789	-1.138	2.56E-13
ST6GALNAC3	ENSG00000184005.10	0.265	3.04	-1.675	4.73E-47
TMPRSS2	ENSG00000184012.11	0.995	36.331	-4.226	1.84E-49
NR2C2AP	ENSG00000184162.14	20.36	9.499	1.025	1.09E-18
OLIG1	ENSG00000184221.12	0.115	2.9	-1.806	7.10E-30
PCDH9	ENSG00000184226.14	0.04	1.1	-1.014	3.61E-49
SLIT3	ENSG00000184347.14	1.69	13.704	-2.45	7.06E-17
TMEM255B	ENSG00000184497.12	1.97	6.801	-1.393	1.04E-18
LPAR5	ENSG00000184574.9	2.44	0.45	1.246	1.96E-17
TMEM173	ENSG00000184584.12	9.685	22.399	-1.131	6.80E-05
RALYL	ENSG00000184672.11	0	3.84	-2.275	3.32E-73
TCEAL2	ENSG00000184905.8	0.16	10.92	-3.361	3.25E-36
FMNL1	ENSG00000184922.13	10.105	4.205	1.093	5.76E-10
LCN12	ENSG00000184925.11	4.76	14.624	-1.44	7.45E-18
WT1	ENSG00000184937.12	0.07	8.738	-3.186	1.49E-39
SORCS2	ENSG00000184985.16	0.205	6.229	-2.585	2.47E-22
FLRT2	ENSG00000185070.10	0.18	1.62	-1.151	3.51E-27
FAM43A	ENSG00000185112.5	1.755	6.07	-1.36	6.65E-13
INPP5J	ENSG00000185133.13	1.96	20.619	-2.869	2.04E-46
MFSD6L	ENSG00000185156.5	0.06	1.865	-1.434	4.74E-39
TNFAIP2	ENSG00000185215.8	130.038	43.954	1.543	2.94E-10
WBSCR17	ENSG00000185274.11	0.04	2.695	-1.829	1.28E-23
NUPR1L	ENSG00000185290.3	0	9.505	-3.393	3.75E-57
SOCS1	ENSG00000185338.4	2.295	7.405	-1.351	1.45E-08
PARK2	ENSG00000185345.18	2.145	7.32	-1.403	5.87E-07
STAC3	ENSG00000185482.7	4.215	0.915	1.445	1.03E-27
MUC1	ENSG00000185499.16	93.347	276.272	-1.555	3.75E-07
P4HB	ENSG00000185624.14	609.675	282.684	1.106	3.27E-26
PBX1	ENSG00000185630.18	8.24	17.744	-1.02	5.76E-04
NTF3	ENSG00000185652.11	0.23	1.835	-1.205	2.98E-34

MORN5	ENSG00000185681.12	4.525	1.215	1.319	3.48E-08
PRAME	ENSG00000185686.17	7.275	0.74	2.25	1.55E-11
NAT8L	ENSG00000185818.7	0.21	7.114	-2.745	1.36E-38
EVI2B	ENSG00000185862.6	6.395	2.145	1.233	9.25E-11
IFITM1	ENSG00000185885.15	31.554	103.063	-1.677	4.47E-06
LINC00839	ENSG00000185904.11	0.18	6.355	-2.64	6.37E-23
LRRC70	ENSG00000186105.7	0.43	2.185	-1.155	3.30E-35
MST1P2	ENSG00000186301.8	1.96	6.155	-1.273	1.81E-07
SLC36A2	ENSG00000186335.8	0.02	10.047	-3.437	1.18E-39
THBS2	ENSG00000186340.14	15.825	3.829	1.801	6.23E-11
RXRA	ENSG00000186350.9	8.33	22.19	-1.314	9.21E-09
NAP1L2	ENSG00000186462.8	1.545	4.889	-1.21	6.33E-09
MIR22HG	ENSG00000186594.12	8.95	27.27	-1.507	3.00E-14
PDE2A	ENSG00000186642.15	1.07	13.859	-2.844	6.37E-61
MST1L	ENSG00000186715.10	3.14	39.05	-3.274	2.15E-21
FOXI2	ENSG00000186766.7	0.01	1.249	-1.155	7.43E-29
LILRB4	ENSG00000186818.12	11.21	1.3	2.409	3.59E-26
TRABD2A	ENSG00000186854.10	3.185	0.665	1.33	1.16E-10
QRFPR	ENSG00000186867.10	4.19	0.845	1.492	9.24E-08
KANK3	ENSG00000186994.11	3.615	9.009	-1.117	9.27E-16
ESPN	ENSG00000187017.14	6.99	35.551	-2.194	3.19E-13
MT1X	ENSG00000187193.8	39.495	323.585	-3.003	3.84E-23
GJA4	ENSG00000187513.8	3.015	14.348	-1.935	8.33E-20
PLEKHN1	ENSG00000187583.10	2.895	0.36	1.518	1.78E-09
TCL6	ENSG00000187621.14	0.09	3.709	-2.111	2.42E-29
SAMD11	ENSG00000187634.10	2.03	10.71	-1.95	5.52E-08
PERM1	ENSG00000187642.9	3.08	0.915	1.091	7.68E-06
SPRY4	ENSG00000187678.8	3.835	9.232	-1.082	4.35E-05
FANCA	ENSG00000187741.14	7.915	1.78	1.681	1.41E-19
TMEM72	ENSG00000187783.11	0.81	48.544	-4.775	1.00E-40
PEAR1	ENSG00000187800.13	0.81	4.795	-1.679	1.04E-28
PALM3	ENSG00000187867.8	6.175	16.904	-1.319	2.37E-14
RAB42	ENSG00000188060.6	4.225	0.535	1.767	1.34E-18
MESP2	ENSG00000188095.4	1.62	0.205	1.121	2.07E-11
MAPK12	ENSG00000188130.13	3.095	8.395	-1.198	2.53E-03
HEPACAM2	ENSG00000188175.9	0.02	7.725	-3.097	2.60E-72
PP7080	ENSG00000188242.4	18.91	137.324	-2.796	7.59E-29
SBK1	ENSG00000188322.4	3.975	1.389	1.058	2.06E-06
SLC38A3	ENSG00000188338.14	0.09	1.857	-1.39	1.10E-13
ZP3	ENSG00000188372.14	10.855	2.185	1.896	1.58E-10
SERPINA5	ENSG00000188488.13	2.535	111.784	-4.996	3.15E-39
NCCRP1	ENSG00000188505.4	0.065	1.305	-1.114	7.36E-20
FAM83G	ENSG00000188522.14	0.6	3.66	-1.542	7.44E-05
HBA2	ENSG00000188536.12	32.716	179.14	-2.418	6.30E-08

PRELP	ENSG00000188783.5	1.35	9.201	-2.118	5.58E-10
FAM26F	ENSG00000188820.12	6.05	2.24	1.122	2.20E-08
LRRK2	ENSG00000188906.13	41.475	7.59	2.306	2.88E-16
RELN	ENSG00000189056.13	4.395	0.61	1.745	2.80E-06
PLAC9	ENSG00000189129.13	3.15	8.273	-1.16	2.12E-11
HN1	ENSG00000189159.15	113.346	40.566	1.46	2.89E-17
PCDH18	ENSG00000189184.11	0.295	2.169	-1.291	1.20E-20
FAM180A	ENSG00000189320.8	0.115	2.145	-1.496	5.59E-40
KIAA0408	ENSG00000189367.14	7.075	0.38	2.549	3.31E-24
SH2D5	ENSG00000189410.11	1.58	0.195	1.11	2.05E-12
SPATS2L	ENSG00000196141.12	158.561	62.926	1.32	1.34E-17
SEMA4A	ENSG00000196189.12	3.755	11.965	-1.447	1.62E-14
GIMAP5	ENSG00000196329.10	4.165	13.21	-1.46	1.62E-11
ESRRG	ENSG00000196482.16	1.78	10.421	-2.039	7.24E-24
TPK1	ENSG00000196511.13	23.865	3.475	2.474	8.93E-27
MME	ENSG00000196549.10	4.295	47.33	-3.19	1.16E-11
SULF2	ENSG00000196562.14	121.25	38.468	1.631	3.81E-14
TCF4	ENSG00000196628.13	1.75	5.48	-1.237	1.76E-07
HRH1	ENSG00000196639.6	7.305	1.155	1.946	7.96E-20
GM2A	ENSG00000196743.8	28.445	10.23	1.391	2.06E-29
S100A2	ENSG00000196754.10	14.839	114.423	-2.865	7.28E-24
SMIM10L2B	ENSG00000196972.7	0.72	3.465	-1.376	2.17E-24
GAL3ST4	ENSG00000197093.10	3.465	0.82	1.295	1.88E-17
RP11-573D15.8	ENSG00000197099.8	0.04	4.904	-2.505	1.31E-78
SLC25A29	ENSG00000197119.12	20.095	47.511	-1.201	2.16E-11
HIST1H4J	ENSG00000197238.4	6.985	1.93	1.447	2.72E-09
FAM110D	ENSG00000197245.4	0.39	2.415	-1.297	5.86E-33
TPSB2	ENSG00000197253.13	19.235	3.59	2.14	4.42E-05
GATA3-AS1	ENSG00000197308.8	0	3.07	-2.025	2.85E-43
STMN3	ENSG00000197457.9	51.069	10.805	2.141	4.35E-12
PDGFA	ENSG00000197461.13	31.084	13.78	1.118	5.20E-09
SPN	ENSG00000197471.11	2.53	0.68	1.072	1.02E-12
GPX1P1	ENSG00000197582.5	4.66	1.57	1.139	4.00E-05
KCNMB2	ENSG00000197584.11	0.26	1.805	-1.155	1.53E-05
KLHL14	ENSG00000197705.9	0.685	2.965	-1.235	2.84E-11
S100A10	ENSG00000197747.8	642.275	294.688	1.121	1.84E-13
ZNF44	ENSG00000197857.13	5.63	14.292	-1.206	1.75E-20
SLC22A12	ENSG00000197891.11	4.775	77.437	-3.764	4.96E-14
SLC22A6	ENSG00000197901.11	0.235	58.7	-5.595	1.99E-12
PLCG2	ENSG00000197943.9	4.025	29.834	-2.617	1.91E-23
S100A6	ENSG00000197956.9	2322.398	926.983	1.324	8.09E-09
SIRPA	ENSG00000198053.11	54.026	22.265	1.242	1.82E-15
SULT1C4	ENSG00000198075.9	60.31	9.425	2.556	7.09E-09
PEG3	ENSG00000198300.12	0.32	2.025	-1.196	1.11E-15

FAM109A	ENSG00000198324.13	14.265	6.345	1.056	1.49E-15
TMEM207	ENSG00000198398.2	0	2.925	-1.973	5.15E-54
MT1F	ENSG00000198417.6	23.324	149.007	-2.625	8.76E-20
TPM2	ENSG00000198467.13	27.054	76.757	-1.471	1.40E-10
PLN	ENSG00000198523.5	0.57	6.85	-2.322	2.33E-21
SLC34A3	ENSG00000198569.9	0.1	5.791	-2.626	1.19E-40
MT-CYB	ENSG00000198727.2	9017.738	18341.829	-1.024	7.65E-05
PPP1R14C	ENSG00000198729.4	16.979	5.445	1.48	6.99E-06
MSRB1	ENSG00000198736.11	108.034	53.004	1.014	6.45E-05
SLC5A3	ENSG00000198743.5	2.605	10.995	-1.734	2.24E-19
MT-ND2	ENSG00000198763.3	8397.533	17330.792	-1.045	1.33E-05
RASSF9	ENSG00000198774.4	0.11	1.305	-1.054	3.70E-21
FAM169A	ENSG00000198780.11	0.065	1.155	-1.017	4.29E-22
ZNF521	ENSG00000198795.10	0.255	2.39	-1.434	1.88E-27
SUCNR1	ENSG00000198829.6	0.29	6.792	-2.595	3.47E-20
SELM	ENSG00000198832.10	20.735	107.314	-2.317	3.87E-13
TOX	ENSG00000198846.5	0.465	3.025	-1.458	1.19E-10
MT-ND1	ENSG00000198888.2	6652.818	15947.027	-1.261	8.23E-07
L1CAM	ENSG00000198910.12	1.465	23.342	-3.304	2.81E-09
NOS1AP	ENSG00000198929.12	0.23	1.83	-1.202	6.21E-20
GPRASP1	ENSG00000198932.12	1.48	4.785	-1.222	4.59E-14
SOWAHA	ENSG00000198944.5	0.91	4.415	-1.503	2.07E-16
RNU1-14P	ENSG00000199629.1	2.105	0	1.635	1.75E-05
RNU4-1	ENSG00000200795.1	3.489	0	2.166	2.56E-03
SERTAD4-AS1	ENSG00000203706.8	13.369	4.895	1.285	5.33E-07
SAMD5	ENSG00000203727.3	1.01	3.815	-1.26	2.17E-06
FCGR3A	ENSG00000203747.9	18.545	4.29	1.885	2.97E-19
PPAPDC1A	ENSG00000203805.10	1.12	6.97	-1.91	2.42E-16
SOX18	ENSG00000203883.6	1.71	11.196	-2.17	4.47E-33
NEU4	ENSG00000204099.11	0.14	1.704	-1.246	1.58E-10
PSMB8	ENSG00000204264.8	138.193	48.62	1.488	4.90E-32
NOTCH4	ENSG00000204301.6	2.955	13.975	-1.921	6.62E-29
SMIM5	ENSG00000204323.5	1.525	20.43	-3.085	8.24E-44
NEU1	ENSG00000204386.10	155.88	63.546	1.281	1.54E-24
LST1	ENSG00000204482.10	28.1	6.249	2.005	2.26E-17
MCCD1	ENSG00000204511.2	0.13	27.87	-4.675	1.19E-42
ZNF814	ENSG00000204514.9	5.69	13.359	-1.102	1.45E-11
PSORS1C1	ENSG00000204540.10	14.495	1.145	2.853	6.01E-27
HLA-J	ENSG00000204622.10	6.49	1.449	1.613	9.86E-10
HLA-G	ENSG00000204632.11	3.545	0.595	1.511	4.75E-09
HLA-F	ENSG00000204642.13	96.844	45.614	1.07	6.00E-08
TTC25	ENSG00000204815.8	5.745	2.294	1.034	6.14E-12
MROH6	ENSG00000204839.8	8.505	3.7	1.016	1.69E-04
TCTN1	ENSG00000204852.15	82.238	32.105	1.33	2.64E-20

ERICH4	ENSG00000204978.2	0.135	2.86	-1.766	4.15E-22
SYCE1L	ENSG00000205078.5	10.195	1.733	2.034	1.14E-12
PSMB10	ENSG00000205220.11	114.114	38.851	1.53	4.16E-23
MUC12	ENSG00000205277.9	7.665	0.23	2.817	4.35E-15
MT1H	ENSG00000205358.3	0.52	269.786	-7.477	1.98E-62
MT1M	ENSG00000205364.3	2.61	11.644	-1.808	1.92E-11
RGL3	ENSG00000205517.12	20.513	44.682	-1.086	1.10E-09
STAG3L1	ENSG00000205583.13	6.135	2.525	1.017	2.66E-05
GYG2P1	ENSG00000206159.10	0	1.764	-1.467	5.33E-23
HBA1	ENSG00000206172.8	16.475	114.091	-2.719	1.78E-09
HLA-H	ENSG00000206341.7	61.98	23.09	1.386	2.22E-11
HLA-A	ENSG00000206503.11	1575.615	541.474	1.539	6.37E-22
LNP1	ENSG00000206535.7	9.569	2.375	1.647	1.59E-06
GPX3	ENSG00000211445.11	392.894	4249.713	-3.432	1.03E-13
TRBV25-1	ENSG00000211751.7	7.64	2.032	1.511	7.27E-06
TRBC2	ENSG00000211772.8	13.619	3.655	1.651	1.55E-06
TRAV8-2	ENSG00000211786.3	0.11	1.48	-1.16	6.13E-19
RP11-64B16.2	ENSG00000213144.2	5.135	1.65	1.211	1.85E-13
LINC00671	ENSG00000213373.7	1.24	9.39	-2.214	2.92E-12
RPLP0P6	ENSG00000213553.4	16.75	7.765	1.018	6.34E-11
S1PR3	ENSG00000213694.3	1.415	10.025	-2.191	6.17E-20
SLC35F6	ENSG00000213699.8	41.865	18.514	1.135	8.72E-23
SLC23A3	ENSG00000213901.10	2.625	9.906	-1.589	1.69E-10
LIPE-AS1	ENSG00000213904.8	11.555	5.185	1.021	1.30E-08
ITGA1	ENSG00000213949.8	4.11	11.337	-1.272	1.52E-07
TAX1BP3	ENSG00000213977.7	178.118	88.043	1.008	5.22E-11
TMEM213	ENSG00000214128.10	0.325	64.691	-5.632	9.90E-61
LINC00887	ENSG00000214145.6	2.7	0.2	1.624	2.53E-14
NEURL1B	ENSG00000214357.8	1.22	4.89	-1.408	1.99E-12
PLIN5	ENSG00000214456.8	3.185	8.015	-1.107	4.30E-14
MEG3	ENSG00000214548.14	0.685	9.864	-2.689	2.16E-36
RP11-480I12.5	ENSG00000214796.8	0.84	3.358	-1.244	1.92E-15
UBE2QL1	ENSG00000215218.3	0.05	2.055	-1.541	2.03E-33
GCGR	ENSG00000215644.9	0.06	16.91	-4.079	3.61E-63
RPS2P55	ENSG00000216866.5	3.845	1.23	1.119	7.12E-26
FTH1P8	ENSG00000219507.4	11.755	4.47	1.222	1.26E-11
PPP1R3G	ENSG00000219607.3	1.95	0.42	1.055	4.96E-12
MTMR9LP	ENSG00000220785.7	2.22	7.209	-1.35	7.09E-13
SLC12A8	ENSG00000221955.10	7.6	3.298	1.001	1.46E-10
LINC00473	ENSG00000223414.2	0	3.419	-2.144	2.20E-44
MIR503HG	ENSG00000223749.7	0.895	9.495	-2.469	4.20E-27
RP11-54O7.3	ENSG00000223764.2	0.4	2.789	-1.436	4.57E-07
RP11-554I8.2	ENSG00000223784.1	2.5	0.13	1.631	1.28E-25
IL10RB-AS1	ENSG00000223799.1	4.54	1.74	1.016	1.08E-15

HLA-DPB1	ENSG00000223865.10	169.853	81.435	1.051	7.87E-08
HAGLR	ENSG00000224189.6	2.48	10.204	-1.687	3.03E-09
LINC01272	ENSG00000224397.5	4.03	0.485	1.76	9.57E-15
LINC00240	ENSG00000224843.6	5.52	1.765	1.238	6.44E-10
HSPA7	ENSG00000225217.1	3.735	0.98	1.258	1.39E-05
TMEM246-AS1	ENSG00000225376.5	0.44	3.495	-1.642	1.89E-16
LINC01504	ENSG00000225434.2	3.115	0.655	1.314	2.59E-13
LINC00475	ENSG00000225511.6	2.48	0.18	1.56	2.53E-18
RPL35P5	ENSG00000225573.4	6.52	2.509	1.099	8.65E-19
MTND2P28	ENSG00000225630.1	283.537	628.818	-1.146	2.42E-04
UQCRFS1P1	ENSG00000226085.3	2.65	6.925	-1.118	6.46E-03
NPY6R	ENSG00000226306.6	6.465	1.669	1.484	1.06E-08
PARD3-AS1	ENSG00000226386.1	3.08	0.93	1.08	3.62E-08
FTH1P20	ENSG00000226564.1	15.49	5.304	1.387	5.21E-18
RP11-128M1.1	ENSG00000226644.5	1.57	0.235	1.057	1.99E-12
TEX41	ENSG00000226674.8	0.835	6.122	-1.956	5.50E-11
RP11-397G17.1	ENSG00000226733.1	0	3.484	-2.165	9.26E-32
ERVMER34-1	ENSG00000226887.7	0.285	2.665	-1.512	3.43E-32
RP3-417G15.1	ENSG00000227008.2	4.72	1.745	1.059	1.52E-08
LINC00511	ENSG00000227036.6	6.245	0.895	1.935	1.91E-14
RP11-395B7.4	ENSG00000227053.1	9.335	0.38	2.905	6.93E-14
LINC01268	ENSG00000227502.2	2.445	0.37	1.33	4.26E-10
HCG4P5	ENSG00000227766.1	3.645	1.152	1.11	7.66E-06
RP11-807H17.1	ENSG00000227869.1	30.708	2.9	3.023	2.51E-10
LINC00864	ENSG00000228055.2	0.28	3.745	-1.89	6.87E-27
RP11-251M1.1	ENSG00000228401.4	0.16	1.725	-1.232	8.89E-57
RP3-434P1.6	ENSG00000228620.1	1.37	0.04	1.188	2.19E-16
HNF4A-AS1	ENSG00000229005.2	0.055	1.338	-1.148	5.35E-18
RP5-952N6.1	ENSG00000229051.1	0	1.1	-1.07	1.39E-36
NPM1P25	ENSG00000229417.1	0.93	4.815	-1.591	3.47E-24
LINC00379	ENSG00000229557.1	0.8	3.245	-1.238	1.53E-10
LINC01315	ENSG00000229891.1	7.495	2.015	1.494	4.86E-15
RP11-284F21.7	ENSG00000229953.1	4.275	0.23	2.1	4.18E-25
RP11-550H2.2	ENSG00000230027.1	0	1.77	-1.47	4.02E-49
RP4-737E23.2	ENSG00000230387.2	0.02	1.359	-1.209	1.78E-44
GPAA1P2	ENSG00000230596.2	0.595	3	-1.326	1.26E-13
RP11-359N11.1	ENSG00000230631.1	0.1	1.968	-1.432	1.02E-33
LINC01508	ENSG00000231107.1	4.985	1.815	1.088	7.66E-12
TAPBP	ENSG00000231925.11	140.691	47.28	1.553	8.73E-27
RP11-149I23.3	ENSG00000232110.7	32.089	4.295	2.644	8.71E-15
FTH1P7	ENSG00000232187.1	27.025	9.4	1.43	1.82E-16
SEMA3B-AS1	ENSG00000232352.1	1.805	4.804	-1.049	2.83E-14
RP11-14N7.2	ENSG00000232527.7	36.81	10.179	1.758	3.58E-15
RPL3P4	ENSG00000232573.1	56.883	25.864	1.107	1.80E-04

HLA-DQB2	ENSG00000232629.8	3.21	1.06	1.031	1.60E-06
ZFY-AS1	ENSG00000233070.1	0.06	1.924	-1.464	3.46E-12
HOXB-AS3	ENSG00000233101.10	2.98	22.169	-2.541	4.53E-25
RP4-655J12.4	ENSG00000233154.5	0.13	7.15	-2.85	4.73E-67
LINC00472	ENSG00000233237.6	1.64	6.252	-1.458	1.24E-17
GPX1	ENSG00000233276.3	698.152	231.504	1.588	1.96E-28
GAS6-AS1	ENSG00000233695.2	1.495	0.16	1.105	4.99E-13
LINC00284	ENSG00000233725.7	0.19	1.555	-1.102	3.69E-12
RP11-255H23.2	ENSG00000233836.7	0.775	2.845	-1.115	4.43E-20
LINC01503	ENSG00000233901.5	4.91	22.251	-1.976	1.14E-16
RP5-887A10.1	ENSG00000234184.5	19.11	2.835	2.391	5.05E-10
MAGI2-AS3	ENSG00000234456.7	12.36	25.754	-1.002	1.81E-05
PINLYP	ENSG00000234465.10	2.12	13.405	-2.207	1.32E-08
RP3-510D11.2	ENSG00000234546.2	1.81	0.39	1.015	1.24E-18
NUS1P2	ENSG00000234685.1	19.932	2.819	2.454	3.04E-06
HLA-B	ENSG00000234745.9	1348.275	489.714	1.459	2.56E-15
FABP5P7	ENSG00000234964.4	10.615	3.73	1.296	4.36E-06
RP4-763G1.2	ENSG00000235200.1	0.17	2.005	-1.361	5.14E-32
MCF2L-AS1	ENSG00000235280.2	0.05	2.54	-1.753	3.95E-21
LINC01055	ENSG00000235366.2	0	8.119	-3.189	1.92E-49
RP11-91K11.2	ENSG00000235434.1	0.12	1.4	-1.099	8.24E-09
MSC-AS1	ENSG00000235531.9	5.395	1.669	1.261	5.02E-10
LINC00494	ENSG00000235621.8	2.49	0.57	1.153	1.10E-11
KIAA0040	ENSG00000235750.9	7.31	15.945	-1.028	1.83E-07
HSPB1P1	ENSG00000236060.2	13.91	2.619	2.043	7.86E-24
RP11-231P20.2	ENSG00000236155.6	2.945	7.36	-1.083	2.50E-14
ZEB1-AS1	ENSG00000237036.4	9.355	3.783	1.114	1.41E-11
NR2F1-AS1	ENSG00000237187.8	0.845	8.185	-2.316	1.69E-38
LINC00857	ENSG00000237523.1	4.475	0.83	1.581	3.98E-14
RP5-1018K9.1	ENSG00000237707.1	0	2.215	-1.685	2.14E-47
PAGR1	ENSG00000238045.9	10.335	4.54	1.033	3.67E-13
IFNWP19	ENSG00000238271.2	1.54	0.24	1.035	6.70E-08
RP11-245J24.1	ENSG00000238276.5	0	1.325	-1.217	2.15E-16
SNORD10	ENSG00000238917.1	2.765	0	1.913	2.64E-05
GATSL3	ENSG00000239282.7	4.38	11.194	-1.18	4.30E-18
NME1	ENSG00000239672.7	112.341	39.36	1.49	8.12E-33
LILRA4	ENSG00000239961.2	1.26	0.055	1.099	2.33E-09
LILRA2	ENSG00000239998.5	3.37	0.725	1.341	4.14E-14
PSMB9	ENSG00000240065.7	74.829	22.849	1.669	8.48E-22
PCDHGC3	ENSG00000240184.6	49.86	20.525	1.241	5.29E-14
PNMA2	ENSG00000240694.8	9.7	1.745	1.963	3.64E-22
KRBOX1	ENSG00000240747.7	0.47	2.464	-1.236	3.55E-03
UGT1A9	ENSG00000241119.1	0.94	77.04	-5.33	7.79E-10
TDGF1	ENSG00000241186.7	0.08	9.72	-3.311	1.79E-27

FLJ22763	ENSG00000241224.6	0.64	4.645	-1.783	2.04E-06
RPL37P6	ENSG00000241431.1	2.425	0.335	1.359	4.29E-14
INMT	ENSG00000241644.2	1.475	7.4	-1.763	1.42E-18
RP13-452N2.1	ENSG00000242048.3	0.02	6.708	-2.918	1.88E-61
RP11-302F12.1	ENSG00000242640.1	2.935	0	1.976	1.10E-05
STON1	ENSG00000243244.5	1.605	6.565	-1.538	2.01E-17
VSIG8	ENSG00000243284.1	0.03	1.82	-1.453	1.54E-51
RP11-379F12.3	ENSG00000243350.1	0	1.235	-1.16	9.13E-22
NME1-NME2	ENSG00000243678.11	793.369	327.862	1.272	5.35E-24
MRPS6	ENSG00000243927.5	35.041	88.76	-1.316	5.87E-22
GSTA1	ENSG00000243955.5	2.33	76.553	-4.542	7.79E-16
RPS24P17	ENSG00000243988.1	0	3.524	-2.178	2.02E-45
GSTA2	ENSG00000244067.2	0.32	24.061	-4.247	1.27E-27
LINC01322	ENSG00000244128.5	2.585	0.22	1.555	4.08E-15
GATA2-AS1	ENSG00000244300.2	0.22	1.825	-1.211	1.08E-13
MTHFD2P1	ENSG00000244681.1	2.085	0.11	1.475	3.42E-12
FCGR2C	ENSG00000244682.7	3.785	1.05	1.223	1.01E-03
PTCHD4	ENSG00000244694.7	3.25	0.36	1.644	1.32E-14
HBB	ENSG00000244734.3	23.625	207.671	-3.083	4.27E-11
SOCS2-AS1	ENSG00000246985.7	0.515	2.385	-1.16	5.34E-22
MIR210HG	ENSG00000247095.2	13.485	5.029	1.265	5.42E-04
TWF2	ENSG00000247596.8	53.031	24.432	1.087	2.24E-21
PCED1B-AS1	ENSG00000247774.6	10.104	2.7	1.585	3.60E-13
RP11-421F16.3	ENSG00000247903.1	12.52	4.99	1.175	9.47E-15
FOXD1-AS1	ENSG00000247993.2	0.16	1.97	-1.356	6.23E-13
RP11-752D24.2	ENSG00000248115.1	0.01	5.355	-2.654	2.92E-56
LINC01197	ENSG00000248441.6	0.495	2.054	-1.03	2.01E-11
RP11-386B13.4	ENSG00000248517.1	0	2.775	-1.916	3.13E-68
RP13-644M16.5	ENSG00000248763.2	2.4	0.265	1.426	6.10E-10
RP11-362F19.1	ENSG00000248810.1	0.38	3.265	-1.628	3.25E-36
TNFSF12-TNFSF13	ENSG00000248871.1	2.185	0.4	1.186	1.77E-06
RP11-317M11.1	ENSG00000249341.1	0	1.834	-1.503	1.01E-44
LINC01187	ENSG00000249601.2	0	8.707	-3.279	3.00E-73
ECSCR	ENSG00000249751.3	3.04	14.815	-1.969	3.63E-25
PVT1	ENSG00000249859.7	7.229	1.14	1.943	1.32E-11
RP11-256P1.1	ENSG00000249971.1	3.08	0	2.029	3.94E-06
RP11-789C1.1	ENSG00000250266.1	0	1.209	-1.144	2.80E-34
TUNAR	ENSG00000250366.2	1.435	0.115	1.127	3.48E-07
RP11-834C11.5	ENSG00000250432.5	0.515	3.684	-1.629	3.81E-35
IQCJ-SCHIP1	ENSG00000250588.6	41.72	11.665	1.754	2.82E-09
SEPP1	ENSG00000250722.5	77.842	206.399	-1.395	1.39E-06
RP11-834C11.4	ENSG00000250742.1	12.415	29.497	-1.185	7.17E-10
RP11-63A11.1	ENSG00000250781.1	0.325	2.925	-1.567	3.80E-16
PRODH2	ENSG00000250799.9	2.313	62.736	-4.266	1.88E-17

ZFPM2-AS1	ENSG00000251003.7	4.975	0.685	1.826	3.23E-09
F11-AS1	ENSG00000251165.5	0.02	2.155	-1.629	6.63E-47
ERVH-1	ENSG00000251292.1	2.565	0.43	1.318	1.26E-11
RP11-81H14.2	ENSG00000251301.6	0.25	1.98	-1.253	1.03E-13
SHANK3	ENSG00000251322.7	3.01	10.829	-1.561	6.58E-23
LINC01094	ENSG00000251442.5	1.86	0.32	1.116	4.13E-25
FOXD1	ENSG00000251493.3	0.13	1.58	-1.191	6.10E-11
RP11-119D9.1	ENSG00000251637.6	0.925	3.555	-1.243	2.70E-05
LINC01606	ENSG00000253301.5	0	5.88	-2.782	5.96E-56
TRNP1	ENSG00000253368.3	4.355	13.354	-1.423	7.97E-03
RBPMS-AS1	ENSG00000254109.5	9.38	3.375	1.246	4.49E-11
LINC01485	ENSG00000254211.5	0.075	3.19	-1.963	6.63E-26
HSPB2-C11orf52	ENSG00000254445.1	10.225	3.93	1.187	1.07E-05
RP5-1024C24.1	ENSG00000254489.1	0	1.58	-1.367	7.06E-67
RP11-728F11.4	ENSG00000254528.7	2.895	26.88	-2.839	4.81E-37
RP11-468E2.1	ENSG00000254692.1	11.695	5.189	1.036	2.56E-03
RP11-396O20.1	ENSG00000254695.1	0	2.435	-1.78	3.01E-50
RP11-565P22.6	ENSG00000254706.2	0.26	2.31	-1.393	1.23E-17
RP11-805J14.5	ENSG00000254721.1	11.905	5.34	1.025	8.23E-19
RP11-531H8.2	ENSG00000254789.1	0	1.69	-1.427	2.89E-64
NAV2-AS1	ENSG00000254894.1	0.31	1.665	-1.025	9.13E-19
RP11-326C3.2	ENSG00000255026.1	21.073	3.351	2.343	4.85E-03
GRM5-AS1	ENSG00000255082.1	1.63	0.16	1.181	1.07E-07
RP11-166D19.1	ENSG00000255248.6	0.68	3.429	-1.399	6.93E-20
SOGA3	ENSG00000255330.8	2.195	0.185	1.431	2.70E-14
RP11-732A19.5	ENSG00000255390.1	1.895	4.934	-1.036	3.01E-08
RP11-627G23.1	ENSG00000255545.7	0.395	2.45	-1.306	8.94E-13
RP11-190A12.7	ENSG00000256029.5	0	3.213	-2.075	4.91E-52
RP11-116G8.5	ENSG00000256462.1	0.015	2.381	-1.736	4.01E-29
SALL3	ENSG00000256463.8	0	1.32	-1.214	2.87E-24
RP13-895J2.3	ENSG00000256542.2	0.13	1.7	-1.257	1.91E-18
RP11-611O2.3	ENSG00000256664.1	9.785	4.25	1.039	4.58E-07
LIMS3L	ENSG00000256671.6	0.42	4.635	-1.988	1.19E-30
RP11-783K16.5	ENSG00000256940.1	5.215	2.025	1.039	5.76E-09
U47924.27	ENSG00000257084.1	0	4.08	-2.345	3.83E-36
NHLRC4	ENSG00000257108.1	0.325	6.3	-2.462	2.93E-46
RP11-611O2.5	ENSG00000257181.1	3.01	0.794	1.16	2.35E-04
MGAM	ENSG00000257335.8	0.34	3.88	-1.865	6.72E-09
RP11-579D7.2	ENSG00000257653.1	3.245	0.18	1.847	7.55E-06
RP11-620J15.3	ENSG00000257698.1	33.306	13.068	1.286	1.56E-16
LBX2-AS1	ENSG00000257702.3	17.81	4.316	1.823	1.14E-21
RP11-1143G9.4	ENSG00000257764.2	13.365	0.404	3.355	5.43E-09
RP11-626P14.1	ENSG00000257845.1	0.59	2.659	-1.202	3.35E-03
RP11-386G11.10	ENSG00000258017.1	113.397	26.375	2.063	7.30E-10

RP11-347C12.3	ENSG00000258130.7	5.075	1.98	1.028	1.24E-04
RP11-161H23.5	ENSG00000258232.2	139.768	43.576	1.659	4.17E-22
RP11-644F5.10	ENSG00000258311.5	3.475	1.11	1.085	6.59E-07
RP11-649E7.5	ENSG00000258377.1	2.995	0.305	1.614	1.12E-03
PDF	ENSG00000258429.1	23.709	10.415	1.114	1.96E-08
RP11-192H23.4	ENSG00000258472.8	1.175	5.713	-1.626	9.36E-25
LINC00645	ENSG00000258548.5	0.06	4.593	-2.4	2.23E-48
RP11-369C8.1	ENSG00000258616.5	0.03	2.285	-1.673	1.18E-06
RP11-841O20.2	ENSG00000258757.1	1.89	0	1.531	1.07E-07
TUBB3	ENSG00000258947.6	42.413	1.44	4.153	8.67E-26
PTCSC3	ENSG00000259104.2	0.11	1.654	-1.257	1.78E-21
RP11-298I3.5	ENSG00000259132.1	11.52	4.4	1.213	1.86E-07
LINC00924	ENSG00000259134.5	0.505	3.398	-1.547	2.39E-19
RP11-50C13.1	ENSG00000259250.1	4.485	1.36	1.217	7.74E-10
RP11-617F23.1	ENSG00000259291.2	10.045	26.345	-1.308	1.06E-15
RP11-798K3.2	ENSG00000259347.5	1.135	3.789	-1.165	2.44E-03
RP11-316M1.12	ENSG00000259357.2	28.899	1.87	3.381	4.12E-11
RP11-736N17.4	ENSG00000259374.2	9.789	2.145	1.778	6.79E-05
SORD2P	ENSG00000259479.6	4.07	9.745	-1.084	4.07E-06
RP11-244F12.2	ENSG00000259627.1	1.575	0	1.365	1.37E-04
PWRN1	ENSG00000259905.5	0.115	1.569	-1.204	3.99E-15
RP11-999E24.3	ENSG00000259969.1	2.285	10.04	-1.749	4.72E-24
RP11-20I23.1	ENSG00000260272.1	1.54	0.115	1.188	1.87E-11
MT1L	ENSG00000260549.1	4.865	17.418	-1.651	5.51E-10
RP11-49I11.1	ENSG00000260552.1	5.625	1.575	1.363	1.89E-14
RP11-24N18.1	ENSG00000260570.1	14.619	4.339	1.549	4.87E-03
RP11-161M6.2	ENSG00000260807.6	3.18	1.055	1.024	1.71E-06
WFDC21P	ENSG00000261040.6	11.335	0.67	2.885	1.95E-17
TMEM178B	ENSG00000261115.5	5.235	0.895	1.718	2.72E-19
RP3-523K23.2	ENSG00000261116.1	0.04	1.87	-1.464	8.95E-18
RP11-532F12.5	ENSG00000261183.5	12.7	27.642	-1.064	3.17E-11
RP11-389C8.2	ENSG00000261269.1	0.475	2.395	-1.203	8.15E-29
PECAM1	ENSG00000261371.5	10.14	36.479	-1.75	4.67E-09
LA16c-329F2.1	ENSG00000261399.1	0.855	10.658	-2.652	2.37E-22
RP11-527L4.2	ENSG00000261514.1	0	1.204	-1.14	4.61E-41
RP11-554A11.4	ENSG00000261625.1	0.02	1.265	-1.151	3.15E-66
RP11-352D13.6	ENSG00000261634.3	0.895	4.335	-1.493	4.59E-21
LA16c-390E6.5	ENSG00000261641.2	18.638	8.76	1.009	1.94E-04
RP11-265N6.1	ENSG00000261684.1	2.965	0.449	1.452	3.12E-08
RP11-626G11.3	ENSG00000261759.1	12.66	5.143	1.153	5.93E-06
RP11-1260E13.4	ENSG00000262061.5	0.2	1.565	-1.096	6.52E-48
RP11-498C9.3	ENSG00000262413.1	2.939	0.74	1.179	1.00E-03
RP11-667K14.4	ENSG00000262533.1	8.825	0.453	2.757	8.80E-09
SPON1	ENSG00000262655.3	22.407	6.79	1.587	3.40E-04

RP11-1055B8.4	ENSG00000262877.4	0.97	3.565	-1.212	1.81E-05
RP11-1260E13.1	ENSG00000262920.5	0	1.524	-1.336	1.59E-10
RP11-294J22.6	ENSG00000262967.1	4.63	0.568	1.844	7.37E-04
RP11-849I19.1	ENSG00000263146.2	0	5.02	-2.59	2.55E-37
MYZAP	ENSG00000263155.5	0.235	2.589	-1.539	7.88E-27
RP11-810M2.2	ENSG00000263165.1	24.525	9.554	1.274	9.76E-05
RP11-473I1.9	ENSG00000263244.2	3.835	1.37	1.029	1.19E-04
LINC00675	ENSG00000263429.3	0.02	1.442	-1.26	1.86E-41
IKBKE	ENSG00000263528.7	5.495	2.08	1.076	2.70E-19
RP11-334E6.12	ENSG00000263873.1	3.75	37.866	-3.033	1.83E-16
RP11-746M1.1	ENSG00000263986.1	43.11	10.902	1.89	3.12E-05
RP11-6N17.6	ENSG00000264019.1	13.23	0.795	2.987	2.88E-06
LINC00864	ENSG00000264404.2	1.03	4.23	-1.365	1.30E-05
RP11-138C9.1	ENSG00000264558.1	16.65	7.3	1.089	1.70E-05
RP11-192H23.8	ENSG00000264608.1	1.47	5.095	-1.303	3.64E-18
GJA5	ENSG00000265107.2	1.355	12.79	-2.55	4.29E-30
RP11-690G19.4	ENSG00000265460.6	0	9.751	-3.426	5.97E-83
CTSLP2	ENSG00000266217.2	0.205	1.535	-1.073	2.06E-07
RP5-890E16.4	ENSG00000266341.1	7.085	1.443	1.727	4.46E-03
RP1-56K13.5	ENSG00000266588.1	0.075	1.642	-1.297	6.02E-29
FXYD1	ENSG00000266964.5	1.63	16.448	-2.73	1.58E-22
PCAT19	ENSG00000267107.6	0.42	2.422	-1.269	1.58E-34
TBX2-AS1	ENSG00000267280.5	1.28	9.855	-2.251	7.51E-22
ZNF582-AS1	ENSG00000267454.5	1.51	5.19	-1.302	4.15E-19
LUZP6	ENSG00000267697.1	25.37	8.269	1.508	1.44E-04
SMIM22	ENSG00000267795.5	1.425	24.41	-3.389	2.01E-10
ZNF350-AS1	ENSG00000269235.1	0.12	2.085	-1.462	2.49E-20
RP3-333A15.2	ENSG00000269933.1	0	5.028	-2.592	1.15E-58
RP11-394O4.5	ENSG00000269936.3	0.29	4.14	-1.994	6.34E-35
MEI4	ENSG00000269964.2	0.16	1.41	-1.055	3.80E-13
RP5-940J5.9	ENSG00000269968.1	34.701	1.137	4.063	2.73E-05
RP1-232P20.1	ENSG00000269985.1	2.405	0.14	1.579	3.03E-12
RP11-420L9.5	ENSG00000270504.1	4.79	1.68	1.111	5.20E-20
NUDT4P2	ENSG00000271121.2	4.635	13.356	-1.349	5.05E-04
MILR1	ENSG00000271605.5	3.02	0.98	1.022	4.11E-11
RP11-1C8.7	ENSG00000271830.1	1.605	0	1.381	8.30E-11
RP11-284F21.9	ENSG00000272068.1	7.359	0.09	2.939	1.03E-30
RP1-261G23.7	ENSG00000272114.1	8.69	42.833	-2.177	3.59E-07
RP11-465B22.8	ENSG00000272141.1	2.18	5.744	-1.085	1.12E-06
RP11-284F21.10	ENSG00000272405.1	9.885	0.21	3.169	7.51E-38
RP4-680D5.8	ENSG00000272510.1	0.76	3.425	-1.33	1.09E-18
MUSTN1	ENSG00000272573.5	1.035	5.645	-1.707	8.79E-19
RP11-343C2.12	ENSG00000272617.1	21.565	9.14	1.154	2.56E-17
RP11-259N19.1	ENSG00000272711.1	1.945	0.175	1.326	4.39E-28

RP11-357H14.17	ENSG00000272763.1	4.6	1.225	1.332	1.09E-05
RP11-286H15.1	ENSG00000272789.1	0.26	1.945	-1.225	4.97E-30
RP11-309L24.4	ENSG00000272899.2	6.455	2.044	1.292	1.26E-04
RP11-1191J2.5	ENSG00000272927.1	3.24	1.075	1.031	4.86E-05
RP11-134L10.1	ENSG00000272941.1	8.04	1.855	1.663	2.26E-25
RP11-20I20.4	ENSG00000273179.1	15.625	1.04	3.027	5.08E-14
RP11-61L19.2	ENSG00000273335.1	0.21	5.815	-2.494	1.80E-51
LHX1	ENSG00000273706.4	0.06	6.924	-2.902	3.37E-27
RP11-115H13.1	ENSG00000273906.1	0	1.34	-1.226	7.13E-24
RP4-568C11.4	ENSG00000274173.1	0.04	14.147	-3.864	1.21E-55
RP11-147L13.12	ENSG00000274561.1	7.995	3.455	1.014	6.50E-06
RP11-35J10.6	ENSG00000275612.1	0	1.995	-1.583	5.92E-29
RP4-545L17.11	ENSG00000276026.1	1.42	0.196	1.017	6.28E-07
RAB7B	ENSG00000276600.4	2.39	0.375	1.302	1.75E-15
RP11-35J10.7	ENSG00000276668.1	0	3.015	-2.005	6.44E-34
RP5-875H18.9	ENSG00000276851.1	2.115	0.41	1.144	1.35E-06
RP13-895J2.6	ENSG00000277011.1	0.05	4.577	-2.409	5.14E-45
RP11-445F12.1	ENSG00000277268.1	0.06	14.535	-3.873	5.93E-36
SRCIN1	ENSG00000277363.4	4.14	0.62	1.666	2.02E-14
MARCKS	ENSG00000277443.1	40.115	11.318	1.739	1.06E-19
GPIHBP1	ENSG00000277494.1	0.7	4.775	-1.764	3.52E-37
RP11-434E6.4	ENSG00000277559.1	2.44	0.3	1.404	1.12E-17
NEFL	ENSG00000277586.1	30.595	2.55	3.154	1.68E-09
RP11-295M3.4	ENSG00000277639.1	4.405	11.467	-1.206	1.71E-04
RP11-452N17.1	ENSG00000277998.1	40.104	8.28	2.147	6.60E-16
RP13-608F4.5	ENSG00000278603.1	13.75	5.754	1.127	4.20E-05
RP11-12A20.10	ENSG00000278735.1	0	5.055	-2.598	5.18E-27
RP13-895J2.4	ENSG00000278872.1	0.095	1.615	-1.256	6.74E-27
RP11-799B12.1	ENSG00000279332.1	2.965	0.85	1.1	4.54E-12
RP11-671J11.5	ENSG00000279423.1	29.93	13.985	1.045	7.84E-18
RP11-185E8.2	ENSG00000279673.1	1.81	0.32	1.09	3.81E-25
RP11-205M5.3	ENSG00000280064.1	17.16	6.84	1.212	1.63E-07
LINC01230	ENSG00000281769.1	0	2.115	-1.639	2.38E-62
TMEM265	ENSG00000281991.1	23.219	9.405	1.219	1.83E-06
RP11-27G14.4	ENSG00000282221.1	0.4	2.905	-1.48	2.76E-09
RP3-335E1.1	ENSG00000282408.1	0	2.96	-1.985	7.21E-46
ADORA3	ENSG00000282608.1	4.485	0.575	1.8	6.54E-20

Table S4

normal	tumor
TCGA-GL-6846-11A-01R-1965-07	TCGA-Y8-A8S1-01A-11R-A37K-07
TCGA-P4-A5ED-11A-11R-A28H-07	TCGA-Y8-A8S0-01A-11R-A37K-07
TCGA-BQ-7059-11A-01R-1965-07	TCGA-Y8-A8RZ-01A-11R-A37K-07
TCGA-GL-A9DE-11A-11R-A37K-07	TCGA-Y8-A8RY-01A-11R-A37K-07
TCGA-BQ-5879-11A-01R-1592-07	TCGA-Y8-A898-01A-11R-A355-07
TCGA-BQ-5891-11A-01R-1592-07	TCGA-Y8-A897-01A-11R-A36F-07
TCGA-BQ-5877-11A-01R-1592-07	TCGA-Y8-A896-01A-11R-A36F-07
TCGA-GL-A59R-11A-11R-A26U-07	TCGA-Y8-A895-01A-11R-A36F-07
TCGA-BQ-7051-11A-02R-1965-07	TCGA-Y8-A894-01A-11R-A36F-07
TCGA-BQ-7046-11A-01R-1965-07	TCGA-WN-AB4C-01A-11R-A42S-07
TCGA-BQ-7044-11A-01R-1965-07	TCGA-WN-A9G9-01A-12R-A37K-07
TCGA-BQ-5884-11A-01R-1592-07	TCGA-V9-A7HT-01A-11R-A33Z-07
TCGA-DZ-6132-11A-01R-1965-07	TCGA-UZ-A9Q1-01A-11R-A42S-07
TCGA-BQ-5882-11A-01R-1592-07	TCGA-UZ-A9Q0-01A-12R-A42S-07
TCGA-A4-A4ZT-11A-11R-A26U-07	TCGA-UZ-A9PZ-01A-11R-A42S-07
TCGA-BQ-5888-11A-01R-1592-07	TCGA-UZ-A9PX-01A-11R-A42S-07
TCGA-DZ-6134-11A-01R-1965-07	TCGA-UZ-A9PV-01A-11R-A42S-07
TCGA-BQ-7055-11A-01R-1965-07	TCGA-UZ-A9PU-01A-11R-A42S-07
TCGA-BQ-5890-11A-01R-1592-07	TCGA-UZ-A9PS-01A-11R-A42S-07
TCGA-P4-A5E8-11A-12R-A28H-07	TCGA-UZ-A9PR-01A-11R-A42S-07
TCGA-A4-A57E-11A-11R-A26U-07	TCGA-UZ-A9PQ-01A-11R-A42S-07
TCGA-BQ-7045-11A-01R-1965-07	TCGA-UZ-A9PP-01A-11R-A42S-07
TCGA-DZ-6133-11A-01R-1965-07	TCGA-UZ-A9PO-01A-11R-A38C-07
TCGA-GL-7966-11A-01R-2204-07	TCGA-UZ-A9PN-01A-11R-A38C-07
TCGA-B9-4115-11A-01R-1758-07	TCGA-UZ-A9PM-01A-21R-A38C-07
TCGA-BQ-5894-11A-01R-1592-07	TCGA-UZ-A9PL-01A-11R-A38C-07
TCGA-DZ-6131-11A-01R-1965-07	TCGA-UZ-A9PK-01A-11R-A38C-07
TCGA-BQ-5887-11A-01R-1965-07	TCGA-UZ-A9PJ-01A-11R-A38C-07
TCGA-BQ-5878-11A-01R-1592-07	TCGA-UN-AAZ9-01A-11R-A38C-07
TCGA-Y8-A8RY-11A-11R-A37K-07	TCGA-SX-A7SU-01A-11R-A36F-07
TCGA-BQ-7061-11A-01R-1965-07	TCGA-SX-A7SS-01A-11R-A36F-07
TCGA-BQ-5875-11A-01R-1592-07	TCGA-SX-A7SR-01A-12R-A36F-07
	TCGA-SX-A7SQ-01A-12R-A36F-07
	TCGA-SX-A7SP-01A-11R-A355-07
	TCGA-SX-A7SO-01A-11R-A355-07
	TCGA-SX-A7SN-01A-11R-A355-07
	TCGA-SX-A7SM-01A-11R-A355-07
	TCGA-SX-A7SL-01A-11R-A355-07
	TCGA-SX-A71W-01A-12R-A355-07
	TCGA-SX-A71V-01A-11R-A33Z-07
	TCGA-SX-A71U-01A-12R-A33Z-07
	TCGA-SX-A71S-01A-11R-A33Z-07
	TCGA-SX-A71R-01A-12R-A33Z-07

TCGA-Q2-A5QZ-01A-11R-A28H-07

TCGA-PJ-A5Z9-01A-11R-A28H-07

TCGA-PJ-A5Z8-01A-11R-A28H-07

TCGA-P4-AAVM-01A-11R-A42S-07

TCGA-P4-AAVL-01A-11R-A42S-07

TCGA-P4-AAVK-01A-11R-A42S-07

TCGA-P4-A5ED-01A-11R-A28H-07

TCGA-P4-A5EB-01A-11R-A28H-07

TCGA-P4-A5EA-01A-11R-A28H-07

TCGA-P4-A5E8-01A-11R-A28H-07

TCGA-P4-A5E7-01A-31R-A28H-07

TCGA-P4-A5E6-01A-11R-A28H-07

TCGA-O9-A75Z-01A-11R-A33Z-07

TCGA-MH-A856-01A-11R-A355-07

TCGA-MH-A855-01A-11R-A355-07

TCGA-MH-A854-01A-11R-A355-07

TCGA-MH-A562-01A-11R-A26U-07

TCGA-MH-A561-01A-11R-A26U-07

TCGA-MH-A560-01A-11R-A26U-07

TCGA-MH-A55Z-01A-11R-A26U-07

TCGA-MH-A55W-01A-11R-A26U-07

TCGA-KV-A74V-01A-11R-A33Z-07

TCGA-KV-A6GE-01A-11R-A31O-07

TCGA-KV-A6GD-01A-11R-A31O-07

TCGA-J7-8537-01A-11R-2404-07

TCGA-J7-6720-01A-11R-2139-07

TCGA-IZ-A6M9-01A-11R-A31O-07

TCGA-IZ-A6M8-01A-11R-A31O-07

TCGA-IZ-8196-01A-11R-2404-07

TCGA-IZ-8195-01A-31R-2404-07

TCGA-IA-A83W-01A-11R-A355-07

TCGA-IA-A83V-01A-11R-A355-07

TCGA-IA-A83T-01A-11R-A355-07

TCGA-IA-A83S-01A-11R-A355-07

TCGA-IA-A40Y-01A-11R-A24Z-07

TCGA-IA-A40X-01A-11R-A24Z-07

TCGA-IA-A40U-01A-11R-A24Z-07

TCGA-HE-A5NL-01A-11R-A26U-07

TCGA-HE-A5NJ-01A-11R-A26U-07

TCGA-HE-A5NI-01A-11R-A26U-07

TCGA-HE-A5NH-01A-11R-A26U-07

TCGA-HE-A5NF-01A-11R-A26U-07

TCGA-HE-7129-01A-11R-1965-07

TCGA-HE-7128-01A-11R-1965-07

TCGA-GL-A9DE-01A-11R-A37K-07
TCGA-GL-A9DD-01A-11R-A37K-07
TCGA-GL-A9DC-01A-11R-A37K-07
TCGA-GL-A59T-01A-21R-A28H-07
TCGA-GL-A59R-01A-11R-A26U-07
TCGA-GL-A4EM-01A-11R-A24Z-07
TCGA-GL-8500-01A-11R-2404-07
TCGA-GL-7966-01A-11R-2204-07
TCGA-GL-7773-01A-11R-A32Z-07
TCGA-GL-6846-01A-11R-1965-07
TCGA-G7-A8LE-01A-11R-A36F-07
TCGA-G7-A8LD-01A-11R-A36F-07
TCGA-G7-A8LC-01A-11R-A36F-07
TCGA-G7-A8LB-01A-11R-A36F-07
TCGA-G7-A4TM-01A-11R-A31O-07
TCGA-G7-7502-01A-11R-2204-07
TCGA-G7-7501-01A-11R-2204-07
TCGA-G7-6797-01A-11R-1965-07
TCGA-G7-6796-01A-11R-1965-07
TCGA-G7-6795-01A-11R-1965-07
TCGA-G7-6793-01A-11R-1965-07
TCGA-G7-6792-01A-21R-1965-07
TCGA-G7-6790-01A-11R-1965-07
TCGA-G7-6789-01A-11R-1965-07
TCGA-F9-A97G-01A-11R-A38C-07
TCGA-F9-A8NY-01A-11R-A36F-07
TCGA-F9-A7VF-01A-11R-A33Z-07
TCGA-F9-A7Q0-01A-11R-A36F-07
TCGA-F9-A4JJ-01A-11R-A24Z-07
TCGA-EV-5903-01A-11R-1592-07
TCGA-EV-5902-01A-11R-1592-07
TCGA-EV-5901-01A-11R-1592-07
TCGA-DZ-6135-01A-11R-1965-07
TCGA-DZ-6134-01A-11R-1965-07
TCGA-DZ-6133-01A-11R-1965-07
TCGA-DZ-6132-01A-11R-1965-07
TCGA-DW-7963-01B-11R-A28H-07
TCGA-DW-7842-01A-11R-A32Z-07
TCGA-DW-7841-01A-11R-A32Z-07
TCGA-DW-7840-01A-11R-A32Z-07
TCGA-DW-7839-01A-11R-2139-07
TCGA-DW-7838-01A-11R-2139-07
TCGA-DW-7837-01A-11R-2139-07
TCGA-DW-7836-01A-11R-2139-07

TCGA-DW-7834-01A-11R-2139-07

TCGA-DW-5561-01A-01R-1592-07

TCGA-DW-5560-01A-01R-1592-07

TCGA-BQ-7062-01A-11R-1965-07

TCGA-BQ-7061-01A-11R-1965-07

TCGA-BQ-7060-01A-11R-1965-07

TCGA-BQ-7059-01A-11R-1965-07

TCGA-BQ-7058-01A-11R-1965-07

TCGA-BQ-7055-01A-11R-1965-07

TCGA-BQ-7053-01A-11R-1965-07

TCGA-BQ-7051-01A-12R-1965-07

TCGA-BQ-7050-01A-11R-1965-07

TCGA-BQ-7048-01A-11R-1965-07

TCGA-BQ-7046-01A-11R-1965-07

TCGA-BQ-7045-01A-31R-1965-07

TCGA-BQ-7044-01A-11R-1965-07

TCGA-BQ-5894-01A-11R-1592-07

TCGA-BQ-5893-01A-11R-1592-07

TCGA-BQ-5892-01A-11R-1592-07

TCGA-BQ-5891-01A-11R-1592-07

TCGA-BQ-5890-01A-11R-1592-07

TCGA-BQ-5889-01A-11R-1592-07

TCGA-BQ-5888-01A-11R-1592-07

TCGA-BQ-5887-01A-11R-1965-07

TCGA-BQ-5886-01A-11R-1592-07

TCGA-BQ-5885-01A-11R-1592-07

TCGA-BQ-5884-01A-11R-1592-07

TCGA-BQ-5883-01A-11R-1592-07

TCGA-BQ-5882-01A-11R-1592-07

TCGA-BQ-5881-01A-11R-1592-07

TCGA-BQ-5880-01A-11R-1592-07

TCGA-BQ-5879-01A-11R-1592-07

TCGA-BQ-5878-01A-11R-1592-07

TCGA-BQ-5877-01A-11R-1592-07

TCGA-BQ-5876-01A-11R-1592-07

TCGA-BQ-5875-01A-11R-1592-07

TCGA-B9-A8YI-01A-21R-A37K-07

TCGA-B9-A8YH-01A-11R-A37K-07

TCGA-B9-A69E-01A-11R-A31O-07

TCGA-B9-A5W9-01A-11R-A28H-07

TCGA-B9-A5W8-01A-11R-A28H-07

TCGA-B9-A5W7-01A-11R-A31O-07

TCGA-B9-A44B-01A-11R-A24Z-07

TCGA-B9-7268-01A-11R-A32Z-07

TCGA-B9-5156-01A-01R-1592-07
TCGA-B9-5155-01A-01R-1592-07
TCGA-B9-4617-01A-01R-1193-07
TCGA-B9-4117-01A-02R-1351-07
TCGA-B9-4116-01A-02R-1351-07
TCGA-B9-4115-01A-01R-1193-07
TCGA-B9-4113-01A-01R-1193-07
TCGA-B3-A6W5-01A-12R-A33Z-07
TCGA-B3-8121-01A-21R-2404-07
TCGA-B3-4103-01A-02R-1351-07
TCGA-B3-3926-01A-02R-1351-07
TCGA-B3-3925-01A-02R-1351-07
TCGA-B1-A657-01A-11R-A31O-07
TCGA-B1-A656-01A-11R-A31O-07
TCGA-B1-A655-01A-11R-A31O-07
TCGA-B1-A654-01A-11R-A31O-07
TCGA-B1-A47N-01A-11R-A24Z-07
TCGA-B1-A47M-01A-11R-A24Z-07
TCGA-B1-7332-01A-11R-A32Z-07
TCGA-B1-5398-01A-02R-1592-07
TCGA-AT-A5NU-01A-11R-A28H-07
TCGA-AL-A5DJ-01A-11R-A26U-07
TCGA-AL-7173-01A-11R-2139-07
TCGA-AL-3472-01A-01R-1193-07
TCGA-AL-3471-01A-02R-1351-07
TCGA-AL-3466-01A-02R-1351-07
TCGA-A4-A7UZ-01A-12R-A355-07
TCGA-A4-A772-01A-11R-A33Z-07
TCGA-A4-A6HP-01A-11R-A31O-07
TCGA-A4-A5Y1-01A-11R-A28H-07
TCGA-A4-A5Y0-01A-11R-A31O-07
TCGA-A4-A5XZ-01A-11R-A31O-07
TCGA-A4-A5DU-01A-11R-A28H-07
TCGA-A4-A57E-01A-11R-A26U-07
TCGA-A4-A4ZT-01A-11R-A26U-07
TCGA-A4-A48D-01A-11R-A24Z-07
TCGA-A4-8630-01A-11R-2404-07
TCGA-A4-8518-01A-11R-2404-07
TCGA-A4-8517-01A-11R-2404-07
TCGA-A4-8516-01A-11R-2404-07
TCGA-A4-8515-01A-11R-2404-07
TCGA-A4-8312-01A-11R-2404-07
TCGA-A4-8311-01A-11R-2404-07
TCGA-A4-8310-01A-11R-2404-07

TCGA-A4-8098-01A-11R-2404-07
TCGA-A4-7997-01A-11R-2204-07
TCGA-A4-7996-01A-11R-2204-07
TCGA-A4-7915-01A-11R-2204-07
TCGA-A4-7734-01A-11R-A32Z-07
TCGA-A4-7732-01A-11R-2139-07
TCGA-A4-7585-01A-11R-2139-07
TCGA-A4-7584-01A-11R-2139-07
TCGA-A4-7583-01A-11R-A32Z-07
TCGA-A4-7288-01A-11R-A32Z-07
TCGA-A4-7286-01A-11R-A32Z-07
TCGA-5P-A9KF-01A-11R-A42S-07
TCGA-5P-A9KE-01A-11R-A42S-07
TCGA-5P-A9KC-01A-11R-A42S-07
TCGA-5P-A9KA-01A-11R-A42S-07
TCGA-5P-A9K9-01A-11R-A42S-07
TCGA-5P-A9K8-01A-11R-A42S-07
TCGA-5P-A9K6-01A-11R-A42S-07
TCGA-5P-A9K4-01A-11R-A42S-07
TCGA-5P-A9K3-01A-11R-A42S-07
TCGA-5P-A9K2-01A-11R-A42S-07
TCGA-5P-A9K0-01A-11R-A42S-07
TCGA-5P-A9JZ-01A-11R-A42S-07
TCGA-5P-A9JY-01A-11R-A42S-07
TCGA-5P-A9JW-01A-11R-A42S-07
TCGA-5P-A9JV-01A-12R-A42S-07
TCGA-5P-A9JU-01A-11R-A42S-07
TCGA-4A-A93Y-01A-11R-A37K-07
TCGA-4A-A93X-01A-11R-A37K-07
TCGA-4A-A93W-01A-11R-A37K-07
TCGA-2Z-A9JT-01A-11R-A42S-07
TCGA-2Z-A9JS-01A-21R-A42S-07
TCGA-2Z-A9JR-01A-12R-A42S-07
TCGA-2Z-A9JQ-01A-11R-A42S-07
TCGA-2Z-A9JP-01A-11R-A42S-07
TCGA-2Z-A9JN-01A-21R-A42S-07
TCGA-2Z-A9JM-01A-12R-A42S-07
TCGA-2Z-A9JL-01A-11R-A42S-07
TCGA-2Z-A9JK-01A-11R-A42S-07
TCGA-2Z-A9JJ-01A-11R-A42S-07
TCGA-2Z-A9JI-01A-11R-A42S-07
TCGA-2Z-A9JG-01A-11R-A42S-07
TCGA-2Z-A9JE-01A-11R-A42S-07
TCGA-2Z-A9JD-01A-11R-A42S-07

	TCGA-2Z-A9J8-01A-11R-A42S-07
	TCGA-2Z-A9J7-01A-11R-A38C-07
	TCGA-2Z-A9J6-01A-11R-A38C-07
	TCGA-2Z-A9J5-01A-21R-A38C-07
	TCGA-2Z-A9J3-01A-12R-A38C-07
	TCGA-2Z-A9J2-01A-11R-A38C-07
	TCGA-2Z-A9J1-01A-11R-A38C-07
	TCGA-2K-A9WE-01A-11R-A38C-07

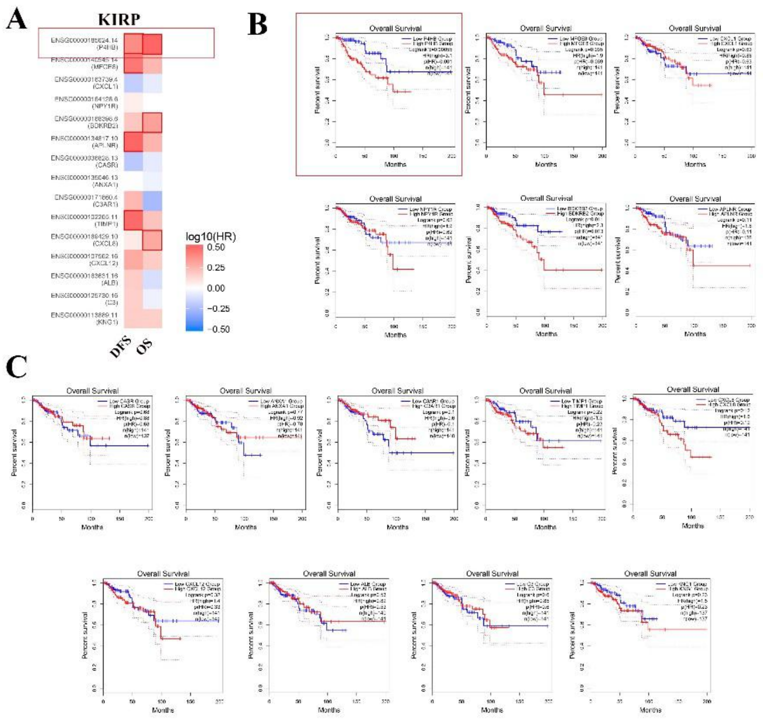

Figure S1

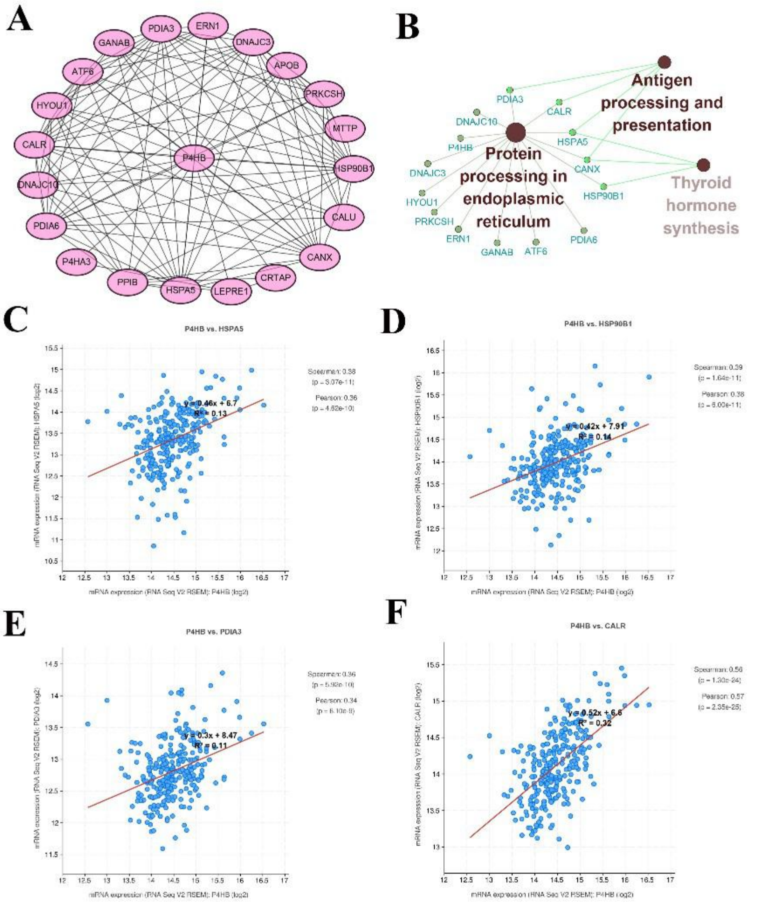
A
PDIA3
ERN1
GANAB
DNAJC3
ATF6
APOB
PRKCSH
HYOU1
MTTP
CALR
P4HB
HSP90B1
DNAJC10
CALU
PDIA6
CANX
P4HA3
CRTAP
PPIB
HSPA5
LEPRE1
B
Antigen processing and presentation
Protein processing in endoplasmic reticulum
Thyroid hormone synthesis
C
P4HB vs. HSPA5
y = 0.46x + 6.7
R² = 0.13
Spearman: 0.38 (p = 3.07e-11)
Pearson: 0.36 (p = 4.62e-10)
mRNA expression (RNA Seq V2 RSEM): HSPA5 (log2)
mRNA expression (RNA Seq V2 RSEM): P4HB (log2)
D
P4HB vs. HSP90B1
y = 0.42x + 7.91
R² = 0.14
Spearman: 0.39 (p = 1.64e-11)
Pearson: 0.38 (p = 6.00e-11)
mRNA expression (RNA Seq V2 RSEM): HSP90B1 (log2)
mRNA expression (RNA Seq V2 RSEM): P4HB (log2)
E
P4HB vs. PDIA3
y = 0.3x + 8.47
R² = 0.11
Spearman: 0.36 (p = 5.92e-10)
Pearson: 0.34
mRNA expression (RNA Seq V2 RSEM): PDIA3 (log2)
mRNA expression (RNA Seq V2 RSEM): P4HB (log2)
F
P4HB vs. CALR
y = 0.52x + 6.6
R² = 0.32
Spearman: 0.56 (p = 1.30e-24)
Pearson: 0.57 (p = 2.35e-25)
mRNA expression (RNA Seq V2 RSEM): CALR (log2)
mRNA expression (RNA Seq V2 RSEM): P4HB (log2)

Figure S2

Supplemental material description

Table S1 DEGs of KIRP in GSE11151 downloaded from the GEO dataset, including 19 pRCC samples and five normal samples.

Table S2 DEGs of KIRP in GSE15641 downloaded from the GEO dataset, containing 11 pRCC samples and 23 normal samples.

Table S3 DEGs between pRCC and normal kidney tissue in TCGA database screened through the GEPIA website.

Table S4 32 normal kidney tissues samples and 271 tumor samples downloaded from TCGA GDC data portal.

Figure S1. Survival analysis of Hub genes in PRCC.

(A) Analysis of Hub genes' OS and DFS in PRCC through TCGA database through DEPIA online website.

(B)(C) Analysis of the relevance of Hub genes and OS in PRCC through the DEPIA online website.

Figure S2. Pathway analysis and co-expression analysis of P4HB related genes.

(A) Analysis of genes have a PPI with P4HB via STRING website, and visualize PPI genes by Cytoscape software.

(B) P4HB related genes for KEGG analysis use CLUE GO plugin in Cytoscape.

(C-F) Co-expression analysis of genes involved in antigen processing and presentation in the KEGG pathway via cbioportal online tool.
