# Supplementary figures and images for: Identification of immunization-related new prognostic biomarkers for papillary renal cell carcinoma by integrated bioinformatics analysis
Source: BMC Med Genomics. 2021 Oct 7;14:241. doi: 10.1186/s12920-021-01092-w (PMC8499437; doi:10.1186/s12920-021-01092-w)

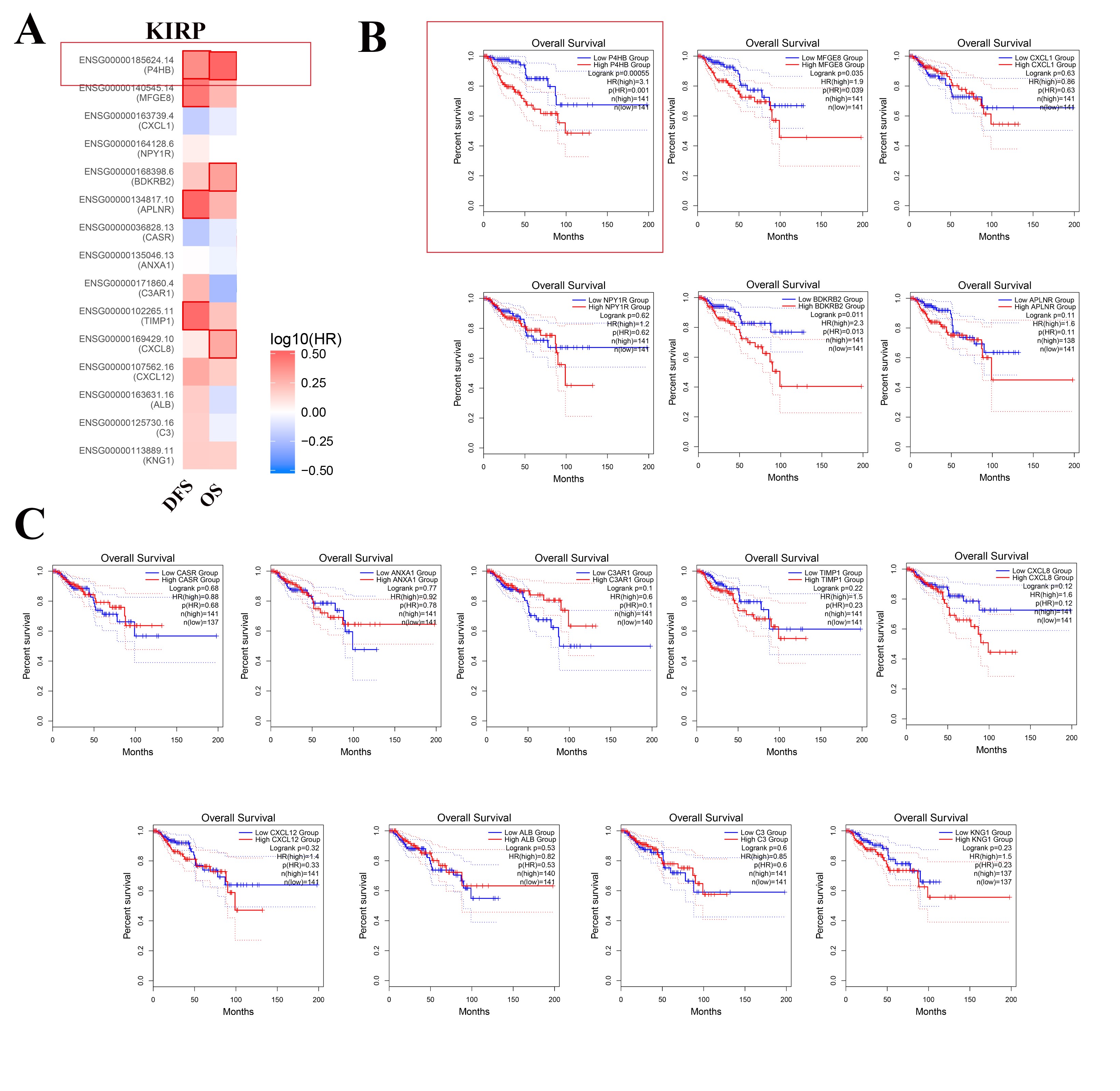

Supplement: Supplementary file 2 — Additional file 2: Figure S1. Survival analysis of Hub genes in PRCC. (A) Analysis of Hub genes' OS and DFS in PRCC through TCGA database through DEPIA online website. (B, C) Analysis of the relevance of Hub genes and OS in PRCC through the DEPIA online website. [file 12920_2021_1092_MOESM2_ESM.jpg]

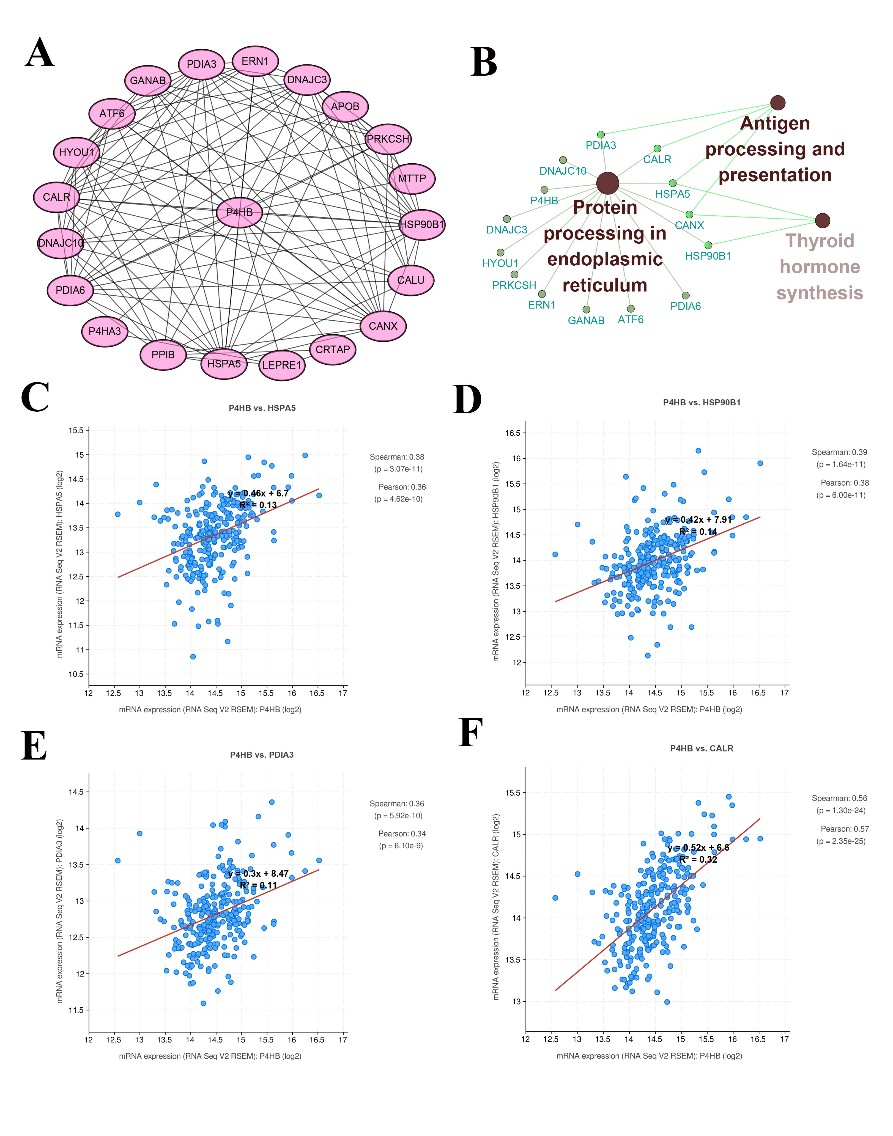

Supplement: Supplementary file 3 — Additional file 3: Figure S2. Pathway analysis and co-expression analysis of P4HB related genes. (A) Analysis of genes having a PPI with P4HB via STRING website, and visualization of the PPI genes by Cytoscape software. (B) P4HB related genes for KEGG analysis using CLUE GO plugin in Cytoscape. (C–F) Co-expression analysis of genes involved in antigen processing and presentation in the KEGG pathway via cbioportal online tool. [file 12920_2021_1092_MOESM3_ESM.jpg]
